# Characterization of biochemical properties of an apurinic/apyrimidinic endonuclease from *Helicobacter pylori*

**DOI:** 10.1371/journal.pone.0202232

**Published:** 2018-08-15

**Authors:** Aigerim Turgimbayeva, Sailau Abeldenov, Dmitry O. Zharkov, Alexander A. Ishchenko, Yerlan Ramankulov, Murat Saparbaev, Bekbolat Khassenov

**Affiliations:** 1 National Center for Biotechnology, Astana, Kazakhstan; 2 L.N. Gumilyov Eurasian National University, Astana, Kazakhstan; 3 Institute of Chemical Biology and Fundamental Medicine, SB RAS, Novosibirsk, Russia; 4 Novosibirsk State University, Novosibirsk, Russia; 5 Groupe «Réparation de l’ADN», Equipe Labellisée par la Ligue Nationale Contre le Cancer, CNRS UMR8200, Université Paris-Sud, Gustave Roussy Cancer Campus, Villejuif, France; 6 School of Science and Technology Nazarbayev University, Astana, Kazakhstan; Hopital Maisonneuve-Rosemont, University of Montreal, CANADA

## Abstract

Apurinic/apyrimidinic (AP) endonucleases play critical roles in the repair of abasic sites and strand breaks in DNA. Complete genome sequences of *Helicobacter pylori* reveal that this bacterial specie has a single AP endonuclease. An *H*. *pylori* homolog of Xth (HpXth) is a member of exonuclease III family, which is represented by *Escherichia coli* Xth. Currently, it remains unknown whether this single AP endonuclease has DNA repair activities similar to those of its counterpart in *E*. *coli* and other bacteria. We report that HpXth possesses efficient AP site cleavage, 3’-repair phosphodiesterase, and 3’-phosphatase activities but not the nucleotide incision repair function. Optimal reaction conditions for HpXth’s AP endonuclease activity are low ionic strength, high Mg^2+^ concentration, pH in the range 7–8, and temperature 30 °C. The kinetic parameters measured under steady-state conditions showed that HpXth removes the AP site, 3’-blocking sugar-phosphate, and 3’-terminal phosphate in DNA strand breaks with good efficiency (*k*_cat_/*K*_M_ = 1240, 44, and 5,4 μM^–1^·min^–1^, respectively), similar to that of *E*. *coli* Xth. As expected, the presence of HpXth protein in AP endonuclease—deficient *E*. *coli xth nfo* strain significantly reduced the sensitivity to an alkylating agent and H_2_O_2_. Mutation of active site residue D144 in HpXth predicted to be essential for catalysis resulted in a complete loss of enzyme activities. Several important structural features of HpXth were uncovered by homology modeling and phylogenetic analysis. Our data show the DNA substrate specificity of *H*. *pylori* AP endonuclease and suggest that HpXth counteracts the genotoxic effects of DNA damage generated by endogenous and host-imposed factors.

## Introduction

Cellular DNA is continuously and inevitably assaulted by a variety of endogenous and environmental genotoxic agents. Reactive oxygen species (ROS) produced by aerobic respiration generate oxidized bases, sugars, and DNA strand breaks [1–2]. In addition, hydrated DNA is prone to spontaneous decomposition such as base loss, resulting in apurinic/apyrimidinic (AP) sites, and hydrolytic deamination, yielding mostly uracil and hypoxanthine [3]. The majority of endogenous DNA base lesions and single-strand breaks with 3’-blocking groups is removed through two overlapping pathways: base excision repair (BER) and nucleotide incision repair (NIR) [4–6]. The common BER pathway consists of two sequential excision or incision steps. First, one of many DNA glycosylases hydrolyzes the *N*-glycosidic bond of the damaged base and produces either an AP site or a single-stranded DNA break with a single-nucleotide gap flanked by a 5’-phosphate and 3’-blocking group, which may be a phosphate or an unsaturated aldehyde remnant of deoxyribose [7–9]. In the second step of BER, an AP endonuclease cleaves 5′ to the AP site or the 3’-blocking groups, leaving a free 3’-hydroxyl and either a 5’-terminal deoxyribose phosphate moiety, or a single-nucleotide gap with a proper 5’-phosphate [10]. Although BER can handle the majority of oxidized DNA bases, some lesions, such as α-anomers of deoxynucleosides (αdN) generated by free radicals under anoxic conditions, are not repaired by DNA glycosylases but rather by AP endonucleases via the NIR pathway [11–13]. In this glycosylase-independent pathway, an AP endonuclease directly cleaves DNA 5’ to the lesion and generates a single-strand break with a 5’-dangling damaged nucleotide [5, 14]. AP endonucleases also use their 3’-repair phosphodiesterase function to clean single-strand DNA breaks with “dirty ends” generated either by ROS or by bifunctional DNA glycosylase/AP lyases, producing 3’-hydroxyl termini that can be used by DNA polymerases to initiate the repair synthesis [15–16]. Noteworthy, human major AP endonuclease 1, APE1, in addition to NIR and BER functions contains abasic RNA cleavage activity, including the ability to process abasic ribonucleotides embedded in DNA [17].

Despite this multitude of functions, both the primary activity (AP site cleavage) and less efficient but also important 3’-phosphodiesterase, 3’→5’ exonuclease, 3’-phosphatase, and NIR activities of AP endonucleases are catalyzed by a single active site [15, 18–21]. All known AP endonucleases are grouped in two distinct superfamilies based on their structural and sequence similarity to either *Escherichia coli* exonuclease III (Xth; Endonuclease—Exonuclease—Phosphatase superfamily, or EEP) or endonuclease IV (Nfo) [10]. AP endonucleases from the EEP superfamily, including *E*. *coli* Xth and human APE1, require Mg^2+^ or other divalent metal ions for catalysis. Both Xth and Nfo homologs are present in prokaryotes and eukaryotes, and have been biochemically characterized in *E*. *coli*, *Saccharomyces cerevisiae*, *Caenorhabditis elegans*, and some other species [16, 22]. Not all AP endonucleases have all repair functions; for example, human APE1 contains very weak 3′-phosphatase activity, whereas the NIR activity in bacteria is generally present in homologs of Nfo, but not of Xth. Importantly, bacterial AP endonucleases as key genome protection enzymes are required for successful infection in view of host defense systems, thus representing a promising target for antimicrobial drugs [23–26].

*Helicobacter pylori* is a gram-negative bacterium that colonizes human gastric mucosa. The presence of *H*. *pylori* is sometimes pathogenic causing peptic ulcers and gastroesophageal reflux disease but may also have beneficial effects such as alleviation of childhood asthma and other allergies [27]. The genome of *H*. *pylori* can undergo dramatic changes that may help this bacterium to escape from host immune surveillance. Surprisingly, as compared to *E*. *coli* and non-symbiotic bacteria, *H*. *pylori* has a limited number of genes, with little redundancy in the BER pathway [28]. Whole-genome sequencing has found a single AP endonuclease, XthA (hereafter referred to as HpXth), homologous to *E*. *coli* Xth, encoded by ORF HP1526 [29–30]. Cell-free extracts from the mutant *H*. *pylori* strain with disrupted ORF HP1526 no longer have Mg^2+^-dependent AP site cleavage activity [31]. The *H*. *pylori* single *xthA* and double *xthA mutY* mutant strains show a four- and 37-fold increase in the spontaneous mutation rate in comparison with the wild-type control [32]. Taken together, these data strongly indicate that HpXth is involved in the repair of spontaneously occurring AP sites and other mutagenic DNA lesions *in vivo*. Nevertheless, at present, detailed biochemical characterization of this enzyme is lacking.

Since *H*. *pylori* seems to lack Nfo homologs, it was interesting to define the spectrum of DNA repair activities in its only AP endonuclease, HpXth. Here, we demonstrate that HpXth possesses AP site cleavage and 3’-repair phosphodiesterase functions. The kinetic parameters of HpXth-catalyzed repair activities were measured and compared to those of homologous AP endonucleases. The ability of HpXth to rescue an AP endonuclease—deficient *E*. *coli* strain after exposure to an alkylation agent and H_2_O_2_ was studied. The evolution, mechanisms of DNA damage recognition, and biological roles of HpXth in the defence against spontaneous and host-induced damage to bacterial DNA are discussed.

## Materials and methods

### Bacterial strains, plasmids, and reagents

The plasmids pET28c(+) (Novagen, UK) and pBluescript II SK+ (further referred as pBSK) were used to construct the expression vector. The plasmid pBW21 encoding the *E*. *coli* Nfo proteins was generously provided by Dr. B. Weiss (Emory University School of Medicine, Atlanta, GA). Restriction enzymes, T4 DNA ligase, and Phusion High-Fidelity DNA Polymerase (Thermo Scientific, USA) were employed for the amplification and cloning of the target gene. H_2_O_2_ and Methyl methanesulfonate (MMS) were purchased from Sigma-Aldrich Chimie S.a.r.l. (Lyon, France). *E*. *coli* strain BH110 (*nfo*::*kan*^R^ [Δ(*xthA-pncA*)*90X*::*Ƭn10*]) comes from the laboratory stock, and *E*. *coli* expression strain Rosetta 2 (DE3) was acquired from (Novagen, Merck4Biosciences, France). *E*. *coli* BH110 was lysogenized with a helper phage (λDE3) harboring a copy of the T7 RNA polymerase gene, using the DE3 lysogenization kit (Novagen, Merck4Biosciences, France). The lysogenized *E*. *coli* BH110 (DE3) cells were transformed with pBSK, pBW21-Nfo, pBSK-HpXth and pBSK-HpXth-D144N vectors.

### Cloning and expression of *H*. *pylori* AP endonuclease in *E*. *coli* and purification of the HpXth protein

Genomic DNA of *H*. *pylori* was provided by the Republican Collection of Microorganisms (Astana, Kazakhstan). Reference gene sequence of *xthA* of *H*. *pylori* was retrieved from the published genome sequences: CP011330.1 (range: 1144587–1145339). The following PCR primers (shown in the 5’→3’ direction) were applied to amplify the target HpXth cDNA: HpXth-NdeI, d(GGGAATTCCATATGAAACTGATTTCATGGAATGTGAAC) and HpXth-BamHI, d(CGCGGATCCGTTAAACTAATTCCAACCCTACCGG). The ORF of *XthA* from *H*. *pylori* was cloned into the pET28c(+) vector at the *Nde*I/*BamH*I sites, resulting in expression plasmid pET28c/HpXth. The encoded HpXth protein carries an N-terminal 6xHis-tag sequence. The DNA sequence of plasmid insert was verified against the sequence from the GenBank database in the Vector NTI software Advance^™^ v11.0 (Invitrogen, USA).

Site-directed mutation D144N in the coding sequence of HpXth in plasmid vector pET28c/HpXth and pBlueScript II SK+/HpXth were generated with the QuickChange site-directed mutagenesis kit (Quickchange^®^ XL, Stratagene). The following oligonucleotide primers (shown in the 5’→3’ direction) were used to generate HpXth-D144N mutant protein: HpXth-F-D144N, d(GTCATTGTGTGTGGG**AAC**TTGAATGTGGCCC); HpXth-R-D144N, d(GGGCCACATTCAA**GTT**CCCACACACAATGAC).

To express the recombinant proteins, *E*. *coli* Rosetta 2 (DE3) cells were transformed with plasmids, and the isolated Kn^R^ transformants were grown in the Luria-Bertani (LB) broth with 50 μg/mL kanamycin. At optical density at 600 nm (OD_600_) of 0.6, the cells were induced by 0.5 mM isopropyl β-D-1-thiogalactopyranoside (IPTG), and incubation was continued at room temperature with shaking (100 rpm) overnight. The induced cells were collected by centrifugation at 6000 × *g* for 7 min at 4 °C. The bacterial pellet containing HpXth was resuspended in a buffer consisting of 20 mM NaCl and 20 mM Tris-HCl (pH 8.0) and supplemented with the Complete Protease Inhibitor Cocktail (Roche Diagnostics, Switzerland). The cells were disrupted by a French press at 18,000 psi (1250 bar), and then sonicated on ice at 40% amplitude. The lysate was cleared by centrifugation at 40,000 × *g* for 60 min at 4 °C. The supernatant was adjusted to 500 mM NaCl and 20 mM imidazole and loaded onto a 1 mL HiTrap Chelating HP column (GE Healthcare) charged with Ni^2+^. The bound proteins were eluted in a 20–500 mM imidazole gradient and the fractions containing a recombinant HpXth protein were pooled and loaded onto a 1 mL HiTrap Heparin column (GE Healthcare). The bound proteins were eluted with a 50–1000 mM NaCl gradient. Fractions containing the recombinant protein were stored at -20 °C in 50% glycerol.

### Oligonucleotides

All the oligodeoxyribonucleotides with modifications and their regular complementary counterparts were purchased from Eurogentec (Seraing, Belgium) and are presented in the 5’→3’ direction. They included the 30mer X-RT d(TGACTGCATAXGCATGTAGACGATGTGCAT) where X is tetrahydrofuran (THF, a synthetic analog of an abasic site), α-2’-deoxyadenosine (αdA), 5-hydroxycytosine (5ohC), or 5,6-dihydrouracil (DHU), and complementary 30mer oligonucleotides containing dA, dG, dC, or T opposite the lesion. DNA substrates were constructed as described previously [26, 33]. Briefly, the complementary oligonucleotides were annealed; the resulting duplex oligonucleotides are referred to as X•C (G, A, T), respectively, where X is a modified residue. The DNA sequence contexts have previously been used to study the DNA substrate specificity of bacterial, yeast, and plant AP endonucleases [26, 34–35]. Chemical structures of DNA adducts used in this work are shown in S1 Fig.

DNA end labeling were performed as described previously [26]. Briefly, oligonucleotides were either 5’-end labeled by T4 polynucleotide kinase (or by phosphatase minus mutant PNK) (New England Biolabs, OZYME, France) in the presence of γ/^32^P]ATP (3000 Ci·mmol^−1^) (PerkinElmer SAS, France) or 3’-end labeled by terminal transferase (New England Biolabs) in the presence of α[^32^P]3’-dATP (cordycepin 5’-triphosphate, 5000 Ci·mmol^–1^) (PerkinElmer) as recommended by the manufacturers. The labeled oligonucleotides were annealed to their appropriate complementary oligonucleotides in a buffer consisting of 50 mM KCl and 20 mM HEPES-KOH (pH 7.5) at 65 °C for 3 min and cooled down to room temperature for 2 h. The resulting duplex oligonucleotides are referred to as X•C (G, A, T), respectively, where X is a modified residue.

The following oligonucleotides were used to measure 3’→5’ exonuclease and 3’-repair phosphodiesterase activities: Exo20, d(GTGGCGCGGAGACTTAGAGA); Exo20^THF^, d(GTGGCGCGGAGACTTAGAGAX), where X is 3’-terminal THF; Exo20^P^, d(GTGGCGCGGAGACTTAGAGAp), where p is 3’-terminal phosphate; 5P-Exo19, d(pATTTGGCGCGGGGAATTCC), where p is 5’-terminal phosphate; and complementary Rex-T, d(GGAATTCCCCGCGCCAAATTTCTCTAAGTCTCCGCGCCAC) containing T opposite of THF. The recessed duplex Exo20•RexT^Rec^ was composed of Rex-T and Exo20, whereas nicked duplexes Exo20^THF^•RexT^Nick^ and Exo20^P^•RexT^Nick^ were composed of 5P-Exo19 and Rex-T, and Exo20^THF^ or Exo20^P^, respectively. Structures of the nicked and recessed duplex oligonucleotides used in the present work are shown in S1 Fig. To measure the 3’-phosphatase activity of the AP endonucleases, we prepared a 20mer (Exo20) oligonucleotide containing a 3’-terminal radioactive phosphate residue: ^32^P, as described [26]. Previously, we have shown that human tyrosyl-DNA phosphodiesterase 1 (Tdp1) can remove a 3’-terminal cordycepin nucleoside, thereby producing an oligonucleotide fragment with a phosphate residue at the 3’ end [36]. For this purpose, Exo20 was 3’-end labeled with cordycepin [α-^32^P]3’-dATP to obtain a 21mer Exo20-[^32^P]3’-dAMP fragment, which was then treated with recombinant Tdp1 (generously provided by Prof. Olga Lavrik, Novosibirsk, Russia). The resulting 20mer Exo20-3’-[^32^P] fragment and 5P-Exo19 oligonucleotide were annealed to the complementary Rex-T oligonucleotide, and the resultant 3’-[^32^P]labeled Exo20^p^•RexT^Nick^ nicked duplex oligonucleotide served as the DNA substrate to measure the 3’-phosphatase activity of AP endonucleases under steady-state reaction conditions.

### DNA repair assays

The standard reaction buffer (20 μL) for measuring HpXth’s DNA repair activities consisted of 10 nM [^32^P]labeled duplex oligonucleotide carrying a modified residue, 5 mM MgCl_2_, 25 mM KCl, 50 mM Tris-HCl (pH 8.0), 0.01% Nonidet P-40, 0.05 mM DTT, 0.1 mg·mL^−1^ BSA, and 0.2 nM enzyme, with incubation at 30 °C for 5 min, unless specified otherwise. To measure the AP site cleavage activity of human APE1, we used the buffer BER (20 μL) which consisted of 10 nM [^32^P]labeled THF•T duplex oligonucleotide, 100 mM KCl, 5 mM MgCl_2_, 10 mM Tris-HCl (pH 8.0), 0.01% Nonidet P-40, 0.05 mM DTT, 0.1 mg·mL^−1^ BSA, and 50 nM enzyme, with incubation at 37 °C for 1 h, unless specified otherwise. To measure nucleotide incision activities toward DNA base modifications, we applied the buffer NIR (20 μL), optimal for nucleotide incision activity of human APE1, which consisted of 10 nM [^32^P]labeled αdA•T duplex oligonucleotide, 25 mM KCl, 0.1 mM MgCl_2_, 50 mM HEPES-KOH (pH 6.8), 0.01% Nonidet P-40, 0.05 mM DTT, 0.1 mg·mL^–1^ BSA, and a limited amount of an enzyme, with incubation at 37 °C for 1 h, unless specified otherwise.

The kinetic parameters of repair reactions were measured as described [37]. Briefly, 10–6000 nM duplex oligonucleotide substrate was incubated under the appropriate reaction conditions. The reaction products were quantified on a PhosphorImager after separation by denaturing polyacrylamide gel electrophoresis (PAGE). The kinetic constants were measured at the initial velocity, and *K*_M_ and *k*_cat_ values were determined by fitting the data to the one-site binding model in Prism 4 (GraphPad Software, USA). The kinetic parameters for AP endonuclease-catalyzed 3’→5’ exonuclease reaction were determined by quantifying the reaction products expressed as a percentage of the total substrate. When an exonuclease generated multiple DNA products, the value obtained for each degradation fragment was multiplied by the number of catalytic events required for its formation, and the total exonuclease activity was calculated as the sum of these products.

The products of the reactions were analyzed as described [26]. Briefly, all reactions were stopped by adding 10 μL of a solution consisting of 0.5% SDS and 20 mM EDTA and then desalted by passing through homemade spin-down columns filled with Sephadex G25 (GE Healthcare) equilibrated in 7.5 M urea. The desalted reaction products were separated by electrophoresis in a denaturing 20% (w/v) polyacrylamide gel (7.5 M urea, 0.5× TBE). A Fuji Phosphor Screen was exposed to the gels and then scanned with Fuji FLA-9500 and analyzed in the Image Gauge v.4.0 software. At least three independent experiments were conducted for all quantitative and kinetic measurements and also gel images.

### Preparation of anti-HpXth antibodies and Western blotting

To produce polyclonal antibodies, a six-month-old rabbit was immunized with the recombinant purified HpXth protein. On the first day of immunization, 0.3 mg of the HpXth protein diluted in phosphate buffer was injected subcutaneously in the back of the rabbit with complete Freund’s adjuvant. After 7 days, a rabbit was immunized with 0.15 mg of the HpXth protein in the same buffer. The third, fourth and fifth immunizations with 0.15 mg of the antigen were carried out every 7 days. Two days later, the sixth immunization was carried out, and the next day the blood serum was collected and the production of anti-HpXth antibodies was checked by immunoblotting. Animal maintenance and experimental procedures were in accordance with the Kazakhstan national regulations based on the provisions of the Central Ethics Commission of Ministry of Health of the Republic of Kazakhstan (Astana, Kazakhstan) and the Legislation of the European Convention for the Protection of Vertebrate Animals Used for Experimental and other Scientific Purposes (ETS 123, Strasbourg, 1986). The protocol used in the present study was approved on January 5, 2016 by the Local Ethical Commission of the RSE "National Center for Biotechnology", Astana, Kazakhstan.

Western blot was performed according to the standard protocol [38]. Briefly, four μg of the cell-free extracts from *E*. *coli* BH110 (DE3) strains harboring plasmids expressing the *E*. *coli* Nfo and *H*. *pylori* HpXth and HpXth-D144N proteins were separated in a 12% (w/v) SDS-PAGE and electro-transferred to the PVDF membrane. To verify protein transfer the membrane was stained with Ponceau S dye [0.1% (w/v) Ponceau S in 5% (v/v) acetic acid]. After membrane blocking with 5% (w/v) dry milk in Tween 20/Tris-buffered saline (TBST, 50 mM Tris-HCl, pH 7.6, 150 mM NaCl, 0.1% [w/v] Tween 20), the HpXth protein was detected with rabbit anti-HpXth polyclonal antibodies (1:5000) as primary antibodies and goat anti-rabbit horseradish peroxidase-conjugated antibody (Sigma-Aldrich Chimie S.a.r.l., Lyon, France) (1:10,000) as secondary antibodies. The bands were detected by chemiluminescent substrate (Applichem GmbH, Darmstadt, Germany) and exposed to an X-ray film (AgfaPhoto GmbH, Germany).

### Alkylation and oxidative DNA damage sensitivity

Drug treatment was performed as previously described [26]. In brief, overnight bacterial cultures were diluted 100-fold in LB broth containing 150 μg/mL ampicillin and 0.05 mM IPTG and incubated at 28 °C until the mid-exponential phase (OD_600_ = 0.6). The cells were collected by centrifugation, washed once, and resuspended in phosphate-buffered saline (PBS). Ten-fold serial dilutions were prepared in PBS as well. Alkylating agent MMS was added to 2.5 mL of molten soft agar (0.6% agar in LB) at 46 °C containing 1 mM IPTG, followed immediately by 0.25 mL of each cell dilution, and the mixture was poured onto the surface of a 1.5% solid LB agar (25 mL) plate. For an oxidizing-agent sensitivity test, each cell dilution was exposed to 10 mM H_2_O_2_ for different periods, and then the cells were mixed with molten soft agar and poured onto LB plates. Colonies were scored after 1–2 days of incubation at 37 °C. Data used in survival curve construction and analysis are representative of at least three independent experiments.

### Phylogenetic analysis and model building

The sequences of the conserved domains of *E*. *coli* Xth, human APE1 and APE2, *Neisseria meningitidis* Nape, and *Methanothermobacter thermautotrophicus* Mth212 were used to search the nonredundant NCBI database of protein sequences by means of BLAST [39]. One representative sequence from each family per taxonomic class was selected for analysis, unless the same species had two paralogs from the same family, in which case, both were selected. Structural model of HpXth was built by multiple iterative threading with searching of the full PDB database using I-TASSER [40]. All sequence alignments and phylogenetic trees were built in Clustal Omega [41]. The conservation analysis was carried out with the Taylor set of amino acid properties [42] and the hierarchical analysis approach outlined elsewhere [43], except that the conservation at any position was expressed as the mean of the number of crossed borders in the Venn diagrams covering all sequence pairs in the compared alignments and normalized to the number of pairs. Images were prepared in the PyMol software (Schrödinger, New York, NY). Phylogenetic trees were visualized using iTOL [44].

### Liquid chromatography with tandem mass spectrometry (LC-MS/MS)

Electrophoretic separation of proteins was performed by SDS-PAGE in a 4–14% Bis-Tris gradient gel. The protein bands were excised from the gel and processed as described previously [45–46]. Next, 15 μL of 190 mM ammonium bicarbonate was added to 100 μL of 13.33 ng/μL trypsin (Promega, V5280) for activation of the enzyme, and the samples were incubated at 37°C for 16 h. The peptide mixture was next purified in ZipTip_C18_ pipette tips (Millipore, 0.6 μL bed, ZTC18S096). The peptide-containing supernatants were dried at 35 °C in a SpeedVac for 30 min, then resuspended in 10 μL of 0.1% trifluoroacetic acid for mass spectrometry experiments.

We used a trapping column setup (Acclaim PepMap100 C18 precolumn, 5 mm × 300 μm; 5 μm particles; Thermo Scientific) and a Dionex high-performance liquid chromatography pump (Ultimate 3000 RSLCnano System, Thermo Scientific). For this experiment, peptides were separated on a Acclaim Pep-Map RSLC column (15 cm × 75 μm, 2 μm particles; Thermo Scientific) with a 75 min multistep acetonitrile gradient (Buffer A: 0.1% formic acid; buffer B 90% acetonitrile/10% H_2_O in 0.1% formic acid) at a flow rate of 0.3 μL/min, according to the following time table: 0 min, 2% B; 10 min, 2% B; 58 min, 50% B; 59 min, 99% B; 69 min, 99% B; 70 min, 2.0% B; 75 min, 2.0% B. The unmodified CaptiveSpray ion source (Capillary 1300 V, dry gas 3.0 L/min, dry temperature 150 °C) was employed to interface the LC system with an Impact II (Bruker). For quantification, full-scan MS spectra were acquired at a spectral rate of 2.0 Hz followed by acquisition of one MS/MS spectrum. For data acquisition from the sample, the two most abundant precursor ions were selected for fragmentation, resulting in a total cycle time of 3 s. The mass range of the MS scan was set to extend from m/z 150 to 2200 in positive ion polarity mode.

We used Mascot software to perform searches against the SwissProt 2014_08 database (546,238 sequences; 194,363,168 residues). Search parameters were set as follows: variable modifications, oxidation (M), fragment ion mass tolerance, 20 ppm; parent ion tolerance, 20 ppm.

## Results

### cDNA cloning and purification of the HpXth protein

The cDNA coding for AP endonuclease HpXth was prepared by PCR from the DNA of *H*. *pylori*, and oligonucleotide primers as described in Materials and Methods. The gel-purified PCR 753 bp fragment was inserted into pET28c(+) to create pET28c/HpXth expression plasmid. Competent *E*. *coli* DH5α cells were transformed with the ligation mixture, and plasmids containing inserts were isolated from the transformant colonies. The primary structures of inserts in the plasmids were verified by sequencing. A comparison of the cloned cDNA with the respective reference sequences present in GenBank revealed polymorphism in HpXth, manifested in the form of amino acid substitution: G181D. The calculated molecular mass of His-tagged HpXth is 31.6 kDa.

To characterize the DNA repair activities of the recombinant HpXth protein, we affinity-purified the bacterial AP endonuclease from the *E*. *coli* Rosetta 2 (DE3) strain expressing the N-terminal His-tagged form of HpXth as described in Materials and Methods. After that, the homogeneity of protein preparations was assessed by SDS-PAGE in a 12% gel, revealing >95% purity of the HpXth preparations (S2 Fig). To confirm the identity of the purified protein, the bands were excised from the gel and in-gel digested with trypsin. The resulting peptide mixtures were subjected to matrix-assisted laser desorption ionization-time of flight MS and electrospray ionization tandem mass spectrometry, respectively, and individual peptide sequences were identified based on their molecular weights. Most of the identified peptides matched the expected amino acid sequences of the HpXth protein, with ~80% sequence coverage of the protein. These results indicated that the purified protein is indeed the AP endonuclease from *H*. *pylori*.

### Characterization of AP site cleavage activity of the *H*. *pylori* AP endonuclease in the presence of metal cofactors

**Fig 1:**
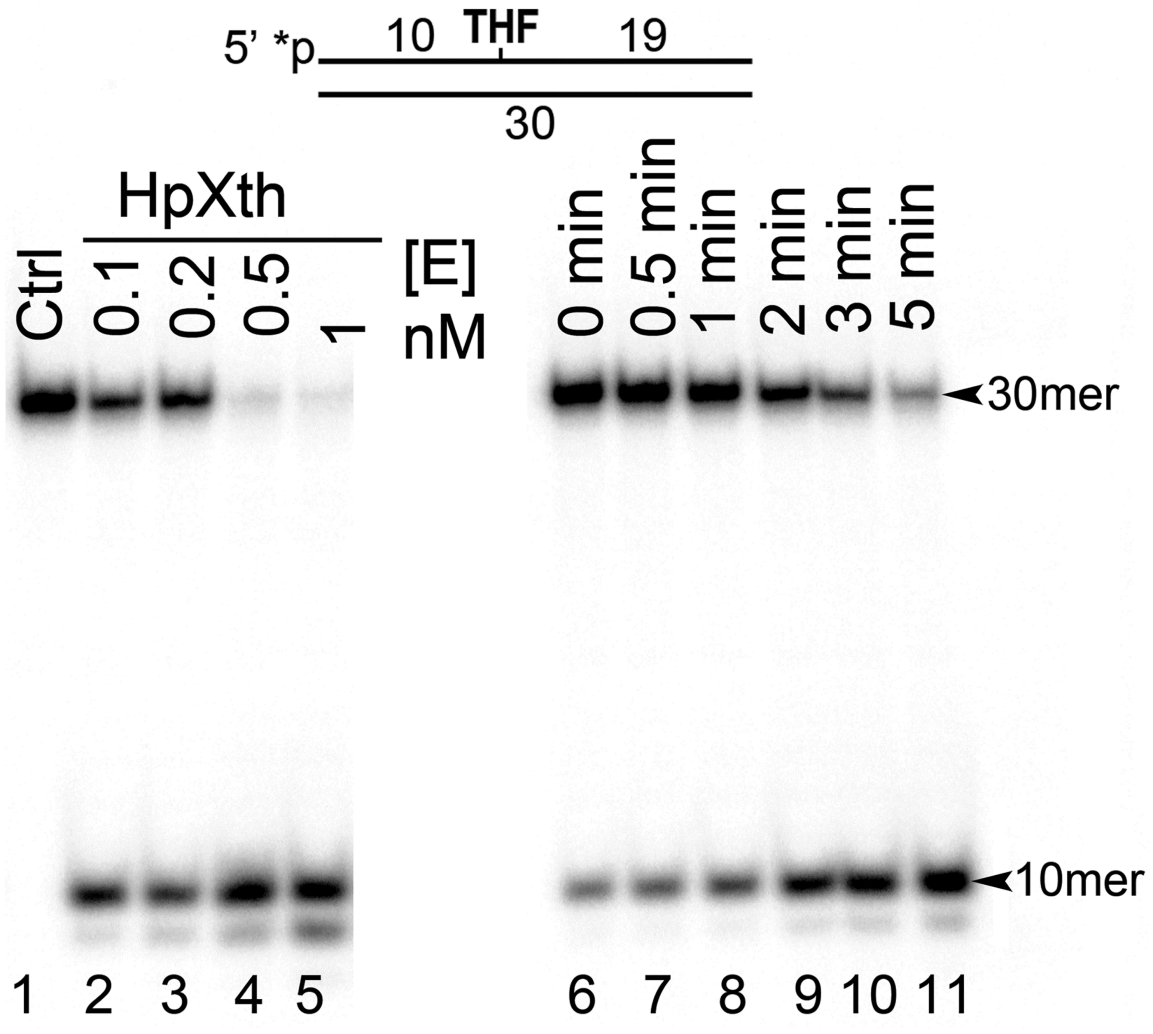
Purified *H*. *pylori* AP endonuclease, HpXth, has an AP site cleavage activity. In brief, 10 nM 5’-[^32^P]labeled 30mer THF•T duplex was incubated with various concentrations of HpXth for 0–5 min at 30 °C in the standard reaction buffer. Denaturing PAGE analysis of products of the reaction. Lane 1, control THF•T, no enzyme; lanes 2–5, as in lane 1 but 0.1, 0.2, 0.5, and 1 nM HpXth with incubation for 5 min, respectively; lanes 6–11, as in lane 1 but incubated with 0.5 nM HpXth for 0, 0.5, 1, 2, 3, or 5 min, respectively. For details, see Materials and methods.

Previous study has shown Mg^2+^-dependent AP site cleavage activity in a cell-free extract of *H*. *pylori* dependent on the function of gene *XthA* coding for a homolog of *E*. *coli* Xth [31]. Nonetheless, it remained unclear whether the HpXth protein, the product of *XthA*, has efficient AP endonuclease and other DNA repair activities. To clarify this issue, we measured incision activities of the purified HpXth protein on the 5’-[^32^P]labeled 30mer oligonucleotide duplex THF•T, containing THF, an abasic site analog, at position 11, in the presence of 5 mM MgCl_2_. As shown in Fig 1, incubation of THF•T with HpXth generated a fast-migrating 10mer cleavage product (lanes 2–11) indicating the presence of AP endonuclease activity. HpXth exhibited a very efficient AP site cleavage activity because 0.5 nM enzyme was able to cleave >95% of 10 nM oligonucleotide substrate after only 5 min incubation under our experimental conditions (lanes 4 and 11). Incubation of THF•T with 1 nM HpXth generated the second minor cleavage product migrating similarly to the 9mer fragment (lane 5) suggesting degradation of the initial 10mer cleavage product by the 3’→5’ exonuclease activity of the enzyme.

**Fig 2:**
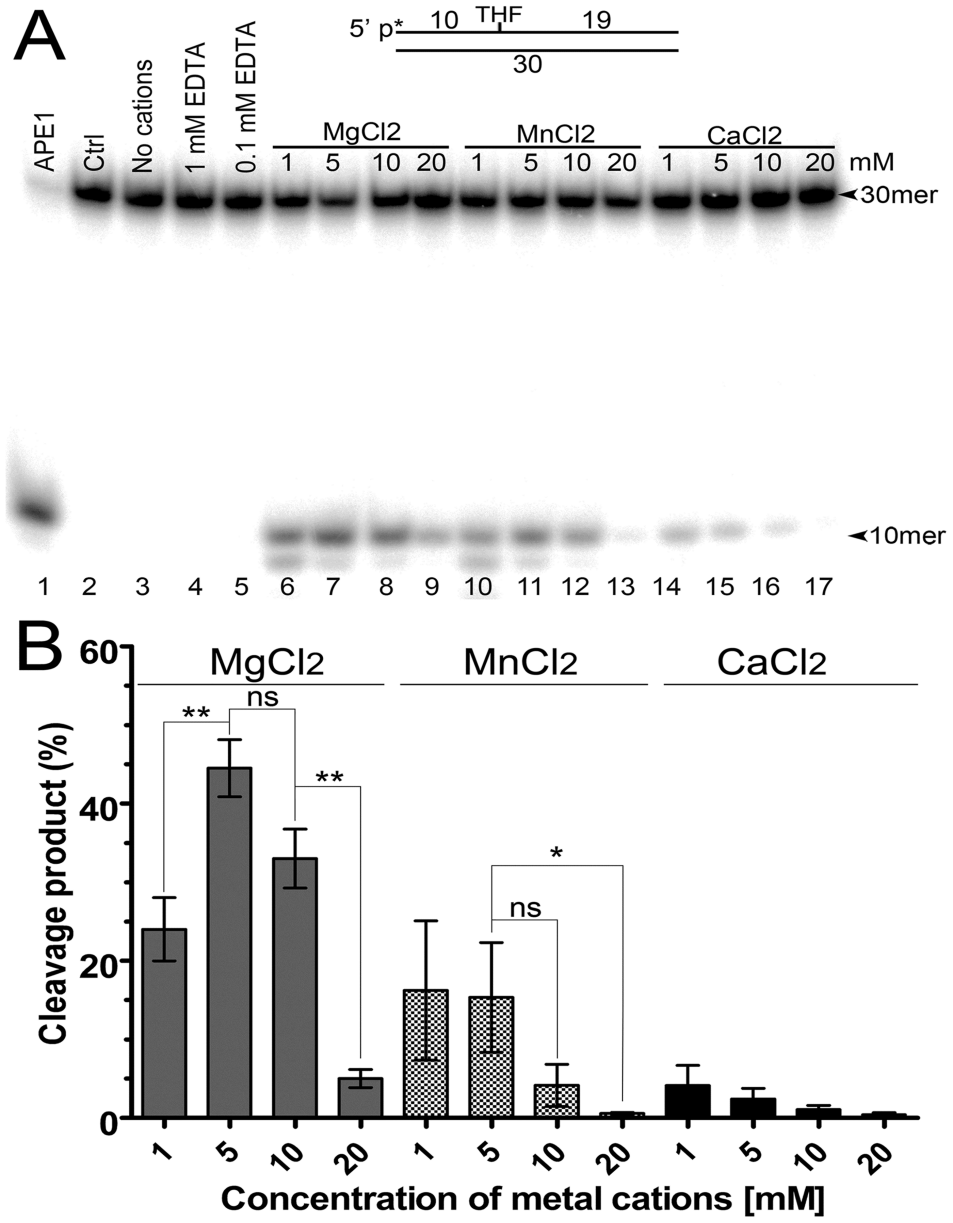
Divalent-metal ion dependence of HpXth’s AP site cleavage activity. In brief, 10 nM 5’-[^32^P]labeled 30mer THF•T duplex was incubated with 0.2 nM HpXth for 5 min at 37 °C in the standard reaction buffer containing various concentrations of metal cations. (**A**) Denaturing PAGE analysis of products of the reaction. Lane 1, THF•T and 0.1 nM APE1 in buffer BER; lane 2, control THF•T, no enzyme; lane 3, as in lane 2 but HpXth in the standard reaction buffer but without cations; lanes 4 and 5, as in lane 3 but 1 and 0.1 mM EDTA, respectively; lanes 6–9, as in lane 3 but 1, 5, 10, and 20 mM MgCl_2_, respectively; lanes 10–13, as in lanes 6–9 but 1–20 mM MnCl_2_; lanes 14–17, as in lanes 6–9 but 1–20 mM CaCl_2_. The arrows denote the position of the 30mer substrate and 10mer cleavage product. (**B**) Graphical representation of data from panel A. The statistical significance of the differences among the mean values were evaluated using two-tailed Student’s test (***P < 0.001; **P < 0.01; *P < 0.05 and ^ns^*P* > 0.05). For details, see Materials and methods.

To characterize the AP site cleavage activity of *H*. *pylori* AP endonuclease in more detail, we measured the HpXth protein—mediated cleavage of THF•T in a buffer supplemented with various concentrations of divalent metal chlorides and sulphate, including MgCl_2_, MnCl_2_, CaCl_2_, CoCl_2_, NiCl_2_, CuSO_4_, ZnCl_2_, or FeCl_2_, (Fig 2 and S3 Fig). As shown in Fig 2A, in the presence of 1 or 5 mM Mg^2+^ or Mn^2+^, the purified HpXth protein exerted an efficient AP site cleavage activity by generating fast-migrating 10mer cleavage fragments (lanes 6 and 7 and 10 and 11) which migrated similarly to the 10mer product generated by the control enzyme, human APE1 (lane 1). Nonetheless, higher concentrations of Mg^2+^ or Mn^2+^ (10–20 mM) resulted in a strong decrease in the HpXth-catalyzed AP site cleavage (Fig 2A, lanes 9 and 13, and Fig 2B). The presence of 1 or 5 mM Ca^2+^ caused much less efficient stimulation of HpXth’s AP site cleavage activity as compared to Mg^2+^ or Mn^2+^ (Fig 2A, lanes 14 and 15). Again, high concentrations (10–20 mM) of Ca^2+^ resulted in strong inhibition of the AP endonuclease activity (lanes 16 and 17). It is noteworthy that no cleavage of AP site by HpXth was observed in the absence of any metal cations or in the presence of EDTA (lanes 3–5), indicating the absolute necessity of divalent metal ions for enzymatic catalysis. Taken together, these findings mean that HpXth has a nonlinear dependence on the presence of various concentrations of Mg^2+^ and Mn^2+^: its AP site cleavage activity increased in the presence of 1–5 mM and then decreased in the presence of 10–20 mM MgCl_2_ and MnCl_2_ (Fig 2).

Further characterization of HpXth regarding the divalent-metal requirement showed that at a very low (0.01 mM) concentration, Zn^2+^, Ni^2+^, Fe^2+^, or Cu^2+^ stimulated the AP endonuclease activity, while the same metal cations at 0.1–1.0 mM strongly inhibited AP site cleavage activity of the bacterial enzyme (S3 Fig). To further substantiate inhibitory effects of the above metals, we measured HpXth activity toward THF•T in a buffer supplemented with various concentrations of both metals MgCl_2_ and ZnCl_2_. The results revealed that even low concentrations of ZnCl_2_ (0.1–1 mM) in the reaction mixture already containing 1–5 mM MgCl_2_, strongly inhibited AP site cleavage activity of HpXth (S4 Fig), suggesting that Zn^2+^ and heavier metal cations can efficiently compete with Mg^2+^ for the enzyme’s metal-binding site. Finally, all these results suggest that among metal cations heavier than Mg, only Mn^2+^ and Ca^2+^ can stimulate HpXth AP endonuclease activity in the concentration range 1–5 mM, whereas all other cations, except Co^2+^, stimulate this enzymatic activity only at very low concentrations.

### Effects of pH, temperature, ionic strength, and substitution of a conserved amino acid on *H*. *pylori* AP endonuclease activity

**Fig 3:**
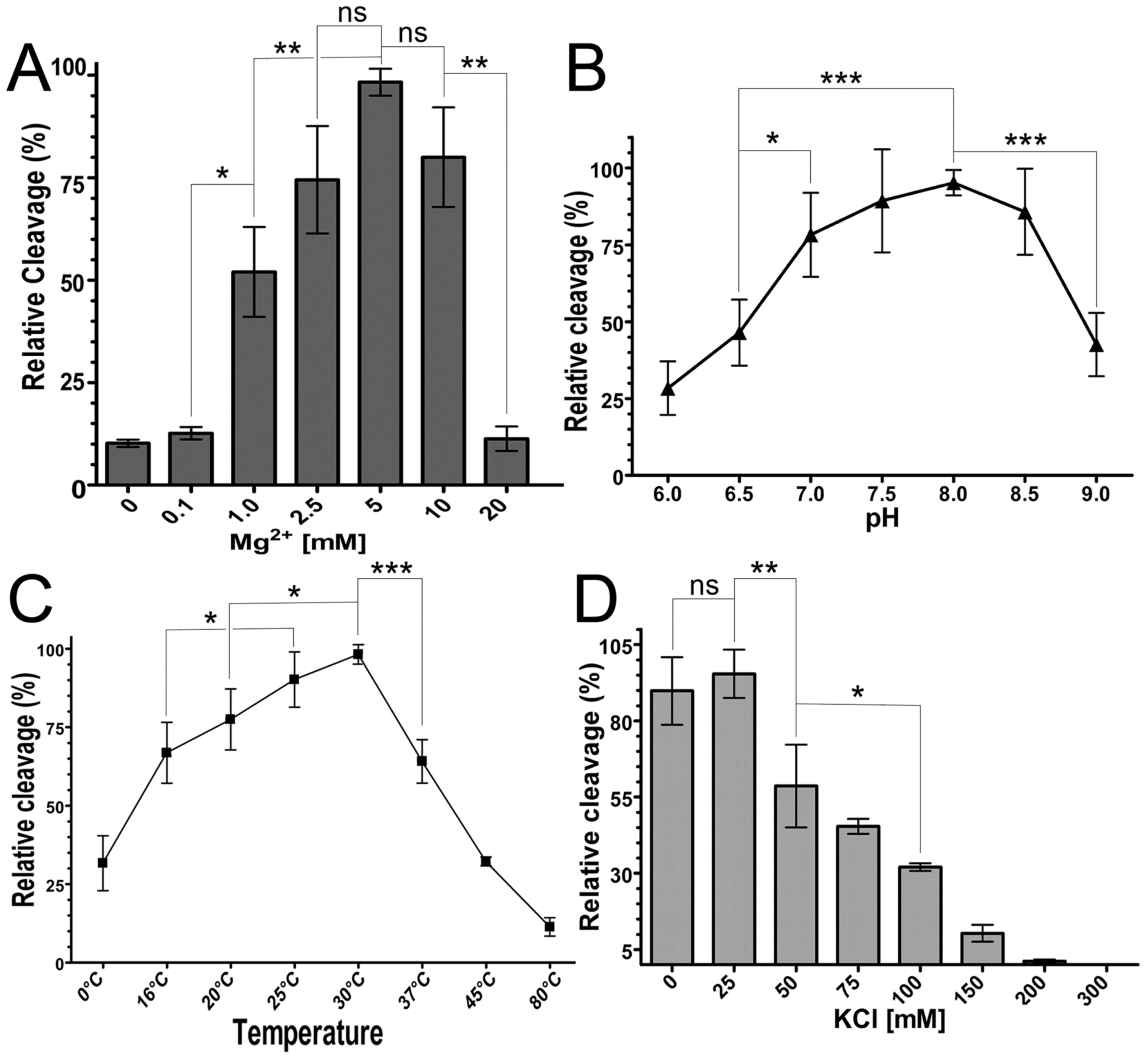
Dependence of HpXth’s AP site cleavage activity on reaction conditions. Briefly, 10 nM 5’-[^32^P]labeled 30mer THF•T duplex was incubated with 0.2 nM HpXth for 5 min in the standard reaction buffer but with varying temperature, pH, concentrations of Mg^2+^ and salt. Products of the reaction were resolved by denaturing PAGE, then visualized by phosphorimaging and quantified in ImageQuant. (**A**) Graphical representation of Mg^2+^ concentration dependence. (**B**) Graphical representation of pH dependence. (**C**) Graphical representation of temperature dependence. (**D**) Graphical representation of ionic-strength dependence. The statistical significance of the differences among the mean values were evaluated using two-tailed Student’s test (***P < 0.001; **P < 0.01; *P < 0.05 and ^ns^*P* > 0.05). For details, see Materials and methods.

To confirm the biochemical properties of the purified HpXth protein, we measured cleavage of the 30mer THF•T duplex at various MgCl_2_ concentrations, ionic-strength levels, pH levels, and temperatures. As shown in Fig 3, MgCl_2_ (panel A), pH (panel B), and temperature (panel C) dependence of the HpXth-catalyzed AP site cleavage was clearly bell-shaped. HpXth manifested the highest AP endonuclease activity at 5 mM MgCl_2_, pH 8.0, and 30 °C. Notably, the *H*. *pylori* AP endonuclease was sensitive to elevated temperature and ionic strength and was inhibited at 37 °C and 50 mM KCl; furthermore 10-fold inhibition was observed at 150 mM KCl (Fig 3D). These results suggest that the requirements for reaction conditions of HpXth are different from those of *E*. *coli* Xth and human APE1 AP endonucleases.

**Fig 4:**
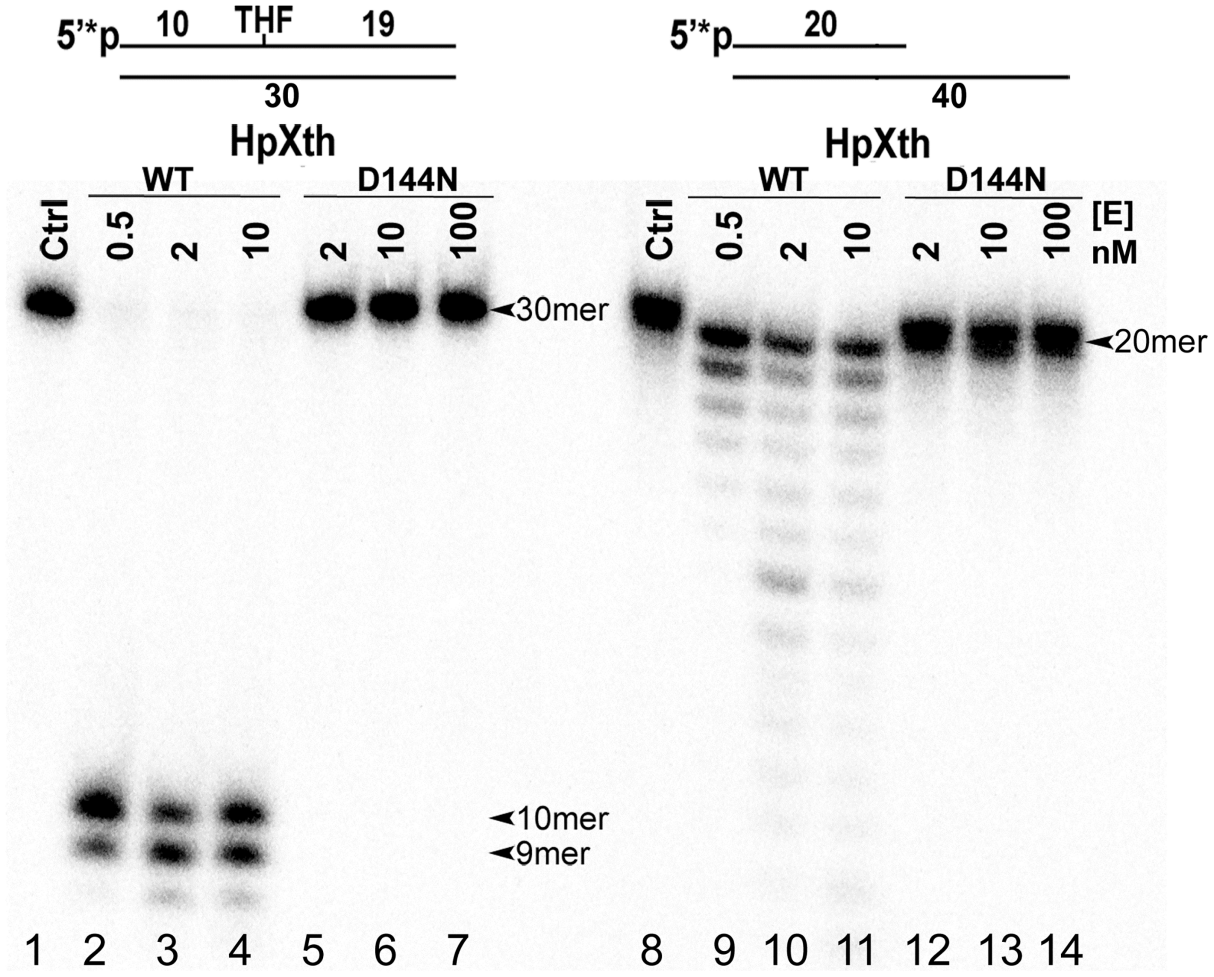
Comparative characterization of AP site cleavage and 3’→5’ exonuclease activities of wild-type HpXth and mutant HpXth-D144N. In brief, 10 nM 5’-[^32^P]labeled THF•T and Exo20•RexT^Rec^ were incubated at 30 °C with increasing amounts of the HpXth-WT and HpXth-D144N proteins for 5 min at 30 °C in the standard reaction buffer. Lanes 1–7, THF•T; lane 1, control, no enzyme; lanes 2–4, 0.5, 2, and 10 nM HpXth-WT, respectively; lanes 5–7, 2, 10, and 100 nM HpXth-D144N, respectively; lanes 8–14, same as in lanes 1–7 but with Exo20•RexT^Rec^. Products of the reaction were analyzed by denaturing PAGE. The arrows denote the position of the 30mer and 20mer substrates and 10mer and 9mer cleavage products. For details, see Materials and methods.

To ensure that the observed AP site cleavage activity is not due to trace contamination via expression of host endonucleases, we constructed mutant of HpXth by site-directed mutagenesis, then purified it by the same scheme as that for the wild-type enzyme. We introduced the mutation D144N into HpXth because in the Xth family enzymes, the residue homologous to D144 of HpXth forms a coordination bond with the catalytic metal ion. In the human APE1 protein, the corresponding mutation D210N reduces the enzymatic activity ~10,000-fold [47]. The purified HpXth-D144N mutant protein was incubated with the 5’-[^32^P]labeled THF•T and recessed Exo20•RexT^Rec^ duplexes to measure AP site cleavage and 3’→5’ exonuclease activities, respectively. Note that the recessed Exo20•RexT^Rec^ duplex oligonucleotide was composed of a long regular 40mer RexT template fragment and a short complementary regular 5’-[^32^P]labeled 20mer Exo20 fragment. As shown in Fig 4, mutant *H*. *pylori* AP endonuclease, even when present in excess, did not show any detectable AP site cleavage activity (lanes 5–7) as compared to wild-type HpXth (lanes 2–4). Of note, HpXth D144N mutant concomitantly lost its nonspecific 3’→5’ exonuclease activity toward the Exo20•RexT^Rec^ duplex, whereas HpXth-WT exerted a DNA-degrading activity under the same reaction conditions (Fig 4, lanes 12–14 versus lanes 9–11). Altogether, these results indicated that D144 of HpXth is essential for DNA repair activities of the *H*. *pylori* protein and that the preparations of recombinant enzymes used in this study are not contaminated with host AP endonucleases.

### Characterization of 3’-repair and NIR activities of pure *H*. *pylori* AP endonuclease

Hydrolytic AP endonucleases, in addition to their classic AP site cleavage activity, catalyze the removal of 3’-blocking sugar-phosphates and 3’-terminal phosphate remnants from DNA strand breaks generated by ROS or DNA glycosylases/AP lyases. To test whether HpXth has the 3’-repair phosphodiesterase activities, we prepared nicked Exo20^THF^•RexT^Nick^ and Exo20^P^•RexT^Nick^ duplexes composed of a long 40mer RexT template fragment and two short complementary DNA fragments: the regular 19mer and 20mer Exo20 fragment, containing a 3’-terminal THF and 3’-phosphate residue, respectively. Of note, Exo20^THF^•RexT^Nick^ and Exo20•RexT^Rec^ duplexes with 5’-[^32^P]labeled Exo20 were used to measure the 3’-repair phosphodiesterase and 3’→5’ exonuclease activities, respectively. The Exo20^P^•RexT^Nick^ duplex with 3’-[^32^P]labeled Exo20 was employed to measure the 3’-phosphatase activity.

**Fig 5:**
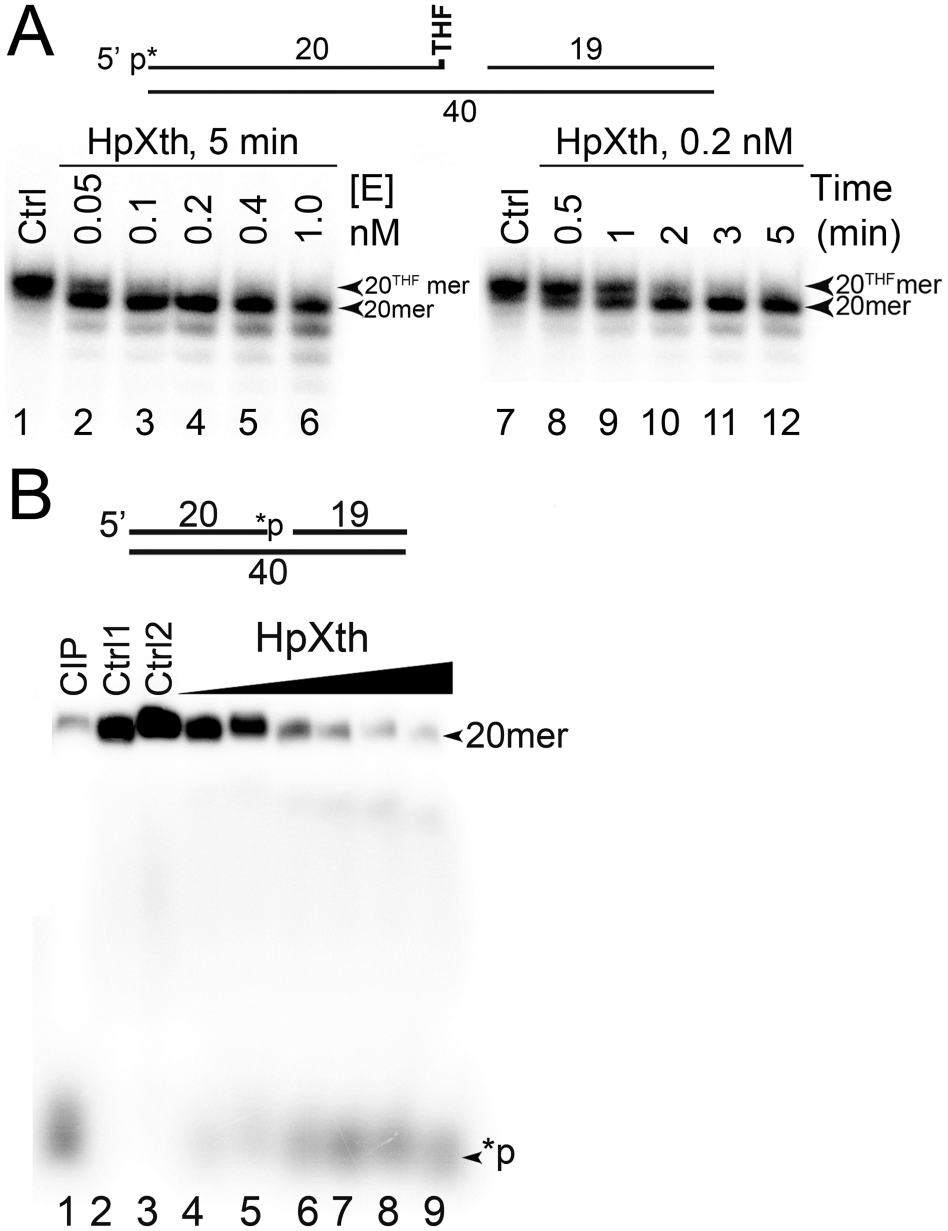
3’-Repair phosphodiesterase and 3’-phosphatase activities of the HpXth protein. (**A**) Enzyme concentration and time-dependent cleavage of the 5’-[^32^P]labeled Exo20^THF^•RexT^Nick^ oligonucleotide duplex by HpXth. In brief, 10 nM Exo20^THF^•RexT^Nick^ was incubated with increasing amounts of HpXth in the standard reaction buffer at 30 °C for various periods. Lanes 1–6, time-dependent cleavage; lanes 7–12, enzyme concentration—dependent cleavage. The arrows denote the position of substrate “20^THF^mer” and cleavage product “20mer.” (**B**) Enzyme concentration—dependent cleavage of the 3’-[^32^P]labeled Exo20^P^•RexT^Nick^ oligonucleotide duplex by HpXth. In brief, 10 nM Exo20^P^•RexT^Nick^ was incubated with various concentrations of HpXth in the standard reaction buffer for 5 min at 30 °C. Lane 1, Exo20^P^•RexT^Nick^ incubated with 1 U of calf Intestinal alkaline phosphatase, CIP; lanes 2–3, control, no enzyme; lanes 4–9, as in lane 2 but 0.5, 1, 2, 5, 10 and 20 nM HpXth. The arrows denote the position of the 20mer substrate (20mer) and 3’-terminal phosphate product (*p). Products of the reaction were analyzed by denaturing PAGE. For details see Materials and methods.

As shown in Fig 5A, HpXth cleaves the nicked 5’-[^32^P]labeled Exo20^THF^•RexT^Nick^ duplex generating a 20mer DNA fragment that migrates faster than the 20mer Exo20^THF^ substrate, indicating that the enzyme removes the 3’-terminal THF residue, thus leaving a Exo20 fragment with 3’-OH (lanes 2–6 and 10–12). Even at a low (0.05 nM) enzyme concentration, HpXth removed the 3’-THF residue in more than 90% of Exo20^THF^•RexT^Nick^ duplex molecules in 5 min (lane 2), whereas 0.2 nM HpXth repaired most of the DNA substrate after only 2 min of incubation (lane 10), suggesting that *H*. *pylori* AP endonuclease has a highly efficient 3’-phosphodiesterase activity. When acting on the 3’-[^32^P]labeled Exo20^P^•RexT^Nick^ duplex, HpXth generates a free radioactive phosphate [^32^P] residue, which migrates at the bottom of the gel (Fig 5B, lanes 6–9), similar to the product generated by calf intestinal alkaline phosphatase (CIP; lane 1), indicating that the bacterial AP endonuclease removes the 3’-terminal [^32^P] residue leaving an unlabeled Exo20 fragment. Of note, a higher protein concentration of HpXth (5 nM) was required to remove more than 50% of phosphate residues from the Exo20^P^•RexT^Nick^ duplex (lane 7) as compared to the Exo20^THF^ substrate.

**Fig 6:**
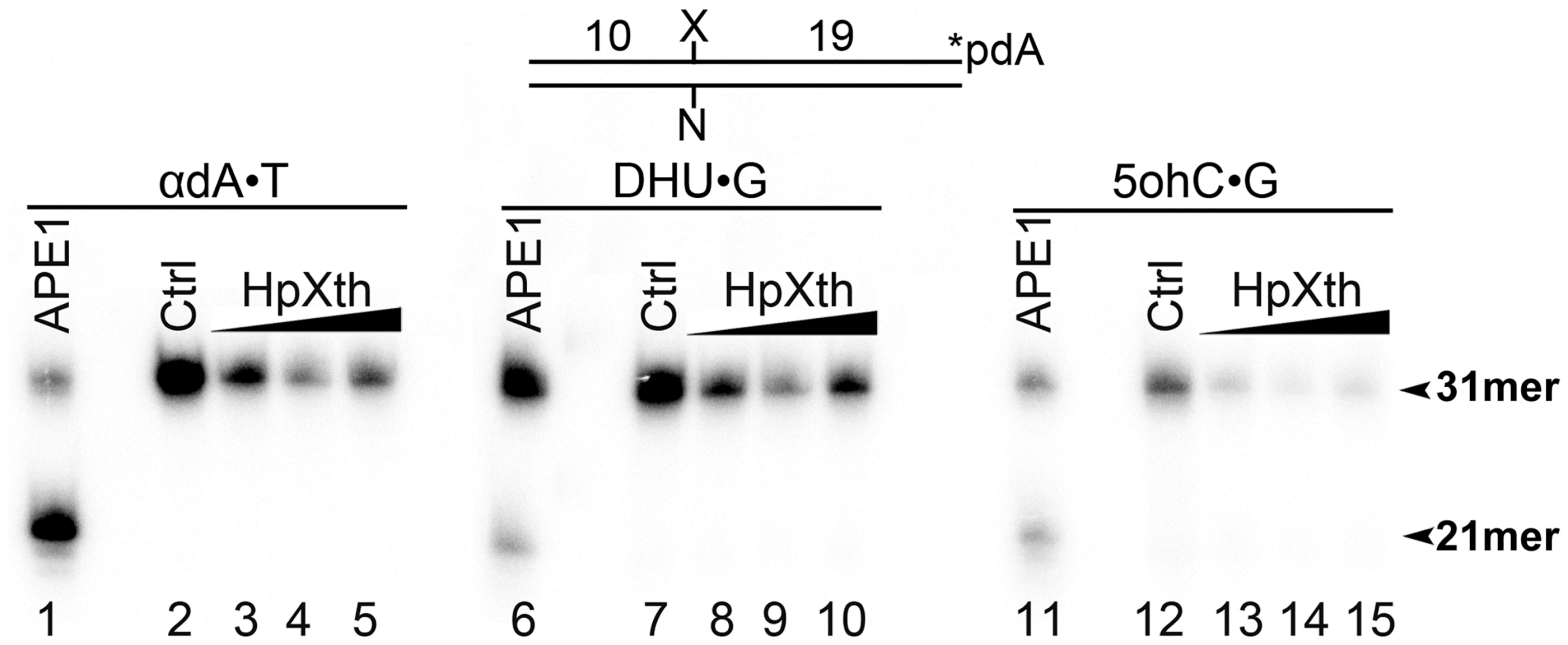
An assay of the nucleotide incision activity of *H*. *pylori* HpXth toward a 30mer oligonucleotide duplex containing a modified base. In brief, 10 nM 3’-[α-^32^P]-dAMP-labeled 31mer oligonucleotide duplexes containing various DNA base lesions were incubated with increasing amounts of the purified HpXth protein in the buffer NIR for 10 min at 30 °C. Products of the reaction were analyzed by denaturing PAGE. Lanes 1–5, αdA•T; lane 1, 2 nM APE1; lane 2, control no enzyme; lanes 3–5, 5, 20, and 100 nM HpXth, respectively; lanes 6–10, same as in lanes 1–5 but DHU•G; lanes 11–15, same as in lanes 1–5 but 5ohC•G. The arrows denote the position of the 31mer substrate and 21mer cleavage product. The statistical significance of the differences among the mean values were evaluated using two-tailed Student’s test (***P < 0.001; **P < 0.01; *P < 0.05 and ^ns^*P* > 0.05). For details see Materials and methods.

We further examined the DNA substrate specificity of *H*. *pylori* enzyme by means of 3’-[^32^P]labeled 30mer duplex oligonucleotides containing a damaged nucleotide: αdA, DHU, or 5ohC. In control experiments, APE1 cleaved αdA•T, DHU•G, and 5ohC•G duplexes (Fig 6, lanes 1, 6, and 11). No cleavage 5’ to the lesion site of DNA duplexes was observed in the presence of HpXth (lanes 3–5, 8–10, and 13–15), suggesting that *H*. *pylori* AP endonuclease does not possess a NIR activity under the experimental conditions tested.

### Steady-state kinetic parameters of the DNA repair reactions catalyzed by *H*. *pylori* AP endonuclease

To further substantiate the DNA substrate specificity of the purified HpXth protein, we determined steady-state kinetic parameters of the repair activities and calculated *K*_M_, *k*_cat_, and *k*_cat_/*K*_M_ values for the cleavage of various DNA substrates. The 30mer THF•T and 40mer Exo20^THF^•RexT^Nick^, Exo20^P^•RexT^Nick^, and Exo20•RexT^Rec^ duplex oligonucleotides with an AP site residue and a 3’-THF, 3’-P, and 3’-OH group were applied to measure AP endonuclease, 3’-phosphodiesterase, 3’-phosphatase, and 3’→5’ exonuclease activities, respectively. For comparison of the kinetic parameters, *E*. *coli* Xth and human APE1 data are presented. As shown in Table 1, steady-state kinetic parameters of HpXth-catalyzed repair reactions revealed that the *H*. *pylori* enzyme has a strong substrate preference for AP sites in duplex DNA. The *k*_cat_/*K*_M_ value of HpXth for AP site cleavage is 28- and 9-fold higher than that of its 3’-phosphodiesterase and 3’→5’ exonuclease activities. It is noteworthy that the *k*_cat_/*K*_M_ values for AP site cleavage and 3’-phosphodiesterase activities of HpXth toward the THF•T and Exo20^THF^•RexT^Nick^ duplexes were quite similar to those of *E*. *coli* Xth, but nearly 10-fold lower than those of human AP endonuclease 1 (APE1). Similar to human APE1, HpXth has a weak 3’-phosphatase activity with ~220-fold lower *k*_cat_/*K*_M_ as compared to that of its AP endonuclease function. Overall, these kinetic parameters suggested that *H*. *pylori* AP endonuclease is similar to *E*. *coli* Xth and can be considered a major cellular enzyme that processes AP sites and DNA strand breaks containing 3’-blocking groups *in vivo*.

**Table 1:** A comparison of kinetic parameters of AP endonucleases from *E*. *coli*, *H*. *pylori*, and humans.

	Protein										
	HpXth			*E*. *coli* Xth^b^				Human APE1^c^			
DNA substrate^a^	*K*_M_, nM	*k*_cat_, min^–1^	*k*_cat_/*K*_M_, min^–1^·μM^–1^	*K*_M_, nM	*k*_cat_, min^–1^	*k*_cat_/*K*_M_, min^–1^·μM^–1^	*k*_cat_/*K*_M_ ratio, HpXth/EcXth	*K*_M_, nM	*k*_cat_, min^–1^	*k*_cat_/*K*_M_, min^–1^·μM^–1^	*k*_cat_/*K*_M_ ratio, HpXth/APE1
THF•T	15.0 ± 2.2	18.5	1240	31	40	1300	1	7.7	97	12,700	0.10
Exo20^THF^•RexT^Nick^	202 ±100	8.8	43.6	731	32.2	44	1	6.0	20	3300	0.13
Exo20^P^•RexT^Nick^	40 ±14	0.22	5.4	-	-	-	-	20	3.6	180	0.03
Exo20•RexT^Rec^	6800 ± 1000	920 ± 140	135	-	-	-	-	2.4	0.86	360	0.38

^a^Each substrate was used to measure a specific DNA repair function under the optimal reaction conditions: the THF•T duplex for the AP endonuclease activity; the Exo20^THF^•RexT^Nick^ nicked DNA duplex for the 3’-repair phosphodiesterase activity; the Exo20^P^•RexT^Nick^ nicked DNA duplex for the 3’-phosphatase activity; and the Exo20•RexT^Rec^ recessed DNA duplex for the 3’→5’ exonuclease activity (see Materials and methods).

^b^Kinetic parameters of the *E*. *coli* Xth-catalyzed AP site cleavage and 3’-phosphodiesterase reaction were taken from other studies [26, 59].

^c^Kinetic parameters of human APE1-catalyzed DNA repair reactions were taken from ref. [37].

### Homology modeling and phylogenetic analysis of HpXth

**Fig 7:**
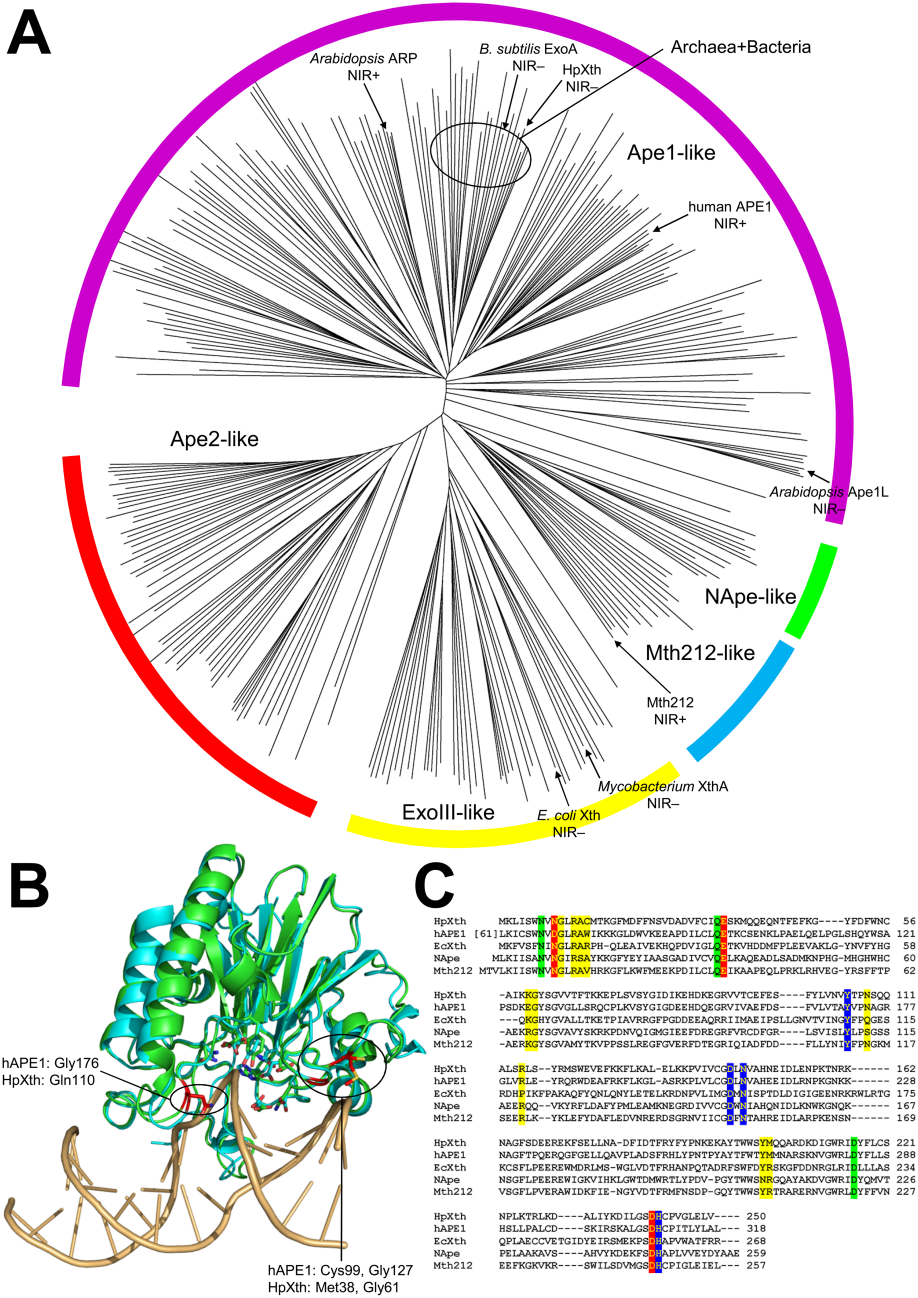
Phylogenetic analysis and structural models of *H*. *pylori* AP endonuclease. (**A**) An unrooted phylogenetic tree of the EEP superfamily AP endonucleases (230 sequences, one species per taxonomic class). Different families are indicated by colored arcs: yellow, ExoIII-like; green, Nape-like; blue, Mth212-like; magenta, Ape1-like; and red, Ape2-like. The clade combining HpXth, *B*. *subtilis* ExoA, and the related bacterial and archaeal sequences is labeled “Archaea+Bacteria.” The known NIR-proficient and NIR-deficient enzymes are indicated. (**B**) Overlay of the structure of human APE1 (1DEW, cyan) and the HpXth model (green). All active-site residues are shown but left unlabeled for clarity. The DNA groove—contacting residues possibly involved in the NIR function are indicated by red carbon atoms. (**C**) Alignment of HpXth and four core members of AP endonuclease families (human APE1 catalytic domain, *E*. *coli* Xth, *N*. *meningitidis* NApe, and *M*. *thermautotrophicus* Mth212). Metal-binding sites A and B are red and blue, respectively, non—metal-binding active site residues are green; other DNA-binding residues are yellow.

AP endonucleases belonging to the Endonuclease—Exonuclease—Phosphatase (EEP) superfamily are classified into five families based on their sequence homology: ExoIII-like (with *E*. *coli* Xth being the prototypical member), NApe-like (similar to *Neisseria* NApe endonuclease), Mth212-like (similar to *Methanothermobacter* Mth212 endonuclease), Ape1-like and Ape2-like (similar to human APE1 and APE2, respectively). Unlike its *E*. *coli* homolog, HpXth is classified as an Ape1-like endonuclease by the NCBI Conserved Domain Database [48]. We re-examined the phylogenetic relations among the EEP superfamily AP endonucleases using a set of 284 sequences evenly spread over all domains of life (Fig 7A and S1 File). HpXth, together with several other bacterial and archaeal sequences, formed a separate clade among the Ape1-like family members (labeled as Archaea+Bacteria in Fig 7A). *Bacillus subtilis* ExoA protein, an efficient AP endonuclease with a low NIR activity [49], also fell into this clade. A closer look at Epsilonproteobacteria, the class to which *H*. *pylori* belongs, revealed that basal Epsilonproteobacteria possess AP endonucleases from ExoIII-like, Mth212-like, and Ape1-like families, but some of those were lost during the evolution: in Helicobacteraceae, only Ape1-like sequences remain, while its sister group, Campylobacteraceae, retained only Mth212-like AP endonucleases.

Multiple threading revealed the best templates for HpXth to be *B*. *subtilis* ExoA [PDB entry 5CFE, [49]], *Archaeoglobus fulgidus* AP endonuclease [2VOA, [50]], *N*. *meningitidis* NApe [2JC5, [51]], *M*. *thermautotrophicus* Mth212 [3FZI, [52]], and 3W2X, unpublished), and human APE1 [2O3H, [53]]. The calculations converged to a single model (S2 File) with the excellent confidence score 1.96. The model showed the best fit to the template proteins plus *Leishmania major* AP endonuclease LMAP [2J63, [54]], *Methanosarcina mazei* exonuclease III homolog Mm3148 (5J8N, unpublished), *N*. *meningitidis* AP endonuclease NExo [2JC4, [51]], and *Sulfolobus islandicus* exonuclease III (5EWT, unpublished). The superposition of the model and human APE1/DNA structure [1DEW, a complex with THF-containing DNA [55]] showed low r.m.s.d. (0.848 over the heavy atoms), with all catalytically important residues in the active site of APE1 conserved in HpXth and occupying similar positions (Fig 7B and S5 Fig). One exception is Asn9 of HpXth, which corresponds to Asp70 in the human protein and forms part of a catalytic Mg^2+^-binding site (Fig 7C). However, Ape1-like enzymes vary at this position, containing Asp, Asn, or small amino acids (Gly/Ala). Despite influencing the metal preference and/or the balance of endonuclease, exonuclease, and 3’-phosphodiesterase activities [33], both Asn and Asp are likely suitable for the biologically relevant function of EEP superfamily AP endonucleases.

### Drug sensitivity of an *E*. *coli* DNA repair-deficient strain expressing the *H*. *pylori* AP endonuclease

**Fig 8:**
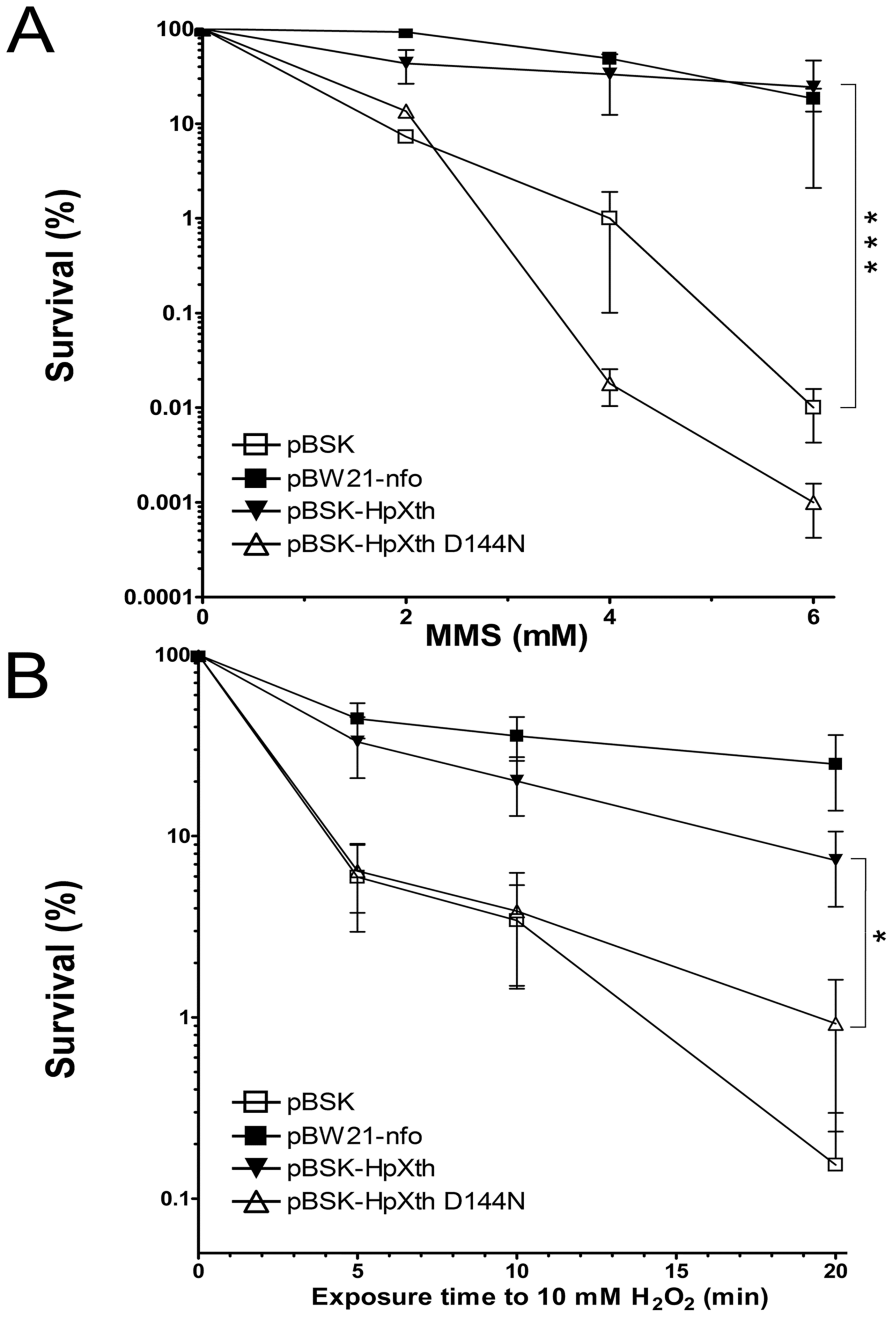
Differential drug sensitivity of AP endonuclease—deficient *E*. *coli* strain BH110 (DE3) carrying a plasmid coding for the *H*. *pylori* AP endonuclease. The strains are represented as follows: the BH110 (DE3) strain carrying control empty vector pBluescript II SK+ (pBSK) (□), strain BH110 (DE3) carrying pBW21-Nfo (■), or pBSK-HpXth (▼), or pBSK-HpXth-D144N (△). Each survival curve represents at least three independent experiments. (**A**) The survival of the MMS-treated *E*. *coli* AP endonuclease—deficient strains. (**B**) The survival of the *E*. *coli* AP endonuclease—deficient strains under oxidative stress. The statistical significance of the differences among the mean values were evaluated using two-tailed Student’s test (***P < 0.001; **P < 0.01; *P < 0.05 and ^ns^*P* > 0.05). For details, see Materials and methods.

To examine the biological roles of HpXth’s activities, we employed the phenotype rescue assay of *E*. *coli* mutant. We measured the sensitivity of AP endonuclease-deficient *E*. *coli xthA nfo* BH110 (DE3) strain harboring plasmid pBW21 coding for the *E*. *coli* Nfo and plasmid pBluescript SK(+) containing inserts coding for wild-type or mutant pathogen’s AP endonuclease HpXth or HpXth-D144N, respectively, to exposure to MMS and H_2_O_2_. Western blotting using rabbit anti-HpXth polyclonal antibodies confirms the presence of the HpXth proteins in *E*. *coli* BH110 (DE3) cells harboring the plasmids that encode the AP endonuclease from *H*. *pylori* (S6 Fig). MMS methylates purines in cellular DNA, subsequently methylated purines are excised by DNA glycosylases in the BER pathway leading to the appearance of AP sites in DNA [56]. Exposure to H_2_O_2_ oxidizes DNA bases and also generates single-strand DNA breaks with blocked 3’ termini [57]. The AP endonuclease-deficient *E*. *coli* BH110 (DE3) strain lacking both Xth and Nfo is very sensitive to both agents [58]. As expected, the plasmid that encodes for *E*.*coli* Nfo rescues BH110 (DE3) cells after exposure to MMS and H_2_O_2_ (Fig 8). Consistent with our biochemical results, the pBSK-HpXth plasmid encoding HpXth restored the resistance to MMS to the level close to that of plasmid-harboring *E*. *coli* Nfo, showing a 10- to 10^4^-fold increase in the cell survival at different concentration of MMS as compared to the control empty plasmid (Fig 8A).

Plasmid pBSK-HpXth partially restored the resistance to H_2_O_2_ showing a 5- to 100-fold increase in the cell survival at different periods of exposure to H_2_O_2_ as compared to the control empty plasmid (Fig 8B). As expected, the plasmid harboring mutant HpXth-D144N did not confer resistance to MMS and H_2_O_2_, as was the case for the empty control plasmid, suggesting that this enzyme’s catalytic activities are essential for their biological functions (Fig 8). Remarkably, the *E*. *coli* BH110 (DE3) cells expressing HpXth-D144N were more sensitive to MMS as compared to cells carrying the empty control vector, suggesting that the mutant *H*. *pylori* protein may inhibit repair of AP sites by other back-up DNA repair pathways. Taken together, these results suggested that HpXth can efficiently repair AP sites and only to some extent 3’-blocking groups in *E*. *coli*.

## Discussion

Unlike many bacteria, including *E*. *coli*, which possess two structurally unrelated AP endonucleases, the *Helicobacter* lineage has retained only a homolog of exonuclease III. Previously, it has been demonstrated that the mutants of *H*. *pylori* lacking HpXth (*nucT HP1526* and *xthA*::*cat*) have undetectable Mg^2+^-dependent AP site cleavage activity and show a 4-fold increase in the spontaneous mutation frequency over the wild-type strain, suggesting that HpXth is a functional homolog of *E*. *coli* Xth [31–32]. Nevertheless, HpXth has not been biochemically characterized to date. In this study, we have cloned cDNA encoding the HpXth, overexpressed and purified it from *E*. *coli*, and characterized the DNA substrate specificity of the enzyme. Our results suggest that HpXth protein possesses AP site cleavage, 3’-repair phosphodiesterase, 3’-phosphatase, and 3’-exonuclease functions. Furthermore, HpXth, just as *E*. *coli* Xth, but in contrast to their human homolog APE1, has no damage-specific nucleotide incision activity.

### Enzymatic activities of HpXth

In line with the well-characterized *E*. *coli* Xth, our data indicate that HpXth is a divalent metal ion—dependent enzyme requiring Mg^2+^ or Mn^2+^ for the full activity (Figs 2 and 3). Of note, HpXth was considerably inhibited in the presence of 0.1–1.0 mM ZnCl_2_ (S3 and S4 Figs), suggesting that Zn^2+^ ions could thwart AP site repair in *H*. *pylori*. The detailed characterization of the reaction conditions showed that HpXth-catalyzed AP site cleavage is optimal at a high Mg^2+^ concentration (5 mM), neutral pH, low ionic strength, and 30 °C (Fig 3). These reaction conditions with low salt and relatively high Mg^2+^ more closely resemble the optimum of *E*. *coli* Xth [59] than that of human APE1 [13]. The Mg^2+^ concentration, ionic strength, pH, and even temperature dependence curves of HpXth-catalyzed AP endonuclease activity are all bell-shaped. These observations may imply that the catalytically active protein conformation is sensitive to changes in the reaction conditions; perhaps *in vivo*, the HpXth-catalyzed reactions are tightly regulated.

The preparation of recombinant HpXth displayed nonspecific 3’→5’ exonuclease activity degrading nicked DNA after the AP site cleavage and undamaged recessed DNA duplexes (Figs 1 and 4). To confirm that the exonuclease activity is an intrinsic property of this AP endonuclease, we constructed and characterized the HpXth containing a D144N mutation that should disrupt the metal-binding shell. The mutant completely lost both the ability to cleave AP sites and the 3’→5’ exonuclease activity (Fig 4). Hence, the the 3’→5’ exonuclease function is intrinsically present in HpXth and may play an important physiological role.

To get an idea about comparative importance of the observed enzymatic activities of HpXth, we have measured the steady-state kinetic parameters of its AP site cleavage, 3’-repair phosphodiesterase, 3’-phosphatase, and 3’→5’ exonuclease activities. The analysis of kinetic constants revealed that HpXth possesses efficient AP endonuclease and 3’-repair phosphodiesterase activities with *k*_cat_ and *k*_cat_/*K*_M_ values very similar to those of *E*. *coli* Xth (Table 1). For both bacterial enzymes, these constants were 2.5–10-fold lower in comparison with the human counterpart, APE1. Nevertheless, the kinetic parameters suggest that HpXth can efficiently protect *H*. *pylori* from endogenous and induced AP sites and strand breaks in genomic DNA.

### Phylogenetic distribution and structural features of EEP activities

In addition to the AP site and 3′-end processing activities, certain EEP superfamily enzymes possess the NIR activity that nicks DNA at some sites containing damaged bases. Notably, the NIR activity is distributed unevenly in the phylogeny: it has been reported for human APE1 [13], *Arabidopsis* ARP [33], and *Methanothermobacter* Mth212 [60], but is lacking or negligible in ExoIII-like enzymes [13, 26], bacterial/archaeal Ape1-like enzymes [[49] and this work] and plant Ape1L [35]. Ape2-like enzymes, in view of their low AP endonuclease activity and the predominant 3’→5’-exonuclease activity [61], are also likely to be free of the NIR activity. The identification of NIR-deficient HpXth, grouped with low-NIR *B*. *subtilis* ExoA, allowed us to revisit the requirements for efficient NIR among the EEP endonucleases.

The phylogenetic analysis of the EEP superfamily AP endonucleases suggests that either the NIR function was present in an ancestral EEP endonuclease and then was lost in certain lineages (such as ExoIII-like, bacterial/archaeal Ape1-like, and plant Ape1L), or was independently acquired by APE1, ARP, and Mth212-like homologs. Because the loss of a function is more probable than independent emergence of one in several lineages, we examined the conservation of residues in different EEP groups to find which positions differentiate NIR-proficient enzymes from NIR-deficient ones, assuming the Ape1-like and Mth212-like enzymes to be NIR-proficient unless clustering with *B*. *subtilis* ExoA/HpXth or Ape1L. We identified three positions that could be related to the NIR proficiency; of note, all of them are involved in forming contacts with DNA away from the active site (Fig 7B, carbons colored red). The first two corresponded to Cys99 and Gly127 in human APE1. Gly127 was replaced by Lys/Arg in all Ape1L enzymes, whereas the homologs of Cys99 were found to have small side chains in most NIR-proficient enzymes and aliphatic bulky ones in NIR-deficient enzymes (e.g., Met in HpXth or Val in *E*. *coli* Xth). In human APE1, Cys99 and Gly127 form a part of the structure that is inserted into the DNA minor groove 5’ to the damaged nucleotide [55]. At the same side but closer to the lesion, Gly176 in APE1 corresponds to small-side chain residues in the major groove in most NIR-proficient enzymes, whereas NIR-deficient ones possess a charged or polar group at the equivalent position (Glu in *E*. *coli* Xth, Gln in HpXth). These differences could lead to different modes of DNA kinking upon binding to the enzyme, and these conditions might be crucial for the correct entry of base-containing nucleotides into the active site of AP endonucleases. A comparison of Ape1-like NIR-proficient enzymes with NIR-deficient ones reveals that the former group has Phe/Tyr in the active site pressing against the everted abasic nucleotide (Phe266 in human APE1), whereas all NIR-deficient enzymes have a bulkier Trp side chain at this position, possibly interfering with the correct eversion of the damaged-base—containing nucleotide during NIR.

Many bacterial enzymes, including AP endonucleases, are different from their eukaryotic counterparts by having no or little regulatory additions to the catalytic domains. Comparing human APE1 with bacterial Xth, it is clear that the human protein carries an additional N-terminal tail, where all regulatory Lys residues are located. Sequence alignment of human APE1 and *E*. *coli* and *H*. *pylori* Xth clearly indicate that there is no counterparts of regulatory Lys [62] and redox-active Cys residues [63] in the bacterial enzymes (S7 Fig). This observation most likely reflects the differences in the intracellular distribution of the human and bacterial enzymes and in their involvement in the transcription regulation.

### Biological role of a single *H*. *pylori* AP endonuclease

Efficient AP endonuclease and 3’-end repair functions of HpXth suggest that *H*. *pylori* AP endonuclease plays an important role in the repair of AP sites and single-strand DNA breaks with blocked 3’ termini generated by alkylating agents, ROS, or DNA glycosylases. Consistent with DNA substrate specificities characterized *in vitro*, the expression of HpXth in *E*. *coli* AP endonuclease—deficient BH110(DE3) strain conferred resistance to MMS comparable to that provided by a plasmid encoding *E*. *coli* Nfo (Fig 8A). In addition, HpXth imparted resistance to H_2_O_2_ but to a lesser degree than *E*. *coli* Nfo did (Fig 8B); this finding may be consistent with less efficient 3’-end repair functions as compared to the AP site cleavage activity (Table 1). Also, the absence of the NIR function in HpXth found here is suggestive of possibly increased sensitivity of *H*. *pylori* to oxidizing agents such as bleomycin and organic peroxides, that induce specific lesions that are subject to NIR [58, 64]. In conclusion, our findings together with earlier reports unequivocally identify HpXth as the principal AP endonuclease and 3’-repair phosphodiesterase in *H*. *pylori*, protecting this important human pathogen from the genotoxic effects of endogenous and induced AP sites and DNA strand breaks.

## Supporting information

S1 File Full-length sequences of 284 Endonuclease—Exonuclease—Phosphatase superfamily AP endonucleases, whose highlighted domains were subjected to the phylogenetic analysis. Domains and their families were assigned according to the Conserved Domain Database [48].
(DOCX)

S2 File A model of *Helicobacter pylori* AP endonuclease. (PDB)

S1 Fig Schematic presentation of various DNA substrates used in this study. (**A**) Duplex oligonucleotides used to measure 3’-repair phosphodiesterase and 3’-5’ exonuclease activities. (**B**) Chemical structures of abasic sites and alpha-anomeric 2’deoxyadenosine.
(TIF)

S2 Fig SDS-PAGE analysis of the purified *H*. *pylori* AP endonuclease. Lane 1, Protein Ladder (Thermo Scientific, cat. # 26616); lane 2, 1 μg HpXth; lane 3, 1 μg HpXth-D144N. For details, see Materials and methods.
(TIF)

S3 Fig Graphical representation of divalent-metal ion dependence of HpXth-catalyzed AP site cleavage. Briefly, 10 nM 5’-[^32^P]-labeled 30mer THF•T duplex was incubated with 0.2 nM HpXth for 5 min at 37 °C in the buffer containing various concentrations of metal cations. Products of the reaction were resolved by denaturing PAGE, then visualized by phosphorimaging and quantified in ImageQuant. For details, see Materials and methods.
(TIF)

S4 Fig The effect of Zn^2+^ ions on HpXth-catalyzed AP site cleavage. In brief, 10 nM 5’-[^32^P]-labeled 30mer THF•T duplex was incubated with 0.2 nM HpXth for 5 min at 37 °C in the standard reaction buffer but with varying concentrations of MgCl_2_ and ZnCl_2_, unless otherwise stated. Lane 1, THF•T and 0.1 nM APE1 in the buffer BER; lane 2, control THF•T, no enzyme; lanes 3–4, as in lane 1 but supplemented with 0.1 and 1 mM ZnCl_2_, respectively; lane 5, THF•T and 0.2 nM HpXth in the buffer without metal cations; lane 6, as in lane 5 but 1 mM MgCl_2_; lane 7, as in lane 6 but 0.1 mM ZnCl_2_; lane 8, as in lane 6 but 1 mM ZnCl_2_; lane 9, as in lane 5 but 5 mM MgCl_2_; lane 10, as in lane 9 but 0.1 mM ZnCl_2_; lane 10, as in lane 9 but 1 mM ZnCl_2_. The arrows denote the position of the 30mer substrate and a 10mer cleavage product. For details, see Materials and methods.
(TIF)

S5 Fig Overlay of the active site of HpXth model with known structures of AP endonucleases. HpXth model superimposed over human APE1 structure (1BIX). Sm^3+^ ion replacing catalytic Mg^2+^ in the crystal structure is shown as a magenta ball. Amino acids labels show numbering in HpXth first, followed by numbering in the corresponding human or *E*. *coli* protein. Cartoon traces and carbon atoms of HpXth model are colored green, those of human and *E*. *coli* structures are cyan, oxygen and nitrogen atoms are red and blue, respectively.
(TIF)

S6 Fig Analysis of the expression of HpXth proteins in *E*. *coli* BH110 (DE3) cells. Four μg of cell-free extracts from the BH110 (DE3) strains carrying different plasmids were separated using 12% SDS-PAGE and transferred to the PVDF membrane and then analyzed by Western blotting. (**A**) Western blot analysis of the PVDF membrane using rabbit anti-HpXth polyclonal antiserum. Lane 1, protein molecular weight markers; lane 2, control plasmid-free *E*. *coli* BH110 (DE3) strain; lane 3, empty vector pBluescript II SK+ (pBSK); lane 4, pBSK-HpXth; lane 5, pBSK-HpXth-D144N; lane 6, 50 ng of the purified His-tagged HpXth; lane 7, as 6 but 100 ng. (**B**) Ponceau S-staining of the PVDF membrane. Lanes 1–7, same as in panel A. For details, see Materials and methods.
(TIF)

S7 Fig Amino acid sequence alignment of human APE1 and *E*. *coli* and *H*. *pylori* Xth, with the regulatory Lys residues and the redox-active Cys residue of the human protein underlined, and the catalytic domain highlighted. (TIF)

## Abbreviations

3’OH: 3’-terminal hydroxyl
3’P: 3’-terminal phosphate
3’PA: 3’-terminal phospho α,β-unsaturated aldehyde
3’-THF: 3’-terminal THF residue
αdA: alpha-anomeric 2’-deoxyadenosine
αdN: alpha-anomeric 2’-deoxynucleoside
AP: apurinic/apyrimidinic
APE1: major human AP endonuclease 1
BER: base excision repair
DHU: 5,6-dihydrouracil
EEP: Endonuclease—Exonuclease—Phosphatase
HpXth: *Helicobacter pylori* homolog of *E*. *coli* exonuclease III
IPTG: isopropyl β-D-1-thiogalactopyranoside
MMS: methylmethanesulfonate
NIR: nucleotide incision repair
nt: nucleotide
ROS: reactive oxygen species
THF: 3-hydroxy-2-hydroxymethyltetrahydrofuran or tetrahydrofuran
Xth: *Escherichia coli* exonuclease III

## References

[1] Bjelland S, Seeberg E. Mutagenicity, toxicity and repair of DNA base damage induced by oxidation. Mutat Res. 2003;531(1–2):37–80. . 14637246 10.1016/j.mrfmmm.2003.07.002

[2] Cadet J, Douki T, Gasparutto D, Ravanat JL. Oxidative damage to DNA: formation, measurement and biochemical features. Mutat Res. 2003;531(1–2):5–23. . 14637244 10.1016/j.mrfmmm.2003.09.001

[3] Lindahl T. Instability and decay of the primary structure of DNA. Nature. 1993;362(6422):709–15. 10.1038/362709a0 . 8469282

[4] Barnes DE, Lindahl T. Repair and genetic consequences of endogenous DNA base damage in mammalian cells. Annu Rev Genet. 2004;38:445–76. 10.1146/annurev.genet.38.072902.092448 . 15568983

[5] Ischenko AA, Saparbaev MK. Alternative nucleotide incision repair pathway for oxidative DNA damage. Nature. 2002;415(6868):183–7. 10.1038/415183a . 11805838

[6] Yasui A. Alternative excision repair pathways. Cold Spring Harb Perspect Biol. 2013;5(6):1–8. 10.1101/cshperspect.a012617 . 23645854 PMC3660826

[7] Zharkov DO. Base excision DNA repair. Cell Mol Life Sci. 2008;65(10):1544–65. 10.1007/s00018-008-7543-2 . 18259689 PMC11131669

[8] Wallace SS. Base excision repair: A critical player in many games. DNA Repair (Amst). 2014 10.1016/j.dnarep.2014.03.030 . 24780558 PMC4100245

[9] Svilar D, Goellner EM, Almeida KH, Sobol RW. Base excision repair and lesion-dependent subpathways for repair of oxidative DNA damage. Antioxid Redox Signal. 2011;14(12):2491–507. 10.1089/ars.2010.3466 . 20649466 PMC3096496

[10] Wilson DM 3rd, Barsky D. The major human abasic endonuclease: formation, consequences and repair of abasic lesions in DNA. Mutat Res. 2001;485(4):283–307. . 11585362 10.1016/s0921-8777(01)00063-5

[11] Ide H, Tedzuka K, Shimzu H, Kimura Y, Purmal AA, Wallace SS, et al Alpha-deoxyadenosine, a major anoxic radiolysis product of adenine in DNA, is a substrate for Escherichia coli endonuclease IV. Biochemistry. 1994;33(25):7842–7. . 7516707 10.1021/bi00191a011

[12] Ishchenko AA, Ide H, Ramotar D, Nevinsky G, Saparbaev M. Alpha-anomeric deoxynucleotides, anoxic products of ionizing radiation, are substrates for the endonuclease IV-type AP endonucleases. Biochemistry. 2004;43(48):15210–6. 10.1021/bi049214+ . 15568813

[13] Gros L, Ishchenko AA, Ide H, Elder RH, Saparbaev MK. The major human AP endonuclease (Ape1) is involved in the nucleotide incision repair pathway. Nucleic Acids Res. 2004;32(1):73–81. 10.1093/nar/gkh165 . 14704345 PMC373275

[14] Hang B, Chenna A, Fraenkel-Conrat H, Singer B. An unusual mechanism for the major human apurinic/apyrimidinic (AP) endonuclease involving 5’ cleavage of DNA containing a benzene-derived exocyclic adduct in the absence of an AP site. Proc Natl Acad Sci U S A. 1996;93(24):13737–41. . 8943004 10.1073/pnas.93.24.13737 PMC19409

[15] Demple B, Harrison L. Repair of oxidative damage to DNA: enzymology and biology. Annu Rev Biochem. 1994;63:915–48. 10.1146/annurev.bi.63.070194.004411 . 7979257

[16] Daley JM, Zakaria C, Ramotar D. The endonuclease IV family of apurinic/apyrimidinic endonucleases. Mutat Res. 2010;705(3):217–27. 10.1016/j.mrrev.2010.07.003 . 20667510

[17] Malfatti MC, Balachander S, Antoniali G, Koh KD, Saint-Pierre C, Gasparutto D, et al Abasic and oxidized ribonucleotides embedded in DNA are processed by human APE1 and not by RNase H2. Nucleic Acids Res. 2017;45(19):11193–212. 10.1093/nar/gkx723 . 28977421 PMC5737539

[18] Couve-Privat S, Ishchenko AA, Laval J, Saparbaev M. Nucleotide Incision Repair: An Alternative and Ubiquitous Pathway to Handle Oxidative DNA Damage In Oxidative Damage to Nucleic Acids, edited by Evans Mark D. and Cooke Marcus S. Austin: Landes Bioscience and Springer Science+Business Media; 2007 54–66 p.

[19] Barnes T, Kim WC, Mantha AK, Kim SE, Izumi T, Mitra S, et al Identification of Apurinic/apyrimidinic endonuclease 1 (APE1) as the endoribonuclease that cleaves c-myc mRNA. Nucleic Acids Res. 2009;37(12):3946–58. 10.1093/nar/gkp275 . 19401441 PMC2709568

[20] Mol CD, Hosfield DJ, Tainer JA. Abasic site recognition by two apurinic/apyrimidinic endonuclease families in DNA base excision repair: the 3’ ends justify the means. Mutat Res. 2000;460(3–4):211–29. . 10946230 10.1016/s0921-8777(00)00028-8

[21] He H, Chen Q, Georgiadis MM. High-resolution crystal structures reveal plasticity in the metal binding site of apurinic/apyrimidinic endonuclease I. Biochemistry. 2014;53(41):6520–9. 10.1021/bi500676p . 25251148 PMC4204877

[22] Barzilay G, Hickson ID. Structure and function of apurinic/apyrimidinic endonucleases. Bioessays. 1995;17(8):713–9. 10.1002/bies.950170808 . 7661852

[23] Carpenter EP, Corbett A, Thomson H, Adacha J, Jensen K, Bergeron J, et al AP endonuclease paralogues with distinct activities in DNA repair and bacterial pathogenesis. Embo J. 2007;26(5):1363–72. 10.1038/sj.emboj.7601593 . 17318183 PMC1817638

[24] Richardson AR, Soliven KC, Castor ME, Barnes PD, Libby SJ, Fang FC. The Base Excision Repair system of Salmonella enterica serovar typhimurium counteracts DNA damage by host nitric oxide. PLoS Pathog. 2009;5(5):e1000451 10.1371/journal.ppat.1000451 . 19478870 PMC2680585

[25] Nagorska K, Silhan J, Li Y, Pelicic V, Freemont PS, Baldwin GS, et al A network of enzymes involved in repair of oxidative DNA damage in Neisseria meningitidis. Mol Microbiol. 2012;83(5):1064–79. 10.1111/j.1365-2958.2012.07989.x . 22296581 PMC3749813

[26] Abeldenov S, Talhaoui I, Zharkov DO, Ishchenko AA, Ramanculov E, Saparbaev M, et al Characterization of DNA substrate specificities of apurinic/apyrimidinic endonucleases from Mycobacterium tuberculosis. DNA Repair (Amst). 2015;33:1–16. 10.1016/j.dnarep.2015.05.007 . 26043425

[27] Talebi Bezmin Abadi A. Helicobacter pylori: A Beneficial Gastric Pathogen? Frontiers in Medicine. 2014;1(26). 10.3389/fmed.2014.00026 25593901 PMC4291894

[28] van der Veen S, Tang CM. The BER necessities: the repair of DNA damage in human-adapted bacterial pathogens. Nat Rev Microbiol. 2015;13(2):83–94. 10.1038/nrmicro3391 . 25578955

[29] Tomb JF, White O, Kerlavage AR, Clayton RA, Sutton GG, Fleischmann RD, et al The complete genome sequence of the gastric pathogen Helicobacter pylori. Nature. 1997;388(6642):539–47. 10.1038/41483 . 9252185

[30] Alm RA, Ling LS, Moir DT, King BL, Brown ED, Doig PC, et al Genomic-sequence comparison of two unrelated isolates of the human gastric pathogen Helicobacter pylori. Nature. 1999;397(6715):176–80. 10.1038/16495 . 9923682

[31] Mathieu A, O’Rourke EJ, Radicella JP. Helicobacter pylori genes involved in avoidance of mutations induced by 8-oxoguanine. J Bacteriol. 2006;188(21):7464–9. 10.1128/JB.00851-06 . 16936028 PMC1636264

[32] Huang S, Kang J, Blaser MJ. Antimutator role of the DNA glycosylase mutY gene in Helicobacter pylori. J Bacteriol. 2006;188(17):6224–34. 10.1128/JB.00477-06 . 16923889 PMC1595391

[33] Akishev Z, Taipakova S, Joldybayeva B, Zutterling C, Smekenov I, Ishchenko AA, et al The major Arabidopsis thaliana apurinic/apyrimidinic endonuclease, ARP is involved in the plant nucleotide incision repair pathway. DNA Repair (Amst). 2016;48:30–42. 10.1016/j.dnarep.2016.10.009 . 27836324

[34] Ishchenko AA, Sanz G, Privezentzev CV, Maksimenko AV, Saparbaev M. Characterisation of new substrate specificities of *Escherichia coli* and *Saccharomyces cerevisiae* AP endonucleases. Nucleic Acids Res. 2003;31(21):6344–53. 10.1093/nar/gkg812 . 14576322 PMC275454

[35] Joldybayeva B, Prorok P, Grin IR, Zharkov DO, Ishenko AA, Tudek B, et al Cloning and Characterization of a Wheat Homologue of Apurinic/Apyrimidinic Endonuclease Ape1L. PLoS One. 2014;9(3). 10.1371/journal.pone.0092963 24667595 PMC3965494

[36] Lebedeva NA, Rechkunova NI, Ishchenko AA, Saparbaev M, Lavrik OI. The mechanism of human tyrosyl-DNA phosphodiesterase 1 in the cleavage of AP site and its synthetic analogs. DNA Repair (Amst). 2013;12(12):1037–42. 10.1016/j.dnarep.2013.09.008 . 24183900

[37] Gelin A, Redrejo-Rodriguez M, Laval J, Fedorova OS, Saparbaev M, Ishchenko AA. Genetic and biochemical characterization of human AP endonuclease 1 mutants deficient in nucleotide incision repair activity. PLoS One. 2010;5(8):e12241 10.1371/journal.pone.0012241 . 20808930 PMC2923195

[38] J WE. Molecular cloning. A laboratory manual by T Maniatis, E F Fritsch and J Sambrook. pp 545. Cold Spring Harbor Laboratory, New York. 1982. $48. ISBN 0-87969-136-0. Biochemical Education. 1983;11(2):82-. 10.1016/0307-4412(83)90068-7

[39] Altschul SF, Madden TL, Schaffer AA, Zhang J, Zhang Z, Miller W, et al Gapped BLAST and PSI-BLAST: a new generation of protein database search programs. Nucleic Acids Res. 1997;25(17):3389–402. . 9254694 10.1093/nar/25.17.3389 PMC146917

[40] Yang J, Yan R, Roy A, Xu D, Poisson J, Zhang Y. The I-TASSER Suite: protein structure and function prediction. Nat Methods. 2015;12(1):7–8. 10.1038/nmeth.3213 . 25549265 PMC4428668

[41] Sievers F, Wilm A, Dineen D, Gibson TJ, Karplus K, Li W, et al Fast, scalable generation of high-quality protein multiple sequence alignments using Clustal Omega. Mol Syst Biol. 2011;7:539 10.1038/msb.2011.75 . 21988835 PMC3261699

[42] Taylor WR. The classification of amino acid conservation. J Theor Biol. 1986;119(2):205–18. . 3461222 10.1016/s0022-5193(86)80075-3

[43] Livingstone CD, Barton GJ. Protein sequence alignments: a strategy for the hierarchical analysis of residue conservation. Comput Appl Biosci. 1993;9(6):745–56. . 8143162 10.1093/bioinformatics/9.6.745

[44] Letunic I, Bork P. Interactive tree of life (iTOL) v3: an online tool for the display and annotation of phylogenetic and other trees. Nucleic Acids Res. 2016;44(W1):W242–5. 10.1093/nar/gkw290 . 27095192 PMC4987883

[45] Kulyyassov A, Shoaib M, Pichugin A, Kannouche P, Ramanculov E, Lipinski M, et al PUB-MS: a mass spectrometry-based method to monitor protein-protein proximity in vivo. J Proteome Res. 2011;10(10):4416–27. 10.1021/pr200189p . 21842862

[46] Shevchenko A, Wilm M, Vorm O, Mann M. Mass spectrometric sequencing of proteins silver-stained polyacrylamide gels. Anal Chem. 1996;68(5):850–8. . 8779443 10.1021/ac950914h

[47] Tsutakawa SE, Shin DS, Mol CD, Izumi T, Arvai AS, Mantha AK, et al Conserved structural chemistry for incision activity in structurally non-homologous apurinic/apyrimidinic endonuclease APE1 and endonuclease IV DNA repair enzymes. J Biol Chem. 2013;288(12):8445–55. 10.1074/jbc.M112.422774 . 23355472 PMC3605660

[48] Marchler-Bauer A, Bo Y, Han L, He J, Lanczycki CJ, Lu S, et al CDD/SPARCLE: functional classification of proteins via subfamily domain architectures. Nucleic Acids Res. 2017;45(D1):D200–D3. 10.1093/nar/gkw1129 . 27899674 PMC5210587

[49] Redrejo-Rodriguez M, Vigouroux A, Mursalimov A, Grin I, Alili D, Koshenov Z, et al Structural comparison of AP endonucleases from the exonuclease III family reveals new amino acid residues in human AP endonuclease 1 that are involved in incision of damaged DNA. Biochimie. 2016;128–129:20–33. 10.1016/j.biochi.2016.06.011 . 27343627

[50] Schmiedel R, Kuettner EB, Keim A, Strater N, Greiner-Stoffele T. Structure and function of the abasic site specificity pocket of an AP endonuclease from *Archaeoglobus fulgidus*. DNA Repair (Amst). 2009;8(2):219–31. 10.1016/j.dnarep.2008.10.009 . 19015049

[51] Carpenter EP, Corbett A, Thomson H, Adacha J, Jensen K, Bergeron J, et al AP endonuclease paralogues with distinct activities in DNA repair and bacterial pathogenesis. Embo J. 2007;26(5):1363–72. 10.1038/sj.emboj.7601593 . 17318183 PMC1817638

[52] Lakomek K, Dickmanns A, Ciirdaeva E, Schomacher L, Ficner R. Crystal structure analysis of DNA uridine endonuclease Mth212 bound to DNA. J Mol Biol. 2010;399(4):604–17. 10.1016/j.jmb.2010.04.044 . 20434457

[53] Georgiadis MM, Luo M, Gaur RK, Delaplane S, Li X, Kelley MR. Evolution of the redox function in mammalian apurinic/apyrimidinic endonuclease. Mutat Res. 2008;643(1–2):54–63. 10.1016/j.mrfmmm.2008.04.008 . 18579163 PMC2637456

[54] Vidal AE, Harkiolaki M, Gallego C, Castillo-Acosta VM, Ruiz-Perez LM, Wilson K, et al Crystal structure and DNA repair activities of the AP endonuclease from Leishmania major. J Mol Biol. 2007;373(4):827–38. 10.1016/j.jmb.2007.08.001 . 17870086

[55] Mol CD, Izumi T, Mitra S, Tainer JA. DNA-bound structures and mutants reveal abasic DNA binding by APE1 and DNA repair coordination [corrected]. Nature. 2000;403(6768):451–6. 10.1038/35000249 . 10667800

[56] Wyatt MD, Allan JM, Lau AY, Ellenberger TE, Samson LD. 3-methyladenine DNA glycosylases: structure, function, and biological importance. Bioessays. 1999;21(8):668–76. 10.1002/(SICI)1521-1878(199908)21:8<668::AID-BIES6>3.0.CO;2-D . 10440863

[57] Demple B, Johnson A, Fung D. Exonuclease III and endonuclease IV remove 3’ blocks from DNA synthesis primers in H2O2-damaged Escherichia coli. Proc Natl Acad Sci U S A. 1986;83(20):7731–5. . 2429316 10.1073/pnas.83.20.7731 PMC386795

[58] Cunningham RP, Saporito SM, Spitzer SG, Weiss B. Endonuclease IV (nfo) mutant of *Escherichia coli*. J Bacteriol. 1986;168(3):1120–7. . 2430946 10.1128/jb.168.3.1120-1127.1986 PMC213611

[59] Takeuchi M, Lillis R, Demple B, Takeshita M. Interactions of *Escherichia coli* endonuclease IV and exonuclease III with abasic sites in DNA. J Biol Chem. 1994;269(34):21907–14. . 7520446

[60] Prorok P, Alili D, Saint-Pierre C, Gasparutto D, Zharkov DO, Ishchenko AA, et al Uracil in duplex DNA is a substrate for the nucleotide incision repair pathway in human cells. Proc Natl Acad Sci U S A. 2013;110(39):E3695–703. 10.1073/pnas.1305624110 . 24023064 PMC3785768

[61] Burkovics P, Szukacsov V, Unk I, Haracska L. Human Ape2 protein has a 3’-5’ exonuclease activity that acts preferentially on mismatched base pairs. Nucleic Acids Res. 2006;34(9):2508–15. 10.1093/nar/gkl259 . 16687656 PMC1459411

[62] Lirussi L, Antoniali G, Vascotto C, D’Ambrosio C, Poletto M, Romanello M, et al Nucleolar accumulation of APE1 depends on charged lysine residues that undergo acetylation upon genotoxic stress and modulate its BER activity in cells. Mol Biol Cell. 2012;23(20):4079–96. 10.1091/mbc.E12-04-0299 . 22918947 PMC3469522

[63] Walker LJ, Robson CN, Black E, Gillespie D, Hickson ID. Identification of residues in the human DNA repair enzyme HAP1 (Ref-1) that are essential for redox regulation of Jun DNA binding. Mol Cell Biol. 1993;13(9):5370–6. . 8355688 10.1128/mcb.13.9.5370-5376.1993 PMC360239

[64] Ishchenko AA, Deprez E, Maksimenko A, Brochon JC, Tauc P, Saparbaev MK. Uncoupling of the base excision and nucleotide incision repair pathways reveals their respective biological roles. Proc Natl Acad Sci U S A. 2006;103(8):2564–9. 10.1073/pnas.0508582103 . 16473948 PMC1413785

