# Supplementary material for: Characterization of biochemical properties of an apurinic/apyrimidinic endonuclease from Helicobacter pylori
Source: PLoS One. 2018 Aug 15;13(8):e0202232. doi: 10.1371/journal.pone.0202232 (PMC6093668; doi:10.1371/journal.pone.0202232)
Supplement: S1 File — Domains and their families were assigned according to the Conserved Domain Database [48]. (DOCX) [file pone.0202232.s001.docx]

Turgimbayeva A. *et al*., Characterization of biochemical properties of an apurinic/apyrimidinic endonuclease from *Helicobacter pylori*.

**Supplementary file_S1.docx.** Full-length sequences of 284 Endonuclease–Exonuclease–Phosphatase superfamily AP endonucleases, whose highlighted domains were subjected to the phylogenetic analysis. Domains and their families were assigned according to the Conserved Domain Database [1].

1. Marchler-Bauer A, Bo Y, Han L, He J, Lanczycki CJ, et al. (2017) CDD/SPARCLE: functional classification of proteins via subfamily domain architectures. Nucleic Acids Res 45: D200-D203.

**Conserved Domain Database classification**

(Marchler-Bauer A., Bo Y., Han L., He J., Lanczycki C.J., Lu S., Chitsaz F., Derbyshire M.K., Geer R.C., Gonzales N.R., Gwadz M., Hurwitz D.I., Lu F., Marchler G.H., Song J.S., Thanki N., Wang Z., Yamashita R.A., Zhang D., Zheng C., Geer L.Y., Bryant S.H. (2017) CDD/SPARCLE: Functional classification of proteins via subfamily domain architectures. *Nucleic Acids Res.*, **45**, p. D200-D203)

Domain family color code

ExoIII-like_AP-endo

Nape_like_AP-endo

Mth212-like_AP-endo

Ape1-like_AP-endo

Ape2-like_AP-endo

>WP_078265468.1 exodeoxyribonuclease III [Helicobacter pylori]

MKLISWNVNGLRACMTKGFMDFFNSVDADVFCIQESKMQQEQNTFEFKGYFDFWNCAIKKGYSGVVTFTK

KEPLSVSYGINMEEHDKEGRVITCEFESFYLVNVYTPNSQQALSRLSYRMSWEVEFRKFLKALELKKPVI

VCGDLNVAHNEIDLENPKTNRKNAGFSDEEREKFNELLNAGFIDTFRYFYPNKEKAYTWWSYMQQARDKN

IGWRIDYFLCSNPLKTCLKDALIYKDILGSDHCPVGLELV

>WP_074654508.1 exodeoxyribonuclease III [Terriglobus roseus]

MVIATWNVNSIRARMTHALAFLETRQPDVLLLQELKGLEFPVEEFRAVGYESVAIGQKSYNGVAILSRLP

IEMVTNKLAGDDEDTHARYLETILDMPTKLRVVNIYLPNGNPVGTEKFFYKLAWMDRLIQQMMVWKNDPI

PTIIGGDFNVIPEDIDCDKPANWLRDALFQPEPRDRYRAMKAMGFVDAFRALYPDTRDAFTFWDYFRQAF

ERNRGIRIDHFLLSPGLAPRLTACEIDKTPRAQEKPSDHTPLLVTLA

>WP_058866768.1 exodeoxyribonuclease III [Chloracidobacterium thermophilum]

MTVATWNVNSIRVRLPHVLDWLERHEPDILCLQETKVPDADFPFEAFEQLGYVASVYGQKGYNGVAILSF

EPQQQVRYGLPDDTPDDQRRFLLATIGGVTVASVYVPNGEAVNSPKFAYKLSFLERLTHFLTSHLTPEMP

FLLCGDYNIAPADIDLHDPESNRETVMFHSREHAFLARWRQWGLRDSVREQHPDEPGLYSWWDYRTGGFA

RNRGWRIDHIWATPVLADACLSAGIDRAERGRERPSDHAPVVATFRLPPKTSG

>WP_005033658.1 exodeoxyribonuclease III [Holophaga foetida]

MKFVSWNINGFRSALKNGFMDWLEDTDPDVLSLQEIRAEWDQVDLGVRQMLEANYDICWFPATRKKGYAG

SATLSRKGLGWIHTPGLGIEDYDCEGRIVQSERGDLTLLAGYFPNASSGLVRLPYKRQFALDLAERVRQV

LAQGRQLIVTGDMNVAPEEIDLARPKDNRMSPGFTDEEREDFRGYLASGLRDVLREQNPGTPGLYTWWSQ

RGGARAKNVGWRIDLFLVSEGLMGRVKDTRIHPDTLGSDHCPISLELD

>WP_011688374.1 exodeoxyribonuclease III [Candidatus Solibacter usitatus]

MKIATFNINNINKRLNNLTAWLSRAQPDVVCLQELKAEQRAFPASALRDLGYDGFWLGERSWNGVAIFAR

ECTPVLTRSSLPGNPKDSQARYIEAAVNGVLIASLYLPNGNPQPGPKFDYKLAWFQRLIEHGQELMKAGV

PVVLAGDYNVVPTAQDIYPTRSLDKNALIQPQSRQAYARLLAQGWTDALRTLQPEGPLWTFWDYERGRWP

LGKGMRLDHFLLSPTVSDRLVDGGVDRWVRGEENASDHAPAWIVLDRT

>WP_012992345.1 exodeoxyribonuclease III [Thermocrinis albus]

MAMFRVCTYNVNSIRSRRELLFMWLERSQVDVICLQETKVEERAFPFPDFEKIGYHCYVKGQRTYNGVAI

CSKLPLQDVFKDTSMDILDKESRIIGGRLGDIWLINVYFPHGDLRGTPKFTYKLAFYQQFYQFLAENFSP

EDKIILLGDMNVALEDIDVYDPVLLRDTIGTMEEERSALRKILEWGFVDTFRYLYPNKVQFTWWDYIGGA

VWKDQGMRIDYILVTKPLLPSVRDVYVDMWARKRRSPKPSDHAPVVCLLEL

>WP_026167299.1 exodeoxyribonuclease III [Balneola vulgaris]

MKKISSYNINGVRAAHRKGFNDWVAESNPDVICIQELRADETQIPKEIQDLGYHKAFHSAEKKGYSGVAI

FSKEKPLKIHEGMGTEWIDKEGRVIIAEFESYSVCSVYAPSGTTGDIRQDMKYKFLDDFYAFGEQWVNSE

KPMVFCGDFNICHKEIDIHNPDKQHKTSGFLPEERAWITKFLELGYTDVFRTLHEGEPDLYSWWSYRAAS

KQRNKGWRIDYHFATKTLAEKAVSAEIEMKWNISDHAPVSVVYDI

>WP_014452954.1 exodeoxyribonuclease III [Caldisericum exile]

MKIATWNVNSIRSRVEHVLMLLKDNEVDILGIQELKTEEKNFPFKDFESLGYYVEAFGQKAYNGVALISK

FPFESITKNVLNDTEARTIVGIVKGLHILNVYFPHGGEYGSEKFFYKLEFYQKIKNYILKNNLLDVDFIL

MGDFNVAKEPIDVWDPVMLDGTIGFMKEEREAISDLMSIGLVDAFRHFNPDVQEFTWWDYTSGSFRRNWG

MRIDYILVSKSMLPKLQNCYIEKTFRKLEKPSDHVPVVLEISV

>WP_006928723.1 exodeoxyribonuclease III [Caldithrix abyssi]

MKLISWNVNGLRAVTRNGFIDQVKKLDADIICLQETKLQNEQIPEEILRLTDYHQYWNFAQRKGYSGVAT

FSRQEPAKIERELGIEKFDSEGRVLITHYDSFVLFNVYFPNGQRDHGRVAYKLEFYAELLKMANALRDAG

NKVIITGDFNTAHREIDLKNPKSNQKTSGFLPEERAWIDRYLQEGWIDTFRHFYPQLKDQYTWWSYRFNA

RQRNIGWRIDYFMNSENLLPHLKQAFILPEVMGSDHCPLGIELAF

>WP_013506278.1 exodeoxyribonuclease III [Desulfurispirillum indicum]

MQTFICFNINGIRAHFHQLEAIIQKYQPDVIGLQETKVSDELFPVDAIRELGYCSEFHGQPGHYGVALLS

RAQPNSVRKSFPGDGPDVQRRFIGADYALSDGSRITVLNGYFPQGESRDHPQKFPAKEKFYADLTAYLQQ

CHQPAQQLLLMGDMNIAPTDSDVGIGEDNARRWLRSGKCCFLPEERLWYQSLTDWGLRDAHRLLFPDMQE

LSWFDYRSRGFEREPKRGLRIDHMLVTTPLEEKLLDGGIDYEIRALEKPSDHCPIWVKIRLS

>WP_013009845.1 exodeoxyribonuclease III [Denitrovibrio acetiphilus]

MKVVTFNVNSIRTRIHQLEKLIEKHSPDIIGLQETKVRDEEFPVEEISRLGYHVEFHGQKTHYGVAVMSK

EKPVLVQKGFPSDDSEAQRRLIMCDFGSVKFINGYFPQGESRTHPVKFPAKAKFYSDLLAYLNTSFSVDD

NVLLVGDMNVAPTDYDIGISPDSVKRWLKQGKTSFLPEEREWIKNVADWGLTDVYRKIKPESDEFMSWFD

YRSKGFERDPKSGLRIDHIMATEALADCCVDCGIDYEIRGMEKPSDHAPVWADFQN

>WP_012415412.1 exodeoxyribonuclease III [Elusimicrobium minutum]

MTKRFISWNVNGLRAVAKKGFMEWFTKESPDILALQETKASPEQLEGGLKNPIGYHSYFSTAERKGYSGV

AVYSKEEPLSVSESIGNSTMDGEGRTLVLEFKNFYFINIYFPNGGQGEHRIEYKLRFYNEFLKLTQKLMK

QKTVIVCGDVNTAHKETDLARPKENEGNTGFLPKERAWLDKFFENGLTDTFRLFTKDGGHYTWWDYKTKA

RERNVGWRIDYFMIDTLSKNKVKNSYMLPEVQGSDHCPIALEINL

>WP_096525256.1 exodeoxyribonuclease III [Candidatus Endomicrobium trichonymphae]

MKIVSWNVNGIRAIYKKDFTAWFKDENADIVCVQETKAVETQFPKDIKEINGYNFYCSSAEKKGYSGVAV

WSKIKPDFVIASIENKVFDNEGRIIRLDFKDFIIFNVYFPNGGASQERLKYKMEFYDYLIKYLKQFKDKT

VIICGDYNTAHFSIDLARPKENEKVSGFMPEEREKLDNLVSSGFIDTFRYFNKEPGNYTWWDYKTAARSR

NIGWRIDHFFMSQHSVKRLKSADIKKSVSGSDHCPVSITVF

>WP_032570718.1 exodeoxyribonuclease III [Bacteroides fragilis]

MKIITYNVNGLRAAVNKGLPEWLAEENPDVLCLQETKLQPEQYPAEAFEALGYKAYLYSAQKKGYSGVAI

LTKVEPDHIEYGMGIEEYDNEGRFIRADFGDLSVVSVYHPSGTSGDERQAFKMVWLEAFQKYVTELRKSR

PNLILCGDYNICHEPIDIHDPVRNATNSGFLPEEREWMTRFLSAGFIDSFRTLYPQKQEYTWWSYRFNSR

AKNKGWRIDYCMVSEPVCSLLKEAVILNNAVHSDHCPMALEIG

>WP_012791021.1 exodeoxyribonuclease III [Chitinophaga pinensis]

MKIATYNVNGVNGRLPVLLRWLEESAPDVVCLQELKAPQEKFPEQAIRDAGYNAIWHGQKSWNGVAILAR

NLQIEEIRRALPGDPEDVHSRYIEAVINGDFILGCLYLPNGNPAPGPKFDYKLNWFKRLTAHAKELLAQD

VPVILTGDYNVMPTELDVYKPERWVDDALFRPETRAAFKELVELGWTDAIRKLYPEEKIYTFWDYFRDAY

GRNAGLRIDHFLLSPHFGKRLKAAGVDRDVRGWEKTSDHAPVWIEVKGK

>WP_011585441.1 exodeoxyribonuclease III [Cytophaga hutchinsonii]

MKIATYNVNGVNGRLPVLLRWLQEARPDVVCLQELKAPQEKFPEEAFLQAGYHAIWHGQKSWNGVAILSR

SSDIRELRRVLPGDPEDNHSRYIEAFVNGIVIGCLYLPNGNPAPGPKFDYKLKWFKRLTAHAADLIEADM

PIALIGDFNVMPTDLDVYKPESWIDDALFRPEVRAAFKKLMKQGWTDAIRKLHPDEKIYTFWDYFRNAYG

RNAGLRIDHFLLSPPLAAKLKKAGVDKHVRGWEKSSDHAPTWIIL

>WP_011586837.1 exodeoxyribonuclease III [Cytophaga hutchinsonii]

MKIITYNVNGIRAAITKGLLTWVKATNADVLCFQEIKASVDQFDVESFKALGYTTYAFPAQKKGYSGVAI

CTKEIPVHVEYGCGIEKYDFEGRVIRADYPTFSVMNVYMPSGSSGDDRQAFKFGWMDDFLLYISEVRKKI

PNLIVCGDYNICHKAIDIHNPKSNAKTSGFLPEEREWMSKFFDSGFVDAFRHFNEEPHHYTWWSYRAGSR

GKNLGWRIDYHSVTQEMVGKLKRSVILPDAIHSDHCPVLLEIIP

>WP_035124845.1 exodeoxyribonuclease III [Flavobacterium aquatile]

MRIISYNVNGIRAAINKGFLDWLQQANPDVICLQEIKATQDQIPTEEITKAGYPYQYYFSAEKKGYSGVA

ILSKTEPKNVVFGTGIQHMDFEGRNLRADFEDVSIMSLYLPSGTNIDRLDFKFQYMDEFQNYINHLKNDI

PNLIICGDYNICHEAIDIHDPIRNATVSGFLPSERKWLDGFMKSGFVDSFRHFNKEPHNYSWWSYRAGAR

GNNKGWRIDYNLISENIKHRMTRAVILPEAKHSDHCPIVIEID

>WP_002657987.1 exodeoxyribonuclease III [Saprospira grandis]

MKVLSYNVNGLRAAIRKGFVDWVKENDIDIIGLQEAKALREQVDLEELQAAGYEHIYWHAAEKKGYSGVV

VLSKVKADLEVQGMGIERFDKEGRVLRLDFGDWTLLNCYFPSGTSGSVRQEVKYDFLDSIYDWVQELKKE

RPKILLQGDYNIAHEEIDIHNPKRNHKTSGFLPEERAWMSKWLEEGGFVDSFRQQHPEEQKFSWWSMRSK

TARANNKGWRIDYQCVSEDLVPAIKGAELLNDAVHSDHCPCLIELDV

>WP_002999913.1 exodeoxyribonuclease III [Sphingobacterium spiritivorum]

MKIITYNVNGLRAALKKGWLDWLKSTNADVICLQEIKATPDLIPEIALLEQLGYEHYWYPAQKKGYSGTA

IFTKVSPKRVEYGCGHELYDFEGRIIRADFEKFSVMSTYFPSGTTGETRQTFKYQFLDDFQQYSDRLLKD

IPNLIVCGDYNICHRAIDIHNPKSNANTSGFLPEEREWMENFINSGYVDSFRHLNPEPHQYSWWSYRAGA

RARNLGWRIDYNMVSAPLASQIEKSYLLNDAVHSDHCPVVVEISE

>WP_012845104.1 exodeoxyribonuclease III [Rhodothermus marinus]

MIVATWNVNSIRARLERVLQWLDRTEPDIVCLQETKVVDADFPADPFRERGYELAVFGQKAYNGVAILSR

LPLQDVRRGLDGEQEARFLQARCGDRLVVCSVYVPNGGTVGSEAWDYKLRWFDRLRAYLERAASPETPLL

LAGDFNVAPEPRDVAFPEAWADSVLFHEAARAAFRKLIDWGLVDLIRLHHEGPGPFTWWDYRNLAFPRGD

GLRIDHLLATPPVAACCTEAGVHREERKGKKPSDHAPVWARLNL

>WP_012465126.1 exodeoxyribonuclease III [Chlorobium limicola]

MKIASWNINGIRARNASLEAWILRNQPDILLLQEVKAHEEDIPPAISDLDAYRKFWNGSTVRKGYSGTGI

LLRDSCKVDDVVWEVPDFDIENRTGVLHTTHFTLIGTYVPRGESDEHYRIKLDYLESLRRYICSLLDSGR

QVILAGDMNIAHRDIDVHRSQNKPGAVGLRPEERAAIDAQIASGLQDIMRSMHPDAADLFTWWPYWKPAR

ERNLGWRIDCFYLSSELAAKVTVASVDLEEKSSDHAPVILELSLQDTH

>WP_014559383.1 exodeoxyribonuclease III [Ignavibacterium album]

MKAIRLYSWNVNGIRAVHKKGFVDWVLKENPDILCLQETKAHPDQLPKELISINGYQSFFSSSKVKKGYS

GVAVYSKLNPVDVKYGFDIPRFDDEGRTLILDYKEFILFNIYFPNGKMSDERLKYKLDFYDAFLEYAKKL

IQQGRKIIVCGDVNTAHKEIDLARPKENEKTSGFLPIERQWIDKFLANGFVDTFRMFNDQPGNYTWWDMQ

TRARERNVGWRIDYFFVSESFKNNLKNSFILSDVMGSDHCPIGVEILTDTAN

>WP_015424895.1 exodeoxyribonuclease III [Candidatus Cloacimonas acidaminovorans]

MKLLLWSWNVNGLRAALNKGFIEIIQKEQPDILGIQETKLQEHQIPEELNTLNEYLIYWSHSLRKGYSGT

GLFTKLLPLSFSTGFGNPEFDNEGRINIAEYTEFIFFNIYFPNGQKDDERLNYKLRFYDRCLEVMEEKRT

SGKIILVGGDFNTAHHPIDLANPKENEKTSGFLPIERAWLDKIAEMGWIDTFRYFDKSPEQYTWWSYRTK

ARPRNVGWRLDYFWVNKEGIEHIKKAGIRQDIEGSDHCPVFVELEI

>WP_014546960.1 exodeoxyribonuclease III [Fibrobacter succinogenes]

MKIYSWNVNGIRSVLKKGFEDWFSATDPDILCLQEVRAEKNQVSEIATREGYYTYWNACKRKKGYSGVAV

YSKIEPDAVNYGFDIEEFDEEGRVLQLVFPDWVLNCIYFPNGGQGDDRLDYKLRFYDAFLENSKQWLADG

KHVVTVGDYNTCHKEIDIARPKENENVSGFLPIERAWMDKYVENGFVDTFRTLHPDTRDAYSWWSNRFGA

RERNVGWRLDYGFVDAALMPNVVSSEILSNVMGSDHCPISLELEPPFAPIPISHD

>WP_023037532.1 exodeoxyribonuclease III [Fusobacterium nucleatum]

MKLISWNVNGIRAAIKKGFLDYFNEQNADIFCLQETKLSEGQLDLDLKGYHQYWNYAEKKGYSGTAIFTK

EEPLSVSYGLGIEEHDKEGRVITLEFGKFYMVTVYTPNSKDELLRLDYRMVWEDEFRKYLKNLEKKKPVV

VCGDLNVAHKEIDLKNPKTNRRNAGFTDEERGKFTELLDSGFIDTFRYFYPNLEQVYSWWSYRANARKNN

AGWRIDYFVVSKGLEKNLVDAEIHSQIEGSDHCPVVLFLEFNK

>WP_005005685.1 exodeoxyribonuclease III [Nitrospina gracilis]

MLIYSWNVNGIRAVAKKGFGDWMESVDPDVVCLQETKAHPEQVPPEVAYPEGYEAYWCPATRKGYSGVAV

FSKTKPKQVHYGMGIERFDSEGRYLRLEFTKFDLLNIYFPNGTSGDERLQFKMEYYDAFLDHCETLRKKQ

KKKLVICGDVNTAHREIDLKNAKQNEKNSGFLPMEREWIDKFIAHGYVDTFREFCQEPDNYTWWTYRANA

RARNIGWRIDYFFVTRDLLKNVKKSFITPEVMGSDHCPIGLEIV

>WP_053381869.1 exodeoxyribonuclease III [Nitrospira moscoviensis]

MKIATFNVNSLRKRVGIVADWLERHRPDVLCLQETKVQDSEFPLLALASCGYEITYRGMKSYNGVAVLSR

KKPEAVFHGFDDGGDPDEARLLRVVIGGLPIINTYVPQGYEITSPKYAYKLGWYERLRRYCDKHLSPDAP

ALWCGDMNVAPRPIDVHSPEKHLKHVCYHEDARKAYEQVLAWGWQDVFIRLYPDRQQYTFWDYRAPSSLE

ANKGWRIDHILATAPLAERCVRVEVDVEPRRAKDPSDHTFLWAEFSI

>WP_024893407.1 exodeoxyribonuclease III [Acidithiobacillus thiooxidans]

MKVASWNVNSLKVRLPQVLEWLQEKSPDVLCLQETKLSDEKFPVAELAEAGYHALYHGQPTYNGVAILSR

SVPEDGRCDWPDGSDGQARLCAGTFAGTRVVNVYVPNGQSLESDKYPYKLQWLERLRALIAKELQQWPRL

LLVGDFNIAPDDRDVYDPVAWGEEVLCSPPERAALTALLNLGLQDSWRHQHGDLQEWSWWDYRAAGFRRN

RGLRIDLILASNALMTKVQDVVIDRNARAAERPSDHAPVLIQLEEV

>WP_010640564.1 exodeoxyribonuclease III [Acidithiobacillus thiooxidans]

MRLYSWNVNGWRAACRKGLQDWVKETGPDLLCTQETKADPAKLTAAESRLEGYGSCWAVAERAGYSGVAT

FSKIPGQTSHAGLGIERFDREGRVVVTDCGDFDLYNVYFPNGKKDSERLAFKLDFYAAFLAHINQKAAAG

RPVIFCGDVNTAHQPIDLARPKENQRISGFLPEERACMDQWQQAGWVDSFRHLHPDEARYSWWSQRTDAR

SRDIGWRLDYFWVHQSLLPRLRAAGIATEVMGSDHCPVWLEII

>WP_058349444.1 exodeoxyribonuclease III [Caulobacter vibrioides]

MRIATWNVNSVNARLENVLAWFEAEAPDVAVLQEIKCVDEKFPAEAFERLGYNVVVHGQKTYNGVALLSK

HPVEDVRKGLPLLDGDAEDEQARYVEAVISGPKPVRVIGIYLPNGNPIGTEKFAYKLSWMRRLHAHAKGL

LAFEEPLVIAGDYNVIPEVEDVANPEAWLGDALFQPESRAAFRALKNLGFTDAYMQADGTPGGYTFWDYQ

AGAWPRNLGIRIDHLLLSPQAADKLAQVQIHRDERDKEKPSDHVPVVATLDL

>WP_043190389.1 MULTISPECIES: exodeoxyribonuclease III [Burkholderia]

MKIATWNVNSLNVRKQHVLDWLAQSGTDVLCLQELKLPDEKFPRAELEAAGYRSWFTGQKTYNGVAILAR

DTLSVDEADVVRNIPGFDDPQQRVVAATVDGVRIVSAYFPNGQAPDSDKFVYKMQWLDALQAWLRTELQR

YPKLALLGDYNIAPEDRDVHDPAKWEGQNLVSPQERAHFAQLIELGFVDAFRRFEQPEKTFTWWDYRMMA

FRRNAGLRIDHVLLSPALAETCTSCEVDRTPRTWEQPSDHTPVVAVVG

>WP_059521082.1 exodeoxyribonuclease III [Burkholderia cepacia]

MMRVITANLNGIRSAAKKGFFEWLGEQNADCVCVQEIKVSADDLPAEFVEPHGFKSYFHHAEKKGYSGAG

VYSRREPDDVIIGFGSSEFDAEGRYVEARYGKLSVVSVYVPSGSSGEERQQAKYRFMDEFMPHLAELKKK

REVILCGDVNIVHKEIDIKNWKSNQKNSGCLPEEREWLTKLFDDVGYVDVFRTLDPRAEQYTWWSNRGQA

YAKNVGWRIDYQIATPGVAGTAKSTSIFKDIKFSDHAPLTVDYDYKK

>WP_002225717.1 exodeoxyribonuclease III [Neisseria meningitidis]

MLKIISANVNGIRSAYKKGFYEYIAASGADIVCVQELKAQEADLSADMKNPHGMHGHWHCAEKRGYSGVA

VYSKRKPDNVQIGMGIEEFDREGRFVRCDFGRLSVISLYLPSGSSAEERQQVKYRFLDAFYPMLEAMKNE

GRDIVVCGDWNIAHQNIDLKNWKGNQKNSGFLPEEREWIGKVIHKLGWTDMWRTLYPDVPGYTWWSNRGQ

AYAKDVGWRIDYQMVTPELAAKAVSAHVYKDEKFSDHAPLVVEYDYAAE

>NP_416263.1 exonuclease III [Escherichia coli str. K-12 substr. MG1655]

MKFVSFNINGLRARPHQLEAIVEKHQPDVIGLQETKVHDDMFPLEEVAKLGYNVFYHGQKGHYGVALLTK

ETPIAVRRGFPGDDEEAQRRIIMAEIPSLLGNVTVINGYFPQGESRDHPIKFPAKAQFYQNLQNYLETEL

KRDNPVLIMGDMNISPTDLDIGIGEENRKRWLRTGKCSFLPEEREWMDRLMSWGLVDTFRHANPQTADRF

SWFDYRSKGFDDNRGLRIDLLLASQPLAECCVETGIDYEIRSMEKPSDHAPVWATFRR

>WP_013941741.1 exodeoxyribonuclease III [Myxococcus fulvus]

MKIATWNVNSVRARQERLLAWLKKAQPDVLCLQELKCVDEDFPMEAVRELGYHAAVHGQKTYNGVAILAK

EEPKDVVKGLSDGVDDSHARLIAATVGGIRVVSAYAPNGQSVDSPQYQYKLEWYGRLRRYLDARHKPDEP

LVLGGDWNVAPEDLDTYDPKLWEGQTLFTLKERDALQHLGAFGLSDAFRKLHPGVQKFTWWDYRMLGFPK

NLGLRIDHLYVTAPLAERLTVVDVDREERKGKQPSDHAPVWLELRD

>WP_043711384.1 exodeoxyribonuclease III [Myxococcus fulvus]

MRVVSWNVNGLRSVHRKGFLPWLAGCRAQVVGLQEVRARADQLPDEVRSPPRWKTHFVAAERPGYSGVGL

YSRLEPDEVVTVLGVGELDVEGRLQIARFGKLTVVNGYFPNGNGKDRDLSRIPYKLTFYRRLFERLEKPL

RDGGRVLVMGDFNTAHQEVDLARPKENRETSGFRPEEREEFDRWIRAGWVDTFRHFNKGGGHYSWWSQRA

GVREKNIGWRIDYVLASPAAMAYVRKAGIHPDVLGSDHCPVSVDLDPAVR

>WP_075539881.1 exodeoxyribonuclease III [Campylobacter fetus]

MKLISWNVNGLRAAVSKDSFAWLDSVKPDFLGLQETKASEDKIPSEIYSLGFKEININSAARAGYSGVMS

LVNFDTLCTKSLFFDDNEGRVLEHRFGNVVLFNIYFPNGQKDEERLSYKMKFYANFLAYLDELKAQNFDI

IICGDVNTAHQEIDLKNPKANAKTSGFLPIERAWIDELLSHGFIDTFRQIHGDEVKYSWWSYRFKAREKN

IGWRIDYFFISESLKDRLKDAFILDEIYGSDHCPVGIEIDL

>WP_028875008.1 exodeoxyribonuclease III [Tepidiphilus margaritifer]

MKLATWNLNSITARLPHLQRWLADARPDVLALQELKCPAEKFPVHAIAELGYTAAVHGQKTYNGVALLVR

EGLPWREVVRSVPEFDDEQARLIVAEVDEPGSQTPLWVASAYFPNGQAVGSDKFAYKLAWIEALASWIER

ERLTERPFVLMGDFNIAPAAEDTHPDWPQDGILASPPERAAFARFVSLGLVDAFRRFPQPPQSYSWWDYR

QGAFRRNLGLRIDHILVSPPLLPRLRACTIDVEPRRWERPSDHAPVVLELADLT

>WP_028874015.1 exodeoxyribonuclease III [Tepidiphilus margaritifer]

MRILTLNLNGIRSAADKGFFRWLPTQDADIVAIQELKAQEQDLSEEMRAPDGYTGHFHFAEKKGYAGVGL

YLRQPPLELVAGIGEPEFDAEGRYLEAIYPGLSVVSLYLPSGSAGPERQASKFRFMDAFFPKLRELRHCG

REVVICGDWNIAHTEKDLKNWRSNQKHSGFLPEERAWLTRVFDELGWVDVYRRLYPEATEEGYTWWSNRG

RAWEKNVGWRIDYQIATPGIAAAARTARVYKDERFSDHAPLIVDYDFALAQNPR

>WP_051431523.1 exodeoxyribonuclease III [Bdellovibrio bacteriovorus]

MGRIFKGDLDLKIISWNVNGIRACYKKGLMDFVKSQAPDIFCVQETKAHIDQVEVEVKNLGYKNSYWSSA

MKKGYSGVATFTNLDPLKIEFQFDNIAEYESEGRIVMTDHGEFDLYNIYFPNGGSGLERHNFKQQFLKDL

NKHLEAKLATGRQVIVVGDYNVAHENIDVHDPIRLSKESGFLPEERAWFDSFLDLGFIDTFRYFHPQAQE

RFSWWDYRKLARISNRGWRIDYVCVSKGLEKNLVSADVLDQVEGSDHCPVLVNLKF

>WP_061835073.1 exodeoxyribonuclease III [Bdellovibrio bacteriovorus]

MKLISWNVNGLRSVQRKNFREWFEEEKADVVCLQEIKITDEAIKKDETFYHPAKYNSSWAFAEKAGYSGL

ALYSKKEPDDVRVGLGIEKFDREGRWLEADFGPITVVNSYFPNSQRDHARLPFKLEFCAAAEKRLQALRK

KGREVMICGDFNIAHKEIDLRNPKSNAKNAGFLPEERAWMTKFIDKLEWVDSFRKFEQGPDHYTWWSYRP

GVREKNVGWRLDYFLVNKEASDRLKAAAHLPMVFGSDHCPVRLTLKK

>WP_018295000.1 exodeoxyribonuclease III [Mariprofundus ferrooxydans]

MKITTWNVNSLNVRLPHVLAYLRDEQPDVLALQETKTPDAGFPVAELEAAGYKVIFSGQKTYNGVAMLAR

SSMQQVVNELPGLDDPQRRLLAATVDGIRVVNVYIPNGQEIGSDKYAYKMHWLQALCDFLQEELKQHDRL

VLLGDFNIAPADIDVHDPTRWQGKILCSEPEREFFSALLQLGLIDGVRSLNPEKAMFSWWDYRMSAYRRG

WGMRIDHLLVTRALALSAAGVAVKYRELDRPSDHAPVWIEFE

>WP_009851306.1 exodeoxyribonuclease III [Mariprofundus ferrooxydans]

MKIITANLNGIRSATRKGFWEWFAAQDADILCLQELKAHIHQLPPESLLKDYHRHYHFAQKPGYSGVALY

SRKQPDTVHVGLGSIDPATDWSRFDAEGRFVMTDFGTLSVISAYFPSGSGTPERQAGKMVFLEQFLPLIT

RLKDQGRELIICGDINIAHRNIDLKNWRGNRKNSGFLPEERAWFDQCLNAGFVDLFRQLYPEREQYSWWS

NRGQARANNVGWRIDYHLCTQKTAATACEVSVFTDAWFSDHAPVTANFNDLST

>WP_052882517.1 exodeoxyribonuclease III [Kiritimatiella glycovorans]

MKIATFNVNSIRTRLPIVLDWLKEHEPDVLAVQETKTPDDQFPAGEIEAAGYRVGYRGFKGGAGVAVISR

DEPEEVRFGFDDGGPADEDRLIEARWPGLTLINTYVPQGRSIDHEMYAYKLEWLGRLRRYLARRHDSEEP

LIWVGDLNVARGPNDVAKPENKKKHVCYHEDVRAAFEEVLEFGFEDVFRRHHPDEKLFSFYDYRVKDALG

TNRGWRVDYILATEPLAERCTGCAIDLEPRRRERPSDHTVVHADFDCPPGRTGG

>WP_007281074.1 exodeoxyribonuclease III [Lentisphaera araneosa]

MSVKLISWNVNGIRAVEKKGFVDLMQSWDADVICLQETKAHKEQLSDELINIGNYTSYWHSGEKKGYSSV

AIYSRIEPIRVIEGLGIEEFDREGRVIIAEYSDFYLFGVYFPNAQAELKRIDYRLEFGDALIEQLKEKYS

DKACVLCGDFNVAHKAIDLKNPKANEKNPGYSIQERNWMDKFLAAGHVDTFRMFNQNPDEYSWWSYRGGA

RSRNVGWRIDYFCVSESARDRVLDAGIMQEVLGSDHCPVSIELESSQG

>WP_068420000.1 exodeoxyribonuclease III [Planctomyces sp. SH-PL62]

MKIATYNVNSIGARLPVLLHWLSSSKPDVACLQELKTVDEKFPVEAVRDAGYEAVWHGQKAWNGVAILSR

DEKPREVRRGLPGDPDDAQSRYIEAVVGGITVGCLYLPNGNPAPGPKFDYKLKWFERFQAHAAGLLESGG

PVVLAGDYNVIPTDRDVYKPERWVDDALFLPETREAYQRLLAQGWMDALRAMHPDETLYTFWDYMRGAFS

RNAGLRIDHLLLSPELAPRLKAAGVNKDVRAMAKTSDHAPTWVEVE

>WP_085754611.1 MULTISPECIES: exodeoxyribonuclease III [unclassified Phycisphaerae]

MLTATFNANSIRTRKEIIKDWLEEKSPDILCIQETKVQDKDFPSSFFEDIGYNVYFSGQKSYNGVAVLSR

EEALKVEYGLDTEPEDKSRLVYAQYPEINIVNTYIPQGNSPDSDKFQYKLDWYKRLKAFFSRKFSPEDNV

LWLGDMNVAPENIDVHNPEGLLGHVCFRQETWDAFKDVMNWGFTDILRKHYPEDVIYTFWDYRAGSFPNN

KGWRIDHILATAPLSERSIKCFVDLEPRKKHKPSDHTFLAAEFDI

>WP_013043365.1 exodeoxyribonuclease III [Coraliomargarita akajimensis]

MSPTHLLSWNVNGLRAVLKKGFDDFLASQQPDVLCLQETKISADLTADFAFAGYPYVYWNCAEKKGYSGT

AIISQIEPISVRYGLGIEKHDQEGRVITAEFEDYYLVTVYTPNSQNHDENKRPRRLDYRTLEWDVDFLAY

VKGLEVTKPVVFCGDLNVAHTEIDLANPKTNRKNAGFTDEERGRFDAIIEAGFIDTFRHLYPDRTEQYSW

WSYRAAARQRNIGWRIDYFCVSDAVKNQIRDATILADTLGSDHCPVGLRLKG

>WP_075078727.1 exodeoxyribonuclease III [Terrimicrobium sacchariphilum]

MKLVSWNVNGIRSVLGKGLHDMLATAQADIVCFQETKARQEQVGATFDGYEIYWNAAEKPGYSGTAIFTK

TTPLSVRNGIGHDHHDREGRVITAEFADYYVVNVYVPNSQRELTRLDYRVNSWGPDFLAYLKELEKTKPV

IFCGDLNVAHKEIDLARPKGNINNAGFTPQERASFDTYIGGGFIDTFREFQPATGHYTWWSYQSNARERN

IGWRIDYVCISPALRPRLKDAFIWPHVTGSDHCPVGVVLE

>WP_050031009.1 exodeoxyribonuclease III [Verrucomicrobium sp. BvORR034]

MKIASYNVNGINGRLPVLLRWLKEAEPDIVCLQELKAPHEKFPLPPIQRAGYEAIWHGQKSWNGVAILSR

VGKIQEVRRQLPGDPQDSHSRYIEGIVSNMVIGCLYLPNGNPAPGPKFDYKLAWFKRLITHSKALLKYDV

PVMLIGDFNVMPADIDVYKPESWQEDALFRPEVRRAFKRLTDLGWTDAIRKLHPQERIYTFWDYFRNAYA

RNAGLRIDHFLLNRHLENRLKKAGVDKHVRGWEKTSDHAPVWMQLKDR

>WP_010881574.1 MULTISPECIES: exodeoxyribonuclease III [Treponema]

MRPVQRIISWNVNGIRAIERKDFLSWLAREAPDVLCLQEIKAHESQLSAALRAPVWSAGAGGTYYTYFHS

AQRPGYSGTALFSKRAPDAVRFFGVPAFDCEGRMLAARFGELTVVSAYFPNAQEGGKRLAYKLDFCAAFR

AFCDEERTAGQHVILCGDYNIAHKEIDLAHPQENEGNPGFLPQERAWMDTFTEAGYADSFRAFCTEGQQY

TWWSYRARARARNIGWRIDYQCVDQAFLARVTSSQILSEVTGSDHCPVCLTYAD

>WP_037978080.1 exodeoxyribonuclease III [Synergistes jonesii]

MPDIKIATFNVNSVKSRLPLLEGWLAHSGAPDILCLQETKCRDEEFPSAFFEGRGYRCVYKGMKSYNGVA

VISREAPDEFEFGLCDGEEDGREESENARVVRARFGPLTVLNTYIPQGKEIDNPDYPYKLRFIARVRGLL

ERKCTPEDRVVWLGDLNVAPTDIDVTNPKNKKEHVCFHEDVKKALKDAMGWGLVDIFREQLPGAGEYTFW

DYRIKNALERNIGWRIDHILGTKSVAARCTGVKVERGLRAAERPSDHTAVVASFSFD

>WP_041661810.1 exodeoxyribonuclease III [Acidimicrobium ferrooxidans]

MRLATWNVNSIRARIELVVAWAERVAPDVLLLQETKVATDQFPADSLVSLGYHVTHWGTGAWNGVAVLTR

EAPREVRYGTGFARAPLATEEPRALAVRLEAGWFMSVYVPNGRALDDPHYVYKLTWLAELRAHLADLVAD

GVVVGGDFNVAPSDHDVWDPSALEGATHVSLPERRALDALLGLGLTDVYRALHPDDPGFTWWDYRQGAFR

RGMGMRIDLVLASEDIARNVVAVDVDIEARRAPRPSDHAPLVVDLA

>WP_065572820.1 exodeoxyribonuclease III [Micrococcus luteus]

MKIATWNVNSLRARADRVEAFLERHDIDVLAIQETKCRDENFPWELFERAGYEVVHNGTSQWNGVAIASR

VGLSDVQRGFPGQPHFGKGGVDPQEESRAIGATVGAETADGVAPVRLWSLYVPNGRGLEDEHMGYKLEWL

RVLQEHAAARLAEDPDTRLALAGDWNIAPQDEDVWDIELFQREGYTHVSPAERAAFHAFEDAGLVDVVRP

RHPGPGVYTYWDYTGLAFPKKKGMRIDFQLCSPALAQTVTDAWIDREERKGKGASDHAPVVVELG

>WP_031351974.1 exodeoxyribonuclease III [Mycobacterium tuberculosis]

MRLATWNVNSIRTRLDRVLDWLGRADVDVLAMQETKCPDGQFPALPLFELGYDVAHVGFDQWNGVAIASR

VGLDDVRVGFDGQPSWSGKPEVAATTEARALGATCGGIRVWSLYVPNGRALDDPHYTYKLDWLAALRDTA

EGWLRDDPAAPIALMGDWNIAPTDDDVWSTEFFAGCTHVSEPERKAFNAIVDAQFTDVVRPFTPGPGVYT

YWDYTQLRFPKKQGMRIDFILGSPALAARVMDAQIVREERKGKAPSDHAPVLVDLHAG

>WP_013709570.1 exodeoxyribonuclease III [Coriobacterium glomerans]

MPDMQRAGSQPERADERIYRFASWNVNGLRALMKKEPDFSEIAASLNADILAIQETKLQEGQIDLDLAGY

HQTWSYAARKGYSGTAVFSREQPIRTLRADAMLDVVEGGELLEPAIREGRVCALEFERFWFADVYTPNSQ

DGLARLPVRLAWDEAYRAFLKQLERMGDKPVVTCGDFNVAHNEIDLRNPKSNRGNAGFSDEERASFSRLL

EAGFTDTFRYLYPDVRDAYSWWSYRFHARRTNAGWRIDYFLVSDSVRDRIVEATIRQDVFGSDHCPVELV

IAL

>WP_052666075.1 exodeoxyribonuclease III [Nitriliruptor alkaliphilus]

MRVVSWNLNSLKARLPRVLELLEVHAPDVLLVQETKCAAVAFPREELALAGYEVIDHCEGRWNGVALLVP

QGEEVTDVVTGLSGEPNEAEARWIEGTVRGIRFISVYVVNGRTIDDPMFQVKLDFLDAAHLRLRELLAAG

PVVVAGDWNVAPHDRDVWDPALFVGSTHVTPDERIRYQALLDLGLQDAWTTLHGDEVGFTYWDYRAGAFR

RNMGMRIDAALVSQDLTIRSIAVDTVFRRNNQAGDKPSDHAPLVLSLDL

>WP_012932552.1 exodeoxyribonuclease III [Conexibacter woesei]

MRLVTWNVNSLRVRLPRVLEFLAEHRPDVLCLQETKTDAAAFPHAELAAAGYVAADHSGGRWAGVAIVAR

TELGVADPAVGLPGEPAQDESRWIEATVGGELRVASVYVPNGRELESEWYAAKLEFLDAMARRVGELRGD

DAGAPLPLVVAGDMNVAPSDLDVYDPAVFATSTHTSAAERTALRAVEQAGLLDAYRQLHPDDVGYTWWDY

RQGHFHRGLGLRIDLILASPDLAERLTRVGIERDYRKGSRPSDHVPLVADWT

>WP_027004881.1 exodeoxyribonuclease III [Conexibacter woesei]

MRIVTWNVNSVKQRLPRLLPWLDERRPDVVCLQETKLTDDAFTELFGDELSTRGYEVATYGEVSWNGVAI

LSKVGLEDVVRGIPGGPGFPDPEARAVSATCGGVRVTGVYVPNGRSPGSDHYNYKLAWLKALKDMVAPDA

ARAVVCGDINIAPTDDDVFDPEAYEGHTHVTAPERDALADLQSIGLHDVVRDRWPDERRLFTYWDYRAGM

FHQDLGMRIDLVLAGEEIAPRVAAAWVDRKARKGKGPSDHAPVIVDLDEAPDGDIGPVVPPASAPAPLRG

AKRLPQGSPAD

>WP_025228746.1 exodeoxyribonuclease III [Fimbriimonas ginsengisoli]

MKVVTYNAASIRARMPLLVEWLAENEPDVVAIQETKVEDDKFPVEPLADLGYEVAFHGQKSWNGVATLSR

RPIVNSRFGFGDELMPNDARVLTCEIDGVTYINTYVPNGNTVGSDKFEYKLRWLDRFRQYLDQNFRTDDP

LVWLGDINVAPTPDDVYDSKRFYGGVGHHPDEFSRLSKIVDFGLTDVFRKFVQGPGHYTFWDFTLPRGVD

RNLGWRIDHIYATEPLARLCTDCVIDKAARQLERPSDHTFLTATFDM

>WP_025229521.1 exodeoxyribonuclease III [Fimbriimonas ginsengisoli]

MKIATYNVNGINGRLPVLLRWLEMAQPDVVCLQELKAPQEKFPEAAIREAGYGAIWHGQKSWNGVAILAK

GGAPEETRRGLPGDPEDIHSRYIEAAINGIVVGCLYLPNGNPAPGPKFDYKLAWFERLTTYAKSLLNHEV

PVVLAGDYNVIPTELDCHKPASWVDDALFFPESREAYRKLVAQGWTDALRTLHPEEKIFTFWDYFRNAYA

RDAGIRIDHLLLSPQIAGHLVAAGVDRDVRGWEKSSDHAPTWIEINSA

>WP_087862190.1 exodeoxyribonuclease III [Brevefilum fermentans]

MKITTWNVNGFRAVLRKNAFEWIPDVDPDVLCFQEIKATLDQISAEEAIIEPYEGIWNPAERKGYSGTAT

YYKNKPLSHEKGFGIEHFDIEGRVIRLKYPDFYLYNIYFPNGGEGNKRVPYKLEFYEAFLEICDDLHAQG

ENIIITGDFNTAHQEIDLANPKQNEKNTGFLPEERVWIDRYLEHGFKDAYRELYPEEEKYTWWTYRFNSR

ERDIGWRLDYYLVSDALMDRVEDVVIHSDIMGSDHCPVSLILKE

>WP_095044504.1 exodeoxyribonuclease III [Candidatus Promineofilum breve]

MLTLYSWNVNGLRAVHRKGIFLDWLARTQPDILCLQETKCRPDQLSEDVLHPPGYYTYWAVSERPGYSGV

ALYCKQPPLSVQVGLGLPDFDREGRTLVADFGDFTLVNAYFPNGSRDHSRLPFKMQYNADFLDTIENLRQ

GGQSVIFCGDVNTAHQPIDLARPRQNMNATGFMPIEREWIDRVVERGYLDTYRTLYPDTTGAYTWWAQVT

FSRQKNVGWRLDYFFISPDLRPRVMDAAIHPDVLGSDHCPVSLTLAIETDDQ

>WP_015153953.1 exodeoxyribonuclease III [Chroococcidiopsis thermalis]

MKIATWNVNSVRTRLEQVIDWLRQTPVDVLCLQETKVVDADFPRSPFEHLGYYVYASGQKSYNGVAILSQ

QPLQDVSTGFAPLLVESGIVEAELLAALDEQKRVIAGTLDRVRIVNLYVPNGAAVGSEKYEYKLGWLKVL

REYLRSQLLVSPVMCVCGDFNIAPDERDLHDPDKLTGQIMASELERQALQEILALGFADAFRKFITEGGH

YSWWDYRTAAFRRNLGWRIDHLYLSPQLYQQAIACWIDPAPRKLPKPSDHTPVVVEF

>WP_041244387.1 exodeoxyribonuclease III [Gloeobacter kilaueensis]

MTVASWNVNSITVRLAQVIDWLNVHRPDVLCLQETKIPDERFPKAAIEAVGYQVVYSGQKAYNGVAILSR

LPISQVRSSLPGDDETAQKRLIAATIAGIEVVNVYVPNGSEVGSEKFAYKLAWLERLYRWFERDFDPQGA

VLLCGDFNIAPEARDVYDAQAVAGKVLFHPDEHAALARLQQWGLIDTLRIHTEEAGLFSWWDYRAAAFRR

NLGMRIDQIWVSAPLAARCTAGWIDRQMRAADSPSDHVPVVASFEIDSEARDPGGSVLELESR

>WP_012409238.1 exodeoxyribonuclease III [Nostoc punctiforme]

MKIATWNVNSIRTRLEQVTDWLTNNPVDVLCLQETKVADAEFPRSPFEQLGYNLYISGQKSYNGVALISR

QPLLNVSSGFRAILPDLHHEWDEQKRVITGVIDGVRIVNLYVPNGAAVGTEKYEYKLRWLTALHEYLRVL

ALSEPAICVCGDFNIALEDKDIHEQVSTENHIMATETERQALREILQLGFADAFRKFTTEGGNYSWWDYR

AAAFRRNLGWRIDHHYLTPVLYERATSCIIDAEPRKSTQPSDHTPVIVEF

>WP_015151074.1 exodeoxyribonuclease III [Oscillatoria acuminata]

MKIATWNVNSIRTRLEQVTDWLQTNPVEVLCLQETKVVDTDFPRSAIEALGYHLYISGQKSYNGVALFSK

IPLTDVSIGFTPILGPEVTELDEQKRVITGVFGDIRILNLYVPNGSSVGSDKYSYKLTWLKTLQDYLKII

LDKTDKLCICGDFNIALEARDIHRKTTPNDIMASPAERAALTEILELGLTDAFRLFNQEPGQFSWWDYRA

GGFARNRGWRIDHHYVTPKLKERAIACTIDTAPRKLLKPSDHTPVILELE

>WP_072622354.1 exodeoxyribonuclease III [Spirulina major]

MKIATWNVNSIRSRQAIVLDWLAANPVDVLCLQETKVVDKDFPCAPFEEIGYQLAISGQKSYNGVAIFSR

QPMTEISAGFGAVLGEETDLDEQKRVITAVIGGVRVVNLYVPNGSAIKSEKYDYKLRWFATLQTYLAQLQ

SSIVSDLCVCGDFNVAPDDRDIYKPDGKDKHIMASPPEREALASVLGLGFTDIFRHFTPDGGHYSWWDYR

QGGFAKNRGWRIDHIYLTDALCDRAQSCTIDLEPRRREKPSDHTPVIVDLRD

>WP_041443459.1 MULTISPECIES: exodeoxyribonuclease III [Synechococcus]

MEIATWNVNSVRSRQTHICQWLEQTGVDLLCLQETKVVDPDFPRQPFEALGYHTSISGQKSYNGVALLSR

EPLQDVIIGFTPVVGAAIAQDFDAQKRVISGVIGDVRVVNLYVPNGSAVGSEKYDYKLGWFEVLKAYLKE

LCKDDREILMCGDFNIALEDRDIYMPKKPDHIMASPAERETLTAILDFGFQDVFRKFNQEADQFSWWDYR

TRGFSRNRGWRIDHIYLTEKLYGQAKRCWIDREPRGWEKPSDHTPVIVEL

>WP_012695287.1 exodeoxyribonuclease III [Deinococcus deserti]

MKLATWNVNSLNVRLGQVMAWLEAQQPDVLALQETKLPDDRFPVAELEALGYRAVFSGQKSYNGVALLSR

LPLEDVQIGIPGLDDEQRRVVAATVGGMRVVCLYVPNGQAVDSPKYTYKLEWLSAVRAWLQLELMAHPRL

AVVGDFNVAPEDRDVHSPKRWAGQVLVSEPERQAFRALLDLGLHDAFRLHPQPERVFSWWNYGRLGFPRN

WGLRIDHVLVSGILAAECQSCTVDLEPRRHERPSDHAPVVATFSTFTCRPENPAQVPQKP

>WP_012692269.1 exodeoxyribonuclease III [Deinococcus deserti]

MTSASPSSASALKVTTLNVNGLRSALRKGLRDWLLREAPDVLLLQEVRAGPMPEALQDLGYDGAWFPAQK

AGYSGVAVLSRHPLRDIRAGMLHDEMDAEGRVVSAVVQGVRFVSVYLPSGSSGELRQGFKDRILDDYHTW

VQALLDEQTPVVIGGDYNIAHREIDLKNWRSNRKNSGFLPHEREWMTAHLSAGLVDCHRNCLGEAAEYTW

WSNRGNAYANNVGWRIDYLLSAGVQVRGVCVDREARLSDHAPLTGWVERS

>WP_014666138.1 MULTISPECIES: exodeoxyribonuclease III [Bacillus]

MKLISWNVNGLRAVMRKMDFLSYLKEEDADIICLQETKIQDGQVDLQPEGYHVYWNYAVKKGYSGTAVFS

KQEPLRVMYGIGIEEHDQEGRVITLEFENVFVMTIYTPNSRRGLERIDYRMQWEEALLSYILELDKKKPV

ILCGDLNVAHQEIDLKNPKANRKNAGFSDQERGAFSRLLEAGYVDSFRHVYPDLEGAYSWWSYRAGARDR

NIGWRIDYFVVSESLKEQIEDAGISKDVMGSDHCPVELIINI

>WP_043662070.1 exodeoxyribonuclease III [Clostridium butyricum]

MTLKKLISWNVNGLRACVKKGFLDYFNEMDADIFCVQETKLQEGQIDLELEGYYDYWNYAEKKGYSGTAI

FTKEKPISVKMGLGIEEHDNEGRVITLEYDKFFLVNVYTPNSQQKLARLEYRMSWEDVFRNYLKDLERNK

PVILCGDLNVAHKEIDLKNPSSNRKNAGFSDEERSKMSELLNSGFTDTFRYFYPDIEGVYSWWSYRFNAR

ANNAGWRIDYFIVSQSLNDKLEDAKIHTSIEGSDHCPVELEINL

>WP_034886660.1 exodeoxyribonuclease III [Erysipelothrix rhusiopathiae]

MKLISWNVNGLRAVMKKDFEGIFEAMDTDVLCLQETKMQAGQLDYDPEGYYAYYNYAEKKGYSGTAVYTR

IKPLNVTYGIQEDEHNTEGRVITCEYDDFFLVCVYTPNSQPELKRIDYRMQWEDDFREYLKMLDETKPVV

LCGDLNVAHKEIDLKNPSANRKNPGFSDQEREQFTNLLDAGFIDSFRELHPNAIDRYSWWSYRANARSRN

TGWRIDYFVVSERLRNAIEAADILDQVLGSDHCPVMIKLNF

>WP_006193130.1 exodeoxyribonuclease III [Selenomonas sputigena]

MRFVSWNVNGLRACLGKGFMQSFKALDADVFGVQETKMQPEQAILELTGYKQYWNSAEKKGYSGTAVFSR

IEPLSVSYGLGIEEHDHEGRVIALEFDDLYFVTVYTPNSQRGLERLAYRMTWEEAFRDYLLALDAKKPVV

VCGDLNVAHTEIDLKNPKTNRKNAGFTDEEREKMTELLAAGFTDTFRALYPDKTGIYTWWSYLRKARETN

AGWRIDYFLVSDRLAPKIKEATIHNEVFGSDHCPVGLVLG

>WP_072972941.1 exodeoxyribonuclease III [Tissierella praeacuta]

MKLISWNVNGLRACVGKGFLDFFKEIDADIFCVQETKLQEGQIDLNLEGYYQYWNYAEKKGYSGTAVFTK

VEPIDVSYGIDIDEHDNEGRVITLEYDEFYLVNVYTPNSQRELARLDYRMKWEDAFKSYLKGLETGKPVI

LCGDLNVAHKEIDLKNPSTNRKNAGFTDEEREKMTALLESGFIDTFRHFYPDKEDAYTWWSYMRQARDRN

VGWRIDYFIVSEILADRIKSAAIHSDVMGSDHCPVELII

>WP_095545094.1 exodeoxyribonuclease III [Candidatus Izimaplasma sp. ZiA1]

MKFISWNVNGLRAVMKKDFENIFKELNADFFCIQETKMQEEQKTFSFDGYYEYWNDAIKKGYSGTLIYTK

HRPLNVFLGLEDDSYNDEGRIITLEYENFYLVNTYVPNSKRELLRLDYRMEYEDKVREYYGKLSTKKPVI

LCGDLNVAHEEIDLKNPKSNRKNPGFTDEEREKMTTLISSGFIDTFRHIYPEKIKYSWWSYMFNARANNA

GWRIDYFLVSKKLKNNIINVDILNNIMGSDHCPVSLELEV

>WP_022855452.1 MULTISPECIES: exodeoxyribonuclease III [Thermodesulfobacterium]

MVVATWNVNSIKVRKEQVLEFLKEVGPDLLALQETKVKTEDFPFKPYQEIGYHVVHSGGKGRNGVALLSK

VAPKIIKRGFENTSEPESFPDAEERLIGIEVEGFGFASLWVFSVYIPNGGQPESDYYYYKLSFFWQLKEF

FEKTFSPEDPIILMGDFNVAPEDKDVFAPGLLEGHICFTEKERRAFFDLLSFGFYDTLREKYPDARGVFT

WWDYQFGAFKKNQGMRLDHILITRPLLEKLGDVYVEKSFRTKPKPSDHAPLIAKFLV

>WP_014295982.1 MULTISPECIES: exodeoxyribonuclease III [Marinitoga]

MKILSWNVNGIRAAIKKGFLDFLDKENPDILCVQETKAREEQLTKKFLTYGDWKKYFVSAEKKGYSGVAT

FTKIKPKNVLKGLGNEMFDSEGRTLITEYDNFSLFNIYFPNGKAREERLQYKMDFYYYLLEFLEDYKKKQ

PNIIICGDVNTAHKEIDLARPKENENTSGFLPIEREWIDKLLESGFVDTFRMFNKEPGNYTWWDYKTRAR

ERNVGWRIDYFFVSTSLKEKVKDAFILSEIMGSDHCPIGIEIDI

>NP_001632.2 DNA-(apurinic or apyrimidinic site) lyase [Homo sapiens]

MPKRGKKGAVAEDGDELRTEPEAKKSKTAAKKNDKEAAGEGPALYEDPPDQKTSPSGKPATLKICSWNVD

GLRAWIKKKGLDWVKEEAPDILCLQETKCSENKLPAELQELPGLSHQYWSAPSDKEGYSGVGLLSRQCPL

KVSYGIGDEEHDQEGRVIVAEFDSFVLVTAYVPNAGRGLVRLEYRQRWDEAFRKFLKGLASRKPLVLCGD

LNVAHEEIDLRNPKGNKKNAGFTPQERQGFGELLQAVPLADSFRHLYPNTPYAYTFWTYMMNARSKNVGW

RLDYFLLSHSLLPALCDSKIRSKALGSDHCPITLYLAL

>NP_001171688.1 DNA-(apurinic or apyrimidinic site) lyase [Gallus gallus]

MPKRSKKGEDGEAEVVSAKSPREAAAPYVDPPVREETADGRPYNFKVTSWNVDGLRAWVRKGGLQWLQSE

APDVVCLQETKCGAESIPSELSQLPHLPHKFWGSAVGRSGYSGVGLLSRTAPIRVTHGIGIEEHDAEGRV

LTAEFPSVYVVSAYVPNSGRGLNRLQYRQRWDGAFKSFLQRLDAQKPVVLCGDLNVAHREIDLRNPKSNR

RSPGFTQEERDAFGALLDGGFLDSFRLLYPDVPNAYTFWTYMGGARERNVGWRLDYFLLSTRLREALCDS

KIRSAAMGSDHCPITLYLAL

>XP_008113582.1 PREDICTED: DNA-(apurinic or apyrimidinic site) lyase [Anolis carolinensis]

MPKRGKKKEEDGELPVEKEVAEPEPKKAKKGGAKAAKEADGPILYEDPPDKLTSSNGKKYTLKVTSWNVD

GIRAWVKKKGVEWVSEENPDILCLQETKCAEKQLPADIRDLAEYPHKYWACSEDKEGYSGVALLSKVKPL

EVKYGIGEEEHDKEGRVITAEFPSYFLVTSYVPNAGRGLVRLEYRQSWDVAFRSYLKGLAARKPLILCGD

LNVAHEEIDLKNPKGNKKNAGFTPEERAGFTKLLEEGFVDTFRHLYPDTAYAYTFWTYMMNARSKNVGWR

LDYFVVSKDLQESICDSKIRSTALGSDHCPITLYIAV

>NP_001085229.1 APEX nuclease (multifunctional DNA repair enzyme) 1 L homeolog [Xenopus laevis]

MPKRGKKEEVCAAEPQEDGNEPEVKKGKKGAGKAAKEPEPVVLYEDAPDNVTSADGKKYTLKISSWNVDG

IRAWIKKQGLNWVREEDPHIMCLQEIKCAEKLLPPDVKDMPEYPHKYWACPDEKEGYSGVAMLCKDKPLN

VTYGIGIEEHDKEGRVITAEFDSFFVIAAYIPNSSRGLVRLDYRQRWDVDFRAYLKGLDSKKPLILCGDL

NVAHQEIDLKNPKTNKKTPGFTPQERQGFGELLAEGYLDSFRELYPDKPSAYTFWTYMMNARAKNVGWRL

DYFVLSKALRPALCDCKIRSKVMGSDHCPITLLMAI

>XP_006009923.1 PREDICTED: DNA-(apurinic or apyrimidinic site) lyase [Latimeria chalumnae]

MPKRGKKEKVGNGEEVQDAVAEPKAKKTKKESGSEAKGTGMYEDPPDKLTSTDGKTANWKITSWNVDGLR

AWIKKNGLDWVREENPDVLCLQETKCAEKALPDDIKNMPEYPHKYWACSDDKEGYSGVAMLCKNKPLNIT

YGIGIEEHDKEGRVITAEFDRFFLVTAYIPNAGRGLVRLEYRQQWDVDFRAYLKSLDARKPLVLCGDLNV

AHQEIDLKNPKGNKKNAGFTKEERDGFTTLLAEGFIDSFRHLYPDAPYAYTFWTYMMNCRAKNVGWRLDY

FVLSEPLVANLCDSKIRSKAMGSDHCAITLYMAI

>NP_998586.2 DNA-(apurinic or apyrimidinic site) lyase isoform 3 [Danio rerio]

MPKRAKKNEEGVDGESDNGTAAAKKEKKGKEPEAPILYEDPPEKLTSKDGRAANMKITSWNVDGLRAWVK

KNGLDWVRKEDPDILCLQETKCAEKALPADITGMPEYPHKYWAGSEDKEGYSGVAMLCKTEPLNVTYGIG

KEEHDKEGRVITAEFPDFFLVTAYVPNASRGLVRLDYRKTWDVDFRAYLCGLDARKPLVLCGDLNVAHQE

IDLKNPKGNRKNAGFTPEEREGFTQLLEAGFTDSFRELYPDQAYAYTFWTYMMNARSKNVGWRLDYFVLS

SALLPGLCDSKIRNTAMGSDHCPITLFLAV

>XP_020381924.1 DNA-(apurinic or apyrimidinic site) lyase [Rhincodon typus]

MWLSRLNQGAAGGKGAVGTAKDDGADTDGAFEDTPDRLETEDGKHSDLKITSWNVDGLRAWVRKNGVQWV

TSESPDIMCLQETKCSEKQVPAEVKELPEFPHQYWSSPQDKEGYSGVGMLCKVKPLNVTFGIGVEQHDNE

GRVITAEFDKYFLVTAYVPNSGRGLVRLDYRQTWDKDFSSYLKGLDSKKPVILCGDLNVAHQEIDLRNPK

TNKKTAGFTAQERDGFGQLLSQGFLDTYRHLRPDARHAYTFWTYLGNCRARNVGWRLDYFVMSQSLLPSL

CDSKIRSGALGSDHCPITLLMAM

>XP_019646973.1 PREDICTED: exodeoxyribonuclease-like isoform X1 [Branchiostoma belcheri]

MIHITTWGLLHALAAGDAEPAKKAKAEAGGTSSQSDFSSNAKTKSGKHWNLKIASWNVDGLRGTVKKNGH

AYITQEDPDIICLQETKCTDKDVPKEFTSTVEGYHGYWYSPTEKKGYAGTALLSKTKPHKVTYGIGVEEH

DDEGRVITAEFDNFYMVTAYVPNAGKKLVRLDYRQTWDEAFTDYLKKLDKKKPVVLCGDLNVAHNEIDLK

NPKTNRNKTPGFTDQEREGFTSLLDQGFKDSYRELYPEETDCYTFWTYMGGARAKNVGWRLDYFVLSDRL

MPHLCDNVIRSGVMGSDHCPIVLLLAMGK

>CBY09188.1 unnamed protein product [Oikopleura dioica]

MAKQAEEVAPRRSSRQREKRKISDEALAAEEAKPKQAKKAATKKQPKKEKNIVAEAEPPKESKNEEDSSS

NFGSETQEKPESQEYSASEGLPVPDLPISDLYPKSSQEQLDSDEEVEPKTATAKVEQAGMFRIVTWNVAG

LYACIKKDFCKAVKTLDCDILCLQETKLSLKKPPPAEIAEQLKEWKYRNYANSEGKAGYSGTAILSKSKP

ISVQRGIGKTKHDDFGRSCTAEFEKFFLVTSYVPNSGRGLVNLDYRTNEWESDLRNYLTKLNKDKPVIYC

GDLNVAHTAIDLKNDKSNYNKSAGYTQAEIDELEKLLELGYVDAYRKLYPAEEDCYTFWSYMGGARSKNV

GWRLDYFLMKIKDCEEWIEDVVVHSKVQGSDHCPVEIKLNLP

>XP_002123983.3 PREDICTED: DNA-(apurinic or apyrimidinic site) lyase-like [Ciona intestinalis]

MIKVFQVKIKSFHFNFVIKRFCVMAPTRASLKREMAEAENVVEKKKAKEENGNGEKATEKREEENKVVEK

KLETKDGRQFNLKISSWNVAGVRAWVKKDGVKWVKGESPDIFTLQETKCSEKDIPQELKDLKEYHMSWNV

AKSTKGFSGVGLFSKVKPLEVKFGIGVEEHDQEGRTITAEYDKFYLVSTYVPNAQRGLKRLEYRLKWNSD

FLAYIKSLDEKKPVVLCGDMNVSHHEIDLANPKGNKKNAGFSQEERDGMTELLQSGFTDTYRDLYPDLTG

QYTFWTYMGNCRAKNVGWRLDYFILSNKWKDNVCDNIIETSAMGSDHCPITLLLAVP

>XP_022081710.1 uncharacterized protein LOC110974410 isoform X1 [Acanthaster planci]

MIFVRRMSRLLVCNKEIDLLQSSFLRWRKTNDRGRCNSHVGQRKQLSWSKLQLVSVKTSTWLENRQFKTE

SIRQGSADSSKRHGTGLCGKQIKSKNTMPKRKSDTKSEQSSKKAEPKAETTDEKPTKKQKVDKNGANEAA

IAALNVPETYTTDKKSVEENEANLKIASWNIAGLQAWIKKDGMSYIKKEDPDIFFVMETKVDSESVPSSA

KVEGYHTYWLGATSKKGYSGTGLYTKKEPISVKYGIGVEKHDEEGRVITAEYDDFYFVGAYVPNSGRGLT

RLEYRQEWDKDFTEYLAKLDETKPVIYCGDLNVAHEEIDLKNPKNNHNKTPGFTDEEREGFSALLNKGFV

DSFRHLYPDREYAYSFWTYMGNCRAKNVGWRLDYFVISKRLVPKLCDTGMRTWIKGSDHCPIVLHMAM

>XP_789515.3 PREDICTED: DNA-(apurinic or apyrimidinic site) lyase isoform X2 [Strongylocentrotus purpuratus]

MWMLRIKATYFHLFHKIVNKTSLFAPCEFLGIRNIHYTAAMPKRQKNEEASNGEEVVAEKKAKPEVAEEP

KEEVYTTDRKSESGEACNLKISAWNVGGMKAWIKKGGIDYLTKESPDIFFAQETKIDATKPPPEADLDDY

HITYNAAEKKGYSGVALFSKKEPLSVTKGMGIEEHDKEGRLITAEYDSFYFVGVYVPNSSRKLVRLDYRQ

EWDKDFHAYLKKLDAKKPVICCGDMNVAHEEIDLKNPKSNRNKTPGFTDQEREGFTSLLDMGFVDSFRHL

YPEEADAYSFWTYMGNCRAKNVGWRLDYGVISKALVPKLCDNQMRLQTFGSDHCPMVVSLAM

>XP_002731016.1 PREDICTED: DNA-(apurinic or apyrimidinic site) lyase-like [Saccoglossus kowalevskii]

MQLNRGIQTLQTLRLLNHLYREAIHRLVAAINMPPKRKSNDSDAMKTKKTKVDNKEAKGAGSEKPTLDSL

DFSLNAKTTDGKEWNIKLVSWNVNGVRAWCKNEGHKYVSKEDPDIFTIQETKCQEGEVPDDVKFDGYHSY

WSYAEEKGYAGTGLYSKVEPISVTYGIGINKHDKEGRCITAEYEKFYFISTYIPNAGKGLKRLSYRQEWD

KDFREYMKKLDEKKPIIWCGDLNVAHQEIDLTNPKTNKKTAGFSKEEREGFTKHLEIGLVDSYRHLNPEK

TGEWSFWTYMMNARGKNIGWRLDYFVLSKRLLPCLCDSIIRSQVYGSDHAPIVLFMAF

>NP_476841.1 recombination repair protein 1, isoform A [Drosophila melanogaster]

MPRVKAVKKQAEALASEPTDPTPNANGNGVDENADSAAEELKVPAKGKPRARKATKTAVSAENSEEVEPQ

KAPTAAARGKKKQPKDTDENGQMEVVAKPKGRAKKATAEAEPEPKVDLPAGKATKPRAKKEPTPAPDEVT

SSPPKGRAKAEKPTNAQAKGRKRKELPAEANGGAEEAAEPPKQRARKEAVPTLKEQAEPGTISKEKVQKA

ETAAKRARGTKRLADSEIAAALDEPEVDEVPPKAASKRAKKGKMVEPSPETVGDFQSVQEEVESPPKTAA

APKKRAKKTTNGETAVELEPKTKAKPTKQRAKKEGKEPAPGKKQKKSADKENGVVEEEAKPSTETKPAKG

RKKAPVKAEDVEDIEEAAEESKPARGRKKAAAKAEEPDVDEESGSKTTKKAKKAETKTTVTLDKDAFALP

ADKEFNLKICSWNVAGLRAWLKKDGLQLIDLEEPDIFCLQETKCANDQLPEEVTRLPGYHPYWLCMPGGY

AGVAIYSKIMPIHVEYGIGNEEFDDVGRMITAEYEKFYLINVYVPNSGRKLVNLEPRMRWEKLFQAYVKK

LDALKPVVICGDMNVSHMPIDLENPKNNTKNAGFTQEERDKMTELLGLGFVDTFRHLYPDRKGAYTFWTY

MANARARNVGWRLDYCLVSERFVPKVVEHEIRSQCLGSDHCPITIFFNI

>XP_021956670.1 recombination repair protein 1-like [Folsomia candida]

MGRPKRSAAPAADVDTEEMEVDKAAEKARIGREKAKKSYREDSASEDEAVAAAAVIDPKDKDFSDEDVLS

PSESEGGSDWAGDSSGSEYGKKSKKTVAKKGRGSVGRGRGRKAASPAKGKGRGRGRPPAASSASKNGKKA

TPAKRGRGGDSDEESESDAETSGSDYGKKKKPAVSISPKKRGPAAKKSPAAKASPKRKRGAAASSSPSSP

PAPRRVGRASASKSYKEMSEDDDIEVSDDDKPVTKKAKLEPKAKPAAKKAEAKKKAASASEDEEDIEDDV

DESMEDEEEDEKKADKKVTTAKKNGSSTKKEVEKKDTTTEEEDVEEEEEDDDEGDKKKKDTKVDKKDNVA

AAAAKNRNNGHTSPDEEESGDEDDVKFKDDKSPASEENGASVEDDDEDKVDKPALKNKTTTDYSKIDFET

DKKTPKGDEWNYKIACWNIGGIKSWLGKNGLEYLVKEDPDILCLNEVRCGEKTKPEQVSKLDKYPHLYWS

FNSESPGHSGVAIFSKEEAKSVEYGLPENEADSSSDKKLREGFNKEGRLITVEFDQFYLINAYVPNSGRA

EKSDKYPKGYPPKVANGDRLKFDEFFRNYVKELEKKKSVIVTGDLNVAHAEIDLANPKTNDRTAGFTKEE

RDGMTKLLEETTLFDSFRELYPDAKGKYSFWSYMHNAREKNTGWRLDYFLLSEKLRDSLCDNVMRTEVYG

SDHCPIVLFMRN

>XP_018026508.1 PREDICTED: exodeoxyribonuclease-like isoform X1 [Hyalella azteca]

MTIGHMLNTFGKISIPIIKLGFAQKYSPLFAKVSTFQKAMAKKRGNDAAKSGNSSSEKGEPASKKLKSNS

VQEKTSSTEASETFHSEAVTNSGEKWNFKIVSWNINGLRAWLQNDGHSIIAKEDPDVICLQETKCSKDKI

PEAALNVPGYKSYFMSAEKEGYAGVAVYSKVAPLSIAYGVSDGEHDKEGRAITLKFEDFYLVTAYVPNSG

RGLVTLDKRLNWDAIFLKYLQELDKEKPVIMCGDLNVSHHEIDLANPKTNRKNAGFTQEERDGFTKLLEA

GFVDSFRHFYPKKENAYTFWTYMRNARAKNVGWRLDYFVVSERFKESLCDSLIRSWVMGSDHCPITLLIH

TKMARSS

>ADD38834.1 DNA-apurinic or apyrimidinic site lyase [Lepeophtheirus salmonis]

MGSGSPPRKRPKRSKATKETKKEEVKEKVEDVLDVKKETESPSDERPDCTQTQKGKKSCNLKITSWNVAG

VRAWIKKGSLSYLLETEKPDIFCLQETKCSETKIPCELKELKDYPHQFWAFAKKEGYSGVALFSKVKPLD

VQVGLQSQDHDEEGRIIIAEFEFFFLLTTYVPNAGRKLVTLDKRLDWDLLLRKKMKELNKKKPVIASGDL

NVAHTEIDLANPKTNQKNAGFTPEERAGFDDLLSLGFVDTYRKLNPEKTGAYTFWTYMMNARAKNVGWRL

DYFLISDRWFESNVCDSVIRSKVLGSDHCPISLLLHV

>KZS01701.1 putative Recombination repair protein 1, partial [Daphnia magna]

CSSRMPPKKKIKVDSSRNPSDDGNLESDCEIRNPIEQKSKTADEKKPKRKKNSSTDAENIKVTQVFQNKT

STEFHEQDFGNTSKTPDGKKWNTKVTWNVDGLRAWIKKGGLDYLEHENPDILCLQETKCSKSKLPQEVVV

PGYHTYWCFSEADGHAGLGLYTKVKPDTVSYGIGIPEFDKEGRLIIAEYNTFYVVNVYVPNSGRKLVTLD

KRLKWNPKFQELIKDLDSKKPVIICGDMNVAHQEIDIANPKSNKRNAGFTQEERDGFSELLKSAALTDCF

RQKHPDVKGAYTFWTYMANARKKNIGWRLDYCLLSKKLMANFCDCCI

>XP_013794330.1 DNA-(apurinic or apyrimidinic site) lyase-like [Limulus polyphemus]

MARKGSTARKAKSKVTHVEKTQSSEQNTTSNTRKRKRNESSDVEVSESKYNKSDKSLKEEATAAESSSAT

PAEKNAFYRISSWNVNGVRAWLDKKGLDFIKQENPDIFCIQETKCSDDKLPANVKNVTGYKTYPLSGDKD

GYSGVCMFSKKQPVSVKYGIGIDKHDKEGRVITAEYDEFFLVAAYVPNAGKKLVRLDYRQDWDKDFCKYL

KTLDAVKPVILCGDLNVAHQEIDLANPKTNKKNAGFTQEERDGFSSLLNEGFIDSFRTLYPNAKGAYTFW

TYMMNARAKNVGWRLDYFVLSSRLRSKMCDSIIHKDILGSDHCPITLTIAF

>XP_003744372.1 PREDICTED: DNA-(apurinic or apyrimidinic site) lyase [Galendromus occidentalis]

MAPKRAARRAASKTDEPSETVQDSTAEISKKTRSKRSRKDHEEDDLVEEKLSGGKAKKTKKTPKRSKSDN

ADVDDSSSTDKTRIASSDYGRKGSWKIVTWNVNGIRSWLKNGGLEYIEEEDADVYCLQETKCSEEKLPPE

VTNYKGYKSYFLAGDKEGYSGVGIMSRKTPLNVEYGLSLEEHDSEGRVITLEFEEFFLVNSYVPNAGRGL

VRLDYRLTWDRDFRKYLVGLKKKKSVILTGDLNVAHNEIDLKNPKTNKKNAGFTEEERQGLTDLLEEGFV

DTFRKLYPEREGAYTFWTYMMNARAKNVGWRLDYFIVSEDLIDNVIDNEIRSTVMGSDHCPVVLHLK

>OWA52855.1 Recombination repair protein 1 [Hypsibius dujardini]

MEVAQTETKEDLIAPSSPVSKAGISASPTMTDTEAASAEKPKMPSPTAALVEASKKRSNKRKSILPDNLH

ALRASFAKTEDISVSPSPDGSVLGTPDRSTSTETVIPVVTVQPPAPSRDHVELNVQEPIAKRLRIRGTEK

PAYVKPTRTARKARSESVDKATSLPPADSPVSDFDFTGLGVRSDVAAHKLPATAGVVDEVAPLKGILKHG

NAPSAKQLAFIDEGVEERQALAESRRVEMEAVPAAAAPNPNKGVELLPGRAPNLLILSWNIAGLKAFVEK

HTWREINTLLPDIICLQETKIQNNDVFESLGISVGTSHAYWHPSLTKGHAGVAILSKIKPIAVTYGLTRP

DGRTFAEKGHAITAEYQNFFLINAYVPNSSAQLKNLTERLEWDKDFRAYIAQLQTKKPVVIGGDLNVALE

EIDLSNPKRSTKLPGFTPQERESLALTIKDEKLVDVFRHLNPDKPGCYTFWSVRTNSRPKNVGWRLDYFL

VSESLMPKVCGMWHFVDVTGSDHCPIMLYLET

>NP_001021584.1 DNA-(apurinic or apyrimidinic site) lyase [Caenorhabditis elegans]

MSKRKAEEAPAPKLASIFTKKVKPAEEDNNQKSWKFVCWNVAGLRACVKKSDFKEVLAEEPDLVFLGETK

CKEWPPEMEETFKNYTKTLVVSTEKNGGYAGVGLLSKCAPMKVHKGIGDPEFDTAGRLIIAEFSKFYFIG

AYVPNSGAKLVNLEKRGRWEKLLTEKMKEMDEKKPVIYGGDLNVAHNEIDLKNPESNRNKTAGFTDQERG

WFSEMLELGFTDTFRAMHPDEKKYSFWSYLANSRQKDVGWRLDYYVVSNRIMNKVKRSDIMSSVMGSDHA

PVVMQIDF

>XP_014672642.1 PREDICTED: DNA-(apurinic or apyrimidinic site) lyase-like [Priapulus caudatus]

MEEPKKRGRAGKVKVADEEPTTKKAKKPDENAAANGAKTAPAGGATDDFSSEATCEDGRRWNFKVASWNV

NGLRAWIEKGGLDYLRKENPDVFFLQETKCSKDKLPAGGERCGRLHGALAGGRQGRLLRCRDVHEDCTLS

IKHGIGMSEHDKEGRAITAEYDKFFVVGVYVPNSQKKLARLDYRQKWDKDFRDYLKKLENSKPVILCGDL

NVCHEEIDLARPANNHRNAGFSDEEREGFTQLLNAGFIDTYRSLHPAQAGAYTFWTYMMNARSKDIGWRL

DYFVISESLLPDLCDSVIRKHTMGSDHCPIALLLAV

>XP_005101256.1 PREDICTED: DNA-(apurinic or apyrimidinic site) lyase-like [Aplysia californica]

MARKRAAKKSKEETNEAAASSATEDTQKKEADEKDTTTEKKPTPAKGRKRKSDAIKDDSGADAEPKPKLK

ARVSISDSLSGTDFKCTAKSPSGKESNFKIASWNINGVRAWLDKEGLSYLNAEQPDVLCVQELKCDVSKI

PAAAEVDGYSTHWLSGDTEGYSGVGMYFKKKPIKITDGIGISKHDKEGRVITAEFEKFFLVNTYIPNSGR

GLVRLKYRSEEWDKDFRNYIKSLDAKKPVVWCGDLNVSHQEIDIKNAKGNKKNAGFTQEERDGFTEMLNE

GFIDSFRHLYPEEEGAYTFWTYFMNARAKNVGWRLDYFVLSERFKEQMCDSVIRSKVLGSDHCPIVLHLA

L

>XP_014780504.1 PREDICTED: exodeoxyribonuclease-like [Octopus bimaculoides]

MLKVTLSPDRQSADNAKAEVKKQKTDAEKQEGSSDKDGQTHNLKVISWNINGIRAFLGKNGFDYFKREDP

DIFCLQETKCPESQVPEDCKIKGYYNYWYSAETDGYAGCCLYSKKKPISVTKGIGIEKHDKEGRVITAEF

EKFYVVTTYIPNAGKKLVRLDYRIKEWDVDFLDYLVKLDGKKPVILCGDLNVSHLEIDLKNPKNNKKTAG

FTQEERDGFSKMLDKGFVDSFRHLYPKKTDVFTFWTYMMNARAKNVGWRLDYFVISKRLVDNLTDTLIHK

DEMGSDHCPIVLLMNM

>XP_011420949.1 PREDICTED: uncharacterized protein LOC105323590 isoform X1 [Crassostrea gigas]

MLNILPWHQILLSSFVRGASRISHRVLSSSDKMPPKRKAKKDDGKTPAPEEGSVGDTAQEDKPSPSKKQK

VDEEEKGKKGKGGKGKKAPPRKEDTIETLPEGTDTSFPTEVNPEAKTTDGRKFTLKIASWNLNGIRAWYD

KDKMSYIKESSPDILCVQETKCQEDQLPPGVLDKDYHIYWSPAEKAGYAGTGLYSKKKPIKVTYGLGIPK

HDDEGRVITAEYEDFYMVNAYVPNSGKGLVRLKYRTEQWDCDFTDYLKKLDAKKPVIMCGDLNVSHTPVD

LKNPTSNRNKTPGYTDKEREGFSKLLEEGFLDSFRVLYPNARDCWSFWTYMMNARAKNIGWRLDYFVISK

RLQKDLCDSIIQQKVMGSDHCPIMLLMAGLK

>ELU05263.1 hypothetical protein CAPTEDRAFT_159745 [Capitella teleta]

MPPKRKSDASSGDGSGAKKKKLARTPSKRESIPDVSGNDYSSEAATKDGKKWNLKFSSWNVNGIRAWVEK

NGHSYVTAEDPDIFCVQETKCAKDLIPDDANIEGYHAYWLSGDKDGYSGTGLYSKQEPLSVTYGIDKEEH

DKEGRVITAEFDKFYFVTAYVPNAGRGLPRLSYRSEKWDPDFREYLKNLDAKKPVVMCGDLNVAHKEIDI

ANPKSNKKSAGFTPQERQGFSELLEAGFVDAFRELYPEETKKYSYWTYMGNARGKNVGWRLDYFVVSEKI

KDGICDSLIRSEVMGSDHCPVVLLMNI

>XP_009026759.1 hypothetical protein HELRODRAFT_186002 [Helobdella robusta]

MAPKRKKLFESTPAVKKSKTAITSRAAIPSVADLNFSSDSKTENGDMWNFKVASWNVNGIRAWAEKNGHS

YIPAENPDIICLQETKCDEKNIPENVKINGYNMFWNSADKGGYSGTAIYSKVEPIKVTKGLGIKKHDNEG

RVITAEFEKFYFVTTYVPNSGQGLVRLDYRVSEWDVDFHDYLVGLEREKPVILCGDLNVSYLDIDIANPK

SNQKSAGFTKEERESFGKLLNKSFVDSFRHLYPTKTGMYSYWSYMGNARSKNVGWRLDYCMVSSSLKPAI

CDSVMRKDVMGSDHCPVVLFLHL

>XP_013416766.1 PREDICTED: DNA-(apurinic or apyrimidinic site) lyase-like isoform X1 [Lingula anatina]

MMSAISTKLAKFRLGSVSAWWEHLMHNYQRGNQHFMNGSHIELLLASRNKQTHEKKICLVNKEGDSSEEP

KKKSKKEEPEASAPSTDWTASSDAKSKDGRPWNMKIVSWNINGIRAWIKKNGHEYVRKENPDIICFQEIK

CAEDKLPPECSIPDYYPYWLTADKEGYAGTGILSKTKPLSIKYGLGIEEHDNEGRAITAEYEKFYLVTSY

VPNSGRGLVRLNYRTKEWDEAFREYLLGLEEKKPVILCGDLNVAHKAIDLANPKSNYNKTPGYTQAEIDG

LSKLLDKGFVDSFRHLYPDVTGAYSFWTYMGNARSKNVGWRLDYFLISKKLLPSLCDSLIRKEVMGSDHC

PIALLLAV

>XP_012792061.1 DNA-(apurinic or apyrimidinic site) lyase [Schistosoma haematobium]

MILKSRIYTDKIHVENTQNSDWCTRYPGSSPDLANPLSPKWNFKIVSWNVNGIRAVIKNNGMEYIKKENA

DIFCIQETKCPLHKIPSEAKVPSYQSFWSSADKAGYAGTALFSKISPIKVTYGIGKKLHDEEGRVITAEY

DKFYLVTAYVPNSGQGLVRLPYREKEWDPDFLEYLRKLDSTKPVIVCGDLNVAHEEIDLARPETNHKTAG

FTDQERSGFTKLLSSANLIDTYRHFYPDRRGVYSFWSYRTGARLINNGWRLDYFLVSERILTNVSDQEIR

CGVTGSDHCPVVLYLQI

>PAA54475.1 hypothetical protein BOX15_Mlig011745g3, partial [Macrostomum lignano]

SQRMPPKKRQSTSKPKAAGSKRAKLDEAASESSAGKEKKPAKAAAKKESDAPAAAAAPSTAAAPSTSATS

LECPAAKDAEGRPWSLKLASWNINGLRAWLKNNGTRYLLEEAPFIIGVQEIKCPSAKVPPEASIPGYHAL

FSSADKDGYAGTGIYSRDKPLSVKEGIGAAKHDSEGRVITAEFEKFYFVTAYVPNSGRQLARLKYRVSEW

DKDFAAYLRGLDAVKPVIVCGDLNVAHLEIDLANPKTNQRTAGFTKEERDSFSSMLSSLDLVDTFRQVHP

DQTKAYTFWSYMRNARAQNIGWRLDYFLVSKRLAGRVCDSLIRSSVMGSDHCPIVLLLSL

>CDS21880.1 DNA apurinic or apyrimidinic site lyase [Echinococcus granulosus]

MARQSQLKFATSKKDQPAKLAKPAKKSQNTARTRKLAKVVKEEVTTTKAGSVASASDGKVVSSSMRVPPF

EWCQTYPGSVCSLSNFSPKTDGYWQMKIASWNVNGLRAWIKNGGLDYLQSEAPDVLCIQEIKCAKKDIPP

LANIGEYTPHWYSANKPGYSGTGLYTKIKPLNVVYGLGISKHDTEGRIITAEYEHFYLINAYVPNSGQGL

VRLDYRTKEWNKDLCKYIKKLDKEKPVILTGDLNVSHEEIDLANPAGNHRSAGFTDEERQGFSEMLADCD

LVDTYRHFYPTRARAYTYWSARHNARKSNSGWRLDYFVVSRRMISSVVDQEIRCGILGSDHCPLVLYLNF

K

>XP_001625612.1 predicted protein, partial [Nematostella vectensis]

RKSNFKITSWNVNGLRAWLKNNSKSFVSKEDPDVFCIQETKCALADIPKEAKLAGYHCYWNSAEQKGYSG

VGLCSKKEPIKVSYGMGNKEHDKEGRVITAEYEDFHLVTSYVPNSGRGLPRLGYRQQWNKDFLSYLKKLD

EIKPVILCGDLNVAHKDIDLANPKTNTRTAGFTKEERADFTTLLGEGFKDTFRELYPDKKSAYTFWSYMG

GARAKNVGWRLDYFVVSDRLVPKVCDSIIRSRVMGSDHCPLSLLLSI

>XP_012561638.1 PREDICTED: DNA-(apurinic or apyrimidinic site) lyase-like [Hydra vulgaris]

MGKRSADSKVDKDAKKTKKNKIRQEEDEKKVTDLKDEKMETMPSDFESKTVTADGRTSTLKICSWNVAGL

RAWVNKNGLNYVIEEDPDVLCLQETKCTENDLPGQVLQMKNGPKVTNVSNSGYYMYWCNAKKKGCSGVSL

WCKLKPLKVTYGLGIKEHDSEGRLITAEFEKYYIITTYVPNSGQKLIRLDYRRIWNTDYKNYVGELKKKK

PVIMCGDFNVAHTEIDIANPNSNKRNAGFTEEERSDFTALLEDGYIDTFRKLNPEKTGAYTFWTYMMNAR

SKNKGWRLDYFIVTKDIEDDICDSVIRSSVMGSDHCPIVLNLAI

>KII63315.1 DNA-(apurinic or apyrimidinic site) lyase [Thelohanellus kitauei]

MSKRKSSEVDAPKVILRCQPTNVEKNYDMKIVTWNVAGFKAILKKGFDEYFKTEQPDVICLQETKCDEIM

PYSHKQATLSKSTRAYKYYWHTNSKPGYAGVSVWTKIPPIETTYGINNDEKNQGRAITLEFEKFYLVNVY

VPNSGVELVNLVYRQKWNRDFQNYIESLDKRKPVIVAGDLNVAHHEVDLANPDTNHRTAGFTDEERQDFT

LLLKSGFTDTYRHLYPQTFKSYTYWSYRRNCRANNTGWRIDYFVVSNRILENVVDCVPREDIVGSDHCPL

VLFIKSATI

>XP_019857082.1 PREDICTED: DNA-(apurinic or apyrimidinic site) lyase-like [Amphimedon queenslandica]

MPAKKKAKVEEEEEQEEIEEEEEEIDSPPDDDDDDEYDPNNDEEQELIMAGKGRKRKRGRQSSSEEDDDD

EYKPSRKPKVQARKQRKRSGDYDSPVKKRKPPPPPAKKTPGRGRKPKKVSLEEETGSPPPSPPNVSPPTA

TPILSKSVTDYSSIDLSSDAKNKDGSPWTHKFSSWNVNGVRAWLKASGIEYVNQECPDVFCIQETKCSEK

DLPKSELEVPGYHSYWNSAEQKGYSGTGLYSKEEPINVTYGIGIPKHDNEGRVITAEFEKFYFVTAYIPN

AGDKLKRLSYRVGEWDPDFKKYLKSLDENKPVVLCGDLNVAHKEIDIANPKTNQRSAGFTKEERESFSSL

LEDNKLIDSFRDLYPDKKEAYSYWSYRSNARKNNKGWRLDYFLVSERLKAAVCDSIIRSQVYGSDHCPIV

LLVAL

>OAF69763.1 hypothetical protein A3Q56_02480 [Intoshia linei]

MKRVKFSQQPKISTMLKNGKEKIKTSKRDFINIISWNINGASAMSKKKDVNYITESDADFYCFQETKSSK

EKLPSFFEMEGYFNYYNFGVKNGYSGTGLCSKEKPIKVTYGFETIKEEEGRLITAEMDSFYLVTAYVPNS

GRGLVRLDYRTKCWEPDMVKYIKNLQKLKPVIYCGDLNVAHQEIDLTNPNTNHKTAGFTDIERKKFTVML

AETEMIDTFRYLYPETKAKYSFWSYMRNSREKNIGWRLDYFLISEKLKKNLIDSLIHDEIKGSDHCPVEL

KLKIPIK

>XP_002117002.1 hypothetical protein TRIADDRAFT_60990 [Trichoplax adhaerens]

MPKRKASVTAVGKAKKQAKKAADSSKAKGGKSSAVDKEPTVEIKHDQSSVEKENQSHGKSSSNWNTKITS

WNVNGIKAWFKKNGHSYLTDSNIDIICLQETKCAESEIPNEIKLDGYHTYFYSAEQKGYSSTGLLSKVKP

LKVTFGITNIILHIKFRTDIEKHDKEGRVITAEYDKFFLVGAYLANPKTNTKTAGFTKEEREGFGKLLDC

GFIDTFRHFYPNKTSAYSFWSYMSNARSKNIGWRLDYFVTSKNLIPNVADSIIRSEIEGSDHCPIELLLQ

I

>XP_001745290.1 hypothetical protein, partial [Monosiga brevicollis MX1]

LHVVSWNVNGLRAVLKNHPKALPDLVQAENPDLLFLQETKLQASHVADYANLLPGYTGLWSCSRAKKGYS

GTAGATPTSDFRALSVQYGIKSAHHDQEGRTITVELPDLFVIGVYVPNSGQDLKRLDYRLNEWNVDFLAY

IRELEASKPVLVVGDLNVAHLDLDIYNAGHLVKSAGCTPQERTAFTEFLDQGFTDTFRKLYPEHTGAFTY

WSARTGGRQDSKGLRLDYAVCSNALLEANSPLRCLDSVMGSDHCPIAIVLAHQ

>XP_009542866.1 hypothetical protein HETIRDRAFT_100374 [Heterobasidion irregulare TC 32-1]

MPPKRAASSSKRKASNSDSEEEFEKTTKKPKAVVTGTESDRAPNGQPTNKVLPTAISFPSRTENSVRIAS

WNVCGLAASQKKGFKYYVEAEDPDILILTETKVNNEPVDPSLTEKFPHRYWSISEKKTYSGTAILSKHKP

LSVEKTLPGHPDPGSVKGRIITLEFENHYLVATYVVNAGEKLKTMDAKKTWNTHFEAYIRDLDKKKPVIW

GGDLNVAPTEIDLSNAKRNWNKTPGYTEAETTAFKNILNPPDDTKDANKFVDVWRKLHPDDHHYTYWSYR

FNCRSKGIGWRLDMFVLSERIVDRVKMCDIRSDIYGASDHCPLTIEIESPL

>CED84802.1 exodeoxyribonuclease iii [Xanthophyllomyces dendrorhous]

MSSLLTSFWISMSPGGLPEQKTTCSYPQPTNPPTFPSIQYINKALPASYDLPPKTGDVKISAWNITSLKS

ACDKKHGSGFRGYMDKEDADIMILTETKRNDSSPIPEDVFLKEKYPFQEWAFCSKKGYAGTAVLSKVKPI

TKSVDLVVKGERITGRIVQMEFPSLFLIGTYAPNASEGLKNMQGKKDWNVAFEAKLRELDAIKPVVWGGD

INVVASGNDISNAAKGWNKLPGWTQDELDGYMSQLNPSSESGHQPLVDAWREIHGPDEVQYSFHSKRSLQ

REKGLGWRLDTFVVSKRLQERVKACEIRREIFGPSDHVPVVIELSGSL

>XP_009268106.1 DNA-(apurinic or apyrimidinic site) lyase [Wallemia ichthyophaga EXF-994]

MPPKRKADGDDKKAPETKKSKAKEPVKPLDPTQPVNKQLPEELGAYEKGEGVTRMTCWNIAGYNASVKKG

MHRYINAEPADLLVLTETKIDKEPNDEILKSNYKHSVWTGSTKKGYAGIAVLWNGEEARNVETILPTHPN

QDSTRGRIITLEFEDKFVIGTYVPNAGQGLKAMDDKVAWNSAFKTYLHDLNEKKSVVWLGDLNVCHDSKD

IRNDESNWNKSAGWTETEVNGYKEQLSDKFVDAWKELHPDNVGQYTYFGYRFDARSKGIGWRIDSCVVSR

GLMGRVEDVVIRMEVYGASDHVPVCIDFKAVDEQASK

>XP_016276399.1 exodeoxyribonuclease III [Rhodotorula toruloides NP11]

MPPKRTSSKRAAVDETTAAKEDKEVQAKAEEDSPLSELSGSEKEAAPKKKRAKKPPVTPLDPSVPTNKEV

PEDLSSFPRPPEGCVRISAWNVAGLRASEKKGFSRYVNAEDADILVVTETKTPELSLPALDDRYEYRYWG

DHVKKGHAGTAIFSKIKPLNVSKGFQASEEVTAADSEGRMITLEFENSYVVGTYVPNAGNGLKTLPEKEK

WNRAFETYLRELDAKKPVIWCGDLNVVPTPTDIRNWKTNYNKSAGCTDAEINGFKAQLNPEEGSGHKRLV

DVWREKHPDLEGHYTYYSYKFQCREKGIGWRLDYQVVSERLLPKVKACEIRAEISSDHVPIVLDIEGPL

>XP_003333171.2 exodeoxyribonuclease III [Puccinia graminis f. sp. tritici CRL 75-36-700-3]

MAIKTMIWWRSAHPSLTNITSSTRRLSSAQSLCTRFLTTMAPSTTTKKKAATTKRKTQDQPDDPASSKPA

ASKKAKGPTVPLHAHLPNNRSFPTKLEFPQKKDDTLRISAWNVCGINACEKKGLKTYLAAEDPDVIILSE

TKMQAEPDIMHIKHQFRYRYWGGDETKGYAGVAILSKHKPIEVVYGLPTATDQSSTKGRIVTLEFSKFFL

IGTYTPNAGDNLKFMDRKKEWNAAFEKYLRELDAKKPVVWGGDINCALSEKDIRNAASNWNKTAGYTQEE

CDGLNSQLSPPQDSGHEKLVDVWRELNPDLEGHYTYFSYRFGCREKGIGWRIDAFIVSQRVFESVKTCEI

RQECYGASDHVPIVIEIALSP

>XP_014566365.1 hypothetical protein L969DRAFT_89779 [Mixia osmundae IAM 14324]

MSSTALPSLEAPGDKTPAASPAVKAKRKANDSASASEPVESSISKTKPKKSKKVEAEDELEDATSKPKKD

KKVKEPVRPLDPSLPTNKTIPAGLSFETPARQPDAVRLTAVNLSGMAASLKKGMWTYLEAEDADVVIMTE

TKIASEPAHEGIQAKYKHRYFSAGAEKGQAGTAVLSKIEPLSVAYDLPTLAEPGKLAGRLVRLEFPSLYV

LGTYTPNAGQGLKSMPRKQDWNEAFERYLRELDSVKPVVWMGDLNVAPTSIDVRNSKKNWDKEPGHTAIE

RDAFVQQLQPTESEHKPLIDVWRHRNPDVEQYTYYSYRFDCRTKGIGWRLDYAIVSERMLARVQACEIRQ

IAYGFSDHVPIMLDLQGPL

>XP_011392526.1 hypothetical protein UMAG_15027 [Ustilago maydis 521]

MAPTAPTRTSARRASSRTTTDTRAASPGASSIQPKRKAVHPVLSEQADATQIDSTSTDATSHAQTLTLAK

KPRKKSASDSDSASAPKSQDTTADPSLPKNTQMPATLSFARPRAPGSLRITAWNITSLKSSEPKGMLRYL

EAEDADIAVLSETKVNDVPMHPALTKIYKHQYWAIGKQKGYAGLAILSKIQPIKAIYGLPSLKDQDTKGR

IVTLEFENSFLVGTYAVNAGLAELDAQKPIIWCGDLNVVQDERDLAAASKKWNKSPGYTAIECDAHRQLL

QGTATPTSKPLVDVWRQQHPDAIGHYTFYGWRGFCRSKGIGWRLDSFILSERIAPKALECEIRHECYGAS

DHVPIYCDIQGPL

>XP_013243232.1 hypothetical protein K437DRAFT_278429 [Tilletiaria anomala UBC 951]

MLEYSLRPEGQVRIVSWNVSSFNSCEKKGLFKYLEVEQPDIVVLTETKVNEAPDMHPGLSAYPHRYWGID

AKTKGYAGVAVFSKIKPLKATIGMPLHPKEYNGRIITLEFSKHFLVGTYVTNAGEGLAKMSNKQQWNAAF

AEYIKELDAQKPVIWCGDLNVMLDQRDLSAPKKKWNKQPGYSKIECDAHRVLLEGKDRGEGRKLVDVWRV

SHPDAVGHFTFYSFRGACRGKGIGWRLDSFIISERALGSDKSRAFVQQCEIRHEVYGPSDHVPVICDVLG

PL

>XP_018740106.1 uncharacterized protein MSY001_1531 [Malassezia sympodialis ATCC 42132]

MVKRAASSTAAASSKKVRASESAPAAAAPSPAAPSGQDHTAPEWVPRNAQLPTGALKLAPRDEAHVRLMI

WNITSLKSSDTKGFMRYLRAEDPDVAVLSETKVNEKPSHAGIDTMYPYQYWGIGEKKGYAGIAVLSKIKP

AQVQYGLPGFDDPSSRGRLLTVEFARTVVVGTYAVNAGDNLKTLETKNRWNAALEKHLASLPRDKDIVWC

GDLNVVWDDRDLAGATKKWNKSAGYTQAECDAHRRVLATNDMCDAWRELHPDAIGHYTYYGWRGNCRARG

AGWRIDSFIVSRKALPRVQSCEIRHDIYGASDHVPVVADMTGPL

>KNE57242.1 exodeoxyribonuclease III (xth) [Allomyces macrogynus ATCC 38327]

MPPATRRQPKRKVAAPKPRAKPAKKEYGSDSDDEYLGSDTEQHQESEEEDENEYVLGNADPEPAPAKRPT

KKQKKTAKKTGGKNTEPAASEFTSRFPTNETLPQNLGEHWAPCPDGHIKVATWMVNGLRAALKKGADTYL

DTEQPAIACMQETKMHTKPTSDIFASLYLSYPHKYLAYPDEDVKGGYAGSAVLSTIKPLNVKFGLHGTDL

EDDQEGRTITLKFPGVFVVAAYVPNAGRGLQRLALKCDWDRALTQYVRELDRRKPVLVCGDMNVAHGDHD

VAQPTTNSKTAGFTQQEHDGFTSFLEATSMVDHNRAKGIGWRLDYIIASERLVPLLGECAIRDEVFGPSD

HVPVVVTAPRTLFECPGSSGEGAGAVAAAVEE

>XP_006682150.1 hypothetical protein BATDEDRAFT_20862 [Batrachochytrium dendrobatidis JAM81]

MVSTPSAAGKRSIDAVSSKQSDISSKKDKADESVNNASASLNKRPKRACSSNLSSSNIQSDTATSTNVAL

QETSDSSNPTVAITENPVDSSHQPCYTNTKLPDTIKFDPTPVGATKFIAWNVAGLRASLKKDMMRYIEAE

DADVVCLQETKMNASLDTSMFSRKQYPHQYWSHCTTKKGYSGVMVLSKVKPLSVEYKMGHSAIDDEGRFV

ILEYQDFYYIGCYIMNAGADLGRLDLKREHYVKLKKFMGELQSKKPVIWSGDLNVAHTEIDLARPATNTK

SAGFTPEEREDFGNLLTDLDMVDSFRLKHPTATGMYSYYSFRHNSRANNVGWRLDYFVVSKALQDDIIEA

DIRSEIYGASDHVPILLLLNRSLGSSVVEPITKEAKLFPLFIKK

>KXS19058.1 hypothetical protein M427DRAFT_95779 [Gonapodya prolifera JEL478]

MDDVTGTGDSVAQAHPELVDGADAAQVEVSAAISPAKKKTAKAPSSKKRAKKDQETSPADAKTAADVIDP

TAKDSTDVKDAEKPEKPDHNYPVNTAMPTSYDFLPPKAEKSVRIVSLNVAGIQAARKKGLDTYIRAESAD

ILCLQETKLQAQDDAVFADVYPWRYWSLSKTKKGYSGTCVLSKLPPLSTRLGIPTTTTTTPAPTEDEDDE

GRAVTLEFESFYLVANYVPNAGQKLERLAHKRDYAGWVREYLASLQTTKPVIWTGDLNVAHTAVDLTRPK

GNTRTAGFTVEEREDFSTTLTQLDLVDLWRHLHPTEQDYTYYSYRFNCRAKHLGWRLDYFVVSRALLDKV

RSCEIRNKVYGCSDHVPLALEIEV

>ORY45789.1 exodeoxyribonuclease III [Neocallimastix californiae]

MKIVSYNVGSLNASLKKGFKNYLEAENPDIICIQETKVNAPTHDISRKDYPYQYWTFSTVKKGYSGCAVF

SKIKPLSVEYGIGCAKFDDEGRNLVLEFENYYLIATYVPNAGNKLVRLDFKQEYSSTIEKFFKKLEEKKP

IIWAGDLNVAHQKIDLAKPDSNLRSAGYTIEERTDFTRILSSNPPRIDTFRHFHPKDQIYSYFSYRFNCH

VKNIGWRLDYFVVSEKLLPQIKDSLIRTKCYGASDHVPICLIMKKN

>XP_018289408.1 hypothetical protein PHYBLDRAFT_134873 [Phycomyces blakesleeanus NRRL 1555(-)]

MGVKRNSSTIDESATGSPKTKAQKTSTGPIDTSLPNNKVFPENFDFPEKPEGTIKISSYNVASLNASIKK

GFNKYVDAEDADILCLQETKVNSPVSTAVNDKVYKYRYWSYEDKKGYGGTAIFSKYKPLSVTYGLPGYED

KSRGRVITASFPSFVIIACYVLNAGDKLKSLDERRVFNSYMEKHIRALQKDNKSVIWCGDLNVAHTADDL

ARPKTNERSAGFTIEERTDFSKVLAPSSDNIPGLIDTWRHLHPDTKGHYTYYSYRFACRDKLLGWRLDYF

VITPDLLDKVVSCDIRHEAWGASDHVPLVLVLKDVKMTE

>ESA16581.1 hypothetical protein GLOINDRAFT_345481 [Rhizophagus irregularis DAOM 181602]

MSKKQNTLEKYLKPEVKEKNIHSNESAESSTRPTKKVKKDETNKTLKIPESLFFEKREGTVKLASWNVSG

LNAALKKGFKTYVEAEDADILCIQETKVNEKVNDVVDSKKYKYRWWGFDEKKGYAGIAVFSKIEPISTSF

GLPTHPNPESTKGRIITLEFESLYYVACYVPNAGDKLARLKEKIVWDEVMEKYLRQLDEKKPVIWAGDLN

VAHKTIDLAKPNTNQKTPGFTPEEREDFERILNGGKSKFIDTWRHFHPNTEGQYTYYSYRFKCYSKNIGW

RLDYHVVSERILDKVIESEIRSNVYGASDHVPIVLVLKDL

>KFH66332.1 hypothetical protein MVEG_08431 [Mortierella verticillata NRRL 6337]

MVTKRTAATRASARVSATDKDSKVEVSSTTATVETSTSKKRTTSDEEDDREESENDDNDDDDSYSGKKAT

KVVKRKIIKTEKTVSTKSSSTAPAAAPALAKKASGSKVPIRDDAPTNTTMPNPLVLPPTPKGCVKIASWN

VSGLNASLKKGFKTYINAENADLICIQEHKVNQPLLNIVDPKVYPFSWWGFEKEKKGYAGVAIFSKITPI

NVTAGLPTHPVPDVTKGRVVTLEFPTCFIVGCYVPNAGQGLVRLKERMLWDVAMKSWLKQLKDKGKQIIW

TGDLNVCHKAIDLRNPSTNTMSAGFTKEERAGFDEILQEVGMIDTFRKIHGEDAIGMYSYYSYRFQCRIK

GIGWRLDYFVTDEEMAKRVEQSVIREECYGASDHLPIVMVVKGSL

>ORX84008.1 hypothetical protein K493DRAFT_239487 [Basidiobolus meristosporus CBS 931.73]

MPGTYSYEELPENQIKLVSWNVNSLNAAMKKGFLEYVKAENPDILCLQETKLNQAPKDVLKKEYKYQYWP

CCQSGKKGYAGTAILSRIEPLQVTYGLDDSDLDDEGRVITLEFEAYFLVACYIPNAGEKLVRLKFREEWD

VKMSEMLDKLNAKKPVIWAGDLNVAHEEIDLARPAQNRNKTPGFTDAERAGFSKCLSLSSGAMVDTFRHL

HPDQSTYTYFSYRFQCRKKNLGWRLDYFVVSEALVPKVVHSAPRCECYGASDHVPIVLILEK

>KXN74098.1 hypothetical protein CONCODRAFT_76979 [Conidiobolus coronatus NRRL 28638]

MAIAKKTSSEESTDSSSKRIKLTKSKSSEVEYPTKHRNAKMPETYEFKGLDNDEQIKLVTYNVASLNASL

KKGFKEYVLAENPTIISIQETKLQQPMRELFDKDVFPHTYWSCSGPDKKGYSGTAVLSKIKPLSVKYGLG

TPNMDTEGRVITLEFEKFYLVSVYSPNAGDKLVRLGYKEKWDKIMLEHLNELESKKSVIYTGDLNVAHNP

IDVAKPKEYENKVAGFTKEERLGFSNILSDEKFKRVDVFRHFNPSETDCYTFFSWRGGCRAKGIGWRLDY

YVVSESLVEKVKDIQIRRDCYGASDHLPLVMWLDNMK

>OMJ21829.1 DNA-(apurinic or apyrimidinic site) lyase [Smittium culicis]

MTAPRSSARLLNKAASNLSETGNSKPASSTILNKNSKETLKIKSNKLDKVSKASKIQDVAIIQTEEVLED

GESLAKTKVTRIKRTRSESKAENTNIENEKDSVSTGKFFEVESKETTKIHVESSKKVKKSEMSQKDVENV

SIEKTNYPNNKVMPTDYSFIKEKPEGCLKIVSYNVNSLSAAIKKGFKEYVKAENPDILCLQETKLNTPMA

FLFPNNEYPHITWNSSTTKKGYSGTAVFSKIKPLKVTKKLGIPEFDNEGRFISLEFETFYLVAAYVPNSG

MKLERLDKRREFDAKMNEYLTSLEKEKPVIYTGDLNVSHNEIDLARPDTNHKTAGFTDSERKSFSQILET

NNRIDTFRAFYPNERIGGYSFYGYRFNSREKLTGWRLDYFVVSSSLFCRVKDSFIRSECYGASDHVPIVM

YLSNE

>XP_009494919.1 hypothetical protein H696_02739 [Fonticula alba]

MASVAPGPPPAEPAPAAAAAAAAGRPQPQAHLLRATWRAFASAANPADKSADSDAAWLRGLPRPPASAVR

IRHDIDSGAFRRANRTNAAGLPTVGFFDLTDRLRRVDHPRRDGAAPAPAPAQWNTLARSLDSNHYFRSLR

PPGPAEVAHLQVMHQALAGLSLPSARRGSPPSHAPGPPASEMLGFDRLVQAVLDTVQHTLDLLKAEYSPE

ATAGFLAQVAAEATASGADPAAALRQAQLTRAYHVLQLHDRMSDCLCLVSDPMEAARNTLPNFEDRSLAS

DSAMDLNNFIQRLNTDLDLCRALFANVFAEVGVVPDPADFRTISSRLTLEPDAAAAGQLPAAPRQRVFDL

LDSEFQAVAHSYAYEYRAGGVAIDDPAIAERLVGLSSDVLSAGMWFSRALMEGRVNVRLTDQQYSRFSHI

LGPPLTSPTTSGHRLVDLPLNTILSDSFMASITCAETRTSIYRQLAGARGLSGEPHRALMRLIESRHKFA

QALGYDSFADLTIIGSASRIVRSPDFIAVFLRSLGERLSPEARKHTSRLAGLLQGHSARFNTSPRSKSPP

APEASLPVDSINFWDLPLARYLDTAQQVRELQAAGQGLSSDERHFYSLGTALSGIAMIAELVFGLLPRLR

RLMTASAAGSSSRPPARRPTASNPAAATSTQGPPKRGRPSNETLRARETLAAKAAPPKPVPATIAPPAPG

ARVTRAMAAAVQAAATATAITTSPVPGLLAHNLQAEGLTAPRAAGPPPDHDPGPPVKAEPKLAGPFALAQ

ARMNAPVMAKKAKVGQSLASAKKSGAPPAKLSRSASAEQQVLRDLAIQAIVRHSRFARRAPRATTAEAAP

SAAFLAATGLVFPVDDVTTPAEAFPSMPRETPADLGFLPPAGDAAAAAAAAAAAAGPAGEGAPPAAAPAH

TPAGALRLVTYNVNGLQAALQKGLLTYVLAEQADILCLQETKLSFMPPGSLEDPRTFDAPALGPEFIQLL

EHFPYRIWSNSLQPLGYSGVAVLSRVPPAANGRVITGMELALARLEETLLPEGLLTEAPMPPRLTRVGPT

EPDAKIPARPVSHDLEGRLLAVDFDDFCLVNVYTPNSGQKLARLDYRRQWDADFARFVRALELGITADPA

AADATAAAATAVADATHADGSTALVPPPPASAAAASGTGSPDEAAASAPAPAPTKAPLVAGRPWRPVMVT

GDLNVSHQPLDLRRAKANQRTAGFTLEERAGFSNLLQTGQLTDVIRQRHPGQAGLFTYWGVRGDTRGPNS

GWRLDYFLASHAPAPEHCRPAPGQDASRALVDRVRGAGIRQAVWGTSDHVPVFLLLESTAARCPDPAPTP

>XP_004365499.2 APEX nuclease [Capsaspora owczarzaki ATCC 30864]

MTRASTKRTAATASASDAAVDPAAAATTAAVAAPITKKTRVSKASASAAGGEDEQEAPTARRSSRAKTTT

TNTATTTTTVTTTVTVASDTASAMEVDTPDDASVKGKGKKAKAPAKKAEKDDADDDAAETDAATEKTVVA

NVKFSAEALKTLTYTTSRAAPNGKPINFKIACWNVNGLKNAIKEGCLQYLEYEKPDVLCIQETKCGEGEI

PADSAVPGYYGYWYSAQKRGYAGTGMYSKTQPIRVSFGLNVPEHDTEGRVITAEFADFYLVTAYVPNSGQ

KLETLPYRQTWDVAFRTYLLSLDAKKPVVLCGDLNVAHNEIDLKNPKTNKRSAGFTPQEREGFTTILGTE

FVDSFRVLYPEESAYSFWSYRHNSRALNVGWRLDYFVVSQRFMQSVADSQIRSQVYGSDHCPIVLHLSL

>XP_728665.1 exodeoxyribonuclease III, partial [Plasmodium yoelii yoelii 17XNL]

PRPRKPSPSIPCLPMRIITLNVNGIRSAARKGFFDWLPKQNADIVCLQEIKAQTTQLNDELFWPESYACY

YLEAEKKGYSGVALYARKEPDDVIQGLGWDDMDTEARYLEARFGELSVVSLYIPSGSSSEQRQAVKFXFL

DRFLPFLDDCARSGRQYIFCGDWNIAHKPIDLKNWRSNQKNSGFLPEERAWLDRVFEEAGWVDAFRAVNP

EPEQYTWWSNRGQAWAKNVGWRIDYQVVSPGLRELIRSAAIYKEERFSDHAPLIIDYDLTL

>XP_018635255.1 exonuclease III APE [Toxoplasma gondii ME49]

MRPLGSFLPSFVHFDWTFCRGLPLAENLPVSKVTLRRLEPPFASSLAVYSPSALSLSSPPFCLILPASLS

GSSAFSSSRSRPGPQPSPAAAGARTRNSQRTSTMSVHRAVNSAQGEEETEGPHRETTTELNSSAASLSSS

PSSAPSCSVSTSAMLQRASDSSSSGAPPVDLNPSRETRETEEDGEGELERRTEGPKRKAPLSIVTWNVNS

IAARIRDSRQWFYFSRFLQKIDPDILCLQEVKLAAHGPPGAKRGDGMPRDHGRIKDSDKVSSVEARELRE

ALHTLLPNHSLLISLADWRYSGQMMFIRKDVQVCSLRYNLSLDGCPAHEHDFEGRVILAEFEAFCVLTTY

SPNNGATPKSFERRRLWDERMLQFVTQLKKPLVWVGDLNCAPEDIDLSDPDQFRSVIHETADGTIDPDNI

GQAGCTDAERRRFRAILERGNLVDAFRELHPRTEPPPLESAEYSWRGFGGSGSRGLLRGLGMRLDHIVLT

ETLMPAVELVRICGAGKSKANFFGSDHCPVLVLFKEKEISALPAVVCEALRSSAPAPAAKKRQDDCRLDS

FFAVKRRAQLPGAADQKRKKPEEVVISDSSDEERKTSQKIALHASLEKPRNVQASS

>XP_011131910.1 exodeoxyribonuclease III [Gregarina niphandrodes]

MTLTKVRKGGKVTKKVAAKESANREVDSAKGERSKGDMAKSVMKKDRKVRAVRSKSRTTAGKFLITEERG

EESHEMEEGGKVVCGTAVKTGDKTSVKTSVKTSVKTGDKTSVKTSVNTSVKTSVKTSVKYNPHEDGKGPS

LSGCWMKQIREDETRPEGHVLISSWNVNGLRAVCRSGKLEFDRENYSQSGLSRLGDLGAYLLVEAPEIVC

LNETKLSESSVTDFDKGAFHGYESYHVLSDTRGYAGVAVLVKEELACAVEEVIRGCDNSKFDHEGRTLCI

VFKGLVLVATYVMNAGQDLRRLDERVDLYDKHMHQWLSSLHTKYQRPVIWCGDLNCAREEIDIWNSKSNQ

KSSGHTIQERNSLTEFYNKQEFEDIWRIRHPLQRRYTFWSARAPQQRAANLGWRIDAFVTSKDISHAAGL

VTIRDKVWGSDHCPIALSLPRNCIYGA

>XP_012655452.1 ExoIII family purinic/apyrimidinic (AP) endonuclease family animal Ape2 subfamily protein [Tetrahymena thermophila SB210]

MPPKKTKNSGTTQTKLTDKIASKQKKAPKKEEEEEEEDQEVEVEQKKKANNKKIEVEDKENDSDQEEEEK

TDAKSKPSSKGDTYSKEGNYQGSLGNGKFGKDTIVISSWNVNGIRAVQKKGELENYVKSLNPDVICLNET

KIDSDAFKKDSSLMKMIPSDYKQFWNFCKTSKGYAGTAIFSKVEPISVQFDIGVKKHDEEGRTITLEYDK

FYLVACYVPNAGQGLKRLDYRVKEWDIDFQKYLNDLKSKKNVILCGDLNVCHQEIDIAKPKGNEKSAGFT

KEERAEFTKFLGQGWIDSFRKKYPTKVQYSYWNLRNGARKTNQGWRLDYFVVNEKLYPFIQDSTINNDIL

GSDHCPIELKIDNKQLKS

>CEL97691.1 unnamed protein product [Vitrella brassicaformis CCMP3155]

MLLALLPLVACRAFIPSRSHRLVRRPTRLTRPVGPHPCLFPVPAAAAGRRLSTMPAKKRSKKEEEAADEG

EEGEPQPTAPKRKKQKAKAKAKADEDNNYEDGDGGGEASGTSLVGVNQLEGVAAHPDKFKIVNWNINGLR

AVAKKPIFREYIDAEKPDVLVLSEVKGDQTAMQEFMVDGWLPGYKSHWNLCKVKKGYSGVAAFWKEEAEP

NEVKVDFCAKHDQEGGRFIQLDYDGFTIIGCYVPNAGEKDKSTDEKLPKNLDFRTREWDVDLRKYLKNLK

NVIWCGDLNVCPEEIDIWKAKGNEKSAGFTLQERDSFAETLEAADLVDAFREKYPDTKAYSYFSARGKGR

EKHQGWRLDHQIVSRELFESSVQDVIIREKVAASDHVPVVLILNK

>CEM10310.1 unnamed protein product [Vitrella brassicaformis CCMP3155]

MRSVLLACVWLLPSSKGRELRRPAFLRTSIADSKGGGVRFRPLSPLRKAPSPPPTPTLPSWSTCCAALTL

SAATNEREVPPLPPPPSHPVPSVPKRSTGASTTTRTRRAAVAPAPTRRRKTTTSTTARKSSSSTSRRGAA

SPSPTPAPKPVPPVTDLPRRLPDECVEIISWNVNGLRANLRNGGIQTLLEEHPHLDFLCLQETRVSPDTP

LPEFDAIRQHFPYCYFHCAARAGYSGTAVLSKTPPLRVLLDINLGGGEGGEEDKEGRVVTWETESFFLLS

VYSPNAQGELQRLEYKHRQWNPAFFAHIERLRRQKPVVIAGDLNVAHTPKDLFSPLESEGLPGYTPEERS

DFSDLLATGYVDTFRNCHPDMGGAYSWFSYRYKDKQRNRGWRIDYVLVSDRCHDAVKGAYIEKSIDGSDH

VPVGVVLNTTVLSAVCGATTAKGGGVKKNEAVKKGVSSRLRSPLRPLCRSRLNSLEEGTSAATAERTAVG

VSPSQANGAPYAAAAPTTRVDGDIEAKGKGVAWYPEWSSDRAIEHITTDVSMTDLVSLCRRRGFVFQSSE

IYNGMNGFWDYGPLGVEVHRNIKNRWWDKMVREREDIVGLDCSIIMAPKTWEASGHVGGFSDPMIDCKES

KMRYRADQLYFARVELESGELLGYISMLDGDNNEAHAKKLVKTLMKKADVKGDTKPLELKDATQAAPEEI

PLIPSPATGIPGSLTPPREFNLMFETSVGALSDAASKAYLRPETAQGIFVNFKNVAQTSRVKVPFGIAQI

GKAFRNEINPRNFIFRSREFEQMEIEYFIPPDEDAWPEYYKQWIGESWDWLKSIGLREDLMSYDVHQKCD

LAHYARACTDIIFKFPFGESELMGIAARGDFDLSQHEKTSGKSMEYFDSDPNAADKRRYVPHVIEPSLGV

DRLFLAVMVSAYVEEKVDGEKRTYLKFHPSLAPYKAAVLPLVKNKPELVSKARGIFEKLKRRYSVFWDAS

GSIGRRYRRADEVGVPFAITVDFDSLQDDSVTVRDRDSCEQTRMKISDLYPFLTQQIDPW

>CEM33536.1 unnamed protein product [Vitrella brassicaformis CCMP3155]

MKRLPEAASAAAEKRVKRSDPLKIVTWNVNGLTTRIKDHKQWKDFESFMETEKPDVVCLQEIKLCAAAPP

GAKKGDGRPRDRGQLRDNDASTREDADRVNRAFKSSPVLSSYSLFWSLADWRYAGCLMMARKDIHVRSVR

YSLLLPDKLQSVHHEEGRVILAEFESFAVMNMYTPNNGWQTEKFSRRHRFDTDVQQFVKHFTTDSTSAGE

ASSSDAPPTSSGKPLIIAGDLNCAATDLDLSPGDERWFRQQTEGPARPPPKYVGQPGCTDGERADFREIL

KAGSLVDAYRQMHPRGAPSAESPCFSWRGWGKYANKGMRIDHILVSESLMGRVESVRITGKGTARGGMEG

FMGSDHCPLVVQLRPTAGGGAAAVAAGGGGGGPSASGQSADAAGQECIDLTD

>XP_001020979.2 ExoIII family purinic/apyrimidinic (AP) endonuclease family animal Ape2 subfamily protein [Tetrahymena thermophila SB210]

MIKSFRFSASSLLNFRNQCFNTQRVGKVFYAPKLLTFPLLKQNFTQLEKGSDLQQSVEKQTKKKKGMSKI

IKKSSSISEPEQSQSSTTTQKTKKQAQLGDFFAGIKKKPETQVEDKVEINTELGDGIWKDRQNIKIACWN

VNGLRAVLKREDLQKYIKQENPDILCLNETKIDESNFIKEKVYKDIPSGYYQYWNFCIPPIKGYSGVSIF

SKEKVISIKKGIDIAEHENEGRVLTAEFDKFYLVVVYVPNAGSELVRLDYRVNSFDKDFQNYLNQLKLKK

NVILCGDLNVAHQEIDIANPKSNRNSPGFTIEERNSFSKFLESGWIDTFRFLYPETIKYSWWSVRTKARE

SNIGWRIDYFVVNKEFKSSIKDSMINNNIYGSDHCPVEFILNLDAIQQNETSSNENTQTNKSN

>EJY66875.1 Exonuclease III (macronuclear) [Oxytricha trifallax]

MSKKNQKRSLKETETVEIVQEDEEKITISKKKTTTMSRTTTKGQSSLSGFFGVTHNPSAKFHGDAKHEDF

KSDQLNIWVWNINGLQAVINKGMLDVFLQGEDPDIVCFNEIKTDPEKIKSASFHTKIPKDYEQYWNCSKS

KKGYSGTGLITKIKPIKVTFDIGIEEHDQEGRVITAEFSKFVLVAVYVPNSGDDLRRLSYRTQEWDKAFF

DYLDRTRIETNKPLILTGDLNVARNELDVFDTKGKDKVACYTPEERKSFESFINRGYIDTFRHLYPDKRE

YTYFSARFNNKVTNKGWRIDYFVVHQDDINMVTDVTIHKDYNGSDHVPVCLHLDLSKIKKEEEEVKGVSK

VKSKVRGKTPTKASKSKSRSKSKSERKEKDDETESEKEDDQEESKETFTQKKVINSDEKVLSNLKSKSKE

ATEQPREDSEEESESLIEVQRKKLTSNKSKDSKTTAMTAFKIPEAVFTNNPFSTNDLMNALPSRSTFKDS

TYPAQMTLPQKRQSNNTMMEMMTEVNKRYHQRMAEYAGQTLSTADQMRIKQEITMQVKRELDARKSNHDS

SESGVVEQKPKWDQEYQQFDEEF

>EJY83183.1 Exodeoxyribonuclease III family protein (macronuclear) [Oxytricha trifallax]

MNRRDPNSFSANKRKQIYERHLVPTDKGPVAKFKKIAEKNAAKGQSSLEKFMGPQNGDGVKSNQAAFQHY

AVQSKSHRQTRSNSPLNNEEYEDNGADTTASSNGKTSQPNQQNQNFENNNKTQYNSNSQYKNSMNGDNQM

LGKRTFVGYQKNEYGKQNGYGGGYNKGNGQFNRNNNSYQNGNKGGNMQYNRDNKYNQKHPNSNQQQQQKG

GLQLRQIQRPMNIQEQNQNIDMSRLQIWSWNVNGIRAILKKNRIQEFFEQANPTILCVQESKIDEERMAA

DKVKDKFPADYLQYWNCCKPPIKGYAGTIIFSKIKPISVSYDIGVHKHDKEGRTITLEFEKFFLVGVYVP

NSGATLKWHDYRCSEWDLDFRAYLKGLERRGKPVILCGDMNVAHQEIDIFDPKRNERMACFTNEERQSFS

NFTKMGFVDTFRELYPGKIKYSFWDIRDKSRKENKGWRLDYFMVSRQIKHALTDAEIHNEFWGSDHCPVS

LTLDTTKINLEEFNEYMNTDLDDDKDELDMEDALNEEVKSDLEDDQNPDEFQYQYDENDEEADENLGEKF

NNGDDENDQENPEENQDDDDQEEDLGDDALAGGQDDDPGNDLEEEDNNNNNAGDGDEDSQ

>EJY82409.1 Exonuclease III (macronuclear) [Oxytricha trifallax]

MSKKNQKRSLKETETVEIVQEDEEKITISKKKTTTMSRTTTKGQSSLSGFFGVTHNPSAKFHGDAKHEDF

KSDQLNIWVWNINGLQAVINKGMLDVFLQGEDPDIVCFNEIKTDPEKIKSASFHTKIPKDYEQYWNCSKS

KKGYSGTGLITKIKPIKVTFDIGIEEHDQEGRVITAEFSKFVLVAVYVPNSGDDLRRLSYRTQEWDKAFF

DYLDRTRIETNKPLILTGDLNVARNELDVFDTKGKDKVACYTPEERKSFESFINRGYIDTFRHLYPDKRE

YTYFSARFNNKVTNKGWRIDYFVVHQDDINMVTDVTIHKDYNGSDHVPVCLHLDLSKIKETKPNDTKLLA

VNDTSNKNEKDQEEEKYNKIKHMDISQFSYSKKKQTSQAQIKAQKDETNKQQKTKQKDRESSDSNSSGDE

QNNSKDNQGINDDANNHVQVLKDCAYSYIEKVYKKPDELEERLNYVMSQTNIPQFAQLSLQDKKEIDKEV

EFRHMMNMRKNTAAGIQTGMKDILTRKWQLYQKVTDEFQDKQINQSSQQEHTQIIKDNMNNDAFESVSNA

KPAQQSSIPEPHYYTADMKEEADRRYIWKIQELTLNGKTLSVPEREQIKNDIIDQLKIEYKNNQSNKDQQ

KSKLDDEYKQYDEEF

>EJY67231.1 Exonuclease III (macronuclear) [Oxytricha trifallax]

MSKKNQKRSLKETETVEIVQEDEEKITISKKKTTTMSRTTTKGQSSLSGFFGVTHNPSAKFHGDAKHEDF

KSDQLNIWVWNINGLQAVINKGMLDVFLQGEDPDIVCFNEIKTDPEKIKSASFHTKIPKDYEQYWNCSKS

KKGYSGTGLITKIKPIKVTFDIGIEEHDQEGRVITAEFSKFVLVAVYVPNSGDDLRRLSYRTQEWDKAFF

DYLDRTRIETNKPLILTGDLNVARNELDVFDTKGKDKVACYTPEERKSFESFINRGYIDTFRHLYPDKRE

YTYFSARFNNKVTNKGWRIDYFVVHQDDINMVTDVTIHKDYNGSDHVPVCLHLDLSKIKETKPNDTKLAV

VNDTSNKNEKDQEEEKASNSKIKHMDMSQFSYSKKKQTSQAQIKAQKDETNKQQKTKQKDRESSDSNSSG

DEENNSKDNQGINDDANNHVQVLKDCAYSYIEKVYKKPDELEERLNYVMSQTNIPQFAQLSLQDKKEIDK

EVEFRHMMNMRKNTAAGIQTGMKDILTRKWQLYQEVTDEFHDKQINQSSQQEHTQIIKDNMNNYAKPAQQ

SSIPEPLYYTADMKEEADRRYIWKIQELTLNGKTLSVPERKQIKNDIIDQLKIEYKNNQSNKDQQKSKLD

DEYKQYDEEF

>EJY86966.1 Exonuclease III (macronuclear) [Oxytricha trifallax]

MSKKNQKRSLKETETVEIVQEDEEKITISKKKTTTMSRTTTKGQSSLSGFFGVTHNPSAKFHGDAKHEDF

KSDQLNIWVWNINGLQAVINKGMLDVFLQGEDPDIVCFNEIKTDPEKIKSASFHTKIPKDYEQYWNCSKS

KKGYSGTGLITKIKPIKVTFDIGIEEHDQEGRVITAEFSKFVLVAVYVPNSGDDLRRLSYRTQEWDKAFF

DYLDRTRIETNKPLILTGDLNVARNELDVFDTKGKDKVACYTPEERKSFESFINRGYIDTFRHLYPDKRE

YTYFSARFNNKVTNKGWRIDYFVVHQDDINMVTDVTIHKDYNGSDHVPVCLHLDLSKIKKEEEEVKGVSK

VKSKVRGKTPTKASKSKSRSKSKSARKAKDDDTESQNEDDDEEKKDNNALQESQDKDTVNDIESESLLLK

SSSTVIENEKISLNDYESLQPSLKLHFSSEKAFQYLTDNKNSSYPLFGNAPGTNPNLNPVTQIDDTSPLL

TNYSNSFLTFYLGVIITNKQIIGCFTAGKSGFIWGNALDSLPDEMQKQLKKRIDKEHQAEMTRNFEQKLQ

KKFGFGIDKIQLTDRNVQNLALLQQISEILLQVKGEQEKKVLGASQNVENYSKSKAWDHDYKEFDEDF

>EJY69586.1 Exodeoxyribonuclease III family protein (macronuclear) [Oxytricha trifallax]

MPPKILQKREANDQPSQNVIIRIQQFLFIQGNAKEEEKKEDVNKRTQKAEQKQLDFFIKKQNGKDIVNDK

NEEIKKEVQQNLYGDASQEEPLKIWHWNVNGIRAVLKSGKFQEFCEKAQPHILCLNETKIDVDALAKENI

KLQIAKWFPIDTQYWNCCIVKKGYAGTAILINKNYKGSKPTKVEYGIGISKHDKEGRMVNAQFDKFNLVA

TYIPNAGVMGLDRLGYRVNEWDRDFHTYLKNTEVTTGKPVIWCGDLNVANEPIDIYNPKGKEKSAGYTIQ

ERNSFKAFLKDRAFIDTYRHLNPHSVKYSYWNLRSGARDKDQGWRLDYFVVSDFMMPSVLTSEINNEYHG

SDHCPLSLSFHPDKIVPNKGAAHLQLVDAKVDAAEGSPQKVNGKLTEQNPESKDIKSKTKELIKSSAAPI

IQQKILPKLTPQKGLVD

>EJY88283.1 Exodeoxyribonuclease III family protein (macronuclear) [Oxytricha trifallax]

MPEVIPHIYGKALYEDFPQNKVNIWHWNINGLQSVINKGKIQDFMTKTNPDIVCFNETKTDLEKIFKDNF

HSHIGQEYQQYWNCCKIRKGYSGTGLLTKVKPLRVDFDLGISKHDNEGRVITAEYNKFVLIGVYVPNAGD

GLKRLDYRTQEWDNDFHDYIDRIKVERGKPVILTGDLNVARNEQDVYDTKGKDKVAGYTPNERGNIERFF

DRGYIDTYRHLHPEKEEYTFFSYRISGRENDMGWRLDYFIVDKDNINMVVDKRIHKVFDGSDHVPIELEI

DLGKIGQKKKENAKKSPNKLINDKEEIQKLKISSTETD

>EJY66828.1 Exodeoxyribonuclease III family protein (macronuclear) [Oxytricha trifallax]

MKTENQNTQINDQSEDSNDFPDNVVTKRREQKQKQSPKKQVKTNEEGEKPLNRDQVVKKYLEELKKQQSK

EEMKKEKVVNVDKYKIGPPTVQKFFGEADLFDIPKKGHMSIWQWNINGLQAVVRKNALQDFLKNYQPLIL

CINELKTDFDKINQLQIHKQLPKNYEQYWNCCSTKKGYAGTGLLTKIKPLKVTFGLGIEKHDTEGRIMTA

EYDHFIVVNSYFPNTGFNFERIDYRIDEWDEDVHDYIDHLRDTSKKPVILAGDLNIGNTELDIYHRPWAR

NFRQGQHPKEYDSLQRLLDRGYIDAYRHLYPNIRGYSYWSSQRKAKEKDNGCRCDYFILNKEHFEMVIDS

RMHRDVEGSDHVPIELEIDIQKLRGYSKSMKLKSKKKLSSNGKESSNVNNNKQSMKKQIIA

>EJY72731.1 Exodeoxyribonuclease III (macronuclear) [Oxytricha trifallax]

MKDIPKEGKITLCQWNINGINAILNKGALQIYMDEMKPQIVCLNEVKTSHERLNETGLHMKLPTNYEQYW

NCSTIKKGIAGTGLLTQIKPEKVLFGIGIEKHDVQGRVLTAEFKHFVLINTYTPNAGMFFERIDYRVDEW

DVDFRDYLDHVRDTYKKPIILTGDFNEAFCAYDIFERPQKKLKHPLQGFHPKEYDSMKLLFDRGYIDSFR

HLYPTTRELTNFSYRSKNPRERNHGCRLDYFIVSKDHFDMVIDSKIHKRFLGSDHVPIELEIDAAKLRKI

SKVKVKQEKFKEPSLSKIQKLNNQEVLSGGKRREKKELSKKKLKNLDLEQTKKLNVKENLLDLAVKVFNI

KEQSKFSGLAEFQGILELQKSLKIWAWNINGLRSITRKRALSQFIESADPLILCLSEIKIDQQALNKSGE

DFQLPTNYEQYWNCSITKKGYAGTGLLTRIKPLKVSFGMGIEKHDEHGRIITAEYEHFVIVNAYFPNGGF

QFEKIDYRRDEWDLDFNSFLDYIRDSTNKPVILAGDLNIGRDQYDLYEGSTLLNRIPKYLLKQENSLTRL

LNKGYIDSFRELYPYKRDFTIYSSLVPNSRQLNKGKRTDYFIVTKDHFDMVIDNKMHKQYEGSDHVPIEL

EIDTKKLLAYQNIK

>OMJ79843.1 hypothetical protein SteCoe_20039 [Stentor coeruleus]

MSKRKVPAEVPKKKPKTLSAQSKTVSGQGIFSRSEIHIVSWNVNGIRAWIKKNNVLDFCSKEEIDILCFN

ETKLQDKDVNDIKKRFPQYPYQYWSCSQAKKGYSGTAILSKIEPMSFTQGIGKDKHDNEGRTITAEFSTF

FVVATYIPNAGQKLERLKYRTKEWDQDFRKYLKTLESRGKGVIWLGDLNVIHQDIDIFNIKGKEKIAGCT

PDERKEFREFLNEGLVDSFRHLYPNERKYSWYSGRSAKARSENMGWRLDYVIISESLVSKLRDSKIYDDV

EGSDHHPIEVILNN

>OMJ89763.1 hypothetical protein SteCoe_8031 [Stentor coeruleus]

MSKRKAVSEAPAPEKKSKISEDTAIARPLSSSSQGQFSKPEISIVSWNVNGIRAWIKKSSVLQFCTKDEF

DVLCFNETKLQDKDVNDIKKQFSQYPYQFWSCSQAKKGYSGTAILSKTEPISFTEGIGIKKHDDEGRTVT

AEFSNFFVVATYIPNAGQKLERLKYRTKEWDTDFRQYLKTLENRGKGVVWLGDLNVVHRDNDIYNMKGKE

KNAGCTLEERKEFSNSLDAGLVDSFRHLYPNARKYSWYSTKNPKAKSDNMGWRLDYAVISQSLVPKLRDS

KIYDNIEGSDHHPIEVILSNN

>OMJ78824.1 hypothetical protein SteCoe_21265 [Stentor coeruleus]

MKRKAASASKPTKLSKTYQTTLFPFTGKPAGRGAFTNKSIRICSWNVNGARAWVKKQGPLQFISENKFDI

LCLNETKIQDKHINKMKSHFKDFPYQYWSCSKAKLGYSGTAILSKSEPINWSEGLTGHPKEGRVTLGEFD

TFYVLSTYVPNSGVTRLDYRLNSWDKDLRKYIKDLQKIKPVIWLGDLNVINLDIDIYRLKGNDKCAGATI

EERSSFWKTLDIGLVDSYRLLYPEVRKYSWFNPKDVNARDKEEGWRIDMAVISHNLVPRIVDSRIYDNVL

GSDHHPIEIVIDNN

>OMJ78347.1 hypothetical protein SteCoe_21865 [Stentor coeruleus]

MNSIKIASWNVCGLRSLLKKNALDKLIETENPDILCLNETMLQTKNVKEIDKLIPQYHYQYYACSEARKG

YSGVAILTKTEPISVTTQNIEKHDREGRTLVAEFPYFFLISTYVPNVGAELKRMDYRIDEWDSDIRTTLN

SYQERKPVIWCGDFNVVHEDIDIYDIRGKECYGCCTPQERGSFRKTLAETRMIDTFRFLHPGEKAWSYFS

RRNVKAKEKGQGWRIDYVLASRELESKLTDAYTRTDIEGSDHHPIVAVFNNLD

>OMJ93116.1 hypothetical protein SteCoe_3930 [Stentor coeruleus]

MKNTLKRKPQITSKPEKKQKTLQSLINLPPKVLSKTSGLHEFSQPEIHIISWNVNGLRAWIKKPGVLDFV

NRAEFDIICFNETKLQEKHVIDFKTKFPQYPYQYWSCSTERLGYSGTAILSKVQPLSWKSGLPSHPNEGR

LTLAEFPNFYLLSTYVPNTISGRSKYRLEKWDQDLSQYIKNLEISGKGVIWIGDLNVINTDIDVYQLEGN

ENCCGGTPEERKSFHNIIQDNLIDTFRYLHPNTRQYTWFNTMRKVAKSRNEGWRFDMSLVSKSLISQVKD

SKIYDQIYGSDHYPIELIMTNGI

>OMJ84431.1 hypothetical protein SteCoe_14441 [Stentor coeruleus]

MRILSWNVNGLRAWSKKPSTLSFVSHKDFDIVCINETRIDSSLVNQASSLFPDFPYKYFSCSNKKGYAGV

AILSRIEPICQDTSSELFFNNNGRVITLEFEKFFLVSSYFPNSGGNDEFIDSKMKWDEDFRNYLKEIMSK

GKEVVWIGDLNIVSGELDYFQAKGKKVKIDDKELQSFRKFVELGFVDSFRHLNPNLKKYTWFSNKFPQNR

INNKGWRIDYAMVTDGALPWVDMSLIHENILGSDHTPIELVLSFSS

>OLP80270.1 Apurinic endonuclease-redox protein [Symbiodinium microadriaticum]

MTLAVDFRHVIFVSAGCASMAMRVSSSDTEAMPKGEDEEEEEEDDDDEYIDEVVSPPLVSNPCATGPSLN

FNYWDRGRQVLQTDLVNLRASLRKLAIDLAKSGYIHKLSAMLREHDPKMLLLPPRQDAQVGPQQTLFSAA

QASGHTAVAEVVEEAATDLLFRELSKLTSMQEVEDDIVQQCLHAGANPTRPGGAHGHLPLQHLCIQEMRQ

PADVFTAKKVAQAASQLAEAAPLVLIMAFARPVARAEATLPLGAAACNKSFGKDIVPVLSFAVSRLLLKR

PDLAAAPLLKASVEAAHNKFPNVPFVLPLVEILRAAAPCLDSATGTHVGKEVRIWEPRRSYESWTQRKQR

LEILRPMLSQVHRADDSCEREEAIARLAEVEAVLDRRRRQHSEQIAELQMAMDKAQRAAEEASAQVQAVL

SQRSQGSVKLRSELHGEVHADGKSDVKFDQSLPHTGSPPDAPAELLRNIRQQRGLDIDYSALPPAVLQSA

GAMQRSIGAAVERLAIDLYASKGHFLLELIQNADDNRYNTSGAEPAMTLHMGSAEDAFMNAMMPNGIWLK

TGRQACLLQSSSNLFWLAADRKVDPSIRSAACDDDMPAKFEKKLSDSVSEVKDLESPSESTPVPKEKQAD

KNATPAKRPAQKRVKKDVKDAEEEEADTSNKKAKRAKKVKEGPYTSFKQGSIPEPQLQRRVDVPEGKAGL

NVLSWNVGSLRAFLRTRTADLQEAVKLAAPHVLGLMEHKLQEGTPETESALEELSKALPNYRVACVNYST

VKKGYSGTLIMLHTEAPEPISVLAEDLPAAAAEGRIVVCEFSSLFLVLCYVPNSGDGLKRLKERVEKWDP

QLRERLHALAEKKSVVLMGDLNVAHQDKDIWNVEAPHVPKSAGLTPEERQSFSKLLESGFKDGFALQHPE

ALGAFTYWSVRAGNRKTNRGLRLDYVLVSDAMTSKGEGEDGPVLCDTFHLPAVATGDHCPVGAVIADSSL

FFASINNELGMTSKDVEALCDINRSTKPAQAGKIGKKGVGWKAVFAISDQPTVLSGPFRFRFDVRCRGRL

GYVTPDDLSAQEQSLLPAFLQEASSGGATVLYLPLRGRPATEDGAAADMGDGGLGIAALIRSSMDRLLMY

PAWLLFLRQLRRVAWDDAVSARPRHVSLERHGDSVFVRQLANKGMAEPDEEFAFFVHRKSGVVPSDLLPA

GVEPGSRIEEVAIAFQHAAVQDQRAEAPESHGQADRESDPVFCFLPVRPVGFRFSLHAPWSLTSNREDFH

LEDPRNVWLRGVAADALAEAIARFGSEPGTNVLTLLDARRVLEPFWRRLLEEAVERIGDAPVVAVVGESK

LYRPSEVLVPTPSLVRCTAAMRFIHALPAESWPAATGKRLARLSANEDVAVEDVRRLLSLHAEPVSAHKM

RVLLNSDSVKAELTKHCRSRNPLGLAQLCELLSAMLAATQREPEKVPTFSEAGELPGDERISDVVTEIQQ

MQVLPLATAGSAPQMTSLSEGDVHLPGSGTWCPGLDEVTASTLLAHKDVRVFDSVSWQSLSSGGQNFLRQ

LGVRKEAKGFRRDWTIEDFRKSTGMGRFRVSGAIFVECSNQPALEEAKWILRLMEETDCPVVGLVANICV

QRGASEVQEFLDQLRDSEGMLPKGLKGARCVCMMWENQADDACLDSRFLEGLDCLGRFGLIWEFCCEPRM

APYLSACIERFPHMVFVIDHLAHNGNRGGEMEVWGPAMDSLSKLKNVYVKLGAPEQWDVPNPADFLDRAI

KAFGFDRLLYESNWFVNEAMGDSYDKTADLVYDACMRAHATEAELRKVFHDNAYKVYQIAMLSEVAAAIV

RIHTLTLAESVNMQCFSGSFARPSTAEDQEEHELAVLWAGLDAIRLAKEQCLDPARPAREDRARAADPLD

PAVSFAPVFRRTDALQMGAAELGHVLWLPTRSASGEVWLRRAKGLILPTFMGLTASEPARGGLEEHMQDL

GDDDGQAEKATVLPPPSRQSGRPCFVVAVPASGQAGGWNECLLLREAFLTELGCAQPGIENLAVDRVAER

LVSRQLWEALAKSMELRRHAARVFRENHGWLPDLEVQARQDQPGTGQRINSYFRRGAFEPVVGEGGLPYV

DAEANCQSLLELLRIRCTPDFENLAVALRIWVDKEPPAMCAPKALVTALLQRAIAERPADSVLAILRNLI

FIPGKGKISAQEAVWSAGTVDGDFARRVCALASTSMLEEIYGQTLRGWFCEFLSVRESPGMWQLLRALQR

LIPSHAGGEGDALAPSTKPSLSFLRQLYAEIMMQLEEYIRESVVGDGQQSRLKRRRTEDGHSLIETVESK

FQSRRLIILPQTRNRPWKFLATTQAYWSVDDELSELPCSQFALKNFYGVQVKTRSEECGTGLAIIDLKDF

FVQVLGVKEVLTREELARRLQPTDRPQRPPQARPDRAAVAGEVANANMDADSDVSSEDDAGVREDDGALD

LEDLLGDEAGALSILPPGAARGGRRGEASQRSAAAPGAHDMDVDAAAMRNLLARCCQRTRAAQPAEVLYS

GPGAGQQASATAEVSLRRMCWSYRGANLYACGPPSLMANVEALRNLGITIAGRQAAHPKGSLLPSSGLQS

RFTGFVDAVLWPLASFFQVPIGSLAAAVGPGIGRGRNMHGLICFSLPETVAVSRPADLCRWYCEMCRLLA

GDNAADFSTAEVSSALTAEHMPKFLQFQDVSTRQPQPLSVDEKAQVTSATHDLHRCELQVSDLETSSSSD

LQRQGFVLFALVSSFVLARGRAPKAVLEASFKERIQEVLNGSATSAAEHLAMRLDSKPGHLFAGYEVEFR

DGHVEWLHGCLDVVTSRDSVENCILRQLQALTALCDLQGLRYNEQVRKTAERHLEVLLKEQMLALLGMSV

QKVSGASILLVQKQPSNSADVDASPAAQASALMALVRCGEAVGNKEHLNDAEAWFQGLNMIYAESIWNKS

FTTFHRAAITWESLAVTALHIPKHSSFRDQLEEYIRDFEVFLRQAWEAHGDFWSFSSARALAVRWSSKGL

RKQQRKLLKRWAQEHVDRFLGRSSGIKAGPEGVSEGILARIGGNRYTCGPLQGLASLAGMLKDAELIQVV

LQLMEKDILRYQLSDSNPGPFRSAPPELLGAFSRDGEQMNLESRQSLRVDDTAMCLIAISHLIETLQSIR

GVNVDPTSEQGELQREARFLESVRGRFATPRPPRGTEVGRKAMASFNDAASTAAAPAFQAVVFDLDGTLV

DTEMATCAWIAELFQPHGLSWTPELHRQIAGTVGFFPQKCLDLLQVPPGRRDELVAVLPQRITSAAYNKF

LAKRMEIKPGAAALVRRLQSMRISLGLCTSRRGDGVESLLQERSDLQTLLEPLKEVRLVGSVDPRTGAKL

PSKPDPQVYQACADILGAAPSSCLVFEDAPAGVQAAKAAGCFTVAVPEAWMEGDAQAEAVFATADLRLKS

LEDFAEDQVLWSRLFGPPCPLLIACGNATVDVMCTVEANELEKLGLSSGTEAAGLSEARKQELVDFAQSQ

EDATVVAGGSAMNTVRVASWSGGDHIRAAFIAAVGMDEHGKLLEEALHEAGVVPLLKRIGQSHTGICGCL

VDAGTRDRTLSVIRGASGLLDPAWIEQPDVSSLIREASVVYITSFVLTTQPRIAAVELLIENGLKSGACL

AVNLSSAGLISKVHSVLQRLLPRATFVFGNQFELRAWGRHLSWSGSDTDMAAELASMLRPGGMAVVTAGA

SPTIVAQTDAGVQVFPVSPIPPEDVVDTNGAGDAFVGGFLAAVLMNTSKEECARSSWSTWVHRGHQCAAD

ILRQQGCTIPCRENGMACTCVRSSSLLLQANLPAPEAHFQ

>OLP75222.1 Apurinic endonuclease-redox protein [Symbiodinium microadriaticum]

MRRSSCWGPFSKMTVTMAVACGVRHHMLMPLSRGFALPQFQPSLKVLSWNVAGLRSMLKRDDGAELFAVV

KDEQPDIIILQEHKLQDVHVPKHEPSVLRIFDEACPGKRPYRATWAVSSQRKGYSGTCAIYHSDAGGGVL

GVTTGGVDKVDQSEGRSVCLNLGCGLRVVGVYVPNSGQNLSRLDYRVKEWDRNLQSYMAEGTGSGVLLCG

DLNVAHEDIDIWNAATPRIRKQAGTTPQERESFSRFLSELDMVDAFRWRYPDVPDAYTFWSRRLRHREVN

KGLRLDYFLVSRALAKGTSQATLDDCRILDGYGGSAVYAKWNAEKFSDVPRLMDKYKDQEDEIYDRIVRK

YVFCRSQKDWQPLIEAMYRRFNPSKLQELDSIFAKYKDSEAALYRALCDKYLQTLSPDGEPLKFNVWELG

TDPVEVGEASELEVLDSPQEEAPPIRLVSPSPSQPEDNDRENNGAEDPKKDREQSEARTSPHEEQDARAE

QPGAAEELLAASLAALDAEGKEEKKKKKKRRDGEAFPPPLPRPPRESMAGVSDFPDVILGLTQAPAEKLP

DKSRRASNSLRPKAAPRPPLTQPQEAGSGQPVPPLDPVLGTTRKVRRKRAENGENVEASAPEGRRKKRRK

VASSRPGNATVSQTAPALPPPTLEQRRLQLKEKLFELKTQISTQAVPKHGPPMPLPRPAEDFWGQPMKAS

HGSAFEPDADSYSYSEDDADWCAERQAESRTVVLRNKLEAQLRAKLMHTIHPKVPEAA

>XP_002786115.1 Apurinic endonuclease-redox protein, putative [Perkinsus marinus ATCC 50983]

MPSRKRSRSEERVTSPQGKENSNEQNVEPPPSSPPHSKKRTKASNKPAAKEKGDVPSKVVNVAYGEVNKK

GEKVPMTTVERHKKGKEDFTAVAWNVGSLRSLLNHKVKELTDLCKKEQPDVLGFMEIKLSNEDMCEECDN

LLREAVEDILGPVEIVWNHCTAKKGYSGTAFVVRKSAGKFDSKLGIDGHEDPEGRTIALEFDDMVVVLCY

VPNSGQDLNRLAHRTSGKECFDAKLAKYCGSFDKPTLLLGDMNVADRDVDIWNVDKPHIPKSAGTTNEER

ESFREHYTSKGFTDTFVNMHPEATGCFSYWSVRARNKPKNRGLRLDYVITAPGFPMDKIKDAFILEDFAP

HGDHCPVGITFRRSKKHPLKRQ

>XP_002776572.1 retrovirus polyprotein, putative [Perkinsus marinus ATCC 50983]

MRKYQTSKGSRRSLMDVMNDAKTAERDLRKRATEFKDTVAVTTDRPEYKHKPFSQRHLPPKPGQAENRVA

CVEGSSSAVESPSDHETVGLCRASSIDDDGDDYVEDGEDPFDIFCSLGTGALIATTCGHVSANSDDTGMP

ARDYEGIRSDIESPSGQPYNLTRSVSIKCGDELLVSPPDAGNPVTLIRPDVATKLIKEGCARGPYSLRGT

GFKLTGVTGGEAITAKTFVVMKVHLKPLGSQEYSLCSFVQALIAPGLTLKFLIGNNTLHSWNWRSFWRGA

PNLIVVDVEKRYNNNLDSCLIPKESIRSETGERWRIALPLERSMGFREKAPDSQEHAKVLCGAVTVIPQD

GVLQVPVTVGGVVTAEHPLLITPSLSLLPKGLEFPECVVTVAAGTYAARILVRNRSATDQLLEAGTILGE

ATCIEENFDVHNMETITHRAALYRCSEQRKACIVVGYLPTTTERRVVVRFPGEENELPFAVDPSYIGIPE

DGRVFESGDESTVVSTGLPKPPPLQHPIEEVQVALIESVVGEILNPDDPKPKRIHRPAITRKRQGGPSML

SWNINGLRGFLKSEANVSCLNRLVRNELPAIMVLTETKLTQEKKDRAFTKLLDSLPVRYDVFSAESAGRK

GYAGVAVLLKRAVDVGYDDGLCLIHEPITGIPSRFFDGEQDEEEDTTSGRVLTIELRQCTLVAVYVPNSG

GDLRFLEYRSKWDVAFRRYMKWLRSRAGEKPLIIAGDLNACGSDLDIGDTRREAPHSPSIQPCEVEAFER

LQREVSLCDTFRIVHPEVAGVYSFYCNAYEKKLNFGRRIDYILVSSEQSDCVVDSTVFYAEFGLGYRPDH

CPILAELRPSFWGATDVAVQCGSNDIIGHCSFDGECHQAAVETFVGQKESQTGSDEQFEEQREVRCRLMR

ELIAGLRSSELPPSTLLDLIAPLLYSDEVDLEEDARTCGLYNAVSAMVCDVEAKCQGMVRSHLHLDETCV

AGNSTEKARLEELVLRHWDVFDYEGRDLPEMTGVFHEIQLSPGAHPVYCAPYRMSPEQKQALEEHVKDLL

SQNLIEPSQSEWQSPVMFIAKKGGGWRVVQDFRRLNQLTEIPRYPLPLVAQVLDELSSSKIYSAFDAKSG

FWQVPLASKSRYLTAFGTHVGQFQWRRMPMGLAGSPPTYQRGMNRAYAPILYRCCILYLDDAVLYSLLRA

GHFEALELFFDLTRDAHITLTARKMQLFRTTLKYVGMRISAEGVRMDEAKVNAILDMPLGRDSPLKNIRS

FLGSTNYFRKWLKDYSRVTAPLRAVLKKDGVGWDNECDIAVAELKRLLTSDPLLVWPNFSKEFELHTDAS

DVGYGGVLIQRDAEHRERVIAYGSRAVGDLERSWTSQEKECYAAVIFVEEFRPYLQDRPFKLVTDAANLR

WLLTTEHTTPRLMRWAIRLSAYGIEIVHREGRLNAVADMLSRVSPKPLEGQGGSTVPVEVATVQEGSGMA

EKTSEYLEALEDPRAEVVLVSGPAGTGKSMLACEAAAKSLDRGRVKRIIITRPVVPVGRDIGYVKGSVAE

KMALWVRPLLSYLSRFLSEGRVKELQAEGSIQVIPISMIRGYSLDDTWLILDEAQNCSASELWAVLTRAG

IGSRMVVIGDMEQCDVSRDSSGFQELLNKVAKLGPEPPLSIRSVVLTKADCKRSPVVKLLLELKDRVIAG

VVECCEKIDYIGYDDLPSSANLLLQEWVGHVCGVVPISKSHLGMSPETWTVRRPLKAVLAAVKQLHPSVT

FEAVRIERGGIRLVKCRGVVGMETATAALDIGFPLSKEIDVSSSTFLPLLAPELVEWIGPLLVEEVEEAL

SSYTCFTGSEDPDDVAVRKAAEAMVHHKPVRRLQPSPSASRTRRQAAYSNPCQKYGCTRTADLSDGIERL

CKYCVDLCKNDTCRRPRATGSLYCTSCKRNFSEGDAECEGLQAAIDYDNPGVQEADDGEVPEGIQQSLDA

SLLADLRRRDPSSETLRYVKQLQEEDPEIGPLIKFLNGDTAGLTKDTVLKIRAKAERHYLDQLLKRTQYD

ESLDTIFHQIVVPQALRASLLDLYHNGLESGHPGRYGMYNMLRRQFYWKGMWRDCSDYVRRCITCRSIRR

GPSVYGNVHQHSRHVAAAMQRVGIDLVGPIHLPQYTPQDQSAFPLYCLVMLDVYTGWLELAPLYTKEAKE

VAQAIVDNWILRHGPFCELLSDRGGEFLNDVMGSICRTLNITHLVSSGHRPQTNGCTERINQELVRKLKI

WAEEFGGEWWRALPVVQHALRVIPRRDCGFSPFELLYGRIPHTMLDQMLQDVLSQVKPEVDDYYIKLKRT

LTKIRERFASVRSFSQDRREEEWNDESRVEDFPLGSHVLYYRELGDMRGGSSKLVRRWHGPYVVVKKLSA

VNYLLADSLDPGSVWLDSFVAHTNYMVDCPEDMRGYYEKRYSLPPPADFWDFNVIPDSVGVGTILLVAGS

TDDRKVWHVGRVIERHADNGRLLLHLHDLPKDSKAPWHGPFRPSYRHRLNGGVFVTVGKVNGRTFRMRVC

EVLGNNIMHVDALDSRGYLRASAVADLEIYGLHSGRSPTTSPL

>XP_642518.1 hypothetical protein DDB_G0277701 [Dictyostelium discoideum AX4]

MTSRTKKLKMDEEEILKKEDGSETTSEEEKEEVEEEEEEDKKRKLVKKTPAKKAPAKKAAAKKKSKDEDE

DEEEKEEEEETNKTTASVSIAIDNLDEPKVEENQMKIISWNVAGFKSVLSKGFTEYVEKENPDVLCLQET

KINPSNIKKDQMPKGYEYHFIEADQKGHHGTGVLTKKKPNAITFGIGIAKHDNEGRVITLEYDQFYIVNT

YIPNAGTRGLQRLDYRIKEWDVDFQAYLEKLNATKPIIWCGDLNVAHTEIDLKNPKTNKKSAGFTIEERT

SFSNFLEKGYVDSYRHFNPGKEGSYTFWSYLGGGRSKNVGWRLDYFVVSKRLMDSIKISPFHRTSVMGSD

HCPIGVVVDLN

>XP_004349623.1 exodeoxyribonuclease III, putative [Acanthamoeba castellanii str. Neff]

MTKTTMTKRARKRRKKSSKDSSDSSSEEEEEEKKPKAKKSKKTAATGNWAPPVTDDDSKFMKLISWNLNG

LNAVHKKGHLVKYVRDEDPDVICFQETKTQTSTAIAQLLGNDYPHEYWNHSVNKLGYAGTATFSKVKPVS

VTYGIGIAEHDGEGRVITTEFDRFYLVNTYIPNAGQKLERLGYRQEWNKDFLAYLKNLEQTKPVVWCGDL

NVAHHDIDIANPKTNQKTAGFTKEERRDFGQLLESGFVDTFRHQNPDLQQFTYWSNRFNCRAKNLGWRLD

YFVVSKDFLPECDKSFVRPNALGSDHCPIGLLVRKAE

>XP_004336355.1 exodeoxyribonuclease III, putative, partial [Acanthamoeba castellanii str. Neff]

TPGEETPAKKQRKPRTRKASGAVGEADGAELVAVDTTTTTTTTTTRTKAAMTAATTTTTTTIKSKKKPAN

PVISRWPLRPVLADQHKYVKLISWNVFSLNAIVEKGSLQHYIKEEEPDVLCLQETKLTNSKIELFKGKTF

HRPDIYQYEFHNCSTAIKGYSGVAMYSKYRPLRVHHGIGVEEHDNEGRVITLEFAGFYLVGSYIPNSGDE

LKRLEYRQRWNRDMEAYLLSLSTSGLKAGLDVHPHGHELEYRVLDESERKGKPVIWCGDLNVAHEEIDLH

DPANNHYTSGFTDEERDDFTRVIKTMDFVDSYRHENPTRQSYSFWSYRSAARARNMGWRLDYFLYQRALT

PYVRKAFIRNFVLGSDHCPVGLLLDPALFEVAKE

>XP_650532.2 exodeoxyribonuclease III [Entamoeba histolytica HM-1:IMSS]

MKRSCKTAKKKLDEEEDKIEEKEVHNKKKGKDDQLYDIEKDNDHYWKEHDFEVFTEDFEKGKPEKANDEI

KIATYNVASWNAAMKKGLNEFIKEENPDILCVQETKLQDGVNPMIDGYHCYFSASTAKKGYAGTAILSKE

EPLSITKKINGKENEHGRIITVEYEKFYLVNSYVMNSGQRLENLVKRTTEWDKDMREHLKALQKKKNVIW

CGDLNVALRWIDVAKPMTRLRCAGFTKEERASTNETIKELNLVDTFQVKYPKKRDFYTFFSFKDKSKTAG

WRLDYFFVSKDLVDSVTQIYRRKEISASDHVPLIIHIKK

>XP_013756823.1 hypothetical protein AMSG_12001 [Thecamonas trahens ATCC 50062]

MPRKRKAEAEAKDKSSPEKRGKTLGGRRALRSGAGGGGEAAAGGIKSGKGSKAGKKGKVGKAGSGADRPM

SEDQKRRAEATNSELELEAIPSAQQAEGARTVKIVSWNINSLKAASRDGRLADYVASEDPDVLALQELKL

ASEAVANEEAFAATVAAAGLGHFPHAVWVPSANKGQSGVALLSKIPWLNVTTKLEMGGHELESDGRFVAA

EFDDFIVVATYTPNAGPKLVRLGRRVDVWDAALGAYLEQLREAAPGKAVVWTGDLNVAPFAIDVANPEKK

ESQPGFSPAERDSFQAICARAGLVDTYRKLRDTVADDHPLAAVMDAQIYSFYSYRVRGARPRNIGWRLDH

FVVSEHALPRVHASIIRPASVVGSDHLPIVLNNLGEWELKQERAFTRWLNAVLACRSLEVTDLARDLADG

SILINTLEILMGAPMPKHRKNPTSPMHQRENVALALDVLGKEGVVVSCSVESVRNGDRKMVLGLVWSMIR

HFHIRADGVSVSKTLLAWINEELHGYACCPVRNFTTNFQNGMVLCALVHHLNPSLIDYDALDPNDAEGNL

KLALQKAHDAFDIPIMFDAADVSHKPDKLSVMTYVEFFRDHTRSREEVRHIFDYYDTDGSNIMDEAEFRQ

YLVDCGYDVTDEAMTAVMSLLDRDNSGTVTFDEFLSWYTSPNRDEMLETTGDVRRRWGERPELSTAVREK

AAAGAAGAAGTGADRSTDTVAETASNGSGIGLASPPASPIKAGSSSRMASPSPARRSRLNSSGHSLNLTN

SRLHAALARARSPMAPERERSISSLRGRSRSPSISSLNRSMPDHAAGQIDPATRSRLTQRRGSLPLGAPL

RAPPHQPDECRRCKQHEREIAKLRRQLQSSETRIDELASAVDETNAPASTTFALRQSGMVLEPELMRQLD

TANSKLASVQAEYDALAKAHKTLEREVTEAQAAHEAEVAEYNDKLAVAANAFNVVKEQWSAQQIEHAAEL

EERSKLEGKLEKALNKSRAARAELEAQLEATRAEAESAAKNATDALAAETTAREDHNRKIADLSTELKNC

QMSQEAAQAELASVREELRSEEQVRSKSEVELRVLREQCDEAKAELAGVLKSASERKERIKQLEAELDAS

YEERSRLEAKVEELAQVETKLVATVAAQREHGSMQKAVQGDLAAVRQELQRTEQARSQLEVEHRVLREHH

ESARAKLASTEASAKDQVAELERVREEVGLLCQQLQGKDAEVRVLQSHQAEARAELERMQKSLDERADRV

AALEAKLDNSLDDRTELQQQIAELQVQAREAKSRAAAAEARAIELEDEAEATSMALEASTASLEESSQLA

RTEAESAQRELRELRARIESLEKVRQADAARAEHEAEARLAAERKLEHAKAECEAAEQALADRVARVDVL

EARVDASLDERNELQQQIVELKAAVRETGAAKDADAAHVAQLQAAAQAAEAARVRGHDELTRTRAEYKAK

LEAAEEAHGRIVARAEREEAARLEAEARLAAAASEHAETQQRLERAEAERRAAEASLEERLARVETLEAK

LDTSLDERQSLQDKVAELQSALHTAQQDARVAEARVIELHEESEARVAALEASTASLEEVSRVARSEVEA

KLEAAHAEVESLRGKLATAGETHAETQRQLERAEAEQRAAEASLEERLARVETLEAKLDTSLDERQSLQD

KVAELQSQIREAEKKTLHLEAQQASSEELIAMVKRDRADVVTRLEAEIAALKGRLAEQEHDAAKVQTKMD

ALEAELTSERQAHAEVANDLRASVSEREAELATRRTECSELQLQLEALQREIAVTEDAAKHAADELDECR

AELEAVQLARSTAECKVEELASELRQSLAAAADAKTELAGCRQELADAGEAQKAIAVRLAEAEAELERER

AAHAHTLEQQTADVALLESVRAEKTELVRERDVLKLELGTLSEARTRDARVQQGVREDLEQGLAEAQAER

ATVEATLGKRLAAAEQASEAASRERDEAKAELATVSVLMARETEAHGETKAELAIIAERARELQTEVSST

SEKCEQIARERDVARVELATTTEARERERRVQDVVRKELEEALGKAKARCETLDQERQDLERRAIELEVL

AASAANAAAVAEADRDQRVAENEVEVKAVREQLIAEKETEIKAVREQLVAEKESEIKAVREQLVAEREAS

VQAVHEQLVAEKETEIKAVREQLVAERKAEMKAMREQLVAEKESEIKAVREQVVVEKETDIKVVREQLVA

EHRAEHEQLVAEKETEIKAVREQLVAEKESEINAVREQVVVEKETEIKTVREQLVAEKESEINAVREQVV

VEKETEIKTVREQLVAEHRAEHKQLVAEKETEIKAVREQLVAEKESEINAVREQVVVEKETEIKTVREQL

VAEHRAEHEQLVAEKETEIKAVHEQLVAEKESEINAVREQVVVEKETEIKTVREQLVAEHRAEHEQLVAE

KETEIKAVHEQLVAEKESEIKAVREQLVAEHKAAHEQLVAEKESEIKAVREQLVAEKESEIKTVREKLIT

EHEAAREQLASEKEAQIQALREQLASQTRVTALAEDDLATASRRLETAVRSLDATSARVTKLEAKLDAAH

DARSKQQAQIAALQDELQAAEQARIHAEVSASSAAERESVLAEEQTAHRAHVNDALAKAEAKIAELTTQL

ERGAFLETQLSSTTKAHKSLLAEYEAFKAEHAVYADVQAKLETALRTKNAEWARQYEALMGEHSHLGLKY

DAHKVRYMEIKHKYIAARDRIMEAVRTAENRKDAEYAEQVHTALDSVARAEADRAQAQADARRFSGELGD

ANDTIARLTADLASYRHKAETAHARYETLLAKLIHYEKHVVPELKLEVQAAQVAVVEEAKRKDVNHTYLD

KVIHDKHALERQLADAKNTIALFEAHQPASPLARKSRERRPAEESGGPSRGPDGPASLEKPASPKAPSQM

TIDTLKTQLRNERAEHAKAKALVLSLQHQLAVAVKHVSAASSSPLAPTPSLPSLKRTRRRVRRTHSAAE

>XP_005840555.1 apurinic/apyrimidinic endonuclease family member in base excision repair [Guillardia theta CCMP2712]

MPPRRKASAGLKAKKSAEDGLKGQENPEEAAADEETKVPQKRGRGRAAKEKVGKDQIADNKGDTQDEKKQ

AEPEKVTKKRGKKETDTAESDKPKKKAKDEQETNKKEKVVQEKVAIEKSDIKHAELPSKRYKIVSWNVTT

IRSLMKKNPELLKKLMEQEQPDLICFQETKLKQEEHDEYEEMLKSQFPGDQGYEAHFSSSLSNKSYAGTL

VLVRSSKDKATAQKQTKMDSFFKGKDSKSAKKDEETACPPLKVTFGLSTLSKDDGEIAAEVIQEGRTITV

EFESFFVVATYVPNSGEKLQRLEFRTKLWDRKVVEYLKKLEASGKPVIWCGDLNVAHLDNDIWNVGAKHL

VKSAGTTPEERDSFSKILEEGYVDCFRHLHGEDAKGWFSYWSVRAGNRPWNRGLRLDYFVASKSLCSQDS

LPRVVDADIHDEFVAIDHAAISLTLAV

>XP_005838854.1 hypothetical protein GUITHDRAFT_65587 [Guillardia theta CCMP2712]

MPGVRRLRCSRDPSGQKNLRLVTWNVNSVRQRLPLIERYIQEYSPDVLALQEIKCQQNDFPAETFSKLGY

HHVHLGQKSYNGVATLSKHPMTNVEFGLKGPEDDEHDPQARYVQCSVQGIAICNLYHPNGNPVGSEKFDY

RLRWSKLLYGRVKSLTQSDMPLVLMGDFNTVPRDEDCYDIIDLSSDAVVHPHCRSQYRSLVYQGLYDALQ

IKPNKEKQYSYWDYRAGSWEKNKGMRIDHILLSPHLADRFVQGGVHKEVRGWQGPSDHAPVWADIRRSDA

MSD

>XP_816327.1 apurinic/apyrimidinic endonuclease [Trypanosoma cruzi strain CL Brener]

MSLSPPGAKFFFFFMVSFWILRLFLCVCLCVGFICIDCATCTRLVCTAVLCSASLSFNLSIHCFCGLFAA

CGRSQGHRAATAPFILLCIFLFLFLFCPHVSFVMCVSIVRLCGAVSVMPSGPKEQKPVAAAGGKRTRSRS

PSATSPKKPATRSTRIRTPTPPSRSLNSAGAEATSPNRPLAAVLTAPPPSDDDTRKTEKDIWSQVEPFQR

RTAAKDFDSKHMLKFITWNVAGLRGLLRKDDQAIQRLLEEEGPDALCLQETKLNPDDPQNEKLGEVPGYR

FVDHVCRAKKGYSGTRTYIKNTAAAEWKTVTVKGFDTLKSPQDVGHSEGDEEGRVLTTYFGTQGKGSETF

ALALVNTYIPNSGMSLERLPYRCQKFDLRIRQHLCTLGRSCNHDKEEGDAPSLAGFIWAGDLNVAERDYD

RYFAGSYKAMQKCSGFTPEERASFRETLRVANAVDTFRALYPKAAPVYTFWSARINGRARGLGWRLDYFV

VSAALARHVVDCFTMPHVMGSDHCPLQMWLRR

>XP_001707172.1 Endonuclease/Exonuclease/phosphatase [Giardia lamblia ATCC 50803]

MPPAPVKMKKTSAAKERPPPPPYSTFQIGSDEKTEPVPFTILAINVNGIRSAFSKGLKDFIHKKNPDIVC

FSETKLGSSAFAAFMEKEATRDPDTGVYTVMKGYKHVFCCSTAKQGYASTAMFAKESVPVLDLCMQMGDP

EFDSEGRFLHVSLPSFELIHVYAPNSGRGSSGKAFTSKNRPANLSTRIKYEKLISKYIGNLVAAGKDVVY

CGDLNVAHNEIDLYNPKSNHFSPGFTDEERNAFSKLLDGHGLIDAFRAMHPHRINYSWFSNFGRAREQKH

GWRIDYFVVTSKIFKRVTTVDILDDYNSYSDHVPIIMTLTGI

>XP_005762479.1 hypothetical protein EMIHUDRAFT_216212 [Emiliania huxleyi CCMP1516]

MPPKKKADQQEEAPAAKKTAAAASAIAANASKITTSAKAIAAGKADMIERTPTPRKPRAESGLVLVHWNV

GGLNGLLNGKNADERKALLSALVEAEKPDVLAISEHKLQQKNVAAAEAALLGLLPGYTAHWAVCTAKNGY

SGVVCLVRSGVEVSSVSLDAVESINEGRTVSIEFPDCHAVAAYVPNAGQDLKRLDYRIDTWEPAMRSYLQ

TLQASGKPVVLFGDLNVAHLDADIWNVTAKHVPKSAGCTPRERAAFGALLASGPFVDCFRQLWPDASGAF

SYWSTRSGNQLLNRGLRLDYAVASASLAAPGAALSLHDCAYLAEYAPNGDHAPSLVALARTAAA

>KOO23530.1 apurinic endonuclease-redox [Chrysochromulina sp. CCMP291]

MPPKYKANAETASASPSKKAKKAIEEEEEAIAAGTADAVTRTPTVRKPHVEGGLVMVHWNVGGLNGLLTG

KSAEERKALLKDLVDKERPDVLALSEHKLQQKNVAAAEKALLELLPGYAAHWCVCTAKNGYSGVVALVRK

GLAPAVALDEVCPSLHEGRTITLTFDDVVAVLAYVPNSGQDLKRLDERIDTWEPAMRAYLKAKSSATGGG

PVVLLGDLNVAHLDADIWNVAAKHIPKSAGCTPREREAFGVLLADGPYVDCFRQLHPDAQGVFSYWSTRS

GGQLLNRGLRLDYAVASAALAKEGGPLTLHECAYMSEYAPNGDHAPTLVALKRV

>XP_002676078.1 predicted protein [Naegleria gruberi strain NEG-M]

MKRKQETKNTKKLSSSSNEGPTKKLKQSNLLSLFKAPSSPPASSQSSTDTDSLTIVEESKTFLGTNNSYE

LNKQFKFISWNVNGISAVFRKEPNYLKMLVEKEEPTCLCLQETKLSEDVKLSEMAQYKFKNYIGHFNTSR

ARKGYSGTACFVLKHGPKVLNVKFGIGKDKHDLEGRTITVEYEDFYLVNTYCPNSGQNLDRLEYRTKEWD

VDLLKYFEKLEKKKPIIWGGDLNVAISEMDIHDPLTLSKSPGFTLEERQSSPLVTKASPPFIDSFRYKNP

YKKQYTYYGYINDMRLQKKGWRIDYFVCSNSLKDKIVNSYILEEYFGSDHLPIGLIINKE

>XP_001299171.1 exodeoxyribonuclease III family protein [Trichomonas vaginalis G3]

MSTTQTDAAPPLKISTINAASLRAAWGKGLHDWALASKPDVICIQETKMHETSQPNIGAFLLDGYQGYFF

DCNKPGFHGTAIYTKIKPISVKESFPDDEGRSITMEFSNFYLINSYVPNAGQKLEKLTYKIETWNPHLKE

YLDELSKTKAVIWTGDLNVAHEEIDIFDPKGKDKTAGYTPQEREWFHKFLESGYTDIFRKRYPDKKEFSF

YTYRGQAKAKGNGWRLDYFIMDNAHYKDELVNDCAMETGDFSDHVPVSLFLDRSLLAAEDVAVDKTFNKR

LNNDTIVDTPEVVPEKAKPKKGKKDQPTEEEPKEEEAKEEAKEEPKKEESEEKPKPKPKKGKQAKEEESK

PAEEAKPVTEEAKPASEESKPAEEAKPAEEEKPKAAKPKKTKKVVEEEPQEPEDEVDDEEEEEDKPKKKR

RGKKGEEQSLKRMQTREKRKTIYFSDDDSDDSTEYTGTPRKTRRSKK

>XP_001324898.1 exodeoxyribonuclease III family protein [Trichomonas vaginalis G3]

MDSTTAQVIKIATWNVASLRARWKDNFTFYINSSKPDIICIQETKFHVDMKESLKNFKLDGYKGYFFHAK

KAGYAGTCIYTKYKPVSVKRSFADPDGRCITMEFKNFYLINTYVVNAGEDLGRLDYKIKEWNPKIRNHIM

ELEKKKPVIWTGDLNVAHKPIDIWQAEGHEKIAGYTDEERKWFDDFLNEGHIDIYRELHPESHEFTFFNY

RGQAKSKNQGWRIDYFITGKGNIEKLGISDCVIEGTIDGSDHQPVILLADKDKIMKDDEPVTSSEVEMLT

AGNIKSFFG

>OHT00257.1 exodeoxyribonuclease III family protein [Tritrichomonas foetus]

MGRPKKGQNTKKNAPQKEENHDDEIPNENNHENSNDNQNESLENENDPKKTNIDTKTVEIAGDVLKIITC

NVASLASAWNKGFYDYIQVDSPDIICIQETKLHAQSKTPFTNFILDGYYGYFLNSEEKKGYAGTAIYTKL

KPLSVKPGFKDSEGRVIQMEFSKFFLVNSYVPMAGMKLERKSYKVETWNPKIGNLIEELSKTKPTVWCGD

LNVAHMPIDIYDTKGKDKVAGYTPEERKWFDDFLKKGFVDVFRHLYPDKQQFSFYSYRFDMKAKNRGWRL

DYFIVPQAAIDEKLPVDCSILSTDFSDHSPVVLLLDKEKVVTDEDLPVTEPGITILNSGKTFTKNNTTTN

ESDSSQKKKKGKTMTMDMFVKPKESKEDENKEEQSKEEVNKEDESKEEGNKEDESKEEKSQEEGNKETDS

SATETRSSPRRSKKENTNENQDTVSKEGGDKKNEKKAAPKKGNAKKATRKKK

>OHT05750.1 exodeoxyribonuclease III family protein [Tritrichomonas foetus]

MSEESQDFSSQISQSTQSTQLTQSTQSTEINAEHSQAIESTQASQTESNHVPSDLLKITSWNLASLNSAW

EKGLRYYIKAAQPDIFCVQETKLHDKSEHPVSYYKLPGYHAYFSHAEKKGYSGTAIYTKIQPISVKISDG

ISDKNGRCITMEFSNFYLLNTYVINLGQDCQRKHEKLEVFNPEIEKHIQTLSKKKPVIWTGDLNVAHMPI

DIWTTEGHESIAGYTNEEREWFDNFIKNNEYVDVFRELYPTKQQFSFFNYRGNAKGKNQGWRIDYFMMPK

SMIKEGLIVDCTIENGDLSDHEPITLLLNRSMILSDADKPVEMTTCEILGKKTKSLLNFFAAAPKKK

>OHT13391.1 Exodeoxyribonuclease [Tritrichomonas foetus]

MTEETTSIVDVTSDIFTIITWNIASLRAAWKKGFKDFVKLHNPDILCIQETKMYNGCKPPISDFVIEGYH

GYFSHAAKKGYSGTAIYTKIKPKSINKTPGITDTNGRCITAEFNNFGLLNSYVPNAGEGTLKNLDYKLIN

FLPEYKKSYTETDDKLSRPLFLLGDLNICHKEIDIWDAKNKENIAGNTPGEREWLNTLFTDNNYYDIFRD

LYPDKRQYSYFEYRRRARETGNGMRIDYFIAKREKIPKGMIVDCYIGSANDISDHDPVFLTLNKSLAISS

DDIVETDYANESLQ

>CEP01813.1 hypothetical protein PBRA_008755 [Plasmodiophora brassicae]

MRILQWNVNGLRALALKGTTLKDVVAREDPDVICLSEVKCREADNPLKLPGYTCFFHESKLKKGGYAGIA

MYSKSKPLDVIRGIGVADDEGRAVAIELEKLCVMCIYVPNSGSKLDRLGYRTTTWDKRLREFVAELSADK

KKPIALVGDLNVAHLDIDIHDPIRNRNKSAGFCDSERENFSLLLKECNLVDVWRHKYPHATVEGYTYYSH

RFNMRAKQKGWRLDYVLLSDQIIDAVSEIRVLQETGSDHLPLVVDLSF

>XP_005706795.1 exodeoxyribonuclease III [Galdieria sulphuraria]

MTKRSKNEQSMELSEKPKKETRKESKKESVEFLGSDQERQKRFQDPGESLLDSENLDGITIPQRRSTSKI

FITWNVAGMRSTVKNPLFLGFLSKLGPDVICLQETKLSDKGQVSEEVTQSLSDQYHDIWNHCTARKGYSG

TAIFTREEPLSVLYGLQEEKHNEEGRVITLEYEKYFLVNAYVPNAGEGLRRLNYRIDSWEKDMCQYLCGL

NQKKPVIYTGDLNVAHQEIDIYQPKGHEKHAGFTPQERQKFTDLLQCGFVDAFRYLYPHRQSFTYWSKRA

KAKERNHGWRLDYFVTSERLVPCILECEMFENIYISDHCPLMLHLDIAQLK

>XP_005703984.1 exodeoxyribonuclease III [Galdieria sulphuraria]

MYTCLGEKRFINDTEDPSDPLSPTKKKNCTQLDSITENSPKKDTETLLKFLSWNVNSLKRVLERKQLVNL

VSQEDPDFICLQETKLTEDKVPGAFLLPQYKKFFNCCETKRGYSGTALFSKMAPIAVLKDFEGVYSEHNK

TGRLIVAEYENFYVASVYVPNSGDKLKNLEYRVHSWDPSFCSYICELQMKKPIVLLGDLNVAHQDIDVYA

PERLGSKAGFVQSERNNFSKFLEETKMVDSFRYLYPHRKEAYSFWDYKTGGRLRNQGWRIDYCLLSSSLV

SNLVDAFILDKIEGSDHCPIGIQMRIF

>XP_005717900.1 unnamed protein product [Chondrus crispus]

MSRSLAAFGLVPPGALARGGRSRVSNFVTSPRCCTSLAGTHSSAITWNVNSLRALMRKNPNALQDLVEEH

NPDILCLQETKLQEIHEGLFADALQGYESSVFHSSRARLGYSGTAVFAKREPQCVQFNIRHEDGDLEGRV

VCLEYPGAIVVNVYTMNAGQNFKRLDQRMTWDSAFRRYVRNLRREGKPVIVLGDLNVARDPVDVHSPKTA

KRLPGFSDEERQSFEDTLEVCGLVDAYRMLYPDEKEKYTFWDYKSRARDRNMGWRIDYALVTDDMVRAVK

DVHILDHVEGSDHCPVSIQFTPGLLW

>XP_005716978.1 unnamed protein product [Chondrus crispus]

MKSPRKRKRPLSDSQRTTEKIAKRNHRPAKPKDDKKDVDENEVKVGDQAISDAMRNNCAAAAEPENAVEN

EGAPDRPWGALDDPHKLKVITWNVASCRSMIKNGSLLRYIQQEAPDILCLQETKMTDKAVKEFPEVAAYD

VHWNHSEKKGYSGVAILVRRDLDERKKVRVKRVEAGIGLAEADKEGRVLVCYLSSGAAIVNAYVPNSGQK

LARLEFRTKEFEAGLRKFLDDLAKEHRVVYCGDLNVAHNEIDIHNSKANRKNAGHTPEEREEFSKLLSSG

EGWVDSFRELYPSVPGYTYFSRRFGAKLKNEGKGWRLDYHVIDKASFKSGVVGDVYVRTQVEGSDHYPVV

LEYRFGESG

>XP_008910806.1 exodeoxyribonuclease III, partial [Phytophthora parasitica INRA-310]

MPRQSMRLKVAAEKAVASVTASPAKKKRKSVTAVKVAAEVAASASSLKPKKAKTSSSDNKSAWGALFSKP

NKQERARPLVAEAISPELQAKIDAFPSFQDVSRRSTDDIDTKIIAWNVNGLRAVLKRDESVHLRAYVAQE

DPDIFCLSETKICRDELQKLEDFLPQYEHQYWSCAIKKGYASTAIFSKTKPLSVKDEIVVGDHDSKGVTK

SANGGKDQEGRFLALEFPKFWLVHTYVPNAGGKLERLDFRTDQWDKAILREMKEMEKTKPVIWCGDLNVA

HQEIDIHNPKGNHKSAGFTDEERESFGNILESGLVDTFRHLHPDKVEYSYFGYRHNMRAKNKGWRLDYFV

VSEKLMPHVRSSYIRQSVVGSDHLPIGLELGGLS

>XP_002181718.1 predicted protein [Phaeodactylum tricornutum CCAP 1055/1]

MKSCIQLGHFRQSFRGKKSCGWVGLSAFSLNTFALFSRGTIALCPPLSAAPYRIRPTSYRRLYVTTALRM

APRSSKKEPDAASGTPNKHDRVTRGSTKTPPSAASIDSQKTSEAPLKKSAKRTLPEDTTKENSPKKKAPT

HQVLTERDDIPRLWSDEQAAKNGSYTMRIASWNVAGLRALMRNSPHALSDFVREHNVDVLCLQETKLQES

HLDDPKLKIRGHLLEKEGFDSYYSCSTARKGYSGTSVFVKRRQLIKGSKVAKKQKTLGSYFGKNDERETS

SNSLKGTEELSIDPHLLVPEGVSFQMNVDKHDSEGRIVVVDFPSFTMCNVYVPNSGQKLERLSYRTEEWD

KDFLSFIQKKQKDRGVPVLWLGDLNVAHTNLEVWNDGAKHLAKQAGVTAEERASFEAQLNAGFIDAFRRL

HPTAKGHYSYWSQRAGNREPNKGLRLDYFICDPSLFDEESKTIVRDSYVLPLQQGSDHCPVVLELEIKA

>XP_002292842.1 predicted protein, partial [Thalassiosira pseudonana CCMP1335]

PIDVATELGMSAHDGEGRTITAEFPLFYLTNVYVPNSGQKLERLGYRTNSWDKDFLTKMQQLEKDGDKPI

IWVGDLNVAFDEKDTWNEGAKHLAKSAGTTAEERASFAEQLGAGYIDAFRHLHPEGRGHYSYWSQRAGNR

EPNKGLRLDYFVCSKGIMGDGDGTKAIVRDSYMVPDQLGSDHCPIVLEIEIKK

>EWM23176.1 apurinic endonuclease-redox protein [Nannochloropsis gaditana]

MLLALLTLPVQLAFMLNSPTIIHVFGLNTCVNLVSGLCFLPGPLSRGCSSKALMTSRHSSSRFRESFGEF

SSTVRTQKWKSSRSISTRTSTSVFSSSGASLHMSPSEPEGARDPLKKRGRPAKGPKPSAQDAAMVAGAES

SARRVPLHGDQTRKEVASVGRGREPRSRKAAASPEAMNGTDPSSSTLSPPKKARKGRSRKGDDAVSQGTA

VSTERLKDAAAPTSAGSSAVPKSKRPPLQRATIPRGLTPRLEDGPPVSSSSSSSSSRPRRRLRLLSWNVA

GLRGLLKKGETAAEAFRSLLLRERPDVLCLQETKLQEEHVAEVAAALRELHLPTFEGNWHCSTAKKGYSG

TSVLVSESAGLAVGPGGVTCGMEGEGDGEGEGRVISVDLGAFTLVNVYTPNSGEGLRRLAFRTQAWDRAF

ERHLEALQGAGDGNRAVLCCGDLNVAHLDADFYNPGEARMAKAAGTTPEERASFGRLLEGAGMVDSFRHY

HPDVTGCYSYWSMRAGNRPYNRGMRLDYFLASKNMLRGGDEGGGVPRLLDSFILDQDPAVSKLSDHAPVG

VLLEV

>EWM28136.1 Endonuclease/exonuclease/phosphatase [Nannochloropsis gaditana]

MSKLKRIKLKSQDPVEIVKVRDTIDDRNNDDAEEEGTRQLLNGGVEGKEGDKTPPPDDCERQLLSSSKQV

TELVLQTERDLLARNSLPEKSFKVLSWNVNGLNGILNGKAAQINGNKGSNVLQQLVQEEGNVDVIFLQET

KLQDAKCAAFEDVLPGYVAHWSCSKTKKGYAGVAAFVHEKYVKRCISSYSDANKGSSRGKQATLASFFPT

KGKETEPTKGAEGRIAAMKTAACKSASMASSCSIKDKEIEDGDSSVEALQVLSVRRGCDGEDDEDEEGRV

LTLEFGSFWVVGVYVTNSGMKLERLEYRLKQWNPSFQAYLARLNAVKPALVVGDMNVAPEASDVYNAGAK

HLAKVPGLTPEEREAHAAWLRTGWKDTFRALHPDARGAYTYWNVKTRARAENKGLRLDLAVIGAADMDGG

RGVRVRDSFILHEATIGFSDHAPIGVVLDLGA

>CBN76988.1 ARP; DNA-(apurinic or apyrimidinic site) lyase [Ectocarpus siliculosus]

MTKARGIATDEMTEATDGACKAIFLEHDGKWVSCDKTLGMTEQQQNLTKLQLEGLTVPELRHEARRRGQT

ATGSKKDLIQRLFSQEAPVPKRPRRRNHGKPVDTPEGYVERQTTTRPRPLHPYTPQLRVLSWNVNGIRAQ

MDKEEGRKALQGIIAQERPHVLGLQEIRTSATTSTPPGKTRRKKTPPQTSNFADIVKTILPDYDTIWLSS

VPPARQGYAGTALFVRKKRSWDIGCPKLLGVRGGIGHPEGDLEGRVTTAELDVAFVVNVYTPNAGAGLKR

LDFRTKDWDQAFAAYVRDLEKIKPVVVVGDMNVAHEDVDFYNPEQKRTRKAAGTTPEERSSFAVNLLGCH

PPPSCAAALAVDTTSAADDSFRSSAASSGGGGEAGGGGDDNKAEGSGCSLVDTFREKHPKVTGVFSYWSV

RARNRPVNRGMRLDYCLASRGLVGGDGVHDAFVLDRDTVGVSDHCPVGVVLRLGDHYPE

>CBJ48391.1 conserved unknown protein [Ectocarpus siliculosus]

MSASQEESQGMTTAAEESQDTPGDSFDDTQELKEQEEEEEGGGKKKKGKKKKAELPIFTKRDPIDRVPFP

STGTTFTVLSWNVNGVRATAKKGLEPLRRMVEKEMPDIVCLQETKIQEKDVEALRAQDILPGYASEWSCS

RTKLGYSGTAVFFTKQSDPWKGDGGGIDATGNGLPSDEPARKKLKQGKISTFFSPKPSKKSAKGSAPESE

TPTRAPAAEPATSAAASTNSADSLPGGRSGFKVLSVRFGIGGDSKHNNEGRSITVEYEKFFVVTVYVPNS

GEKLVRLDYRTKEWDVALKAYVESLEAQGKPVVLNGDLNVAHLDLDIYNRGAKHLPKNAATTKEERDSFD

SWVNGGKVSDAFRRLHPDAEGAYTYWSVRTGARPVNRGLRLDYFVCSNAVFEDGAAGGGQAKATARRAKG

SAPKGKGATGAKGMVVHDCFMLDEATVGVSDHCPIGITLRST

>NP_181677.1 apurinic endonuclease-redox protein [Arabidopsis thaliana]

MNNVLQFGLQSSAIYVAKFLVVPLRSLRVGSSFVGVGVGTRSFNKRLMSNATAFSINNSKRKELKIPGAA

IDQNCHQMGSDTDRDEMGTLQDDRKEIEAMTVQELRSTLRKLGVPVKGRKQELISTLRLHMDSNLPDQKE

TSSSTRSDSVTIKRKISNREEPTEDECTNSEAYDIEHGEKRVKQSTEKNLKAKVSAKAIAKEQKSLMRTG

KQQIQSKEETSSTISSELLKTEEIISSPSQSEPWTVLAHKKPQKDWKAYNPKTMRPPPLPEGTKCVKVMT

WNVNGLRGLLKFESFSALQLAQRENFDILCLQETKLQVKDVEEIKKTLIDGYDHSFWSCSVSKLGYSGTA

IISRIKPLSVRYGTGLSGHDTEGRIVTAEFDSFYLINTYVPNSGDGLKRLSYRIEEWDRTLSNHIKELEK

SKPVVLTGDLNCAHEEIDIFNPAGNKRSAGFTIEERQSFGANLLDKGFVDTFRKQHPGVVGYTYWGYRHG

GRKTNKGWRLDYFLVSQSIAANVHDSYILPDINGSDHCPIGLILKL

>NP_566904.2 DNAse I-like superfamily protein [Arabidopsis thaliana]

MKRFFKPIEKENSPAAKKPCLSPEKRDGDGDGVEEEKNQNEPSKFMTWNANSFLLRVKNDWSQFSKFVSD

FDPDVIAIQEVRMPAAGGKGKPKNHEELSDDTKVLREEKQILTRALSSPPFGNYGVWWSLADSKYAGTAL

LVKKCFKPRKVYFNLDKLASKHEPDGRVILAEFETFRLLNTYSPNNGWKDEENAFQRRRKWDKRIVEFLN

KTSDKPLIWCGDLNVSHEEIDVSHPEFFATAKLNGYVPPNKEDCGQPGFTPSERGRFGATIKEGRLVDAY

RYLHKEQEMESGFSWSGNPIGKYRGKRMRIDYFLVSEQLKDRIVSCKMHGRGIELEGFHGSDHCPVTLEL

SKPSSEMEQNQVSN

>XP_010273319.1 PREDICTED: DNA-(apurinic or apyrimidinic site) lyase, chloroplastic isoform X3 [Nelumbo nucifera]

MGISSNTEKSAVKENTARNQVVEIQTIRDDPARLKAMTVRELRTVMRSAGIPAKGCKNDLVSTLKNFLTN

ENDGQSSVVREEHGTSMIKSVCSETVSSKQKRNNSYDEDQKQNVNAVSEISGIKRRGKAPSVGTGVEVKK

KRVMTKQKLSVKVDAVAGKKPSRASRQSLQICNEDDGTIDKVDVSINSSEPWTVLTHKKPQQGWIPYNPR

TMRPPPLTDDTEFVKILSWNVNGLRALLKLEAFSALQLAQKENFDVLCLQETKLQEKDVEKVRQCLLDGY

ENSFWTCSVSKLGYSGTAIISRIKPLSVRYGLGISDHDSEGRLVTVEFDKFYLISGYVPNSGEGLRRLTY

RVTQWDPSLSNYMKELEKSKPVILTGDLNCAHQEIDIFNPAGNRRSAGFTDEERESFERNFLSEGFVDTF

RRQHPGVVGYTYWGYRHGGRLKNIGWRLDYFLVSESIADKVHDSYILPDVAGSDHCPIGLILKL

>XP_010248774.1 PREDICTED: DNA-(apurinic or apyrimidinic site) lyase isoform X1 [Nelumbo nucifera]

MKRFFKPVEKDGSFKKPALSPSPTTAVTREQKDEKDGEKADERKEPLKFLTWNANSLLLRVKNNWSEFKK

FVETIDPDVIALQEVRMPASGSKGAPKNPAELKDDTSSSREEKQILMRAISNAPFGNYRVWWSLADSKYA

GTVLFVKKCFKPKNIFFSLDRTVSKHEPDGRVILAEFESFRLLNTYAPNNGWKEEETSFQRRRKWDKRML

EFVLQSSDKPLIWCGDLNVSHQEIDVSHPDFFSNAKQNGYVPPNKEDSGQPGFTLAERRRFGNILSEGKL

VDAYRFLHKEKDMERGFSWSGNPVGKYRGKRMRIDYFIVSEKLKDRIVSCEMHGQGIELQGFYGSDHCPV

SLELAPSIDSNKC

>XP_015625662.1 PREDICTED: DNA-(apurinic or apyrimidinic site) lyase, chloroplastic [Oryza sativa Japonica Group]

MPLLLRGGSLFRLYGCGCGLPSANFSPSKLALIRLSLMMAETRATYSRRAASKNTDIKKDDEHVLEKEDV

AESKLEIEQLRNDPDRLQSMTVKELREITRMMGIPVKGNKKDLVSALMDSLGKVGTSSVEKIGVSEVPSK

RKGASVVVEQNIDSSEVISETPSKRSRAKNKGTAEESSGANVKQSKTSVQKKKLVVQGASVDHEEPWTVL

VHKKPQPAWIPYNPKVMRSPSLSKDTKALKILSWNVNGLKALLKSRGFSIHQLAQREDFDILCLQETKMQ

EKDVEVIKEGLLEGYTHSFWTCSVSKLGYSGTAIISRVKPLSIKYGLGVPDHDTEGRVVTVEFNDFYLLT

AYVPNSGDGLKRLTYRVTEWDPSLGNYMKDLEKSKPVILTGDLNCAHQEIDIHDPAGNRRSAGFTIEERE

SFETNFLSKGFVDTFRKQHPNVVGYSYWGYRHNARKTNKGWRLDYFLVSESIAERVHDSYIIPDISASDH

SPLGLVLKL

>XP_015627423.1 PREDICTED: DNA-(apurinic or apyrimidinic site) lyase, chloroplastic isoform X1 [Oryza sativa Japonica Group]

MSAIRASSHRLQTRTVALTRTKMSSMAGLGASQHGYPPRSHEPWTKLVHRERLPEWFAYNPKTMRPPPLS

HDTKCMKILSWNINGLHDVVTTKGFSARDLAQRENFDVLCLQETHLEEKDVEKFKNLIADYDSYWSCSVS

RLGYSGTAVISRVKPISVQYGIGIREHDHEGRVITLEFDGFYLVNAYVPNSGRFLRRLNYRVNNWDPCFS

NYVKILEKSKPVIVAGDLNCARQSIDIHNPPAKTKSAGFTIEERESFETNFSSKGLVDTFRKQHPNAVGY

TFWGENQRITNKGWRLDYFLASESITDKVHDSYILPDVSFSDHSPIGLVLKL

>XP_015618714.1 PREDICTED: DNA-(apurinic or apyrimidinic site) lyase [Oryza sativa Japonica Group]

MKRFFQPVPKDGSPAKKRPAAAAAASASDSDSLGGDAPAAAACAVGEGDSPPAPREEEPRRFVTWNANSL

LLRMKSDWPAFCQFVSRVDPDVICVQEVRMPAAGSKGAPKNPGQLKDDTSSSRDEKQVVLRALSSPPFKD

YRVWWSLSDSKYAGTAMIIKKKFEPKKVSFNLDRTSSKHEPDGRVIIAEFESFLLLNTYAPNNGWKEEEN

SFQRRRKWDKRMLEFVQQVDKPLIWCGDLNVSHEEIDVSHPDFFSSAKLNGYIPPNKEDCGQPGFTLSER

RRFGNILSQGKLVDAYRYLHKEKDMDCGFSWSGHPIGKYRGKRMRIDYFLVSEKLKDQIVSCDIHGRGIE

LEGFYGSDHCPVSLELSEEVEAPKPKSSN

>XP_011627683.1 DNA-(apurinic or apyrimidinic site) lyase, chloroplastic isoform X1 [Amborella trichopoda]

MQAGLASVYVSTSLKFRSRAIGFSEMSRMQCNQRISNLSASLSANFNGRRKSRLKGSVAKESSQAPINNS

EKPGEMGVNLAMDIKVDKMESHEGVQEAEKDLKSFETMTVQELRVLMRKAGIPPRGRKQDLVSALKHSME

SGLGQSSQRLETEGNAASMMETGHHKRATQNRKVKNSSARETRTLSHLSVDNVESLEIISEVSRTHEDDS

HVVLTKNASTTRRKEKTGSMSTDMKPTKVSYKRKAAAREYERVEVANGVGPFMNEQPWTVLAHKKPQEGW

VAYNPSTMRLPPPDGTKHVKILSWNVNGLRALLKLEGFSALQLAQKENFDVLCLQETKLQEKDAEKFKQS

LIDGYENSFWTCSSSKLGYSGTAIISRIKPLFVKYGLGLPDHDHEGRVVTVEFDTFFLISSYVPNSGEGL

KRLTYRIEQWDPSLSNYAKELEKFKPVILTGDLNCAHQEIDIHNPSGNRRSAGFTDEERTSFRTNFLANG

FVDTFRRQHPGVVGYTYWGYRHGGRKTNKGWRLDYFLVSDSIADKVHDSYILPEIDGSDHCPIGLTLKL

>XP_006852280.1 DNA-(apurinic or apyrimidinic site) lyase [Amborella trichopoda]

MKRFFQPIQKDGSSKKPSLSSPEKMYSCSQSPEKTNSGNSIPENAEPLKFVTWNANSFLLRVKNNWPEFT

EFVRRIDPDIIAIQEVRMPAAGSKGAPKNPGELKDDTSSSREEKQVLLRAISSPPFGNFRVWWSLADTKY

GGTALFIKKQFQPNKVSFSLERSGSKHEPDGRVILVEFDSFCFLNTYVPNNGWKDDDSSFKRRRKWDKRM

LEFGCQSSEKPLIWCGDLNVSHQDIDVSHPEYFSNAKLNGYVPPNKEDCGQPGFTIAERQRFSTILSEGK

LLDAYRFLHKEQDLEGGFSWSGNPIGKYRGKRMRIDYFLVSEKLKDRLVACEIHGKGIEMEGFYGSDHCP

VSLELSPVNEANGFKATECGPTEN

>XP_002973537.1 hypothetical protein SELMODRAFT_99746, partial [Selaginella moellendorffii]

KSASPWALLRHKKPKPDWIAYDPSTMRPSSLPSEKNLVKVISWNVNGLRACMKRKNELEEEGSALARLAD

SEDFDVLCLQETKLQEKDVEAIKQKILGGYSNSFWTCSTSKLGYSGCALISRIKPLSVTYGLGISQHDGE

GRVVTAEFDTFFLVSVYVPNSGQRLERLTYRTTEWDPAFSTYLLELEKRKPVIVTGDLNCAHEEIDIYDP

DGNKRSAGFTSEERTSFQTNFIDKGLVDTFRKQHPLAVGFTYWSYRSNARPQNKGWRLDYFLVSQGLVCS

VADSYVLPQLDGSDHCPIGLIIQTEQ

>XP_002993241.1 hypothetical protein SELMODRAFT_187330 [Selaginella moellendorffii]

MKRFFQPVERDGASKKPSIAIANGGENACADPSRFMSWNANSLLLRFKNNREEVMSFIRGFDPDVICIQE

VRLPAAGFKGEKKNPGEIKDDSAAARNDKQAVMRVLSVSPMADYSVWWSLGENKYAGTALFVKNCFKPVS

VAFNIDNKGKDAQRHEIDGRIILAEFGSFTLLNTYAPNNGWKEEENGFVRRRAWDKKVLEFVSSCSKPLI

WCGDLNVSHQEIDVTHPEFFANATQPGYTPPNKEDAGQPGFTLNERARFSAILNRGNLVDTYRHLHEKQD

LEAGFTWSGNPVGKYRGKRMRIDYFLISKALLPRLASSKIHGRGIELEGFLGSDHCPLTMELKNQACQNH

D

>XP_001770524.1 predicted protein [Physcomitrella patens]

MGLESASPTRRKRQKDAAGEVGGEDVANIQVDKSDKKSPKAESRKKTIKKTEAEPSSVGNDKEPWTSLVH

KKPQPGWVAYDPKLMRPKPPAKDEKVVKLLSWNVNGLRALLKEKGAEHEQGSMIARLAAREDFDVLCLQE

TKLQEKDVADIRKSLLASHEVSLWGCSTSKLGYSGTAIISRIKPISVQYGLGIPNHDQEGRLITCEFDTF

YFVVSYVPNSGAKLERLAYRTQEWDVALSSHLRELEKKKPVILTGDLNCAHEDIDINDPAGNRKSAGFTD

EERESFKTNFLDHGFVDTFRKQHPNAVAYTYWGYRTASRPKNKGWRLDYFLVSGSLSENVHDSYTIPDVG

GSDHCPIALILKI

>XP_001757072.1 predicted protein [Physcomitrella patens]

MYHESSTIQAHRQRARYQLLLPQPAGLTTEGEIDCLEYGGKTSRRRGPERTEAGGALKCELADDPTSEVY

NGPRFNPALCRMRIFSWNVNGLRTTLKDVAACHTSASNYFCQVLNADIVCFQEAKIQEEKLEKWIACVDG

YHSHWAFSREKKGYSGVVTYVKEALSPLDAKADWLGENSSAEDLCNEGRLMCTDHGSFVLLNVYVPNSGD

GDKGRPRLDFKMRYLKALEQTCDDLVRSGKHIVIVGDFNVAHKDIDVHSRWKVEEIYSLEEREWLDGFLS

RYIDLYRHFHPDEKNIFSVWDQKSDARTRNEGLRIDYAICNEGFLTEVLETDIVKMQKQWSDHAAVVVTL

KEQPNLPPHPAPALSSCNMKRFIADPRQKKLTALFNKCPNRQVTTIVSDKIVEDEGDELHSKSEMSIDAL

SMQKDVTRKSNTPVCKEHPVFEERGESVKAEVTPATSAVTDRVVDIADPTREAVELPVYVSNSDATMSTD

SSNKESTSQNKGKKLTSLGKRKLEADTKGLNKQRSVASFFKAANAK

>XP_001768323.1 predicted protein [Physcomitrella patens]

MKRFFKSIGREGSFKRLATSRGEDGTPTCAREDSEKREPTKFMSWNTNSFLLRLKTNREEVLSLLRRLDP

DVIAIQEVRIPSAGRKGEPRNHGELKDDTNSAREDKQIMMRALSVSPLSDYGVWWSLSDSKYGGTALLVK

HSCSPISILYSLDDKLKHEADGRVILAEFHSFRLLNTYVPNNSWKDDDNGFARRRAWDARMIEFLKRPHK

KPLIWCGDLNVSNEVIDVSHPDFFSNAKLQGYTPPNAEDIGQPGFTLGERQRFAECLSKGDLVDTYRHLH

KQQDFDAGFTWSGNPVGKYRGKRMRIDYFLLSRKLLDRLVSSDIHGQGIEQEGFCGSDHCPITMEIRTAT

KEIAKVECIQSRQVDSLDL

>OAE34588.1 hypothetical protein AXG93_1487s1340 [Marchantia polymorpha subsp. ruderalis]

MLALPSSLVAQLRFSSLSFGGSGSCAGAFVARTRASSQCAPVLRCIPATACPDLARGCRLCLGRRSFCCS

TSLSAAAGAARPHNWTGSLRRARVWELPGGVCCTRCSLIPLDPRSSCILDSGNASTGGHAGRLLPSRKVD

RSRAESWSFHHCSSGREGGVNSGNFQVKCGITFGTSGEMTSVASREGGRQLRQRKETHISLSTQAIGRKK

TKGVAREATSTDDEMELRQGIEESAAATEFEARLWDLDKSNKLTTLTVLELRGYMKLMSKPTAGRKDELI

STVKEWLANRESSTLMSSRQAAAIQETSSDAESLEGALLKAENKNREAEAEAMSRRIDIPAKIVNQKRKD

AAYSDLKVAQQDTAVSASPKRTSVDISMSGKGRLSKTDSESNGTLDDEIVVKAVTKRKIQSSTVVKTNDA

ASAAAWEDQGKKRKLEQEAKDSQEEISIRIVEEKIVEINGDQKKPWLTLAHKKPQADWVPYNPETMRSAS

LPSSVKAMKLISWNVNGLRALIKEKDKKTEEGLLLQLAKEEDFDVLCLQETKLQTKDVEQMSSLLPGYTF

GSWSCSTAKLGYSGTAIISRVEPLSVKYGMGIPDHDNEGRLITAEFESFYLVTGYIPNSGEKLVRLAYRT

AEWDPALSQYLKELEKKKPVIYTGDLNCANDEIDISNPDVDSEPQLLEVTLSDVLLTCFNIYDLIIIAHH

IPIVVASQQGNRKSAGFTKEERESFKTNFLNKGLVDTFRKQHPNVLGYTYWAYRSGARVKNNGKDYSLYP

VSNMLEQSLVVKMYELLQAYGGYDLTWFSFMHTLGYLMCTSDVLQDGG

>OAE32599.1 hypothetical protein AXG93_3228s1160 [Marchantia polymorpha subsp. ruderalis]

MKRFFQPVAKNGTATKKQAVESKELNDVTESAAAEAESGNEAKEPLRFLSWNANSFLLRLKNNRTEFVDL

IRSFDPDVIAIQEVRLPAAGRKGESKNQGIIKDDTSAAREEKQAVMRALSVSPLSDYQVWWSLADSKYAG

TALLVKHCFKPVSVKFSLESGASRDTHVPDGRVILAEFKTFRLLVTYAPNNGWKEEETSFQRRRKWDKAL

RKFVSQESKKPLIWCGDLNVSHQDIDVSHPDFFRTAKQKDYTPPNAEDVGQPGFTAAEKQRFSQILKDGG

LVDSYRWLHKSQDFENGFSWSGNAVGKYRGKRMRIDYFLISEQLVGRLLHSDIQGSGIDHKGYCGSDHCP

ITLELSPATSTVPAHLPHVSSIDTDSTTGIDVKEITPEGVCP

>GAQ82037.1 putative DNA-binding SAP [Klebsormidium nitens]

MDPSQVEVLHTQNALHKLTVSDLREFIRLKAIQAPSNAKKVELIASVSEFLASEESIETLAQESNTVAVA

EEEVPAKDTRTASKNRAGSQVDDTEGSVAAVGRKSRKRGAGAAKDREASGVVLAGEDAQDAVSPQRRSTR

SRKDSLSRPAEEPPALSKANEVGEENGRPKRARRQSTGSPETAKKPPPLKRKPKTGVLSLTQAQSTVEVT

EVQSDVVDGALVEKKRTKKTTVARGFGLKGDVGKVEISQSEESWSHLVHKKAEPDWIAYNPGLMRPPPPK

GPFKKVVGWNVAGLRAVLKLERRWFDEIAEKERPDVICLQETKIQEKDIASVEEGLLPGYHKFWATSSAK

LGYSGVALFSKEEPLSVRYGLGIPSLDAEGRLITAEFADFFVVSAYVPNSGDGLKRLEERTQVWDPALAQ

HLKGLEASKPVVLTGDLNVAHQEIDICNPDGNKRSAGFTVEERESFERLIMANGFVDTFRRQHPKAVGYT

YWGYRFNSRASNRGWRLDYFVVSEKLAPLVHDSYTLPNVLGSDHCPVGLIVKT

>XP_005848578.1 hypothetical protein CHLNCDRAFT_30910 [Chlorella variabilis]

MRSWLRGGRTCWWQDVFGCRSLVAHVPPLAMAPKRKAVAPPEQAAEPAAATTAGIDKKTAAKPKAAKRVA

KPKAPKPPLGPAWDASMRPPPLPDGTPASHILSWNVAGLRALLKKVKEVEAGTRNENIPTLVALAEAEQA

DVLCLQEIKLQEDHCVEVLKELNLPEGWHVSWNCSRDKKGYSGTAIVSRQAPLSVSCGIGAEEHDGEGRV

ITAEFSAFYLVNCYTPNSGEGLKRLGYRVEKWDKDFSAYLKRLEQHKPVVVTGDLNVAPAEIDIHSPKTN

LKSAGFTPQERASFAANLLGQGFVDCFRTQYPEAVAYTYWGYRFNARGNNKGWRLDHFLVSQQLHSSLHD

CYHLPAIMGSDHCPLGLVLKH

>XP_001703445.1 predicted protein [Chlamydomonas reinhardtii]

MKDDGTSAAKEEQAVAAEPAAAAPRGRGRKTPAGAGAEGAAATAKKSPAKGRGKKAAAAKEPEPESEEAA

ASEGTAEGASEEAAPKRAAKRAKTGAKTKCHTPPPHPKNIVCCHLPSPLAFAVVQYTAALRKPAPPAGST

PLNILSWNVAGLRALLKKTPDAVSSLVSREAAEVVCLQEHKLQANHQKEVEELLGLQGWHHAWAFSTAKL

GYSGVSVHTRSPPLSVVVGLGHGGPGAADPDPEHEGEGRVVTVELEGLFLVNVYVPNSGEGLKRLDYRVG

RWDGAFAAFLQGLQARGKPVVVTGDLNCAHKEIDIHAPKTNLKSAGFTPEERESFGRLLLAEAGLADTFR

RLYPDTVAYTYFTRRFNCREKNKGWRLDYFLTSESMMPPELQQGGEGAAAAAGPASAWAVYDTWIMQDVY

GSDHLPLGLTCVRKAAA

>XP_003078172.2 ARP protein (ISS), partial [Ostreococcus tauri]

XXXXXXSALSFTFFGFCFACTSSNPLYRLHRSAYSLHASSNARPPPLETVTTRSGTARIARAPPAAVTVT

ITRALRPFARPVSLRIDDDVDANSARRRVAPRSMTSANEAKSSGEMNADEPEAKRPKPSETKASKTKEPA

KRFEQTEIARNVAPGAKTTRDGRETFKAISWNVAGLRSFVEKSGAKLRALVEEERPDVIILQEHKLQMSH

IDAFGSKLKELCPGYDTTRFAVSTAKKGYSGIVAVSREGRKGGQVTIEAMLGGASKPAVSDGFKAVSHSE

GLQGGTAYVDEGRTLTIEYEKFYVVSAYVPNSGQDLKRLDYRIKEWERDMKAHLKALDAKKPVIYIGDLN

VAHLDADIWNVTASHIKKSAGTTPQERGAFGVMLEENGLHDSFRFFHGDAAGWFSYWSVRAGNRPFNKGL

RLDYAVASKRLFDGDDSGVEVVDAFILDQVTGSDHAPVGITLAIKD

>NP_055296.2 DNA-(apurinic or apyrimidinic site) lyase 2 isoform 1 [Homo sapiens]

MLRVVSWNINGIRRPLQGVANQEPSNCAAVAVGRILDELDADIVCLQETKVTRDALTEPLAIVEGYNSYF

SFSRNRSGYSGVATFCKDNATPVAAEEGLSGLFATQNGDVGCYGNMDEFTQEELRALDSEGRALLTQHKI

RTWEGKEKTLTLINVYCPHADPGRPERLVFKMRFYRLLQIRAEALLAAGSHVIILGDLNTAHRPIDHWDA

VNLECFEEDPGRKWMDSLLSNLGCQSASHVGPFIDSYRCFQPKQEGAFTCWSAVTGARHLNYGSRLDYVL

GDRTLVIDTFQASFLLPEVMGSDHCPVGAVLSVSSVPAKQCPPLCTRFLPEFAGTQLKILRFLVPLEQSP

VLEQSTLQHNNQTRVQTCQNKAQVRSTRPQPSQVGSSRGQKNLKSYFQPSPSCPQASPDIELPSLPLMSA

LMTPKTPEEKAVAKVVKGQAKTSEAKDEKELRTSFWKSVLAGPLRTPLCGGHREPCVMRTVKKPGPNLGR

RFYMCARPRGPPTDPSSRCNFFLWSRPS

>XP_003215787.1 PREDICTED: DNA-(apurinic or apyrimidinic site) lyase 2 [Anolis carolinensis]

MRLLSWNVNGLRAGAGAAGLKPLLDALGADVICLQETKITRDLLEEPLAVVEGYNAYFSFSRTRSGYSGV

ATFCKATATPEAAEEGLSGLWTKHEGAIGCYGAPGDFTTEELQALDSEGRAVITRHRICTSEQQETTLTV

VNVYCPRADPEKPERGEFKLRFYHLLQARAEALLRAGGHVVIMGDINTAHKPIDHCDPGDLESFQEHPGR

NWLDGFLWEPGKESPHGELFVDTFRFLHPTQKEAYTCWCNVTGSRHLNYGTRIDYILADRALALSELKEA

QLRPEVFGSDHCPVQAVLKSVCLGAPRCPPLCTRFLPEFAGTQQKLSRFLVKVERNGLLEKRQKPDWVET

GAATVVSKKSRTILSQKGQVDLRHFFKGGNRTEGTDSAECQSDTYMGWRATEDSGKEPPGETARVDGHGG

EEVASETQAPVEIVREKSSIGVANERTVDCRKDGVTLKPTQSAALWRSLLPGPARPPLCKAHGEPCVLRT

VKKPGANCGRRFYVCARPLGKSSDPRARCDFFLWASRDNL

>NP_001086779.1 apurinic/apyrimidinic endodeoxyribonuclease 2 L homeolog [Xenopus laevis]

MKIVSWNINGIRATRVGLKETLDSLDADIICLQETKVTRDLLDEPSAIVEGYNSYFSFSRVRSGYSGVAT

FCKSSTTPQAAEEGLSGVFCNRTGSVGCYGNTEQFLEEELQSLDQEGRAVLTQHRILNCEDKEETLTVIN

VYCPRADPEKPERKTYKLRFYHLLQTRAEAILQNGGHVIILGDVNTSHRPLDHCDPTDLETFEENPGRQW

LNQFLGDPIPSQKGDSETVMPPSAGSGLFYDSFRYFHPTQKNAFTCWCSASGARQTNYGTRIDYILGNRE

LVESEFLDSVIMPEVEGSDHCPVKAFMKCQPIAANKCPPLCTKYLPEFAGRQQKLLQFLVKKENTLGNTT

EESSELTGTPSFTEGADISTVRKRPSDKLNSTSKKKSKIVTKNGQGNLLSFFKPERQKLTMATECNPIEV

PICKKEKTVQKDLQPATPAVKYNKPQTAFWKSLLKGPPPPPNCKGHSEPCVLRTVKKAGPNCGRQFYVCA

RPEGHSSNPQARCNFFLWLTKKAGCED

>XP_014352550.1 PREDICTED: DNA-(apurinic or apyrimidinic site) lyase 2 [Latimeria chalumnae]

MKIVSWNINGIRATKSGLKALLDSLDAEIICLQETKVTRDLLDEPTAIVDGYSSYFSFSRGRSGYSGVVT

YCKNCVTPFAAEEGLTGLLTNHSKAVGCYGNTEEFSNEELLSLDSEGRAVITQHKICSSDQREQVLTVIN

VYCPRADPEKPERKVFKLRFYRLLQARAEDILESGSHVIIVGDINTSHRPIDHCDPEGLESFDDHPGRKW

LNEFLYQLKGEGGSSESLEGHAPGSGGAALERQRGGLFVDTFRLFHPAQRDAFTCWSTATGARQTNYGTR

IDYIFAGRVLADEEFEDSFLMPEVEGSDHCPVKAVLKCCCLAASRCPPFCTKYLPEFAGRQQKLLQFLVK

VDKSKVCASDEESLPSSQETSEITENLAPSGKTKLGWKRGRTELGEKNAKKTKVEAGQGTLLSFFKPNCA

TTTAVSQSEEAKLGGASLKTEDLGTPPVQGSEAEIAREVGKKPQSAFWKTVLKGPPPPPICKGHNEPCVL

RTVKKAGPNYGRQFYVCARPEGHASNPAARCNFFLWVGKSR

>NP_956440.1 DNA-(apurinic or apyrimidinic site) lyase 2 [Danio rerio]

MKIVTWNINGIRTFKNGIKKILDSFDADIICVQETKVTRDLLDEKTAIVDGYNSYFSFSRGRSGYSGVAT

YCKDAATPFLAEEGLTGLLSNQGAVIGCYGDQVELTSEELLALDNEGRAVITQHHFIGQDGLQKLTVINV

YCPRADPDKPERKEFKLQFYRLLQCRAEAILSSGSHVIILGDVNTSHRPIDHCDPDDVDNFEDNPGRKWL

DQFLFETAENSENGNAADEPAEDFQESASGGKFVDSFRYFHPKRSNAFTCWSTLTGARQTNYGTRIDYIF

SNHSLVKTFFIGVDIMPEVEGSDHCPVWAQLSCTLQSSPRCPPVCTRHMPEFIGRQQKLSRFLFKIPEKQ

NISNSSEKSLPGSQDAGEIRENLNPVVQKQNVGKKRPTDREDTNAHKSKKSKTTKTESNAKGSLLAFFKP

KQTQLIPTKEKQIESCQDGPGTGSNSKICPSDLQNDMNVSKLLEMETENQDEVEGESTSMDKPNDCKKGP

CTGFWKAVLHGPPQPPLCKSHNEPCVLRTVKKAGPNLGRQFFVCARPQGHASNPQARCNFFAWVEKGK

>XP_020374300.1 DNA-(apurinic or apyrimidinic site) lyase 2 isoform X1 [Rhincodon typus]

MAMAVAVGETEVSGLRLVSWNVNGIRARGKQLRSMLQRLGAEIICLQETRITRELLDEPTAIVDGFNSYF

SYSRRRRGYSGVATFCTDNAAPFAAEEGLSGILSNPTSCALEGFSDKELKELDGEGRALITKHRVRTCEQ

KEVMLTVINVYCPRADPGREDRGHYKLRFYQLLQARTQALLQDGGNVIVLGDLNTAHRPIDHCQPGDPNQ

FAEHPDRVWMDRFLSCASDQSQLDEINQNSGSKPGLEVRERSGNGFFVDTFRLFHPTEREAYTCWRTNTG

ARKTNYGTRIDYIFANKSLAETELEDCILMPEIEGSDHCPVKVFLKSCCVPATKCPPLCTKYMPEFAGQQ

QKLSKFLVNIQERQSRLDLQNYAETKQHSKLQLNLQPKKVKLNLRQTGKGLMREKGKKGRGNFHPSGSLL

KFFKPADTNPAFKLETCDDLGLFNKTAPTDTGQQQANSKEIKQDMQIENPQSSSRAAFWKSLLKGQPAPP

NCSGHREPCLLRVVRKPGPNQGRQFYICPRPEGAVSNPESRCNFFCWVNTRN

>XP_019627661.1 PREDICTED: DNA-(apurinic or apyrimidinic site) lyase 2-like isoform X1 [Branchiostoma belcheri]

MRILTWNINGVRATKAPLKQVLDSLDADIVCLQETKVTRDQLDEASAIVEGYSSYFSFSRARSGYSGVAT

YCKDGAMPIKAEEGLTGLLTNQREADIIGYYGDQTMFTEEELRSLDKEGRAVITQHAICETNGKECLLAV

INVYCPRADPEKEERKDFKLRFYRLLQARAQALLQAGSHVIVLGDVNTSHRPIDHCDPDDDIEYFSENPG

RKWLDQFLFDPHQTEDSGGTLNPNNQSDASSGQLDTSLNSGIQNASEKEPTGGHFVDAFRFFHPTQRSAF

TCWSTVTGARQTNYGTRIDYIFANRDFLNQFQDCTIMPEVEGSDHCPVRGELACTVVPAVKCPALCSKYM

PEFAGKQQKLSVFFTRVKKGEVGEEKSSQDSVSTSQSSYDGHSESETCSQQSSSQLWESNSIKSEGSSVM

STTKQSQSRTSMKRTASDPAGRNVKKQKTDQGKQGTLLSFFGKKTKVETGDRRKANMSDIPKENPEADVK

EDAVHDNAISNQSVNSSNTTVDSDDSKAKGSKDAENGKSTAETKPKQNQAAFWKTVLKGPPPPPPCKGHK

EPCVLRTVKKQGPNFGKQFYVCARPQGHASNPEARCDHFQWVSRGKDKL

>XP_022082585.1 DNA-(apurinic or apyrimidinic site) lyase 2-like [Acanthaster planci]

MNILTWNINGIRASKVAIKELLDSLDSDIICLQETKVTRDLLDDAIVNVDGYSSYFSFSRKRTGYSGVAT

YCRNSVTPSRAEEGLTGRLAKGSDDIGCYGNQTSFTNEELQSLDSEGRAIVTQHTIKDTEGGLHELVVIN

VYCPRADPDNQERMAYKLHFYNLLQLRAEALVKAGRHVVVVGDINASHRRIDNCDPGPDFEEHPGRQWLN

SFLHDNTPGKLSSETETSLTLTNDSESSSNKQQHKPSDSSPRIFVDTFRRFHPTRQKAFTCWSTRTGARQ

TNYGTRIDYIFTNVGLFQRMVTSCDIMPEVEGSDHCPVKASMSCEPVPSTKLPELCSRNMPEFLGKQQKL

LSFFQKLSPSKVALARKVEMQESRPRDDSKLGLKRTGSDPGSHRSQKKFKMDPIEPRRGSLMNFFPKKKS

QEADSGNKMTDADVVTKPELVKSTSMPPADLNAMIDNNTNGGSPDTCISETSLNLKSKRFSQDSLTDGSN

RSKANSASVWKQLLSGPPPAPACKGHNEPCLLRTVKKPGPNLGKKFYVCPRPEGHKNNPEARCNTFIWVT

KAK

>XP_784420.3 PREDICTED: DNA-(apurinic or apyrimidinic site) lyase 2 isoform X2 [Strongylocentrotus purpuratus]

MCLTLAWHVVLSRSLSIHIPCSLTKMKVLTWNINGLRACKVPLKELFANLDADIICLQETKITRDQLDND

LVNVDDYNAYFSFSKKRSGYSGVATYCKNSTTPVIAEEGLTGLLSHGTKQSLIGCYGDQSAYSPEELLSI

DAEGRTIITQHQYRKKDGALGDLVIINVYCPRAGDDNPERKSFKMRFYNLLQLRAEAMLQAGKSVVIVGD

VNASHRRIDHCDPSGNWGKFESHPSRKWLDGFLKDCSCPETAETSPSSQDDLQKSPQEEQELSDEERHVH

DDSNIDVEDIPSKDNLFVDSFRYFHPKRENAFTCWSTLTGARQTNYGTRIDYIIVNERLCVDELTECDIM

PEFEGSDHCPVKATLLGGCSPAEQLHPMCAKLMPEVAGKQQKLSNFFQKVSPSKKFEMHEKAMSGENKQG

GRSRLDIGKREGQSLVGKPGKRAKTDGKVTKTGNIASFFKKKADAKLRSQTDLEIHQSSSSSYNTRLMPN

SQRSDGDIDNGSDFQNRLKDIPVDSGISGSDDAVSDASQIVKETSQELFEDKPKAKQQLASAWKNLFKGP

PPSPMCKGHQEPCLLRTVKKAGPNLGKQFFVCKRPEGHKNNPEARCNHFEWVSKKLNLKSV

>XP_002739716.1 PREDICTED: DNA-(apurinic or apyrimidinic site) lyase 2-like [Saccoglossus kowalevskii]

MRILTWNINGIRAAKKPLKSLLDSLEADIICLQETKVTRDQLDEANAVVEGYNSYFSFSKGRQGYSGVAT

FCHNDFTPLAAEEGLSGILSSSAGNEVVGNYGNQTDFTDEELRSLDNEGRALLSLHKIKSSAGKNEDVVI

INVYCPRCEPDDQERLNFKLRFYSLLQTRCEALLSAGRHVIVVGDMNVSHKAIDHCDAAVNPTKPRQWMD

QFLCKLSDMGEIIDKTSEVNHLIGAVGISPEKVSNYSQSTGSFVDTFRHFHPQQQEAFTCWRTTTGARQL

NYGTRIDYILASMSLQVELLDSIIMPNVEGSDHCPVKVTLRCTCETSKKIPTLSTKHMPEFAGKQQKLSG

FFTKAAIESSHINTTPEIDSKISHPKLTNHYENCSASNLLKRTSSANESNATKKQKTTNRPKMHGNISDF

FQKKSVSKTSDHPSIQSKDQVIPLECAISVAPRAGEDASHSIPMQSGMKDEKKKVAAKWKNMLSGPKPPP

LCRGHKEPCVLRTVRKTGLNQGRQFYVCTKPEGPKSNPNARCDFFQWADKK

>XP_021930302.1 DNA-(apurinic or apyrimidinic site) lyase 2-like isoform X1 [Zootermopsis nevadensis]

MSENQQELNKVKIITWNVNGIRSLYKDAAFIKKGLDSLDGDIICLQETKITRSALDQGTAIVDGYNSYYA

FSRRSGYSGVATYCKDAFTPFRAEEGLSGLFESSGAVDSVGCYGNIRAEFSKIELREIDLEGRAVITQHK

IKISSGEDKTLTIINVYCPRADLNNTERLRVKLRFYHLLEVRTRAILDSGSYVIIVGDINACHKPIDHCD

PSDVEEFNENPCRKWLNHIIYNPSAEVNTNNVNISTFHLIDAFRFVNPVEENAFTCWCTTLNARSTNYGT

RIDYILIDLRLGGEMDDCIILRDVLGSDHCPVKAIIYLKFLRSPKYPSFCTKYYPEFAGTQKKLSEFFSK

KSCDQEICTNTTIDYTEDATISFGKRKLPGTKVMPNRSKGDTKKQRGISSFFKLLSSETQSTDSCESVDV

HSQDSGKESSLESLSSLSALSEVRPDSCESFQPLPIVDTPSSGDMTEEGSSIVVCNESEAEQNCSAISGR

EKKAASDAWKSMLTGPKAPPKCKGHREPCVQRCVKKKGPNFKRKFWVCARGEGRSNDPHAKCDYFQWYSN

R

>EFX88187.1 hypothetical protein DAPPUDRAFT_305561 [Daphnia pulex]

MFNIVTWNINGIRSSQQNLKDLFDSLGADIICLQETKITRDMLDESTALVDGYSSFYSFSRKRSGYSGVA

TYCKNSYSPFQAEEGLAGTFNVHDDKIDFYDNVHSSFTDEGLRALDAEGRCVMTLHKFKKEETSISLVVI

NVYCPNAGENDERLPYKLEFYRALNLRCHDFLSQGYYVIVLGDMNVSHRLIDHCEPEDVSSFPKSPSRLW

LDGFLHEGGGKFVDSFRHLYPTKEKAFTCWNTKLSARVNNYGTRIDYILLSNQLTDALQDCIIMSDVYGS

DHCPVKSSLALDIIPSTKLPALCTKFFKEFSGKQLKMSTFFCKRTLAVEPSNEIHGEPEGSEPKKMRLSM

KTTTKQQQSSLLKFFSSGTANSVKESVSSSNSLDTKKSEDDSKYFESTTSTFTTSISSSSASAAWKSVLK

GPPPPPLCSGHQEACTLRTVKKKGPNMNRQFWTCSRATGHSSDLNAQCNFFVWAKR

>XP_013773132.1 DNA-(apurinic or apyrimidinic site) lyase 2-like [Limulus polyphemus]

MKILSWNINGLRTLKRPLKETLDSFNADIICFQETKVSRSLLEEELAIVEGYSSYYSFPRWQSGYSGVAT

YCRDSCQPERAEEGLSGLITGQNCHDDSLGFNGNFKEFTSQELMLLDAEGRAVVTSHEVQCNKKVEKLVI

INVYCPRADPEKPERADFKLRFYYLLQKRAEKLVQNGFHVIIVGDLNTAHKPIDHCDPGDEVEFSLRPDR

LWLSSFLGECSPNEDNNKEHDITFIDTFRHFNKSHTEAFTCWMTSTGARQTNYGTRLDYILCNRNFISHV

MSSIILPEIKGSDHCPIAIELRCDPVASRRCPSLCTKYWLEFVGKQQKLSLFLRQPVQVTAQITDFQPVN

SYIPKRRANSHSETVSKKKQNVQKISQKSITSFFVPQTSTLQTTIPSKQGILDSVDNSTNSNLNIFTTID

HHYSSKNSASSCYSNPHMLFSHTACVSDDEKSVNIIPSGSSETSMICSHDAESDCNSDGNLSSQSSTKSF

SLKCENITKNFLQKSTYCVLEETDEQYQNPSFTLNTTKNNQCQEYHINNQMEKQKQLWKSVLCGPQPPPL

CKGHKEPCVLKTVKKQGPNNGRQFFTCAHPEGRAENPEAKCNHFQWVNPPKVKGKR

>XP_002405543.1 ap endonuclease, putative, partial [Ixodes scapularis]

MKLVSWNINGLRSFKAGVKGLLAELDADIICFQETKATRDVLEETSAIVEGYSAFFSFPRYQSGYSGVAT

FCKDAFRPFAAEEGLTELWTSSGCPDSVGFYGDTSDFDSKNISSVDGEGRTVLTLHHVRCGDKLKRIAVI

NVYCPRADPEKPERGQIKLDFYELLKRRAMTLLRNGLEVIILGDLNTSHRKIDHCDPSDDEDFDSNPGRI

WLSQFLENSTTNTCFHDTYRRLHPTTEKAFTCWNTRLGARQTNYGTRIDYVLCSPALVPFLQTADILPHV

LGSDHCPVEAIFRCDGVPSPRCPSMATKFWPEFAGRQQRLSAFLSKKPGDQEEAERDRRPQSSEGWEDSC

AVSVELRNGGPPVKKSKNVPARTTDKSHKTAQSTLNRFFVSSNKTDVGVSKQSSRSSHGQAKLGDCGLPV

VNSGTNVSGAPEPAPKRAVDVACAWKSLLKGPPVPPPCKGHGEQCVLRTVKKPGPNLGRQFFVCARPTGK

SGDVNASCEFFQWVNPA

>OQV14763.1 DNA-(apurinic or apyrimidinic site) lyase 2 [Hypsibius dujardini]

MKIVTWNINGIRTVRGPLKTLLDSFDADIICLQETKVTREMLDEGIAVVDGYNAYFSYSRKKSGYSGVVT

YCRDSARPVAAEEGLSGHWSSMKAASTLDSVGVYGRITDYPSKELQELDAEGRTVVTEHRFRRPDGTEGT

LCVINVYCPRADKEREDRKAYKLWFCEMLQVRTEAMLAQGRHVIVLGDLNTSHRPIDTCDSSGDDYATSP

TRVWLDGFLCESSTATDIQLDYQSADPGHRMIDTFRQCHPGREKAYTCWSTATGARTLNYGCRIDYILCD

VQFANSCLAQSEILPETAGSDHCPVMSEFDALFLPAVKCPSLCAKWMPEFTGKQQKLSTFFQKAAKQEVE

DDQSVRGRLRLTDTVSSTKIAKPGAKRKNDFTTGQVQRSVLDFYGSKVKKVEVEGSPTSQSSISFPVVRT

ASVTGSSAVSFDVSEEFESSSLPDFVDIAEMDEEEDLLAEFAIETAGSSSYNGSSSSSGSSSQGPSSQPD

SQWVKSSSQESAAQWRSLLRGPDAAPLCNGHGEPCILKTCRKAGPNRNRQFYMCSRPEGEKTDKSARCAS

FKWIGKGGSVLRK

>XP_014665229.1 PREDICTED: DNA-(apurinic or apyrimidinic site) lyase 2-like isoform X1 [Priapulus caudatus]

MSKDIIKILTWNINGIRATKLPLKDVLNDLDADIICFQETKITRDLLDEATAVVDGFNSYFSFSLVRSGY

SGVATYCKDHVTPVRAENKLSGLLTAGNDASSDTIGYYGNLEQFTEEELKCLDNEGRAVMTEHKIRGQDG

AENSLVVINVYCPRADPEREDRKSYKLRFYQLLQIRAEALLNSGRHVVIVGDVNTSHKPIDHCDPAQCKA

FASHPGRKWLDGFLWTPEKEGTQPGTTNNDRSCEWPPGVAAKRFVDCFRFLHPTVQGAFTCWSTVTGARQ

TNYGTRIDYIFADRCLATTALVDCDVLQHVQGSDHCPVKATFNADVVAANNCPSLCTKYGFSGKQQKLAE

FFPKTHERAVAQPLDVGTTAIARDRKPQSKQQSRNPAEHARKKAKIETGKQRTLAGFLLKGAKKLDAVDS

SQLTVAKQIIWKDVLNGGSETITGTRVQSTLTSTRSQYGVDTRNNALTNELYFSVRGECNGQNGADNADV

AAAAAGNRGKDPASNVHCAADAPAPAAAAKSQAAQWKGLLRGPPPPPVCRGHGEPCVLRTVKKSGPNVNR

QFFVCARPEGHKSNKEARCEHFKWVTKPKKYL

>XP_013381669.1 PREDICTED: DNA-(apurinic or apyrimidinic site) lyase 2-like [Lingula anatina]

MKIITWNINGIRATKSPLKKVIDDLDADIVCFQETKVTRDQLDEPTAIVEGYSSYYSFSKVRSGYSGVAT

YCKTHATPVKAEEGLTGLLATTGDDSALIGCYGNHADFTDEELQSLDNEGRVIITQHKIRAEESKEELLT

IINVYVPRADPEREDRQVFKQRFCQLLQTRAEALVHAGSHVIILGDLNISHRQIDHCDPVEKEEFDARPS

RKWLNAFLKPIGTAAEPAQDVEADTDSIVTGGIFVDTFRYFRPNEKNAFTCWNTSTGARQTNYGTRIDYI

LASADLCEGLVRDCVIRPDIEGSDHCPVKAELTCDIVQSAKYPPICTKFMPEFSGKQQKLSEFFSKSVKE

PKHTSVELVKSEATIFKEEFCEKDESVTDARTITEVKHKTETRLLSKRAAPLLMKQTSKKSKMHDRKGKQ

SSLMNFFVSKTITPAKEKGSLDSGIENRTCIEDGIFLSQNSDGEGTYSSQESNETSSVTYSESISLHSSQ

ESNETPSSQETINKSQNLKDAWKTMLKGPPKPPSCKGHKEPCVLRTVKKEGVNFGRQFWCCARPQGHASN

PLARCEHFEWLPKKKKGSLK

>XP_005112838.1 PREDICTED: DNA-(apurinic or apyrimidinic site) lyase 2-like [Aplysia californica]

MKVLTWNINGIRAGRGKTSLKVLLDSLAADIICLQETKVTRDMLDEPTAIVDGYEAYFSFSRKRTGYSGT

ANYCVSRTGPVKAEEGLTGRLYISADCESLVGHYGDMEHLSDGDLDALDAEGRCIITQHKIRLAGEEKEV

AIINVYVPRVGEDREDRHEYKVKFLGMLQGRAEALLEEGIHVIILGDLNLTHKPLDNPEYDDNFQVRPSR

VWINNILQAKEQDSSLPPSTGMISELCPGNLKGGRFSDVFRQLHPQKDDGFTNWNTATDARKANFGRRLD

YILVNLELAKYVTSCEIMAEVEGSDHCPVVVTLSADPVAASVTPNLCTKLMPEFRGQQQKLSSFFSKTEK

FAASSLSSSPVQVDSDSIVLSSSQEDSGNVLSCSPFTKSKSLPPQVSEKSKLKRNSSAVDNTKNKKLKKN

DTGSKQASLISFFSSSSSSKSDKVSNNSKASLNVQRRSDSASKNNTIEVRDDKCIDNSVKAETLEIVETV

QLGEEGEVNKLCDSSTAKQEVPLRRTSTNISSSSSSSVAPINPPPTKSSASVWKSLLGGLPPPPLCPGHK

EPCVLKTVKKAGPNKNKQFYSCPRPDGAPGKPEFRCNFFQWLNDKKKKKT

>XP_014779818.1 PREDICTED: DNA-(apurinic or apyrimidinic site) lyase 2-like [Octopus bimaculoides]

MKLLTWNINGIRASKIPIHQLLESLQADIICFQETKITRDMLDEMTAIVDGYNAFFSFSRKRSGYSGVVT

YCKDHCLPVKAEEGLSGTLNKKGADDVVGYYGDHSEFSKDELDALDAEGRTVITQHEIRNPDGSCSQMAI

INVYCPRYDSENEERHSFKLRFFALLQTRAEALIESGSHVIILGDLNTTHQNMDHCEPDSEGGSHSIPSR

CWLNQMLCHKQQTDELSDKSDLHDKDTQPATVTGGHFVDTFRYLHPDQLEAYTNWCTLTGARQTNFGRRL

DYILCNERLNSALTSCCIMANIDGSDHCPVKASFNFSVVTALKAPSHCAKFMPEFAGKQQKLSMFFTKSY

GKPSTDMSGNNNIVQHSASTEWKDKRKHSPHSLKRQSSESLGSQKKKGKSCLENKPKQINLMGFFNKKPS

SCPGQLGRNSSSELDEFKNSKKTSSDDSDINDLSESQEVTTEPSISSHLEISSKNVQDSSLLEADKEELI

VSKVKSTTDQTSNSQVLAWKNLLKGPPPAPLCPGHSEPCVLRTVKKPGPTQGRQFYTCSRPDGAANNQEA

RCNFFKWIKDLKK

>XP_011447834.1 PREDICTED: DNA-(apurinic or apyrimidinic site) lyase 2 [Crassostrea gigas]

MKILTWNINGIRASKIKLSELFDSLDADVICLQETKVTRDMLDEPTAIVEGYNSYFSFSKKRSGYSGVAT

FCKASATPVEAEEGLYKELNPKLEGKVGHYGDVSEFSSDELEALDAEGRAVITKHKIRKHDGVEAELAII

NVYCPRVDPDREDRLLYKLRFFALLQTRAEALLQSGCNVIVLGDMNVKHKSIDRSEEDEVGTVTTPSRVW

VNQFVWDKDQDPSIAAVENKEDFAGTTSSVRGGLFVDSFRYFYPDQTGAYTNWCTLTSARETNYGRRLDY

IFTNVELAQSDLKDCVILADVEGSDHCPVKAEYMAEFISAEKCPPLCTKHMPEFCGKQIKLSSFFMKREK

NIENSIEIKTSQDTVDQTHIKNTKNDKTEKVTLKRPSNGDKIGNAAKKKKSDSSDSRQGSLKGFFLKTST

KKEVVDIKMEEVSSETKGENHSSEMKVKIESTITVKSNTASAWKNLLQGPPPPPLCKGHQEACVLRTVKK

PGPNKGKQFFVCARGEGHSTNPEARCDFFKWVDYKKK

>ELU06313.1 hypothetical protein CAPTEDRAFT_176829 [Capitella teleta]

MKILTWNINGIRASKVPLPQLFETLDADIICIQETKITRDMLDEATAIVEGFNAYFSFCKTRSGYSGVAT

YVKNSCTPTKAEEGLTGVHEADKAEHSIGFHGDYSLYTEDEIRSLDNEGRCIMTQHKMLSDDGEEIRLTV

INVYCPMAVLDDPDSVERMAFKLKFYDLLQKRAEAILQSGSHVIILGDVNASHRPIDHCDPCDEKEFNQH

PSRKWLNNLLRTPTNSNGLFVDSFRHFHPDQREAYTCWQTLTGARATNYGTRIDYIIGDTRLVEDSFSDC

VILPDVRGSDHCPVKAVLHWECIPAKKYPPLCSKYLPEFVGKQQKLSSYFSKGSGPKSGSDTSSQESNDS

TKSYTASKRSGDSTVEVKPNKKLKSVGFKQGSIQSFFKQNRTQVTSNSAVALLQTVAAESGKRQSDCVTT

WKSIFKGPKAPPTAPLCSGHKEACLLRTVKKDSLNKGRDFWVCNRPEGHKSNKEARCEHFEWVSKK

>XP_009019767.1 hypothetical protein HELRODRAFT_94522 [Helobdella robusta]

MKKKTFKMIIATWNINGIRTTLSKTPLKKLFQELGCDVICLQETKITGDYLDGSTAIVEECNSYFSHSKV

KSGYSGVAIFAKNNFTPVSAEEGLTGKLRANQNAPNSQVGNSGDDLTQFSDGLTQFSDDLNQFSDDELLA

LDGEGRCLMTEHIILDNEERERKLVIINVYCPRADPDRPERLSFKLRFYQLLQIRAEALLKSGRHVVIAG

DINTIHKPIDHCDPASDQEFMEDPGRQWMDGFLRSSPTPSLPPQNNLLLPQLRQERQPKFVDLLRLFEPK

TANLFTNWCSTTGARATNYGCRLDYIFADIEFASTYCTSCYIRPDIMGSDHCPVVAHFNCRVIPAERCPP

LCSKYLPQFAGKQTKLSTYFVCGDLSSSQESCSGSGRGVSDFSSSSNFSIGSINDSYEKSNKHGSPLSIT

NNKRTLGGKCKKASNFKTDSKTIIKFLSREKLHANSETHSTSEIKTNFDESVDSLEDTKNRTNLIKDNLK

ITATSYFQSSSLMETKPPQSKPTKLNESWGKLLKGPPKSPYCTGHNEPCVLRTVKKEGPNKGKQFWVCCR

PEGHKSNPAARCDYFLWIHGK

>XP_001638192.1 predicted protein [Nematostella vectensis]

MKILTWNINGLRAVTREKKLKDFLNSLEADVMCFQETKITRDMLEEATCMADGYTAFFSFSRVKSGYSGV

ATFCKDSTTPLAAEEGLTSQLSASPDIGFYGDDEEFTSDQLSRLDSEGRTILTEHTLSSGGTVVIINVYC

PRADMENEDRIQFKLEFHRLLSKRVKALLNSGKHVIVLGDINAAHKPIDHCNPCKYEDFSSFPGRAWLDE

LLVSLPLPDNSCDSSWKCVSGLLIDSFRYFHPLQREAYTNWSTSTGARQTNYGTRIDYILVDPPLLQQEF

VDCVIRPEVEGSDHCPVVCTLKNRFLAANKTPLLCTKFMPEFSGKQQKLVSFFTKKTDNAFTVPTDLNNE

KVISETELSASNTTKKRAPSSGSAVPNPKRAKTEKSTRQNTLLNFFGSKNVTKASSPKCIVDIVTDKPSG

EVTREDTKSNQNMLLNPEPYSSVSAWKSILKGPPPAPLCPGHNEPSVLRTVKKKGPNYGRQFYCCARPEG

HASNKEARCNFFKWRK

>XP_012559420.1 PREDICTED: DNA-(apurinic or apyrimidinic site) lyase 2-like [Hydra vulgaris]

MKILTWNINGIRATSRNHSLGNLLDSLEADIICLQETKITRNLLTDHLANVEGYYAFFSFSKRKTGYSGV

ATFCKKTLCPIAAEEGLTGLFTSKMEQVVGFYNNPPEYSDELLRSLDSEGRCMITEHVTSDGQRLCIINV

YCPRADVEEKERFHYKMCFYRLLQMRIISLMREKRNIIVVGDLNVSHKRIDSCDPCEDFEDSPARKWLDS

IIDCSIIKSLVSTTSSINQKSDTLVNDENLKVKGSESFSEKNVNNFLVDTFRIFHPNEQNAYTCWNTQTR

ARETNFGTRIDFILADHQLCTVLTNCVILADVQGSDHCPVVADFNILLKASNLIPKTCAAFMPEIFGKQS

DIKSFFSSKRFESDREGIKRKLNDSTESRFYKKKKNVDQDFSIIKYFKASDTENHSRSSHVEISNFQSST

EEISCSRLSNLELSHNSSSSNFAYIIDNSNMNYYAVKEASSFESAVLYESSMKQKTENTNKEIWKKILKG

PESTPLCHGHKEKAVLRTVKKQGNNFGRQFYACSKPVGSSSNKNAGCGFFVWKS

>XP_002108178.1 hypothetical protein TRIADDRAFT_19413 [Trichoplax adhaerens]

MKILCWNVNGIRTTAYGKGSIKDLLEFLNADILCFQETKLLRDQLDESIAFVEGYNSYFSFSRGKVAYSG

VATYCKTDCAPIRVEEGLSNVWAAKNADRIGHYGNISKFNSEELSLLDAEGRAILTEHKTDFGRNVVVIN

VYCPRADPEKPERLNFKLKFYELLQERAEALLIAGKHVVIVGDVNTSHKPIDHCEPDIKAMQNSPSCHWL

DNLLIPLDSEGKVDSSISAGKKFIDSFRYFYPRRKEAFTCWSTVTSARKTNYGTRIDYIIADKDLVVSYF

AESQIQPEIEGSDHCPIYADLKFTINPSDKLPWLCTKYMSEFSKKQSKLTEFLKLGKESTERSEPTNTTR

INKMKDTNVTVDTKGSNQKRDSTCPVNIDVKRSKSSSNKSCNLLNYFFTKPSNTHKSNTSIIQPTVEKEP

LEAIKDKKDNNNDSRELNNSQQNNKENKKEDEAAMVANDKSETTKAWKSILSGPPPIPLCSGHKVPSVMR

TVKKSGPNQGRRFYVCTLPEGRRGNPNARCNFFQWAN

>NP_009534.1 DNA-(apurinic or apyrimidinic site) lyase APN2 [Saccharomyces cerevisiae S288C]

MSSSENTLLDGKSENTIRFLTFNVNGIRTFFHYQPFSQMNQSLRSVFDFFRADIITFQELKTEKLSISKW

GRVDGFYSFISIPQTRKGYSGVGCWIRIPEKNHPLYHALQVVKAEEGITGYLTIKNGKHSAISYRNDVNQ

GIGGYDSLDPDLDEKSALELDSEGRCVMVELACGIVIISVYCPANSNSSEEGEMFRLRFLKVLLRRVRNL

DKIGKKIVLMGDVNVCRDLIDSADTLEQFSIPITDPMGGTKLEAQYRDKAIQFIINPDTPHRRIFNQILA

DSLLPDASKRGILIDTTRLIQTRNRLKMYTVWNMLKNLRPSNYGSRIDFILVSLKLERCIKAADILPDIL

GSDHCPVYSDLDILDDRIEPGTTQVPIPKFEARYKYNLRNHNVLEMFAKKDTNKESNKQKYCVSKVMNTK

KNSNIKNKSLDSFFQKVNGEKDDRIKESSEIPQQAKKRISTPKLNFKDVFGKPPLCRHGEESMLKTSKTS

ANPGRKFWICKRSRGDSNNTESSCGFFQWV

>XP_003299056.1 hypothetical protein PTT_09967 [Pyrenophora teres f. teres 0-1]

MVRITTWNVNGIRNPFGYKPWSTTRTFNAMFDILEADIVIMQELKIQRKDLTDDMVLVPGWDCYFSLPKH

KKGYSGVGIYTRQSVCAPIRAEEGVLGALCPPNSSTPYRDLPGSASIGGYLSASQIASLPPDLDADSLDS

EGRCLVLEFPAFVLFGVYSPANSNGQRDGFRLGFLTALETRIRNLTKMGKNVILTGDLNVSRDLIDTAKA

EDNMRAEGMTHAEYLSTPNRRIFNQLLLNGNVPGERDQGREEPVLYDLCREFHPDREGMFTHWEQKINAR

PGNFGSRIDFILCSLAMKDWFKDANIQEGLMGSDHCPVYAVTKDMVPIVEEGTSHVTNVTHILDLMNPAG

VFQNGVRQRDYDSAKDMPPLSGRLLDEFTKRRSIKDMFSKKPAPRKPTTTPTPVTVQPKPSTEALVAASS

NTAASQMDRDLELAIAASLADTYPVTNTSPASTQSPLKPAEKRRPSASGSPSKPIKRTKSTIPIAPSKNQ

QSLKGFFQTRTTPSGPTTPSDPPASSPPTSADPPSQSTPSTIAADVPPLPPASPPPTASDSFETDPRASQ

EASKEGWTKLFSKKPPPRCEHGEPCTLYKTKKKGQNFGREFWVCPRPIGPSGVKETGTQWRCPTFTWCSD

WKSS

>XP_001246634.2 exodeoxyribonuclease III (xth) [Coccidioides immitis RS]

MTFRLTTWNVNGIRNPFSYEPWRGTQSFQGMFDLLEADIIVLQELKIQRKDLRDDMVLVPGWDCFFSLPK

YKKGYSGVAIYTRNATCSPIYAEEGVTGILCPPNSTSSYRELPESEQIGGYPTDEQLSTSELSAETLDSE

GRCVILEFPAFVLLGVYCPANRDETRDGFRLGFLNALDHRIRNLISLGKRVVVAGDLNISRDEIDSAHAL

EQIRKSRLTTDEFLSSPARIIFNRLVEGGRVSHIEGVDKKDAVLWDMCRSFFPARSGMYTCWEQRINARP

GNFGARIDYILCSLDMKDWMSIADIQEGLMGSDHCPVYAIFKENVDLHGDQIDFVNLMNPSVDFEAADNS

LSRSHLPLSGRLIPEFNRRRNIRDMFLRQSSTQNPSPGEINQNATSMSRMTNKGRPISLDLERSHEIDER

DPKRRKKEPIAGVLPTTSQKSIRGFFSPRQPRLKQSNLAKDCEAINDSVPNERKNDSGPGHRTSGISPPD

ASPDFEADPQTSQESTTVSAVPSRIVAGINVMSSIPEQESWNKIFKKRPPPRCEGHEEPCVRLVTKKPGI

NRGRSFWICPRPLGPSGNKETGTQWRCPTFIWCSDWNSKQ

>SLM38449.1 dna lyase apn2 [Umbilicaria pustulata]

MVRPPFSPIHAFSLINGEVNGIRNPFSYQPWRDKKTFASMFDVLEADIVIFQETKIQRKDLRDDMVLVPG

WDCYFSLPRHKKGYSGVVIYTRQSVCAPIRAEEGITGVLCPPNSSTKFADLPEGERVGGYPTPIQLASSP

VDATTIDSEGRCVVLEFPAFVLLGVYSPANRDESRDDFRLGFLDLLDARVRNLIDMGKRVVLTGDLNISR

EEMDTANAEASMRKNGFADQEYTSTPARRLFNQLLEGGKVFGERDEGREQPVLWDTTRGFHPTRKGMFTC

WEQKKNARPGNFGSRIDYVLCSLDMKNWFSDSNIQEGLLGSDHCPVYATLKDKIVLDGKAVDLLDAMNPP

DMFVGGQRQREYSMKDMLPLSGKLIPEFDRRRSIRDMFSKPSLPTRPSTGLSIAVQECDLIPAQPDEPND

RRPVERETENLQIGESEPASPAPPMQDPASVKSASPGSGSGGNKRSVSEISTNRPLKRAKSGSSAWAPDG

TGKGQQSLKGFFKPKTTAPEEAVGNGIAAAAHSRQHGKPGAEVVEQASQSPLYDSKSSGTRPTGISQGSV

QDQADVHDPVESKESWSKLFTKPAAPRCEGHGEPCVSLVTKKSGINCGRSFWMCARPLGPTGAKERNTQW

RCQTFIWCSDWNGLTRDDCGDGM

>XP_008080972.1 DNase I-like protein [Glarea lozoyensis ATCC 20868]

MEQRQGLRITTWNVNGIRNPFGYQPWRQSRTFAAMFDILEADIVVMQELKIQRKDLRDDMVLVPGWDVYL

SLPKHLKGYSGVAIYTRNSTCAPIRAEEGITGILTPPNSTISFRDLPEDQQIGGYPTASQLSDFSLGAAD

LDCEGRCVIVEFPGMVVIGVYCPANRDESRDDFRIGFLNALDSRVRNLIAMGKNVILTGDLNIIREEMDT

ANAEEQLKKHGMTGEEYVSTPARRMLNQLLVGGKVIGERDEDKEGAVMWDICRGFHPTRKGMFTCWEQKI

NARPGNFGSRIDYVLCNWKCYDWFRDSNIQEGLMGSDHCPVYAIFKDKVEIDGEEVDIRDIFSDGMFKNG

VRQRDWTIKDLLPTSAKLIPEFDRRRSIRDMFSKKPALLPEKSSVTNGASSLAVDRTTPTRELSQPENLD

PNGEPCFKPSVNKSPITPATSRPSPGRISGTKSPVKQNGKRGLDNGNGTDPPQKRTKASGTSKSVAANKP

PLTKGQSSLMGFFKPKLQPAAVLKDQDDSSTSFNSEMDIYDAPVEKSGVLDDLAALKPDENRSIPDSAAL

ADKTEGLSVENAFNPADQKDVVDPVAVKARWDLLGLGKRTVPLCEHEEPCKSMDTKKPGVNQGRSFYMCQ

RPLGPTGKKERNTQWRCATFMWSTDWKGENGQ

>XP_007913912.1 putative dna-(apurinic or apyrimidinic site) lyase 2 protein [Phaeoacremonium minimum UCRPA7]

MGLRITTWNVNGIRNPFGYQPWNQTRTFQAMFDILEADIVVMQETKIQRKDLRDDMVLVPGWDVYFSLPK

HKKGYSGVAIYTRNSKCSPIRAEEGITGVLCPPNSTTKFRDLPPDQQIGGYPEPNQLSSGLDEATLDSEG

RCVVLEFPAFVLIGVYSPATRDETRTEFRLGFIDAMDARVRNLVAMGKQVILTGDLNIIRSEIDSAGLMD

QLRKEGMTMEDFFSMPSRRFFHQLIFEGQVTGERDKGREEPVMWDICRVFHPDRLGMYTCWETKKNARPG

NFGSRIDYVLCSAGMKEWFQDSNIQEGLMGSDHCPVYATLADVIKLDGKDVRTLDVMNPPGMFEEGQRKK

EWTTKDLLPLSAKLIPEFDRRRSIRDMFSKKGGASKPKDQSAAAGSKKIPEQDLAPPTPLPLTTTPEAVL

DEDPAVGDDPFAIPQHPSRAIPITTPKRQASTVSDRPQKKQKPSLVKQGSKSTSGKAQSSLTGFFKPKTA

SPSKKESEEPESPFGTESPQKGRTSIPVLSLRGADIESDILAGLPVSWTGNPRKGRR

>XP_018186049.1 DNA lyase Apn2 [Xylona heveae TC161]

MSLRITTWNVNGIRNPFSYQPWRDQRTFTAMFDILQADIVIMQELKIQRKDLKDDMVLVPGWDCYFSLPK

HKKAGYSGVAIYTRQSVCAPIKAEEGIIGVLCPPNSSTCYCDLPEDQKIGGYPTATQLSVCPVDASALDS

EGRCVVLEFPAFILIGVYSPANSAGNRDDFRLGFLNALDARVRNLVSAGKHVIVAGDLNVSRDELDSANP

VETMRKSGLTPEEYISTPTRRLFNQLLFEGKVLGQRDEGREQPMLWDTCRGFHPERKGMFTCWEQKINAR

PANYGSRIDYVLCSIGIKDWISDANIQEGLLGSDHCPVYTVIKEKINVGEKQVHIYDLMNPNGVFKAGKR

IRSYDTKWLPALSGRLIPEFDRRRSIRDMFTKKPSLTAASTGRTSNALTPEAELHSLGSETLTPNSEPSN

LPSPERLKFPSSNGSTLGEKRDAQMPHQSQRPAKRSKSTAVSAASGPERGQKSLKGFFKPRSSGQLNGRI

DSKADDLSVPSSSLESSLQRSSSSTDDVKVQSNQFRAGFSFTKTGDSQDVSKTDSQSNTFRPPSLPSAES

ELKESQECEGVVDPIVTKESWSKLFSKPVAPRCEGHNEPCIKLTTKKSGFNCGRAFWICPRPVGPTGSKE

KGTEWRCQTFIWSSDWNARDSGP

>CCX30259.1 Similar to DNA-(apurinic or apyrimidinic site) lyase 2; acc. no. P87175 [Pyronema omphalodes CBS 100304]

MALRITTWNVNGIRNPFGYHPWSSQKSFQHMFDVLEADIVCFQETKIQQKDLTDDMVIVPGWDSYFTFPK

HKKGCYSGLAIYTRNSKCHPVKAEEGITGILESQGHAKKTYKSLPDSECIGGYPDLSNEEAVKLDSEGRA

LVLDFGAFVLIGVYCPAATDSARDHFRIAQVIVVGDLNIARDEIDAAGAKDMMKELGMRYWKETPTRKAL

DLLLDPNPAGQMVDLCREFFPHRVGMYTCWDVKKNKRPGNSGSRIDYILCSTAIKSWFLEANIQEGLMGS

DHCPVYATLKPTVNYNGIEKSILDIINPESMFHQGERQSDISTPQPPRLCMKRYPEFTRRQNIKDMFNKM

TSAATMNSGEKVQRHGSDSQGEPPLKKIRRDSAMMPFQRPRLPVNNTQKTLHGFFQHSPDVPNSSSRNST

AHKIHQPIRGELPMTNRERDLKEDTEGVVTESTINAKQNDFLDQTDSKKKWSLLFTKKRPPVCDGHGEEC

IQLSTKKPGPNCGRAFWANRSRWRVYNEVRSKIGMEM

>NP_595522.1 AP-endonuclease Apn2 [Schizosaccharomyces pombe]

MRILSWNVNGIQNPFNYFPWNKKNSYKEIFQELQADVICVQELKMQKDSFPQQYAVVEGFDSYFTFPKIR

KGYSGVGFYVKKDVAIPVKAEEGITGILPVRGQKYSYSEAPEHEKIGFFPKDIDRKTANWIDSEGRCILL

DFQMFILIGVYCPVNSGENRLEYRRAFYKALRERIERLIKEGNRKIILVGDVNILCNPIDTADQKDIIRE

SLIPSIMESRQWIRDLLLPSRLGLLLDIGRIQHPTRKGMFTCWNTRLNTRPTNYGTRIDYTLATPDLLPW

VQDADIMAEVMGSDHCPVYLDLKEEYEGKKLSNFLSHSKEPPLLSTAHHSAYRPSKNIHSMFQHFNSMKK

NKNNSPTQSENVSASASSGSSPTVSRANSVIDVDAYPPEKRRRKEQSKLLSFFAKQKEEKEETNKTEDVS

IEVLDNNNESDIGLTVKKKVENGNAWKQIFSERAPPLCEGHKEPCKYLTVRKPGINYGRKFWICARPVGE

LIKNSNAVSEEDTQPFQCRFFIWDSDWRANSKD

>XP_007872800.1 exodeoxyribonuclease III [Pneumocystis murina B123]

MRLISWNCNGIRNLFDYQSKKSEKSLKFILEKFEADIVCFQETKIQRKDLTHDMINIPGYLSFYTFSKKR

KGYSGVAIYVKESLCIPLKAEEGITGYLIHSGEGIPYIECPKSISIGGYTEMSDKHALELDQEGRCIILD

LNLFVLFGVYCPLNSNSERNKFRQLWIETLDSRIRNLRKMKRNIVIMGDINIIRDDVDSSYKIDELQEGM

EDFKDSCQRAWLNKLLLPHPEGFMIDLCRHFHPDRKEMFTCWNTKINARTCNFGTRIDYILISKELLPWF

EFADIQANVMGSDHCPIFAEIKSQIYNWKGSGKTDNILDFLNKEGAFVDGILVSKLPKPPHLETHNFPEF

SFQDIKLMFKKQEKLDSLEKKNKPYTLKNFLGRSATEPLSHFSKKKRLESFSNQSHLPSYFKNASGQLKN

LDRKHEKDTSLKKEYNSLDSENINTKTILKISDTLKNDTKRDSEVLELRKKTWNNIFTKPSPPLCNVHQK

PCIELRCKKPGLNFGRHFWICSNPISKEYKHQNISDSDMENKFKCGYFRWSSNHN

>XP_019021701.1 DNase I-like protein [Saitoella complicata NRRL Y-17804]

MRILSWNVNGIRNPFAYAPFNSMSKSYKLLLDHLDADIICLQETKIQMKDLTIDMVAVEGYDAFFSLPRQ

KKGYSGVVVYVRRERCVPVKAEEGVTGRLGMIWDKDRMYMDSTNAIGGYPPDSAYTMPPATLDSEGRAVI

LDFNLFVFIGLYCPANSGATDEGREFRRAYLKAVEDRVKCLREEGREVVVVGDLNVCRSGMDTAEGAKLC

KDEEWVNDFGRLWMTRLVGPPPSLDDNEEAEEAPRLLEDLCRKFNPEREGMFTCWNTRLNSRPNNYGARI

DYVLTSPGLAHWSSHADIRPDVMGSDHCPVYADFKHELLPESNEEEKEQRWLLDLVNPQGTFVQNVRVAP

PPPPPQLCAKNTGDFKGRQSLKDLFAKGASSACSGNASPVAAAESVPSNATFQAAAPLKRAASDALGSNA

GETKKTTKIPKITKDASPGQKSLRGFFTAKAPAPAVQQSDPPVKVSKSTQATIVATEAPANLDCVDICDP

PKTLAEAKPSGTITEADYAIIQPETTRSSWSTLFTKPTAPLCTTHNEPCTELTSKKPGPNMGRRFWICSR

PVGPGYDNGERPKGEVNREWRCNYFKWSSEVGRK

>XP_001882601.1 predicted protein [Laccaria bicolor S238N-H82]

MRILTWNINGVRTLPQYHPWNTLKAHDDILNHLEADIICFQEMKSSRPALPKQVAVPPSYDSFFSFPIRK

SGYSGVATYTRHSAVIPLKAEEGLSGVLQPKPPLTAAERISDSDNYPPNAETASIEGDLDYADLDSEGRT

VILDLGLFVLINVYCPNDGNGTEERDKYKMDFHRLLEMRVVGLIKEGREVMVVGDLNACAAIIDHCEGHL

MVAKGLAEGLQGEEGFWGKDSRRWMRGFLVPEDEGMPGGFMIDIVRKLWPERKGMYTCWNTKISARDSNY

GTRIDYILITRGLLPWVKAADIQPLIKGSDHCPVYVDLFDEITDPTTGAVVELRRAMGLQDDGRKDPPRL

AARFWDEYSGKQTQLHKFFGQKSKGSGSSDTPCPALVPSPSPIIVDDNNSATITPSSTPIPTTQTSTSGS

STTKSSPVPSSPLQQPSSSQPRPPSSSPPPVPISTKRKLVADIRSTSKKRKQREEEEKKPSQQSIASFFA

KPMRKPKDSLPPASSSIKAKGKQRAPEGDVDLEEDYQFALQLSQLNEPLVPSKSQSKNSKSHGGKGTAEW

TNLLAPIQPPKCIVHGELAKELTVTKQGANKGKRFFICARPVGPGYDKGRGERLREHVDPHWKCDFFKWS

SEVRREMVRNGNSGADVGVS

>KZP01650.1 DNase I-like protein [Calocera viscosa TUFC12733]

MRILTWNINGVRTLNQYHPWFSLKTLEAMLENEEIKSDIFCFQEMKIARTAIDRSIALPGHYDAFFSLPL

GKSKGGYSGVAVYTDSRTVKPLKAEEGLSGLLQPKPPLSHQERVSDNYPSAHLLDFIPDDKDHVPSDLME

LDNEGRALVLDFGLFVLINTYCPAEASEGRLPFKINFHNMLRERVRILIEEEGREVIVLGDINICSHPID

HCDGQLQSKRSVWWDHPSRVWLRDWLQPKGKLIDVTRKFWPERTGMYTCWNTLIDARTANYGTRIDYFLC

TEGLLPWIKHSDILPSVLGSDHCPVSLDLHEEITLTDGTVRRLQDEMRFDATRELPRISARYWDEFSDKQ

PLLSSFFGRKGVQSPDERGCGDVLLPPKREISEVPETPIDTPPPPDASSKTPSNILMGDTAPLPPKLVDE

VSSRRPKSPEKMSSQSKGKSKTSDKKSKNGQMKLSSFFGSKPSGEPSPSNESVSTDASSWRTVSQSTQQS

ANAVDDVEDDEYWANIIAQSGSSESSKAAWSTLLSRPTAPKCSGHGLPAKEFTVNKPGPNKGKHFYLCSL

PVGTGYDAGHSKRLREEVNPEFRCNFFMWASDVKKRAAPAQSSPETKRPRL

>XP_019001465.1 exodeoxyribonuclease III [Kwoniella mangroviensis CBS 8507]

MRILTWNVNVLRTCLDYHPFSSMKKKNVEGLLDELGAQIACFQEHKTPRAKLEKSMAVPGSYDGFWTFPR

SKTGYSGVCTYVDARYCVPLKAEEGITGLLLDDSKGSTMKPPWTPEERIGSYPDIDGMEWMDEVDGKEFE

PKRLDMEGRAVVCDFGLFVLFNLYCPNETNETRRPYKMNFLKCLQERVRLLQQAGREVIIVGDINIMRAP

IDSGEGGIRTSAEQHYEHPARRILDDWCAPKGPMVDVVRESWPNRDDMFTCWNQKLDARSANYGSRIDLI

LCTPGLRPWIKGGDILNRVYGSDHCPVYIDLHESIDHPEKGELHLKDMLNPPDRPPSTAPVYPNDIPREA

PEPPRFATKFFDEFSGRQTTLKSFFGGGGNKKKDVAIHPTPSPTPATPSRSSATPVPSENTYANTPLTLQ

NTAKTSPSTENGASLATPFSLARAAFDSIDTSTQITASSNSITLPSPPSVIHQVSPQRRRSPTYDAIDMT

HDDDDIHKATSNAIAGPSKPKPTIKPKSNKLSQAKSANTTGSQTKLSSFFSQPPISTSKRKSSPSPSSND

TVQPKTKSARKSISSISPSRSPSIPKQNNHSEEVASGWTEQEDELINQAILEAERDRKAKNDSAKPVWGE

LFAKKLPPMCTVHNKPCKDFLVMKPGPNKGKRFWLCSLPVGAGYDTGRSKRPREDVNRNFRCDFFLWDSA

NSRKEKVKDLNNNEGEKESQNIQ

>XP_016275748.1 AP endonuclease 2 [Rhodotorula toruloides NP11]

MRIVTWNVNGIKTLLQYHPWNQKRTYEGIIEDLGGDITCIQETKITRAQMERGMAVLDAYDSFFSFYRRS

VRGIHGTAIFTKRDVVIPVKAEEGIASSLIPSTMAVADRIGGYPLSSEADLDYNAMKDLDSEGRTTVIDT

GMFVLINLYCPNETNSDRLVFKNNFNTLVDRRVRNLIKLGREVIVVGDLNICASDLDTAEPEQRARDSGL

ENFTDHPPRAWLGAFTGPNGPMIDITRRLHPDRQGMFTCWNTKIEARVSNYGTRLDYILITPGLLPWVKF

SDIQPKIVGSDHCPVYLDMYDELDIPGRGKVSIWDELNPGRKKGDKLPDPPAFASRFFSEFSGKQKLLSN

FFTKKSEMPPDASKPSPSPSPQPNSATTSASVSKNGSLSTMATITAVTNGSSKKGKGKEKSPEEEKPKTG

QQGLSSFFKPPPAPDARPTKKRKKSQTEPTPPSPSNSRPSTSRSSSSSQTKPSPSPTASFRNGDGTEVLV

LSDGDDDAEEELDLKEREDATADPGFNPNAQSASAWSSIFASKPVPLCEGHSEPSRVWTVNKPGPNKGRR

FYLPVGPGYDQGQSKIHVNPEYRCNFFQWETKVKRPAGMVLSSSAKKQKT

>XP_014569941.1 hypothetical protein L969DRAFT_92556 [Mixia osmundae IAM 14324]

MRLITWNINGIKTLPQYKPWNECKGNYGQMLDLLGGDIVCLQETKITKDKIERSMACPDDYDAFFSSYCR

KPPKGYSGTAIYTKRAATVPLQAEEGLTGLSLSGEKDRDDLARQDRIGGYPTPAAVELSSFEMRDIDSEG

RTTVVDLGLFVLINVYCPNETNSDRLTFKMAFNHLLDERVRQLIKLGREVIVVGDLNICAQTIDHCDPEK

RKAEQNLDDFQAHPARAWLSAFIGASGVMIDSTRFFHPTRTNMYTHWETKINARVTNYGTRLDYILVTPG

LLPWIKAADIQAHVVGSDHCPVFIDFHPEISLNDGTKQSLWEAVNPGRERVDPVPDPPRMAARYYNSFAG

TQSLLKTFFVKKKDESPVQARTVTPLPAPEMRRDASTSRVSESPEAPTLASAFATIRSSKPERHATIMIE

DDDDDDEIAEHPQPRSRSTSTATAQSSRPPDKRPRSDSASRSQPPKKQLNVRNGQSTLASFFADPVSGKT

RRPESDTKKRRSTVPGSQSENLDEPSCSASTSQRASATASETAAQADGYERLPLQDSDPQAASQWNALFS

PLAVPLCKMHKEPCTLWTVNKPGPNKGRKFYLCSRPVGQGHEEAASKNKNVILNPDYRCNHFEWSQSMGH

VLAPDGLSYTVFDSEEVQQSQPFVPYAPYHGYHVRAHSSGDEYARSRQSTISQGSVIYAMPADHQQIVPP

GMLPTIPQGMQYYNVCHQCQRNPCACRPMYSSQHVYDIPTYDSHWYAPWPAAPMSNSWPPQVSWRAQRPD

VVSSQIGRVHPKRPPRQEASKFQAHQANQPVSGTRFVPDYRPPPLPSAWAMWVGNVSRDTTHEQFSHFFL

HLPTPQSYCGSEEIATARSELRKRGGSLGHELDAAALSTTGVDSVHLLLHANCGFVNYISALHLKVGIAT

GNGLQLRPGCKGSKPLVCRERDDGDKTGVTSQHGFAVARSTWKTQPRNETTLNRAYRTSQDVFLIFSANG

SGQFLGVARMSGSIAGSSNVNALTDKLRDEIATPTAISHTDGDVLSPDGGVPGMVPTNSPVVEEPVIISP

GVARDLAPNLEKQDLSVANMAGRRPAESAATRRAREIAGNSSTSTPTSSNTGGGSSQATNTTTATSLASL

VISPEKDEPVHRKAADQLSPEPSRYFALEWVAVRPLSFASTKHLKNAFNRNKEAKIGRDGTELDPLLGDA

LLRAFVE

>XP_011386696.1 hypothetical protein UMAG_10924 [Ustilago maydis 521]

MRITVWNINGLRTLKGYQPWYKLPDWEACLDHLGADIACFQETKMTRKQLTEPMCILPSYNAFFNFHPTK

GYSGTVTYVRKSVSIPLKAEQGITGRLSVTNVSTNPLIPNDPIGCIPADTRDDVDAQIWNALDEEGRCVL

LDLGLFVLFNVYCPNETGPERLEYKMTYYQCLAERAHRLIQEGRQVMIVGDMNIIRDPIDHCDAEQSIKE

HGWDDFHQHPARSWFQSFLAPHGKFHDVGRMYHADRKKMFTCWNTLIDARPANYGVRLDYTLATEGILPW

IKGADIQADVYGSDHCPIYIDLYDEREIDGKVVRLVDLMHGGSNRLPPALAACHYDEFSGKQRKLASFFC

ASRKAASAESNNPSNLVTKEQRQMPINAEGSADVKSLNDGFATSQASSPTKVDTESAGSSLVDSLFALHS

QQEGLDMQDASQARNSASALSSVVTAQQVLASTAAVAPSASAPAATTAALSLSPTRVLASRSASNSKRDS

ASSQTACAGSKGKDSIKAKTRASQRGQTKLHSFFAKPKSLAPVATNETSQPIDVSSSDGMVAPKTSTSGN

TVDNTSTDPTRDVTKRHGPNVDEAEDAICDLNEFQATTPPTQPLPPNEQSAADRVGTSLAWGAIFSPMPA

PLCRNHSEPCRAWTVNKPGPNHGRKFWLCNRPVGPGYEKSGRAKGDVNPEYRCNFFVWDSDLRSKKNKPG

RESKDEKFFSEVRKERGKKQNGQQTGDELNGPWGPHKRVRTGDT

>XP_013241303.1 DNase I-like protein [Tilletiaria anomala UBC 951]

MRIVSWNVNGLRTLKGYQPWYSHKSFAESLAHLKADIACFQECKLTRKALLSEASLSQGAASTCVVEGYQ

AFFNLHPDKGYSGTAVWTRSDVCVPVKAEEGITGRYAQEGGPSGSITAAIMGSSGVGLREADRIGFEPCD

MKDDMDIQLFRSIDNEGRATVLDCSMFVLFNLYCPNETGPERLPYKMAFYKCLEERVRRLVEAGREVIIV

GDINICRDEIDHPDPEQSMKDHSLPTFKAHPARAWFDNFLAPNGSFIDVGRHLHPTRRKMYTCWNTIIDA

RPVNYGTRLDYTIVSPGLMPWVKSADIQPDIYGSDHCPVYIELHDERFIDGQTFRIKDLLHAAPANGTVK

APPIACSSWPEFSAKQRKLASYFGSGAGAGMKRSASTASRQPASRPALSFLAPITKESASSGRGFSTPAA

TSTEADGVGASLATALADALFEVPSAEPDYIAKGSGFALTRPANGPCSSTWPTVPSGAAATGSRHGTASK

GPRLSQSKPRYASSSDKVFSEALPKKSGGKPSAAPLSKGVAKTIKPTAAAQKTSAHSQLSLKAFLPKRNV

AETPLEGKSSTRDDEVLASNSDRGKEARAKDTSMVAEQSSVEQITPLDSALTVRADEQAEAEARTDILNA

SRLIDDEVIPISPDKEDGVSTYKSAMAWGQIFTPPPAPLCSMHQEAARAWTVNKPGPNHGRKFWLCNRPV

GPGHEGSGISRDRVNPQLRCDYFSWDSDVKRRQAKASKFVLPVSDQNQCEAAKAFAEVGRCRKDFEAQRE

HPRESQQAHKRVKKN

>XP_017990464.1 ap-endonuclease apn2 [Malassezia pachydermatis]

MRLVCWNVNGLRTLKGYQPWYQLPSWEACLETLEADIACFQEVKMTRKQLTHAMCIMDKYEAFYDLHPTK

GYAGTVIFCGITGYGPGDVIGTPAQTLQETVDPALFQALDQEGRCVVLDCRLFVLINVYAPNETGPERVA

YKMAFYHALEERVRHLIAAGRDVLVVGDMNVVASPVDHCEGALMPPVDFIAHPARQWFRAFLAPSGPLVD

VTRTYHPDRMKMYTCWNTLIDARPANYGTRLDYTLATPSLLAWVRDADIQPSIHGSDHCPVYVDFHESIS

TDQGLVQLPEQLGGTDLTYTIPSMAASQHGAFSIKTQPRLAAMFGAMQERQRIRVGENMGTQAEPTSQSS

DTQSRMAVPMPEKRRAKGRPAKSISSQTTLRTFFQKRAPETKVVEAAQHVVKTTPIPSTRSSAQQRAKAK

EAWGQLFTPTPPPLCRVHQEPAKSFTVNKPGINQGRKFWVCARPVGPGYEGNKHGSVVDPQYRCDFFAWD

SDVRRQRR

>XP_016613110.1 exodeoxyribonuclease III (xth) [Spizellomyces punctatus DAOM BR117]

MRILSWNVNSVRTLRQYHPWCEHKNYQTIIEALNADIICLQEMKITRSSTDSDLALVPGYDAFFSYPKRR

HGYSGVTTYVRTDYTPIEAEEGFSGLLSKSSGIESKIGCYGQLWHEFSPAELLELDSEGRVVITDHRLFV

LINIYFPVDDPSEERQAFRFKFYHAIRLRIEALIAGGRQVVIVGDVNTTHQEIDHCDPRKSMRDLGISCF

HETPTRRWIHELVQPRGPMYDMFRYFHPTQEKAFTCWNTLLNARPVNYGTRIDYVLATEGLIPWMADATV

EADIMGSDHCPVAVTFHDTNPVNGDCLKNVMAPPDWKPGDKRDPPRLCAKFWDEFTGKQQKLSSFFNKKS

TSLSGGSTTELGETRSGGITQEERKETIIATNGKGGTLEITTPPSESCAQPSTISVAAPPAVHAQPSQST

LPARSGPSKLRAPTRKGQKSKQKAIKVGVKVPPSQGQSSISSFLLPKTTTAGTAKPIVGEPMCDATQDLP

STSLETPPSLEDAQPLFENPLACSSDPGVDLDVPTTGRLSQPTTLGTMSASPNSSTDLDQGDSTSSAGTW

STPNAASQWRSLMRPKVIPKCWHDEPAKEWVVNKTGPNQGRMFYLCARPVGPSDIGLLNFEGKSVIVPKS

RRLVGEYRCDFFEWKNGPKRKGQQGGRIRSATENLNGEDTRQGEDSSLKKSRTK

>KXS18871.1 DNase I-like protein [Gonapodya prolifera JEL478]

MTWNVNSFRTMRGYSPWTECKDYKELLDALGADIFCFQEMKVTWDKLDAEIALVPGYDAFFSISRGRGGY

SGVATYIRLDRVPTPIDAEEGFTGLLSRPGAQSKLGCYGDLEEEFERDELVQLDSEGRCMITDHEAFVLF

NVYFPNDHDAEREEFKLKFYRGIEIRVRALVDSGRQVIVAGDVNAVHMPIDHYNPNCGSFIGPIESHPAR

AWLNGFLQPRGPVVDLFRYFHPDSQGVYTCWNTKVDGRSGNFGTRIDYILPTPALIPWFEDCTIEASIRG

SDHCPVVAVLRDSIETEEGEKKLLDILSPTTYDLKGNLVVREPSKLCARFYDKFLAKQQTLKAFLVNKGP

NALAEMEAKPPKGLTAMETMSGGLKGISGTKMSRPLSGGTSAPPAKKAKTAPVPGQKSLLSFFRGAKEES

SNTSGSPSQSGVPSGERNPVKLVSNSIRPPNPSSAPPAPMVASLQLEQDGSRSRMRESGEGNSQSSDYSF

VDAESIPDMTDLADADECGTELSPQEFGASQAAAWGAIFTRPKPPQCRHGEEAKLYSVTKKGPNQGRQFW

MCPRPGPTGMAKELAVNKNERDTFKCDYFMWYRDSARRKMERRINSR

>EPB84226.1 hypothetical protein HMPREF1544_09018 [Mucor circinelloides f. circinelloides 1006PhL]

MRVLTWNVNGLATTLQYHPWSETKSYKVLLDALDADIICFQEVKCQRSKLTREMALVPGYHAYFSFSKVK

LGYSGVAVYVKDTVMQPRRTYEGITGVLDADNDFEYDFSTPAKLLDDEGRCIILDFGFFILFNVYFPNDS

NESRIDFKMDYHHCVQKRIESFLARGKQVLLVGDINAVHEEIDHCDAKQSMKDHDITDFKDLPHRRWLDN

MIDPKGPLIDMTRLYHPGRLKMYTCWNTRMNARPGNFGTRIDYILASKGLKPWFKYSDIQPDILGSDHCP

VYADFLPINQDIIADQDESVYISPLLSSNFPEFKQNKLSNYFAKPSSQPLSSQPLPMQPVSAPPISTLKR

VPSSSSTSNKLQPSKKLKSTKKAAPKNTALMNYFTKNTTDTDTISNTTTTKQQLPQKQDTQQQEEDVDLD

ALIEEAQEKQVTAKAWTSIFKAPEIPTCTFHKLPCVERTVSKKGPNSGRVFYICSKPTGPKDGPREEYSC

DYFQWKQQNNSNTRGKG

>XP_021877736.1 Endonuclease/exonuclease/phosphatase [Lobosporangium transversale]

MKFMTWNVNGIRALTQYHPYCDDLHKNYKEILDYLDADVICLQETKITRAKLESDIALVPGYDSYWSFHR

TKSGYSGVVIYVKDHIKLLAVEEGISGVFSENVSLQPPNAMVPLNTQTCSNSSKAKGVGGYPSLDDGDSA

AIFARYQELDSEGRGLIIDFGLFVLFNLYCPNETDETRLPFKMDYYHLLESRVQALIKEGRQVIVMGDMN

VVPTELDHCDPAKWKKETGESDFTNTLPRRWFNSFLAPAGPMTDLYRIFHKDKPGAFTCWNAKINARPSN

YGTRLDYVLVTKGLLPWFKACDRLPHIVGSDHCPVVAEMFTELVIENSTQEKEQDQKAFQKLHELLESYG

GTTEHSLAAKYYEEFSGKQQKLSAFFKKPSSATLKPITGSELKRSSPLDDHEDQLPRKKSYFSNDQVVND

DPSPKTDTRLSASNVSSLLPLSNSVPSTTLPSQIPKINNKITTKAKSGHLIQDKSSFKKPVNPRSSGQQS

VLSFFSKAEQEYINESQLSNTSSSPPHSQTPDSSFSSATSAPSSLSSTFSPSDFADWIPGSQDILPFSTN

GEATTSKWQSLFQPKTIPKCRVHGVPCTEHTVNKKGPNKGRRFFLCSLPVGPEDDTIRPRPEYRCNFFEW

RKSERKK

>ESA11580.1 hypothetical protein GLOINDRAFT_347717 [Rhizophagus irregularis DAOM 181602]

MRIITWNVNGIRSITNYYPWSKHKDYKQVLDTLNADIICFQETKITLDKLDSSLAIVPGYDAYFSSSRGK

GGYAGVVIYVKTSVIRPIAAEEGISGILNEVPANAKSKDVTTAGIGPSIISEFTTEELLEIDSEGRCVIL

DFGIFVLFNVYCVHESSQGRLPYKLRFYNILQRRAEALLDSGREVIIVGDLNVTHRVIDHCDPQKSIKEH

GLKSFGDHPARKWLDSWISPNGPMIDLCRKFHPDEEGLFTCWNTLINARPINYGTRLDYILVSEGLTKWF

KSCNVEQDIMGSDHCPVSCELFDEIYEGDNKLSLLKENTLMTLTPKLCAKNLVRFSGKQQTLKSFFIKQE

IKEKEAQERKSVKTSNDNIVENIPETLTSNTIPTDSSIDISEGLKEQSEIKRKSGQASVIKVQKAKANSS

KLIKEKSSKAQAKSKLSYPTNQVSLISLFKKASQDTPPEKKLEEVTKSIELKDSQGLQEKSTISENISDS

SNSFEESLDDIINNVEDECIEVSSSNNKSDIQSQWNALFKPRPIPNCIGHGEPCKEYTVNKPGINQGRRF

YLCSRPVGNSQESRCKFFEWTNGNKLKLSKSVKRYSDQKQDNGLNKKKRDF

>ORX89359.1 DNase I-like protein [Basidiobolus meristosporus CBS 931.73]

MRLLSWNVNGIRTLTQYYPWCECKTLKEVLSRLNAEVICFQETKLSANRFTSDIALVDGFDGYFSLSKNA

SGYSGVATYVDSLNVFPTNASEGITGILHRKGVGEPLLKELLLTTEQFEDRKELLDLDSEGRSVVIDLGL

FVLFNLYCPNERGPERLPYKMRYYHALEKQVDHLLNSGRNVVIVGDMNISHKEIDHCDPLTATRDYGLEN

FGDHPSRQWFDQFLSPRGKMVDLFRHFHPGEEKKYTCWNTKINARPANFGTRIDYILVNEGLLKWFKSCD

ILPELMGSDHCPVVAELFTEIVDECGNTLLLQNFFKHSQTNESTTKYKAPSLCAKYHSAFSGQRKLTASF

FKPANKSKVSSTFDRPSINPPITTQKTTEETYNVNATSKLDKLNSPCKKDAPKQSTLQAFFQAPKRTESR

ATATSVKSELKCKGDLSDREPPLLAEYMDIVNSAVATTSLNADTTKQQWNQLFSSPDIPVCKHGEPTNEF

TVNKKGPNQGRRFFVCARPVGPNNAKDPKFRCDFFQWKNGKKRSSSLESPSLSGDSSSKRNKS

>XP_002140854.1 endonuclease/exonuclease/phosphatase family protein [Cryptosporidium muris RN66]

MTFSIVSFNVNGFIPCLIRKGYKENEISSFLDEIYSSEVPWNGVELPRPSIVCIQECKMNSEEDINYCTG

CPNRHHAFYSLPTNYKRYSGVATFCRKDEATPREATSGFSWIEKEDKIFKIEDSEIYSDEIKEFNYEEIL

KEPKNYRKFDYSLSDMDIEGRCIITDHVDFLLVNLYLPLVRKVLADNFENTDNLASLTRASYRLAFNQYL

RLMLDVITTKNKRDVILVGDMNVTLDDIDSYYEYNTCKKDRVGHFKELEDLMDISSSYSLFYNQIRTDMK

LLLRDFNLIDAYRLFYPGKINKYTCWDQTKCSRINNRGSRIDLFLVSRNLINRVKGCEIIDNVCGSDHCP

IVLIMSGNLQNINPNTPPSLCSKYLPSCILKQSLLSNFFVKEKPNDLQRPVLEKDRTSKKLILDNQDKDI

PNCKHGMRCIKKYVSKNGINKNRSFWTCSKSDLLKCNYFEWVKEKRQLCSITTFVKQNQKIN

>XP_008892235.1 exodeoxyribonuclease III, variant [Phytophthora parasitica INRA-310]

MKRKLRLVTWNVNGLRAVLQRLEQNLQQFLESLEADIICLQESKLTRSELDEELVRPPGFDAFYSFCRHR

GGYSGVVTFVKSDLPTVAAEEGLTGLWKTKDNVGHVGSLHNELPSKLVNDLESEGRCIITDHQAFVLLNT

YCPALASSDRLEYKLQFHALLEDRVNALRAANKRVVVVGDINIAHREIDHCDPQAHRSDGNSFGDHPCRK

WMDSFLGSSDETSLSEPNRDEITGVTGVKLIDTYRHFHPDQAKAFTCWNTQTGARQTNYGTRIDYILVDP

TFVECVSTCSIDAERVGSDHCPVAMSCTVELETDSSTNIGITAVLCAKKFAEFAGTQQSIKSFVVQSRSV

GAENLSGQSVTSPVRSSLRSKKRGQQSITSFFSNSGTKRKAPSYREVSWSEESSTYRMFSESVRIVKTSE

NRKPAEKEKLEWAQVLNGRPPPTPLCHCGQPTVLRSVLKTNENWGRKFYVCTKPAVSSI

>XP_002992015.1 hypothetical protein SELMODRAFT_134604 [Selaginella moellendorffii]

MRIVSYNVNGLRARLSPARSLRAFLDSLDADIICLQETKIRRQELTADIATPQGYESFFSCTRTVKKGRL

GYSGVATFCKVECGNNVALPVAAEEGFTGLLTSARAGEGDKEFWLGSYDPVLTVEGFTRQELLDLDNEGR

CIVTDHGHFVLFNVYGPNVGCGDAERQDFKLNFYQVLQCRLESILKQGRRIIIVGDLNISPYPIDSCDPG

PEFDTSPSRQWFRSLLVSEGGAFSDAFRVFHPERAEAYTCWSQASGAEEFNYGSRIDHVLIAGPCAGHCQ

SPDGSHGNSSCDGFAKCGTDMCDILLEFKRAKLDTLPRWSGGRSLKLDGSDHAPVILQLKHLPSIPPHEA

PQLAARFMPELRGRQQSIVSIFQKLNDKATANTEPDTSRNVSKPAFTQETGSRKLQRLSQSRLTRFCYQR

HESSDKMSCTEVAVHGQSSSLTDEIQQSPESCDNNNCSEVAACGESASLMDMSDETPTESSQQSLEKSAA

GWERLKSLMSRNLPLCKGHGEPCVVRTVKKAGPNLGRGFYVCARAKVIFLERFFIIYSLFRAGPSFQS

>XP_001781868.1 predicted protein [Physcomitrella patens]

MRIVTYNVFSLRARLVPPRTLKSFLDSLESDIICLQETKLTRQELTAEIAVADGYESFFSCTRTVSKGRV

RYSGVATFCRTPTAVPVAADEGFTGAQSSTHQTNYKNDHARVGCYEEVLNMENMSRQDLLQLDWEGRCLV

TDHGSFVLFNLYGPSVGPDNEERYEFKLRFYRALQNRWEGLLKNGRRVIAVGDFNISPFPIDSCHSDSNP

DFDKSSIRQWFRSQLRANGGPFVDVFREIHPIREGAYTFWNQMSGSEEFNYGTRLDLIIAAGGCFHQVRS

EESNSLLKAEESHHFGTCEVEDCDILLEFKRFKADSVPRRGGEKTQKLDGSDHVPVYVQLRPQPPLEQHD

VPPLAARFMPEIRGRQQSIASFLQKRSCSLIMDIEAQKETKPRLSLPTATCVRKNPPKVTKFLKSQNSKQ

KSLHSFFMLPTTKGKENTEAANAFKLAFQDSDSETLLKPSLEQSQTFVINSPHDGDSQETNAEPTDSSNT

SSLKQDCTCESQNHVPEAVETVETAETNEKLSAKIEWQRIQKTMMNRVPMCSGHNEPCATYVMKKPGPNH

GRKFHCCARAQGPSSNPEAKCKYFKWQNKYK

>XP_002506628.1 predicted protein [Micromonas commoda]

MAPPPSDSDSPNVTLPEALTGLEGDAIVSWNIGLRGLRQLVASDRGADKTAAKDQHGVSRQLGYGCIDSL

LDSLGSDVDVVALQECKLTSRSDLSPEIACPSGWDSYHAVCGDASGGAIPTTSRGRSKTGYAGVATYVRI

GKRVVAAEEGVTGALTPPERIARGDAVGHYGRMSEAFDRARMLELDSEGRCVLVDLGAFVLFNVYVPSLS

SDDEDRERFKLDFLVAVEIRYRALLDAGRRVVLCGDWNVSYGVIDSAVAIEERLMDEALCERNQSRQWLR

RQVSGGEGAKGAERDPLVDVFRRDYPAARGAYTCWNVSAGAQLTNYGSRIDYFLCDEVTAASVARCGIAP

KHEGSDHAPVFIIVRRGSDCSLKDTGNEGNAVNPLASSACVAAAGRQARLTDSFFKAGAGAPNDIWGSAE

TVGGGVHAMANANGGDRGCLARSQPALAGVKRKHGGGGAGGAASIKSFFGPKDVGKKPTSAASADDTNPP

PDVPIDLGTRDVPAQELTPAPTASIETVAAWKAIQKRMAPPKCRGHGLPCKVRTVKEGANKGRGFFCCPK

PKGMKGDKNADCGFFQWEKVYRK

>WP_010875851.1 exodeoxyribonuclease III [Methanothermobacter thermautotrophicus]

MTVLKIISWNVNGLRAVHRKGFLKWFMEEKPDILCLQEIKAAPEQLPRKLRHVEGYRSFFTPAERKGYSG

VAMYTKVPPSSLREGFGVERFDTEGRIQIADFDDFLLYNIYFPNGKMSEERLKYKLEFYDAFLEDVNRER

DSGRNVIICGDFNTAHREIDLARPKENSNVSGFLPVERAWIDKFIENGYVDTFRMFNSDPGQYTWWSYRT

RARERNVGWRLDYFFVNEEFKGKVKRSWILSDVMGSDHCPIGLEIEL

>OLS27085.1 Exodeoxyribonuclease [Candidatus Heimdallarchaeota archaeon LC_3]

MISWNVNGIKSTIDQGLIEFVKKEDPDFLCLQEVKTHYKELDRLLPNYKSYWNPAERKGYSGTLIYTKIK

PLKSKKGMGIAEHDTEGRITSLEFEKFYLITVYTVNAKRGLIRLDYRMRWDKDFLKFIIKLDKKKTVIVC

GDLNVAHKEIDLAHPKRNKKNPGFTQQERDGFSAYLDSGFVDTFRHFDQSPEKYTWWSNFSNARERNVGW

RLDYFLASNRLMNSVKKSEIMSEIYGSDHCPIKLEIEI

>AMD29902.1 exodeoxyribonuclease III [Candidatus Nanopusillus acidilobi]

MKIISWNVNGLKSIIQKGLIDFIKKYNADFYLFQEIKTKEIPLTFQLLGYNVYSFPAKKSGYSGVLTLTK

IKPNNLKYGIGIEKFDIEGRVITMEYDKFYLINVYFPNAGVGTLKRLDFKLEFDKEFEKYVLSLNKPCIV

CGDFNVAHKDIDIYDPINLKGYAGFTDEERQWFDHFLSLGFIDTFRYIKGNIRKYSWFSYSIIARKKNEG

WRLDYCVVSKELKDKIVNADILVDIFGSDHVPILLEINI

>WP_088820613.1 exodeoxyribonuclease III [Candidatus Micrarchaeota archaeon Mia14]

MELKIISWNVNGIRSATAKGLTDFIKNEDADILCFQEIKAGESDIPQALRSEGYELFVNPAEKKGYSGTM

VMSRIKPLKFQKGIGESRDAEGRVEVLEFEKFYLLNIYFPNSKPMLERLDYKIQFDHELLNYLNDLKKRK

GVVACGDFNVAHKDIDIAQPQNNINHAGFTKEEREWMDEVISSGYIDTFRYINGNKIKYSWWSNFASARQ

RNIGWRIDYFITDTSLKENIKSAEILDQVKGSDHAPVKLTLSL

>OGJ21336.1 exodeoxyribonuclease III [Candidatus Pacearchaeota archaeon RBG_13_36_9]

MSMKIISWNVAGINACIKKGLIEFIRKENADIYCFQEVKASQENFPEELENLGEYDRYNVFADKKGYSGV

SILSRIKPLNVIKGLGKEDFDSEGRVLTLEFDKFYLINVYFPHSNRKLIRLDFKLNFNKEFLEFCNQLEK

NKPLVIASDFNVAHQEIDLKNPKQNKKNAGFTTQEREWFDAFLKQGFIDTFREFVKDGGNYTWWAYRNNL

RERNVGWRIDYFIISKKLKDNLIKSEILKDVFGSDHCPILLEIK

>EEZ93294.1 exodeoxyribonuclease III [Candidatus Parvarchaeum acidiphilum ARMAN-4]

MKILSWNVNGLRSVARKNEIQKIINSQIYDVILLQETKIEEINKLIETNGYVYYIMHSTVKKGYSGVITF

CKETPINVIYGIGHEKYDNEGRVITIELKDYFIVNSYFPNSRRDLSRLDFKKDFNEKILEFLEKLRKRKP

VVIGGDFNVAHTDLDIARPKQNDGNAGFTREERAFVDKFIEKGYIDTFRLFNKEGGNYSWWTYLYKSAKA

NNIAWRIDYFLVSSELKNKVKAAGIIKKQEGSDHAPVFVEVD

>OIO64153.1 exodeoxyribonuclease III [Candidatus Woesearchaeota archaeon CG1_02_57_44]

MRLISWNVNGIRAILNKGFGAFVARERPDMLCLQEVKATQEQALGAGLLTSVTALAGYHVLWHAAERPGY

SGTAVLTKVSPTHTAFGIGSQHDTEGRVMTLDYGSFYLVNCYTVNAQRALTRLPERQEWDRTFKAHLAAL

DRKKPVIACGDLNVAHEDIDLARPGPNRGNAGFTDEEREGFSALLSAGGGFVDTFRHFTKDGGHYTWWAY

MGNARANNVGWRIDYFLTSRRLMQQVKKSVILPSVMGSDHCPVMLDIAL

>WP_010878084.1 exodeoxyribonuclease III [Archaeoglobus fulgidus]

MLKIATFNVNSIRSRLHIVIPWLKENKPDILCMQETKVENRKFPEADFHRIGYHVVFSGSKGRNGVAIAS

LEEPEDVSFGLDSEPKDEDRLIRAKIAGIDVINTYVPQGFKIDSEKYQYKLQWLERLYHYLQKTVDFRSF

AVWCGDMNVAPEPIDVHSPDKLKNHVCFHEDARRAYKKILELGFVDVLRKIHPNERIYTFYDYRVKGAIE

RGLGWRVDAILATPPLAERCVDCYADIKPRLAEKPSDHLPLVAVFDV

>WP_012194374.1 exodeoxyribonuclease III [Methanococcus maripaludis]

MKMISWNVNGIRACLKNGFMNFLERESPDIMCIQETKVQSGQVQLGLDGYFQYWNYAERKGYSGTAVFTK

IKPNEVIYGIGNNEHDGEGRVITLKFDEYYLVNVYTPNSQRGLTRLEYRQKWDQDFLNYVKTLENKKPVI

FCGDLNVAHKEVDLKNPKTNVKNAGFTPEERKGFDNIVNSGFLDTFREFNKEPDNYSWWSYRFNARARNI

GWRIDYFCISESLRGNLKDAFIMPEIMGSDHCPVGIIFG

>WP_011035046.1 exodeoxyribonuclease III [Methanosarcina mazei]

MPEHYNLISWNVNGLRAAVKKGFLDLLLEHRFDIVCVQETKVSQDKLPREVKNIQGYYNYFVSAEQNGYS

GVGTFSKNKPIKLEKGMGIEVFDREGRFLRTDYEDFVLLNIYFPNGKASQERLGYKMAFYDAFLDYANAL

KSEGKKLVICGDVNTAHKEIDLARPKQNEMISGFLPEERAWMDKFLAAGYLDSFRMFNPEGGNYSWWSMR

TGARSRNVGWRLDYVFVSENLRENVKSASIYPEIMGSDHCPVGLELEF

>WP_010901905.1 exodeoxyribonuclease III [Thermoplasma acidophilum]

MLYKFLSWNVNGLRAAVKNGAVSVFKQDDYFGIALQETKADSTSVPEEMYHLGYYLYNNPAKKKGYSGTM

SLVREKPIDVSYGFENEEGRILNLEFDKFYFINVYFPNAQHGLTRLDMKLDFDEKFLEYSNELRKKKPLI

ICGDFNVAHEEIDIARPKDNENNAGFTKQERDWMTKFLDSGYVDTYRIFMKEGGHYSWWSYRFNARAKNI

GWRIDYFVVSDDIRDRVKKAEILETVTGSDHAPVTLEVDL

>WP_008086108.1 exodeoxyribonuclease III [Aciduliprofundum boonei]

MELLLISWNVNGIRACVRNGFLDFLEKYKPDILALQEIKATEDNVPMEVRYYPDYHKYWNPAKKKGYAGT

ALFTKIEPLNVKFGIGEDKFDSEGRVITAEYEKFYLVNAYFPNSQHGLTRLDFKIEFDKLIHSYLNELRK

KKPVILCGDFNVAHKEIDLANPKQNVKNAGFTPQERAWMDKFLQDGYIDTFRMFTKEGGHYTWWTYRFKA

RERNIGWRVDYFVVSEELKDKVKSSWILSEVYGSDHAPIAMVLDI

>WP_012716949.1 exodeoxyribonuclease III [Sulfolobus islandicus]

MKIVSWNVNGIRAALKKNLIDFIENNMFEVIMFQETKGDIVPLDFIMMGYEVISFPAKRKGYSGVMTLTK

IKPINVIKGLQIKEFDDEGRTVTLELKDFYVINAYFPRAGDNLERLDFKLKFNNEIENFVLKLRKAKPVI

LCGDFNIAHQNIDGAFSNPTIPGLTPQERSWFSHFLSLGFIDTFRYLHPNVRKYSWWSYMGKARDKNLGL

RLDYCIVSEELKDRIKMADILIDIQGSDHAPIILELT
