# Supplementary figures and images for: Characterization of biochemical properties of an apurinic/apyrimidinic endonuclease from Helicobacter pylori
Source: PLoS One. 2018 Aug 15;13(8):e0202232. doi: 10.1371/journal.pone.0202232 (PMC6093668; doi:10.1371/journal.pone.0202232)

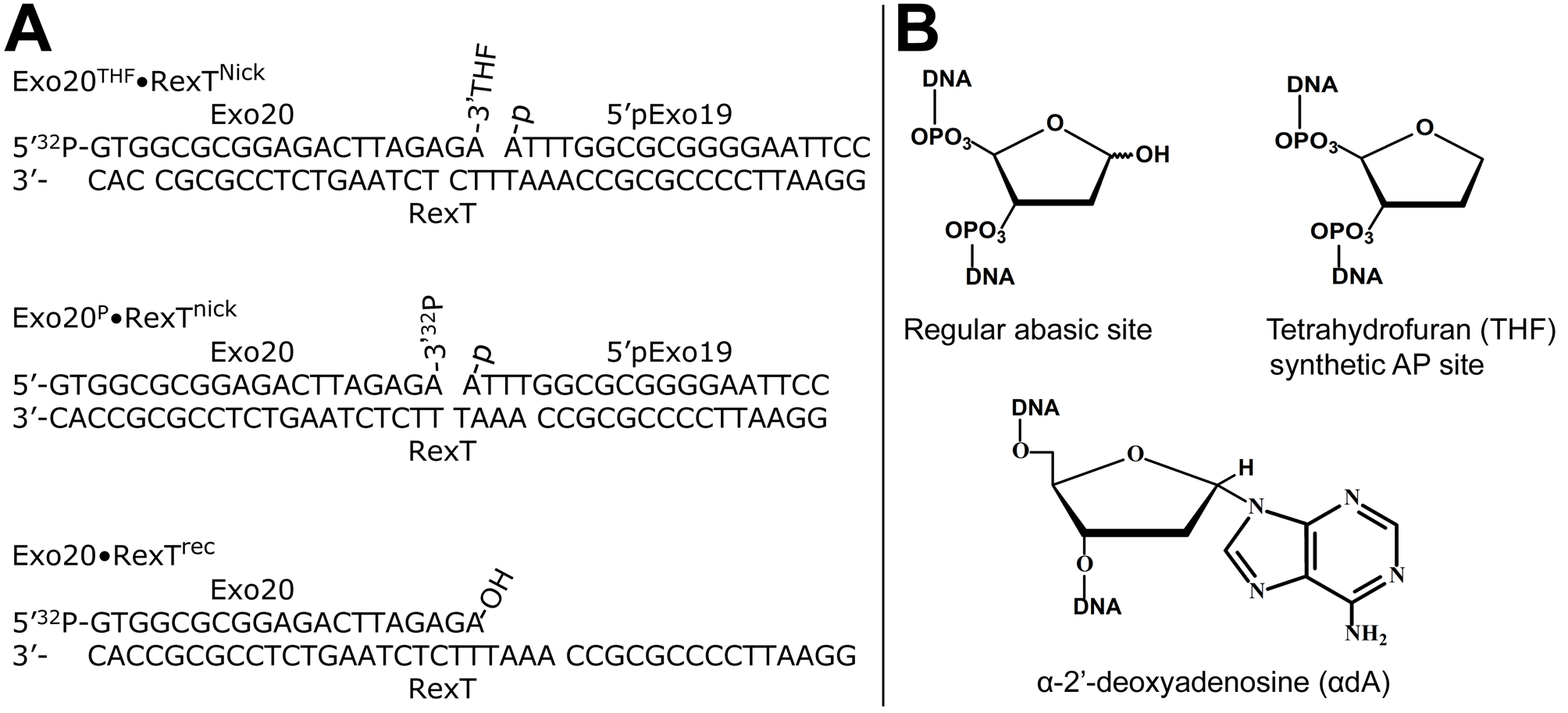

Supplement: S1 Fig — (A) Duplex oligonucleotides used to measure 3’-repair phosphodiesterase and 3’-5’ exonuclease activities. (B) Chemical structures of abasic sites and alpha-anomeric 2’deoxyadenosine. (TIF) [file pone.0202232.s003.tif]

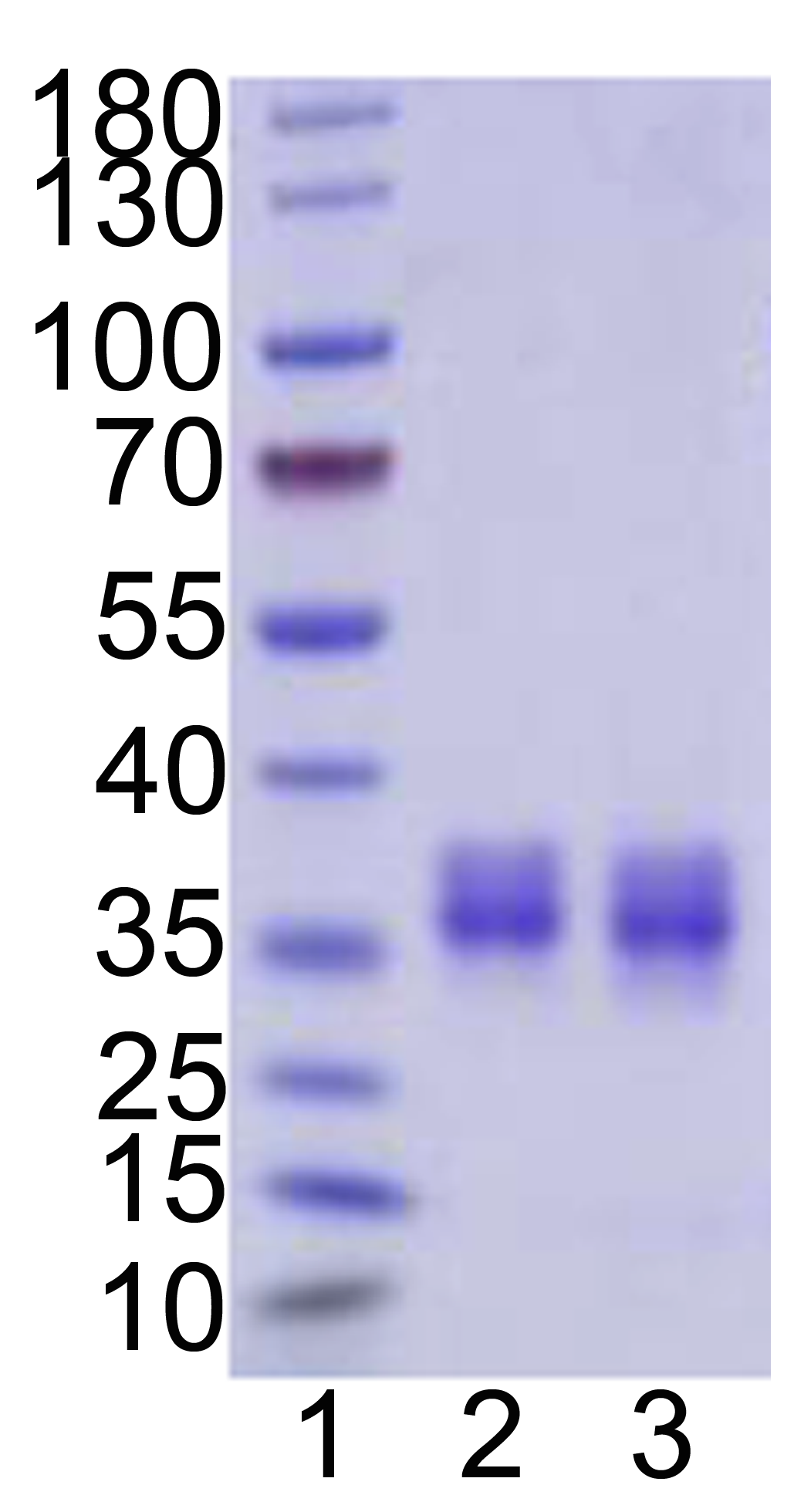

Supplement: S2 Fig — Lane 1, Protein Ladder (Thermo Scientific, cat. # 26616); lane 2, 1 μg HpXth; lane 3, 1 μg HpXth-D144N. For details, see Materials and methods. (TIF) [file pone.0202232.s004.tif]

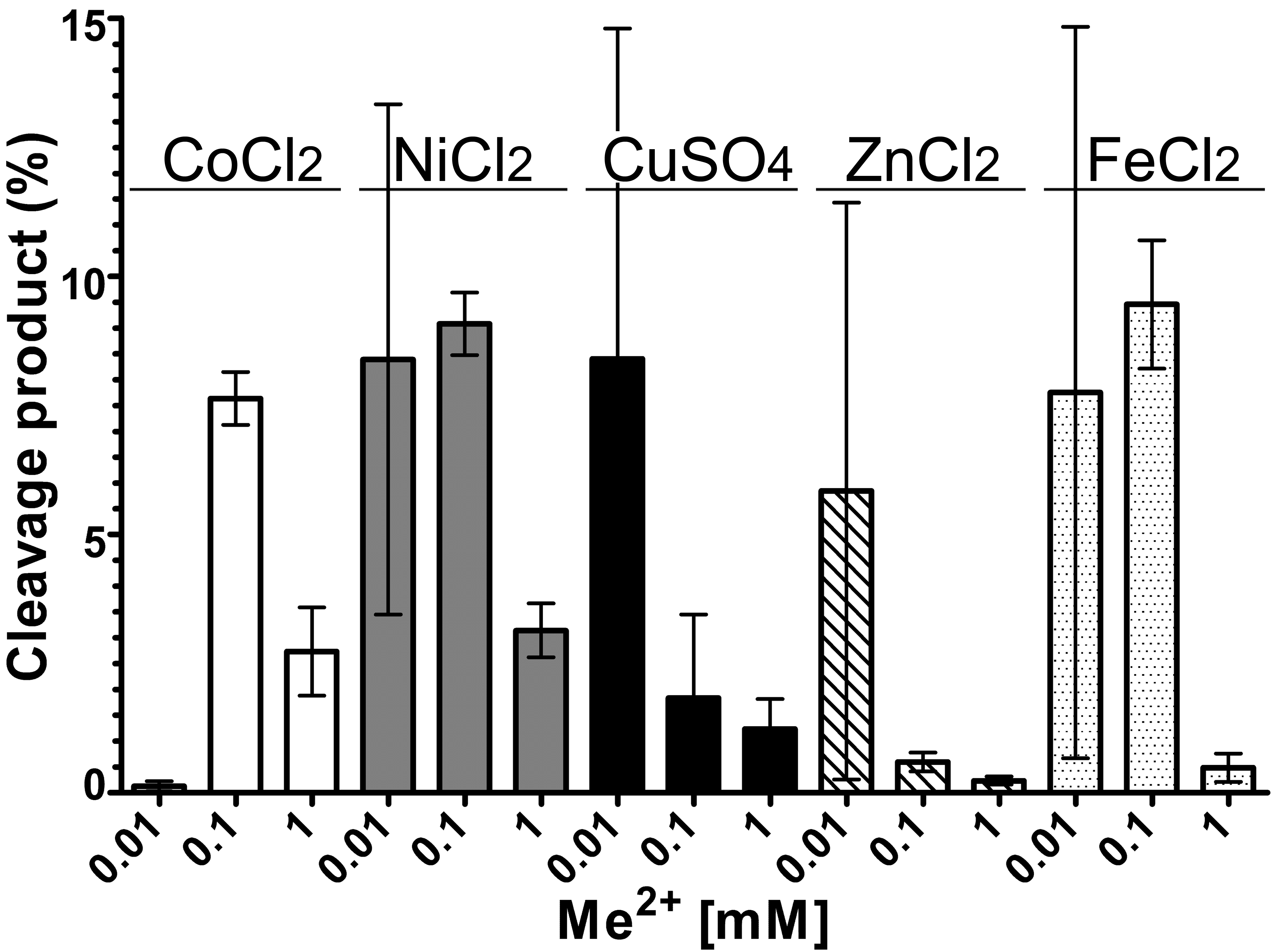

Supplement: S3 Fig — Briefly, 10 nM 5’-[32P]-labeled 30mer THF•T duplex was incubated with 0.2 nM HpXth for 5 min at 37 °C in the buffer containing various concentrations of metal cations. Products of the reaction were resolved by denaturing PAGE, then visualized by phosphorimaging and quantified in ImageQuant. For details, see Materials and methods. (TIF) [file pone.0202232.s005.tif]

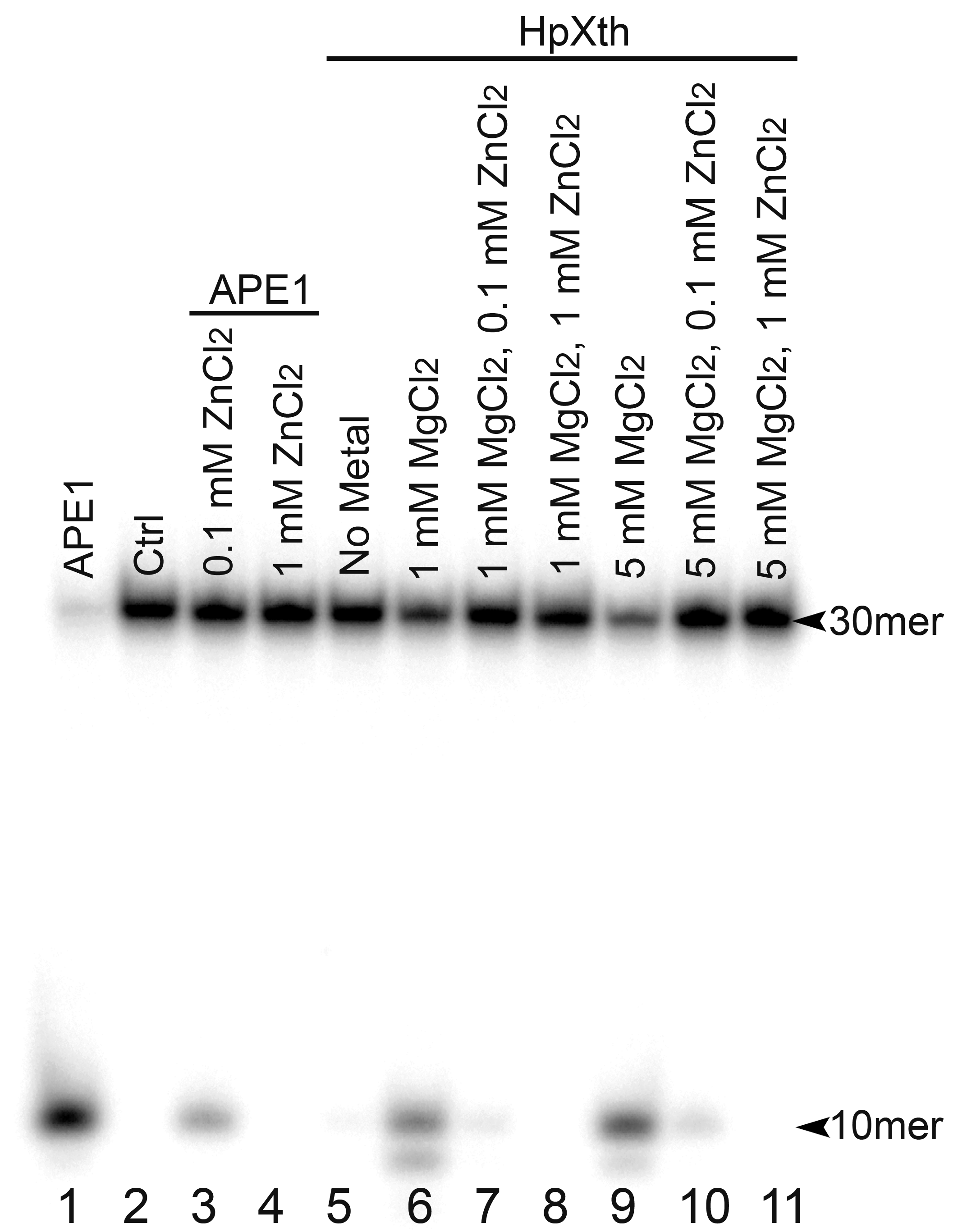

Supplement: S4 Fig — In brief, 10 nM 5’-[32P]-labeled 30mer THF•T duplex was incubated with 0.2 nM HpXth for 5 min at 37 °C in the standard reaction buffer but with varying concentrations of MgCl2 and ZnCl2, unless otherwise stated. Lane 1, THF•T and 0.1 nM APE1 in the buffer BER; lane 2, control THF•T, no enzyme; lanes 3–4, as in lane 1 but supplemented with 0.1 and 1 mM ZnCl2, respectively; lane 5, THF•T and 0.2 nM HpXth in the buffer without metal cations; lane 6, as in lane 5 but 1 mM MgCl2; lane 7, as in lane 6 but 0.1 mM ZnCl2; lane 8, as in lane 6 but 1 mM ZnCl2; lane 9, as in lane 5 but 5 mM MgCl2; lane 10, as in lane 9 but 0.1 mM ZnCl2; lane 10, as in lane 9 but 1 mM ZnCl2. The arrows denote the position of the 30mer substrate and a 10mer cleavage product. For details, see Materials and methods. (TIF) [file pone.0202232.s006.tif]

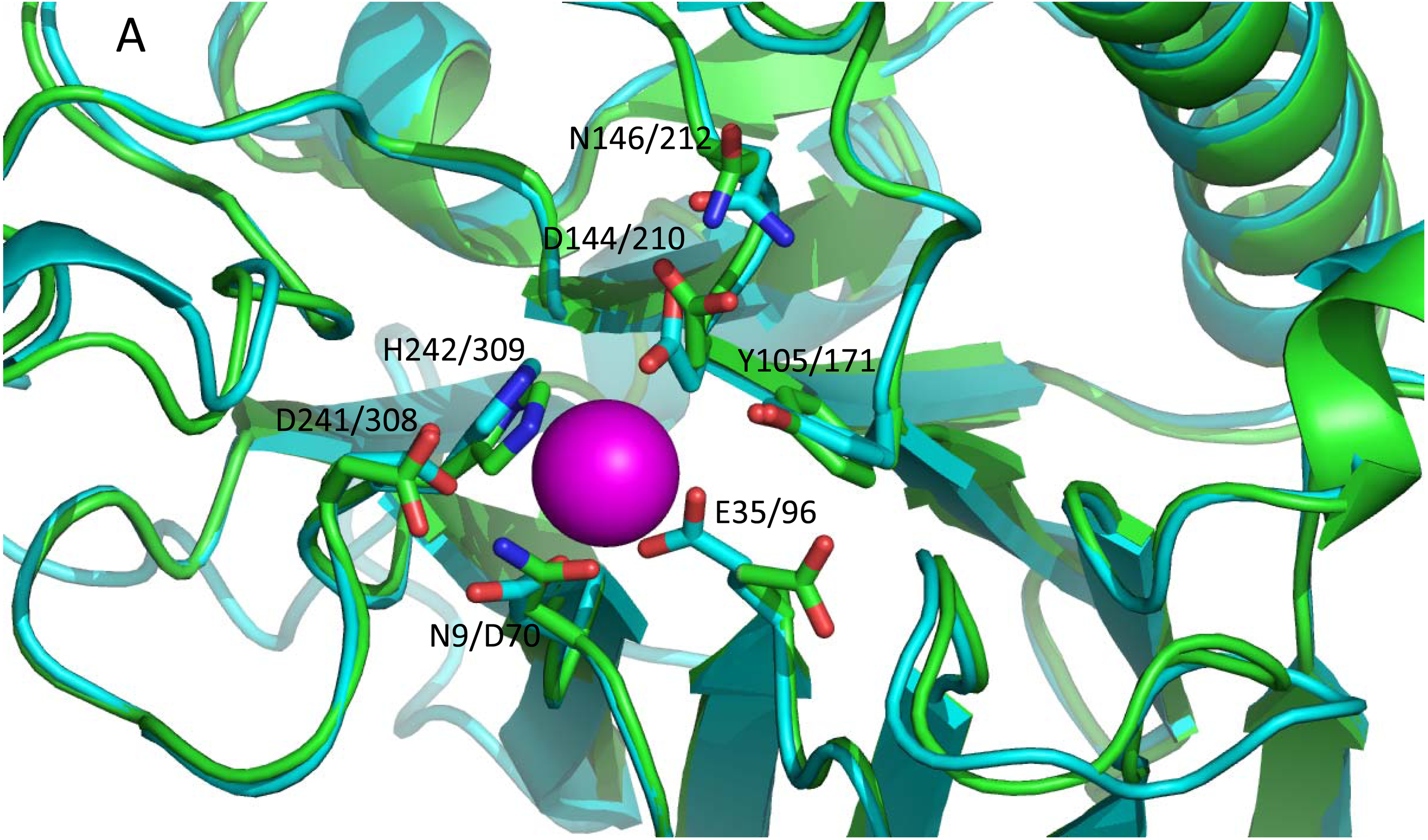

Supplement: S5 Fig — HpXth model superimposed over human APE1 structure (1BIX). Sm3+ ion replacing catalytic Mg2+ in the crystal structure is shown as a magenta ball. Amino acids labels show numbering in HpXth first, followed by numbering in the corresponding human or E. coli protein. Cartoon traces and carbon atoms of HpXth model are colored green, those of human and E. coli structures are cyan, oxygen and nitrogen atoms are red and blue, respectively. (TIF) [file pone.0202232.s007.tif]

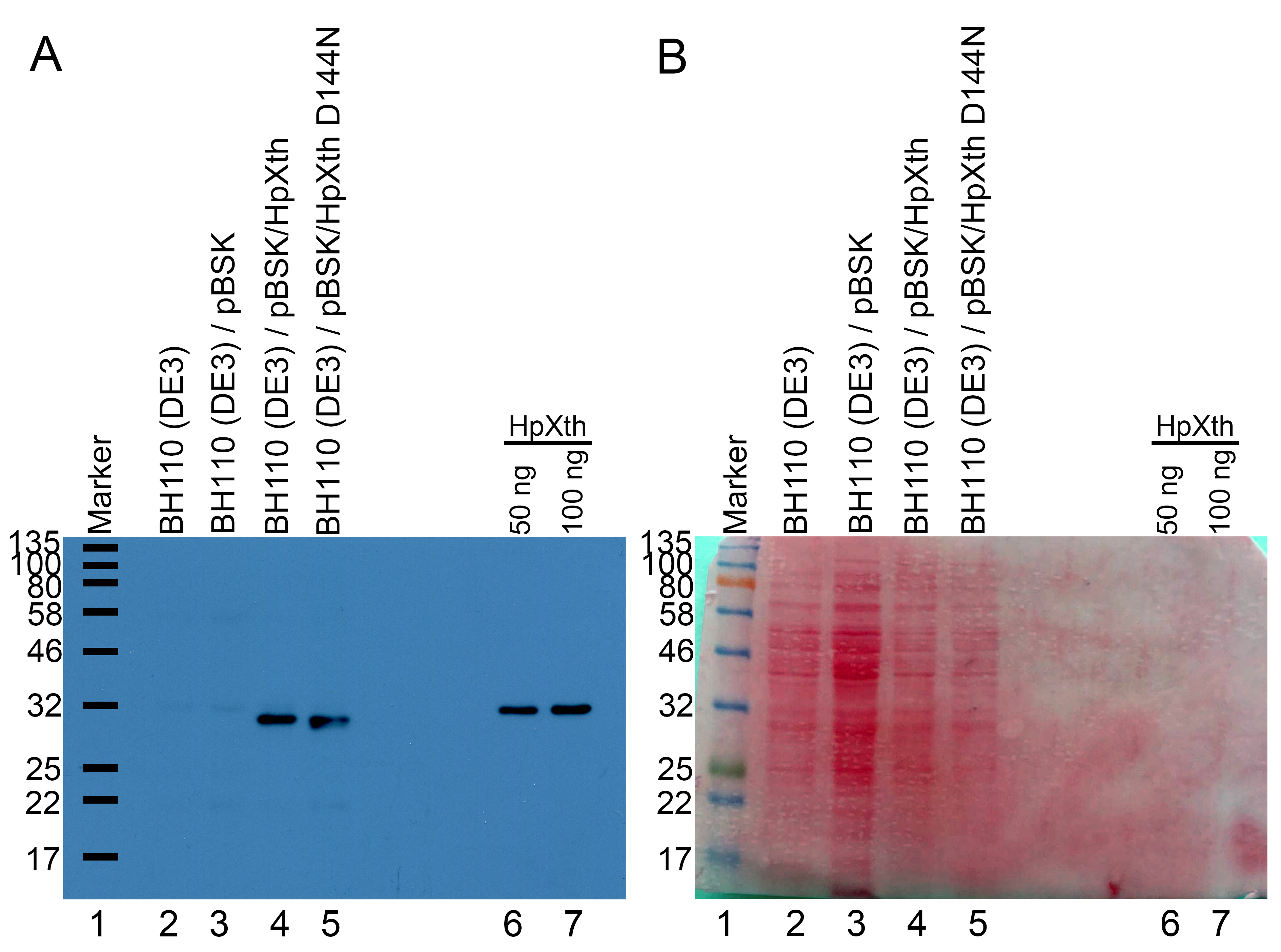

Supplement: S6 Fig — Four μg of cell-free extracts from the BH110 (DE3) strains carrying different plasmids were separated using 12% SDS-PAGE and transferred to the PVDF membrane and then analyzed by Western blotting. (A) Western blot analysis of the PVDF membrane using rabbit anti-HpXth polyclonal antiserum. Lane 1, protein molecular weight markers; lane 2, control plasmid-free E. coli BH110 (DE3) strain; lane 3, empty vector pBluescript II SK+ (pBSK); lane 4, pBSK-HpXth; lane 5, pBSK-HpXth-D144N; lane 6, 50 ng of the purified His-tagged HpXth; lane 7, as 6 but 100 ng. (B) Ponceau S-staining of the PVDF membrane. Lanes 1–7, same as in panel A. For details, see Materials and methods. (TIF) [file pone.0202232.s008.tif]

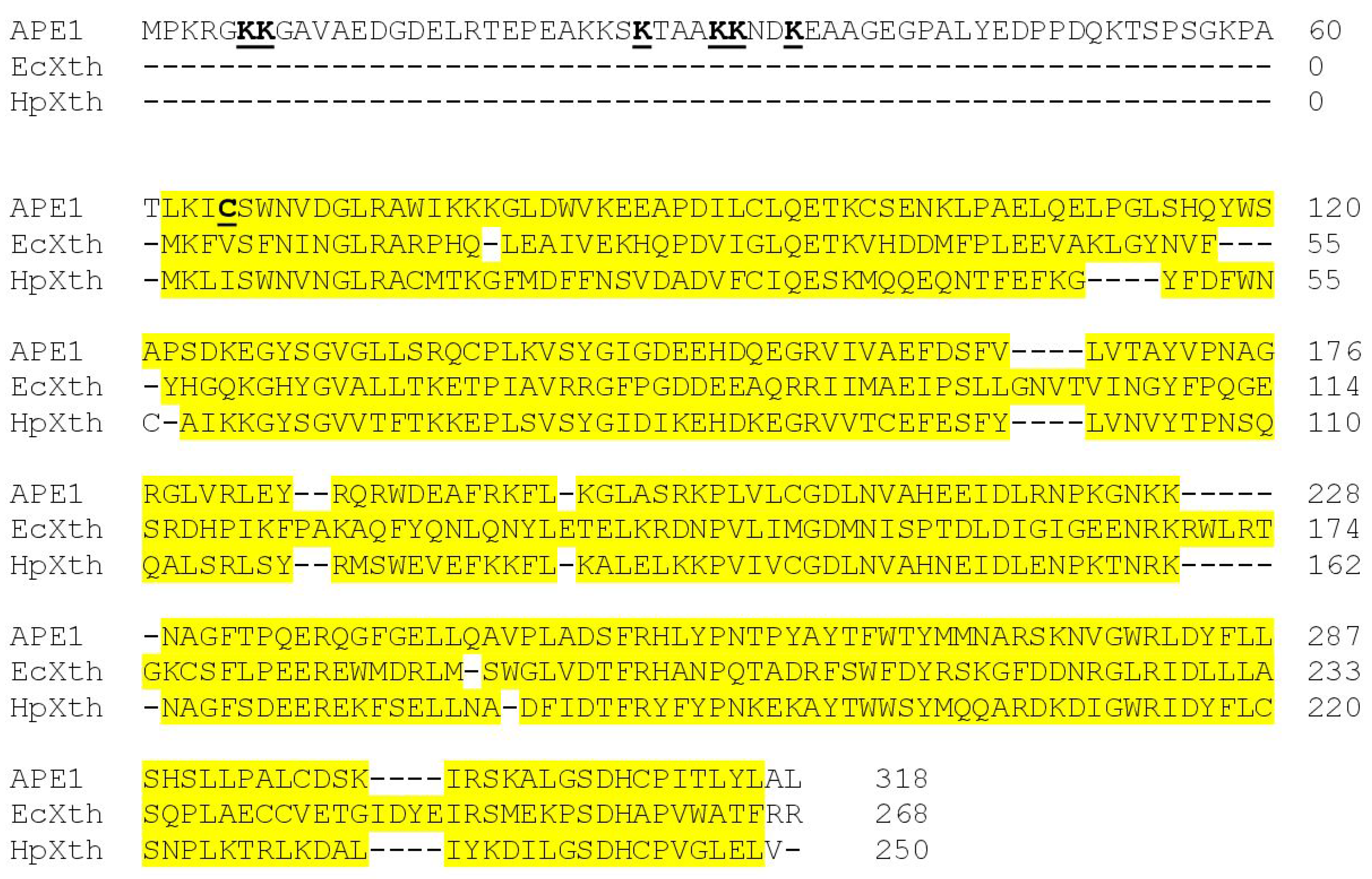

Supplement: S7 Fig — (TIF) [file pone.0202232.s009.tif]
